# Species conservation profiles of tarantula spiders (Araneae, Theraphosidae) listed on CITES

**DOI:** 10.3897/BDJ.7.e39342

**Published:** 2019-11-08

**Authors:** Caroline Fukushima, Jorge Ivan Mendoza, Rick C. West, Stuart John Longhorn, Emmanuel Rivera, Ernest W. T. Cooper, Yann Hénaut, Sergio Henriques, Pedro Cardoso

**Affiliations:** 1 Laboratory for Integrative Biodiversity Research (LIBRe), Finnish Museum of Natural History, University of Helsinki, Helsinki, Finland Laboratory for Integrative Biodiversity Research (LIBRe), Finnish Museum of Natural History, University of Helsinki Helsinki Finland; 2 Institute of Biology, National Autonomous University of Mexico, Mexico City, Mexico Institute of Biology, National Autonomous University of Mexico Mexico City Mexico; 3 Independent Researcher, Sooke, BC, Canada Independent Researcher Sooke, BC Canada; 4 IUCN SSC Spider & Scorpion Specialist Group, Helsinki, Finland IUCN SSC Spider & Scorpion Specialist Group Helsinki Finland; 5 Arachnology Research Association, Oxford, United Kingdom Arachnology Research Association Oxford United Kingdom; 6 Comisión Nacional para el Conocimiento y Uso de la Biodiversidad (CONABIO), Mexico City, Mexico Comisión Nacional para el Conocimiento y Uso de la Biodiversidad (CONABIO) Mexico City Mexico; 7 E. Cooper Environmental Consulting, Delta, Canada E. Cooper Environmental Consulting Delta Canada; 8 Simon Fraser University, Burnaby, Canada Simon Fraser University Burnaby Canada; 9 Ecosur - El Colegio de la Frontera Sur, Chetumal, Quintana Roo, Mexico Ecosur - El Colegio de la Frontera Sur Chetumal, Quintana Roo Mexico; 10 Institute of Zoology, Zoological Society of London, Regent's Park, London NW1 4RY, London, United Kingdom Institute of Zoology, Zoological Society of London, Regent's Park, London NW1 4RY London United Kingdom; 11 Centre for Biodiversity & Environment Research, Department of Genetics, Evolution and Environment, University College London, Gower Street, London, WC1E 6BT, London, United Kingdom Centre for Biodiversity & Environment Research, Department of Genetics, Evolution and Environment, University College London, Gower Street, London, WC1E 6BT London United Kingdom

**Keywords:** Arachnida, Arthropoda, Central America, extinction risk, IUCN, North America, Red List

## Abstract

**Background:**

CITES is an international agreement between governments to ensure that international trade in specimens of wild animals and plants does not threaten their survival. Regarding spiders, all species listed in CITES are tarantulas. They are included in Appendix II, meaning that they are species that are not necessarily now threatened with extinction but that they may become so unless trade is closely controlled.

Many tarantulas are legally and illegally traded in the pet market and they are one of the most traded invertebrate groups. Originally, the CITES list published in 1995 included all the current species of the genus *Brachypelma* Simon, 1891 plus *Aphonopelma pallidum* (F. O. Pickard-Cambridge, 1897) and the so-called *Aphonopelma albiceps* (Pocock, 1903). After that, some taxonomic changes were done, as well as descriptions of new species in the genus *Brachypelma*. The objective of this paper is to assess the 21 taxonomically valid spider species listed on CITES according to the IUCN criteria, study the general patterns and trends and advise on possible future conservation actions critical for the survival of endangered species.

**New information:**

Amongst all 21 species assessed, 16 had sufficient data on their distribution, ecology and threats to properly understand their current status and suggest possible conservation measures. A decline in the area of occupancy (AOO) and extent of occurrence (EOO) was inferred to almost all species, caused mostly by human activities (urbanisation, roads, agricultural and touristic activities), which often lead to the complete loss of subpopulations across their range. Hurricanes and frequent rising water, which are increasing in frequency due to climate change, can cause decline in habitat quality and consequent change in EOO and AOO of some species and should also be considered when planning conservation actions. Severe fragmentation was detected in 13 species and is therefore one of the most relevant threats to the most endangered *Brachypelma* species and should be made a priority aspect to deal with when proposing conservation actions for the group. Regarding the loss of individuals in wild populations, the main cause seems to be the overharvesting to meet the illegal trade.

The most important conservation actions identified across species include preserving their natural habitat through protected areas, establishing management plans for both the species and their habitats and undertaking systematic monitoring to provide information about population recovery and species re-introduction programmes. In general, we propose to prioritise and support research on the population trends and distribution, as well as on the impact of land use and habitat degradation. Special attention regarding conservation actions and research plans has to be given to the central Pacific coastal area of Mexico, particularly around Guerrero State where five species of *Brachypelma* occur. Critically, for some of the most endangered species, such as *B. baumgarteni* and *B. hamorii*, there is no official protected area in their range of occurrence. It would therefore be highly recommended to establish at least one conservation unit which focuses on protecting each of these species *in situ.* In some cases, basic taxonomic research is needed before development of any appropriate conservation action can be proposed.

## Introduction

International wildlife trade, legal or illegal, is a serious threat to biodiversity, both at the source, with the depletion of natural populations through overharvesting and at the destination, as many species can become invasive with negative impact on native communities or be vectors for infectious diseases (Phelps et al. 2010). Illegal wildlife trade, in particular, is one of the most lucrative illicit businesses in the world after drugs, arms and humans trafficking, generating between $7 and $23 billion in revenue annually (Lehmacher 2016). Thus, monitoring and regulating the trade globally is an economic and conservation priority. These roles have largely been played mainly by CITES, the Convention on International Trade of Endangered Species of Wild Fauna and Flora, since 1975.

CITES is an international agreement between governments to ensure that international trade in specimens of wild animals and plants does not threaten their survival (CITES 2019). This agreement is a legal framework for protecting species from overharvesting for international trade (Harfoot et al. 2018) and, despite some criticisms regarding effectiveness (Schonfeld 1985), it is the most important and largest biodiversity conservation agreement, with 183 parties that cooperate to safeguard more than 35,000 different species from overexploitation (CITES 2019).

Within CITES, species are distributed in three different appendices according to the level of protection and requirements imposed to international trade. Appendix I includes species threatened with extinction, whose trade is allowed only in exceptional circumstances (CITES 2019). Appendix II is the most extensive, including the majority of species listed by CITES. It regulates the trade of species not necessarily threatened with extinction but that require management actions to prevent any use incompatible with their survival in the wild (CITES 2019). Appendix III contains species that are protected in at least one country, which has asked other CITES Parties for assistance in controlling the trade (CITES 2019).

Less than 40% of the fauna included in all CITES appendices are invertebrates (CITES 2019), despite being the dominant group amongst multicellular organisms in terms of richness, abundance and often biomass (Cardoso et al. 2011). Regarding spiders, all species listed are tarantulas in Appendix II. Tarantulas are the common name given to spiders that belong to the family Theraphosidae Thorell, 1869. The vast majority can be found in tropical and semi-tropical regions around the world (Bertani 2001), mainly living in burrows in the ground, under rocks and fallen trees. Many tarantulas are legally and illegally traded in the pet market and they are one of the most-traded invertebrate groups. The species traded usually are large, with bright colours, mainly docile and, in general, easy to keep in captivity (Rojo 2004, West 2005). Originally, the list published in 1995 included all the current species of the genus *Brachypelma* Simon, 1891 plus *Aphonopelma pallidum* (F. O. Pickard-Cambridge, 1897) and the so-called *Aphonopelma albiceps* (Pocock, 1903). After that, some taxonomic changes were done such as the synonymy of *Brachypelma annitha* Tesmoingt, Cleton & Verdez, 1997 and *Brachypelma ruhnaui* Schmidt, 1997 with other *Brachypelma* species, the transfer of *A. albiceps* to *Brachypelma* and the transfer of two *Brachypelma* species to *Sericopelma* Ausserer, 1875 (World Spider Catalog 2019). Adding to this, four new species were described (World Spider Catalog 2019). However, to the present assessments, we have considered all the species included in the CITES list, but with their respective taxonomic changes according to the World Spider Catalog (2019): *A. pallidum*, all the 17 species currently included in *Brachypelma* and the three other species that were originally listed as *Brachypelma* in CITES Appendix II and now are in the genera *Sericopelma* or *Sandinista* Longhorn & Gabriel, 2019, totalling 21 species.

A taxonomic review of the genus *Brachypelma* is being conducted by Mendonza and Francke (in press), but currently it comprises 17 species and is present in about one third of the national territory of Mexico (Vilchis-Nestor 2012) and in nearby countries such as Costa Rica and Guatemala (World Spider Catalog 2019), Belize, Honduras and Nicaragua (Reichling 2003, Gabriel and Longhorn 2015, Dor et al. 2008). Although the Mexican territory represents only 1% of the earth's surface, more than 10% of the world's species can be found there (CONABIO and SEMARNAT 2009). Regarding tarantula biodiversity, Mexico is the second most diverse country in the world (Candia-Ramírez and Francke 2017), with a rate of endemism higher than 90% (Rojo 2004). Mexican *Brachypelma* species are no exception to this case.

As for most tarantulas, *Brachypelma* species often have relatively restricted distributions, occupying small areas with specific soil types and are found only sporadically across their ranges (Mendoza and Francke 2017). Thus, they are extremely vulnerable to both natural fluctuations and unpredictable events (Reichling 2003) and to human alterations of the natural environment. Adding to this, other aspects, such as the high mortality of immatures, late sexual maturity (Locht et al. 1999) and their exploitation for the pet trade (Rojo 2004), can cause decline in natural populations.

So far, as with other theraphosid spiders, including *Aphonopelma*, *Sericopelma* and *Sandinista* species, very little is known about the conservation status of Mexican *Brachypelma*, especially those restricted to areas particularly vulnerable to human disturbance, such as deforestation and overharvesting (Mendoza and Francke 2017). The assessment of the conservation status of the spider species listed by CITES is a first and essential step to promote their conservation and can be done using the criteria proposed by the IUCN (2019).

The IUCN, The International Union for Conservation of Nature (www.iucn.org), is the world’s largest environmental network, with 1,300 member organisations that relies on the input of about 16,000 experts. Amongst its many outputs, the Red List of Threatened Species (www.iucnredlist.org) is the most widely known and used by researchers, politicians and the general public. The IUCN Red List is arguably the most useful worldwide list of species at risk of extinction (De Grammont and Cuarón 2006, Lamoreux et al. 2003, Mace et al. 2008, Rodrigues et al. 2006) and whose usefulness is based on its reliance on a number of objective criteria (IUCN 2019). Threatened species are assessed as either Critically Endangered (CR), Endangered (EN) or Vulnerable (VU), but extinct or non-threatened species are also assessed and listed. Besides extinction risk assessment, the Red List provides a plethora of useful information on each included species, including distribution, range and population trends, threats and conservation actions. The quantity and quality of this information allows the Red List to be used in multiple ways, such as to raise awareness about threatened species, guide conservation efforts and funding, set priorities for protection, measure site irreplaceability and vulnerability, influence environmental policies and legislation and evaluate and monitor the state of biodiversity (Lamoreux et al. 2003, Mace et al. 2008, Rodrigues et al. 2006). It therefore constitutes a good basis to assess the current population trends of species listed by CITES and, eventually, to suggest additions, modifications or deletions of species in this agreement.

Only about 31% of the fauna included in the Red List are invertebrate species and 0.37% of the total number of animals are arachnids (IUCN 2019). To date, there are 19 theraphosid species in the Red List of Threatened Species, most of them Asian (IUCN 2019). Out of 19, 12 species are in threatened categories (Critically Endangered, Endangered or Vulnerable), four species in each of them (IUCN 2019). Just one of the species currently assessed is listed by CITES (2019): *Brachypelma smithi* (F. O. Pickard-Cambridge, 1897), known in the pet trade as the Mexican redknee tarantula. Yet, this species was evaluated in 1996 according to previous criteria and its assessment is no longer comparable with the current assessments, thereby requiring an urgent update. It has been traded in large numbers since the 1970s and, although bred in captivity, they continue to be smuggled out of the wild in large numbers (Mendoza and Francke 2017).

The objective of this paper is to assess the 21 taxonomically valid spider species listed on CITES according to the IUCN criteria (IUCN 2019), study the general patterns and trends and advise on possible future conservation actions critical for the survival of endangered species.

## Methods

Biological, population, range and habitat information on 21 taxonomically valid tarantula species (World Spider Catalog 2019) listed on CITES (2019) were collected. Species data were collected from all bibliography available plus unpublished records from the authors. Adding to this, personal communications and information obtained during the Tarantula Trinational Trade and Enforcement Workshop (TTTEW), promoted by the Commission for Environmental Cooperation (CEC) and held in Guadalajara, Jalisco State, Mexico (26 February - 3 March 2018), were also considered. Records from online platforms, such as the UNAM collection (https://datosabiertos.unam.mx/) and iNaturalist (2018) that were verified and endorsed by the authors, were also included. For each record, a spatial coordinate and respective error were attributed. Unless more recent data were unavailable, only records beginning in 2000 were used, as the taxonomic and distribution uncertainty of previous data is high for most species.

For all analyses, we used the R package red - IUCN redlisting tools (Cardoso 2017). This package performs a number of spatial analyses, based on either observed occurrences or estimated ranges. Functions include calculating Extent of Occurrence (EOO), Area of Occupancy (AOO) (see definitions on IUCN (2019)), mapping species ranges, species distribution modelling using climate and land cover, calculating the Red List Index for groups of species, amongst others. It outputs geographical range, elevation and country values, maps in several formats and vectorial data for visualisation in Google Earth.

In this work, the EOO and AOO were calculated using species distribution modelling (SDM), using four environmental datasets at a resolution of 30 arc-second (approximately 1x1 km): (1) 19 bioclimatic variables related to the Mexican territory, that were reduced to two after a Principal Components Analysis (the two first axes); (2) soil type; (3) vegetation type; and (4) ecoregions (see CONABIO (2012) for details on all variables).

After modelling, to avoid overestimation of values and following the precautionary principle, maps were subject to two filtering procedures. First, isolated patches outside the original distribution polygon were excluded from maps. Second, maps were filtered by known distribution ranges according to extensive expertise and field experience from the authors present at TTTEW. All final maps and values were checked and validated by our own expert opinion. The KML files derived from these maps were also produced using the red R package (Cardoso 2017). In addition, the values of AUC of the models were reviewed and they were between 0.81 and 0.98 and all TSS values between 0.73 and 0.96.

To evaluate the severe fragmentation criterion of IUCN, within the potential distribution map of each species, low quality habitat areas (LQH) were identified, based on the map of land use generated by INEGI (2016). Areas with anthropogenic disturbance (crop, urban, water bodies and areas with no vegetation cover) were defined as areas of LQH. Additionally, given that the main threat to tarantulas is the collection for international trade, the experts who attended the Workshop defined that, around the cities, the area of influence of this threat ranges from 1 to 10 km beyond each city border and recommended to take it into account with a buffer of 2 km around the LQH area. Additionally, considering that tarantulas have low dispersion capacity (Reichling 2000, Dor and Hénaut 2012, Janowski-Bell and Horner 1999, Yáñez and Floater 2000), we assumed that populations in LQH will have reduced probability of recolonisation. As an outcome of low quality areas, higher collection probability and low dispersal capacity, we assumed that populations within LQH are small and have low viability.

Following the IUCN guidelines, the relative probability values predicted by SDM were used as a proxy of the density of each species within its distribution range. The area of each polygon of LQH was weighted by the SDM-predicted value (km2*, its corresponding SDM probability). In this way, the next ratio gives the relative abundance of each species in LQH (RA_LQH_):

\begin{varwidth}{50in}
        \begin{equation*}
            RA_{LQH}=\sum(LQ_H)(SDM_L)/(T_H)(SDM_T)
        \end{equation*}
    \end{varwidth}

Where:

LQ_H_ = Area of each low quality polygon within SDM

T_H_ = Area of each polygon within total extension of SDM

SDM_L_ = Relative probability value of low quality polygon within SDM

SDM_T_ = Relative probability value of all polygons within SDM

Thus we considered a species to be severely fragmented if the above ratio was greater than 50%, since it is highly likely that most of its individuals are in isolated subpopulations with low quality habitat and high probability of extinction.

Regarding morphometric data, it should be noted that the sizes of animals given below refer to the 'total body length' taken from the front edge of the chelicerae running longitudinally down the medial body axis to the rear edge of the abdomen, not including the spinnerets.

All assessments were also submitted to IUCN through the Species Information Service (SIS), a web application for conducting and managing species assessments for publication on the IUCN Red List of Threatened species, where the collected information about the taxa are standardised, ensuring the assessments use the same classification systems and can be compared.

## Species Conservation Profiles

### Aphonopelma pallidum

#### Species information

Scientific name: Aphonopelma pallidum

Species authority: (F. O. Pickard-Cambridge, 1897)

Synonyms: *Eurypelma pallidum* F. O. Pickard-Cambridge, 1897 (part); *Brachypelma pallidum* (F. O. Pickard-Cambridge, 1897); *Euathlus pallidus* (F. O. Pickard-Cambridge, 1897); *Avicularia pallida* (F. O. Pickard-Cambridge, 1897); *Rhechostica pallida* (Pickard-Cambridge, 1897).

Common names: Mexican gray; Chihuahua gray, tarántula mexicana gris, tarántula mexicana rosa, mygale grise du Mexique.

Kingdom: Animalia

Phylum: Arthropoda

Class: Arachnida

Order: Araneae

Family: Theraphosidae

**Figure 1.**
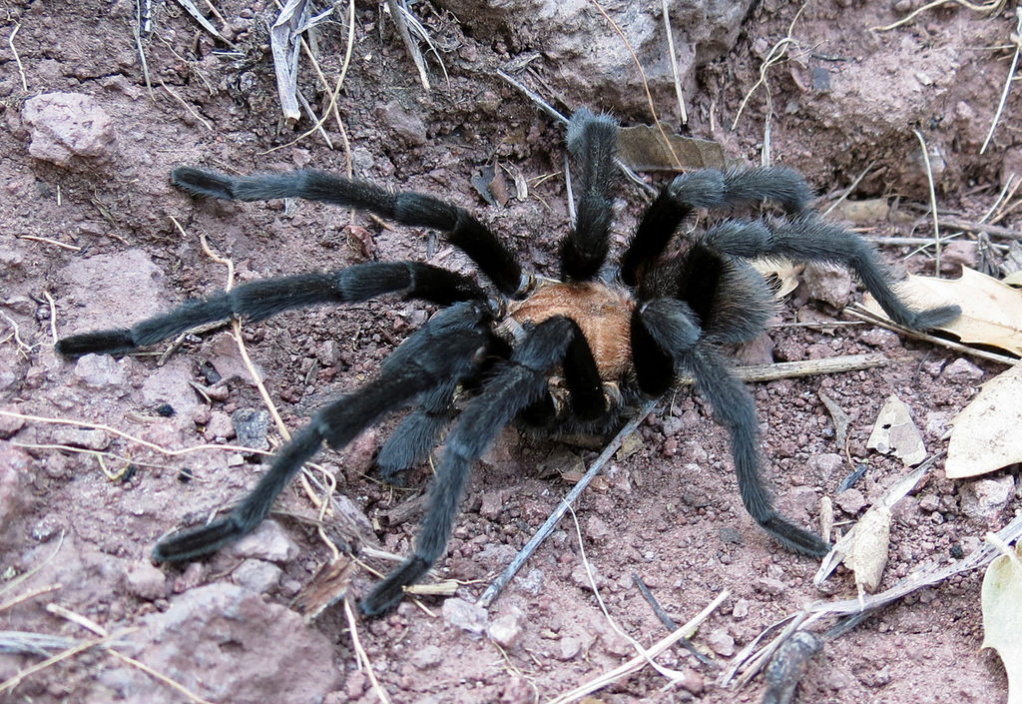
*Aphonopelma pallidum* (F. O. Pickard-Cambridge, 1897) male from east of Nuevo Majalca, Chihuahua state, Mexico. Photo: Fero Bednar.

**Figure 2.**
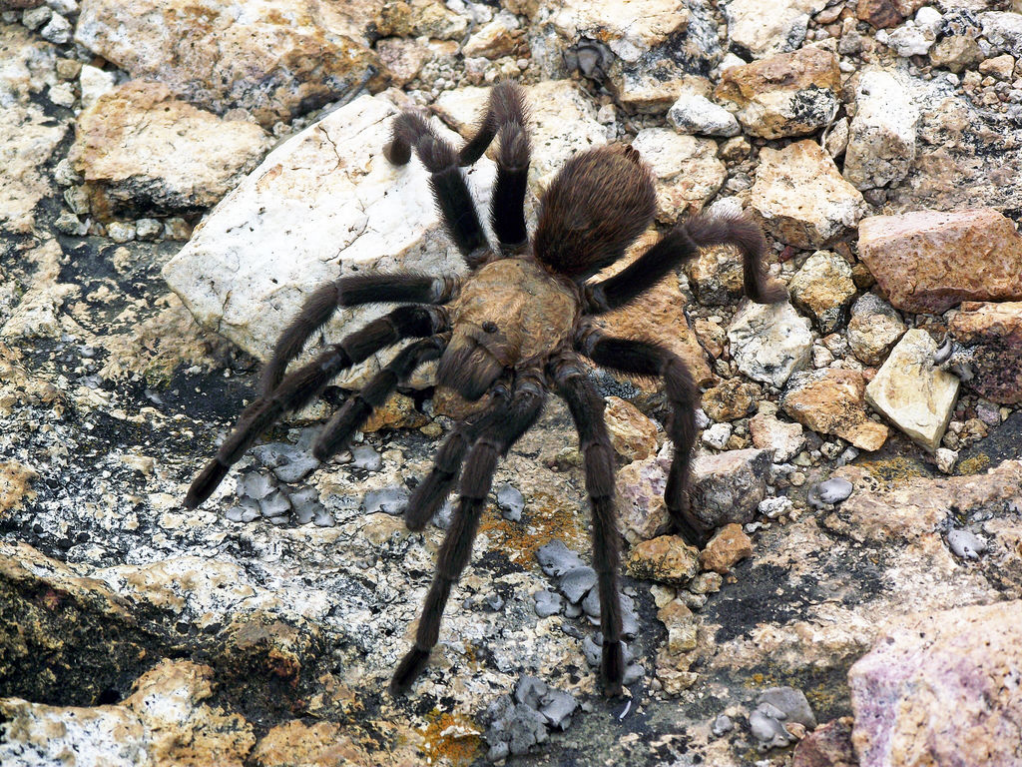
*Aphonopelma pallidum* (F. O. P.-Cambridge, 1897) male with faded colouration from Cumbres de Majalca National Park, Chihuahua state, Mexico. Photo: Alejandra Peña Estrada.

Figure(s) or Photo(s): Figs 1, 2

Region for assessment: Global

#### Geographic range

Biogeographic realm: Nearctic

Countries: Mexico

Map of records (Google Earth): Suppl. material 1

Basis of EOO and AOO: Species Distribution Model

Basis (narrative): Despite few collection sites being recorded for this species, it was possible to perform species distribution modelling to predict its potential range. See methods for details.

Min Elevation/Depth (m): 1205

Max Elevation/Depth (m): 2360

Range description: *Aphonopelma pallidum* is endemic to Mexico and mostly restricted to the highlands of central and southern Chihuahua State (Pickard-Cambridge 1897, Smith 1995, CEC 2017, iNaturalist 2018), with the known range predominantly in the foothills around Chihuahua City. However, there is a small population in the Mapimi Basin in north central Durango State (unpublished data) and the SDMs further extend its potential range to surrounding areas.

#### New occurrences

#### Extent of occurrence

EOO (km2): 200072

Trend: Stable

Causes ceased?: Yes

Causes understood?: Yes

Causes reversible?: Yes

Extreme fluctuations?: No

#### Area of occupancy

Trend: Decline (inferred)

Justification for trend: The AOO of this species, especially around Chihuahua city, is inferred to be declining due to the expansion of urban areas.

Causes ceased?: No

Causes understood?: Yes

Causes reversible?: No

Extreme fluctuations?: No

AOO (km2): 88176

#### Locations

Number of locations: Unknown

Justification for number of locations: Given the large range, the number of locations is much above any thresholds.

Trend: Unknown

Extreme fluctuations?: No

#### Population

Number of individuals: Unknown

Trend: Decline (inferred)

Justification for trend: The population of this species, especially around Chihuahua City, is inferred to be declining due to the expansion of urban areas, causing a decline in AOO.

Basis for decline: (c) a decline in area of occupancy, extent of occurrence and/or quality of habitat

Causes ceased?: No

Causes understood?: Yes

Causes reversible?: No

Extreme fluctuations?: No

Population Information (Narrative): The population of this species, especially around Chihuahua City, is inferred to be declining due to the expansion of urban areas, causing a decline in AOO.

#### Subpopulations

Number of subpopulations: Unknown

Trend: Stable

Extreme fluctuations?: No

Severe fragmentation?: No

Justification for fragmentation: To our knowledge, the species is not subject to severe fragmentation.

#### Habitat

System: Terrestrial

Habitat specialist: No

Habitat (narrative): *Aphonopelma pallidum* inhabits shrubby habitat, with lechuguilla, yucca and creosote shrub with a higher grass ratio than other warm regions to the north and northwest (Smith 1995). The edges of farmland with limited disturbance could provide a good stable habitat for this species, but we lack systematic studies to support this. It can also be found at the fringes of the urban environment but probably just isolated wandering individuals.

Trend in extent, area or quality?: Decline (inferred)

Justification for trend: The habitat of this species, especially around Chihuahua City, is inferred to be declining in area, extent and quality due to the expansion of urban areas.

**Figure 3.**
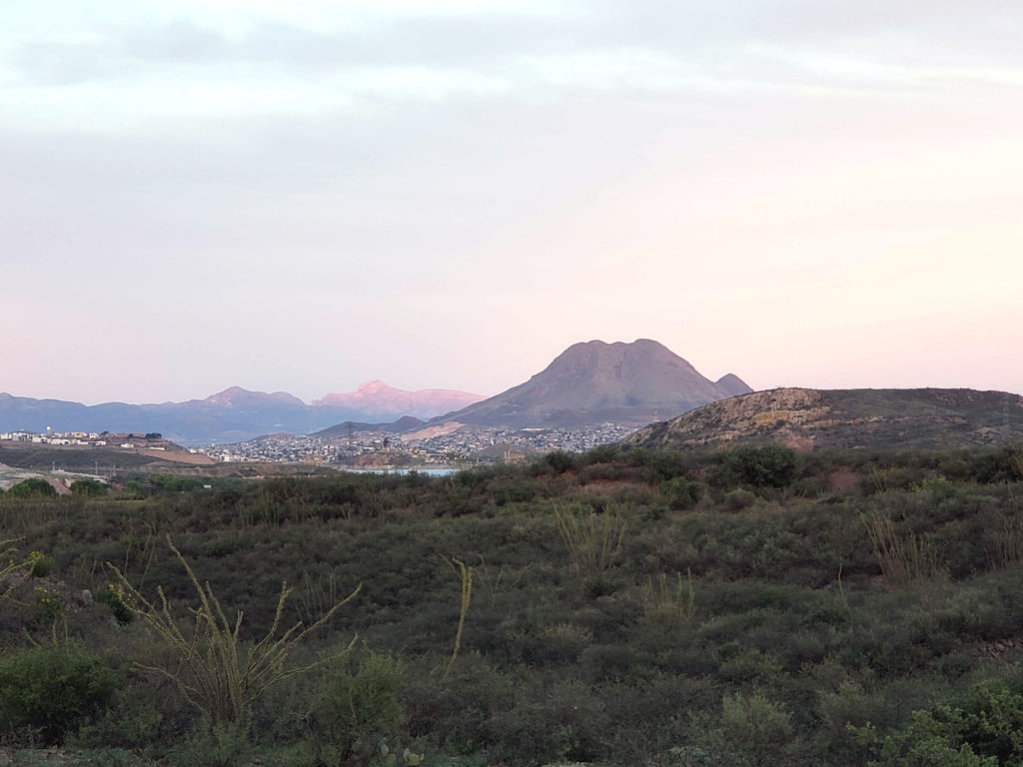
*Aphonopelma pallidum* habitat, Chihuahua state, Mexico. Photo: Alejandra Peña Estrada.

Figure(s) or Photo(s): Fig. 3

##### Habitat

Habitat importance: Major Importance

Habitats: 3.7. Shrubland - Subtropical/Tropical High Altitude

##### Habitat

Habitat importance: Suitable

Habitats: 14.5. Artificial/Terrestrial - Urban Areas

#### Habitat

Habitat importance: Major Importance

Habitats: 3.7. Shrubland - Subtropical/Tropical High Altitude

#### Habitat

Habitat importance: Suitable

Habitats: 14.5. Artificial/Terrestrial - Urban Areas

#### Ecology

Size: 55 mm (female); 43 mm (male).

Generation length (yr): 10

Dependency of single sp?: No

Ecology and traits (narrative): *Aphonopelma pallidum* is a fossorial species that modifies previously excavated burrows or can excavate their own unaided, often found inhabiting clearly defined burrows (hence considered an obligate burrow user). The burrows can be found amongst bushes where they are sheltered from weather conditions and hidden from potential predators, although they can also be found in open areas such as open hillsides. Burrows will typically have a layer of silk around the entrance and can be sealed further with a thin layer of silk across the diameter during daylight that may deter predators (e.g. ants, wasps etc.) and/or help maintain humidity inside the retreat. These spiders are nocturnal predators that wait near the entrance of their refuge from dusk and into the night to feed primarily on ground-dwelling arthropods (insects, other arachnids and some myriapods) or even small vertebrates. Males are seen in the wild during late July to late October (iNaturalist 2018), indicating this as the mating season. The males are likely most active at night, cooler daylight hours and throughout overcast days. Adult females typically moult once per year, just prior to the onset of the annual male emergence. Females will likely produce cocoons (large silken egg sacs) late in the year with young dispersing about a month or two later, but these aspects of reproduction are not yet clearly known in this species.

#### Threats

Justification for threats: The *Aphonopelma pallidum* subpopulation around Chihuahua City is declining due to encroachment by urban development. They are generally deliberately killed when found (especially wandering mature males) or inadvertently crushed by machines during development of land for construction.

##### Threats

Threat type: Ongoing

Threats: 1.1. Residential & commercial development - Housing & urban areas

#### Threats

Threat type: Ongoing

Threats: 1.1. Residential & commercial development - Housing & urban areas

#### Conservation

Justification for conservation actions: The species is present at least in the Cumbres de Majalca National Park. As the subpopulation of *A. pallidum* around Chihuahua city is declining due to expanding urbanisation, it is necessary to protect remaining natural areas where this species is still found, as well as promoting connectivity between them. Adding to this, this species is currently listed on CITES Appendix II and thus its international trade is regulated by an international agreement (CITES 2019).

##### Conservation actions

Conservation action type: In Place

Conservation actions: 1.1. Land/water protection - Site/area protection5.1.1. Law & policy - Legislation - International level

##### Conservation actions

Conservation action type: Needed

Conservation actions: 1.2. Land/water protection - Resource & habitat protection

#### Conservation actions

Conservation action type: In Place

Conservation actions: 1.1. Land/water protection - Site/area protection5.1.1. Law & policy - Legislation - International level

#### Conservation actions

Conservation action type: Needed

Conservation actions: 1.2. Land/water protection - Resource & habitat protection

#### Other

Justification for use and trade: *Aphonopelma pallidum* is currently listed on CITES Appendix II and thus the international trade of this species is regulated by an international agreement (CITES 2019). This species belonged to *Brachypelma* when the genus was included in the CITES Appendix II and remained listed on it despite being posteriorly transferred to another genus (CITES 2019). However, it is not in the international legal trade at least during 2006-2016 (Cooper et al. 2019) and also probably not in the illegal pet trade. During the 1990s when specimens traded under this name were identified taxonomically, they were found to be *B. verdezi* and *A. pallidum* has become absent from the market ever since.

##### Use and trade

Use type: National

Use and trade: 13. Pets/display animals, horticulture

##### Ecosystem services

Ecosystem service type: Less important

##### Research needed

Research needed: 1.2. Research - Population size, distribution & trends3.1. Monitoring - Population trends

Justification for research needed: Since there is little information available about *A. pallidum*, particularly when compared with other *Aphonopelma* species (Hamilton et al. 2016), it is necessary to prioritise and support basic research on the distribution and population size and their trends, mainly on the subpopulation around Chihuahua, to understand the impact of land use and habitat degradation in the population viability of the species.

#### Use and trade

Use type: National

Use and trade: 13. Pets/display animals, horticulture

#### Ecosystem services

Ecosystem service type: Less important

#### Research needed

Research needed: 1.2. Research - Population size, distribution & trends3.1. Monitoring - Population trends

Justification for research needed: Since there is little information available about *A. pallidum*, particularly when compared with other *Aphonopelma* species (Hamilton et al. 2016), it is necessary to prioritise and support basic research on the distribution and population size and their trends, mainly on the subpopulation around Chihuahua, to understand the impact of land use and habitat degradation in the population viability of the species.

#### Viability analysis

### Brachypelma albiceps

#### Species information

Scientific name: Brachypelma albiceps

Species authority: Pocock, 1903

Synonyms: *Eurypelma pallidum* F. O. Pickard-Cambridge, 1897 (part); *Aphonopelma albiceps* (Pocock, 1903); *Brachypelmides ruhnaui* Schmidt, 1997; *Brachypelmides albiceps* (Pocock, 1903).

Common names: Mexican golden redrump, tarántula dorada de México, mygale dorée à abdomen rouge.

Kingdom: Animalia

Phylum: Arthropoda

Class: Arachnida

Order: Araneae

Family: Theraphosidae

**Figure 4.**
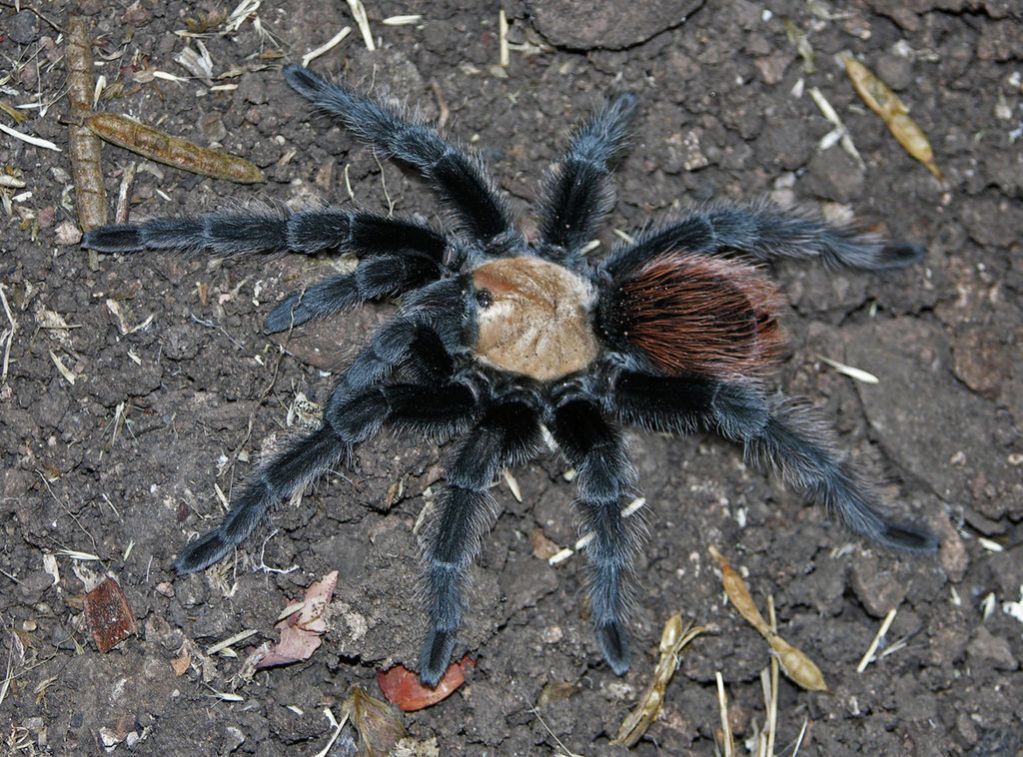
*Brachypelma albiceps* Pocock, 1903 female from Quilamula, Morelos state, Mexico. Photo: Rick C. West.

**Figure 5.**
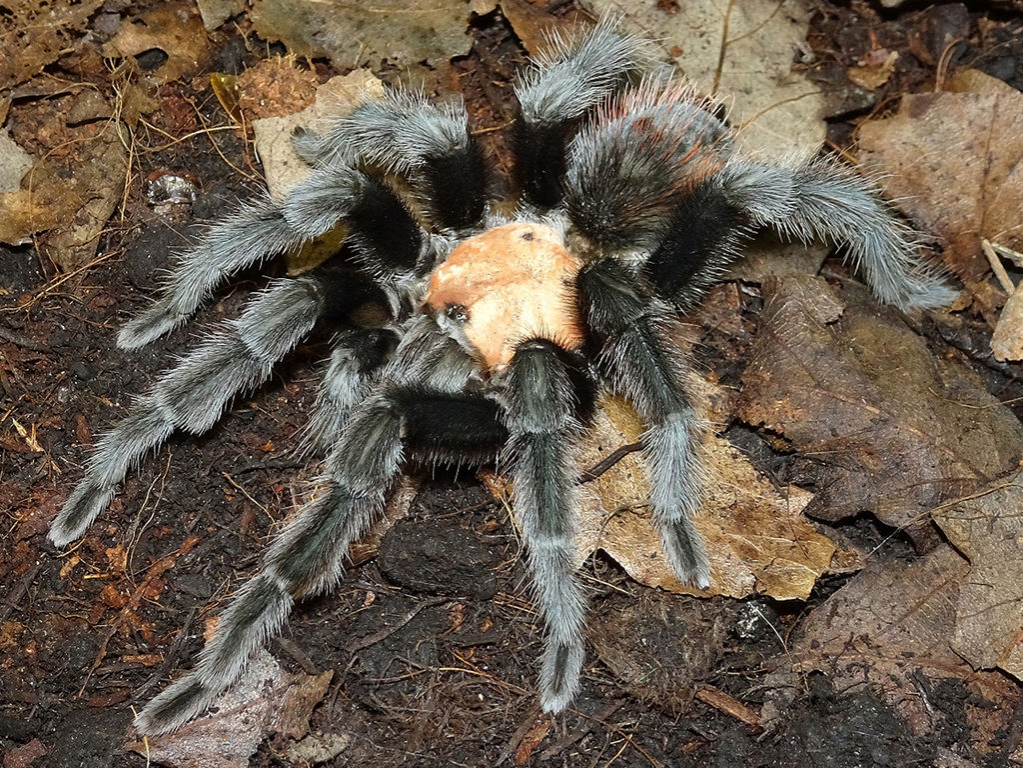
*Brachypelma albiceps* Pocock, 1903 male from Central Guerrero, Mexico. Photo: Guy Tansley.

Figure(s) or Photo(s): Figs 4, 5

Region for assessment: Global

#### Geographic range

Biogeographic realm: Neotropical

Countries: Mexico

**Figure 6.**
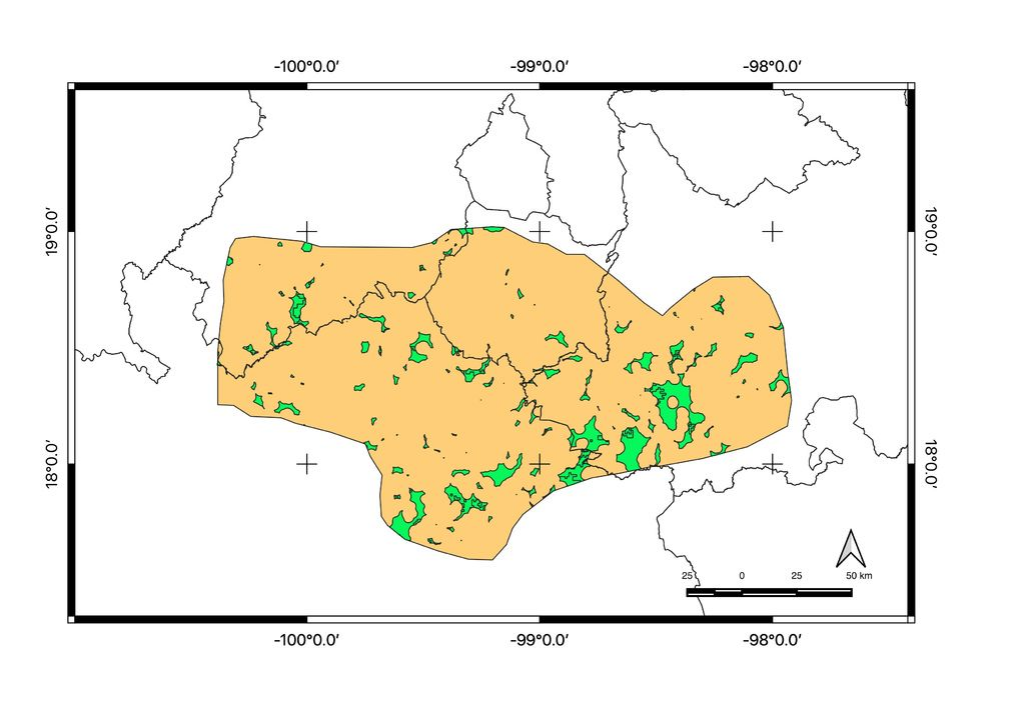
Habitat quality across the distribution of *Brachypelma albiceps* (Pocock, 1903). Low quality habitat areas (LQH) in orange and high quality habitat areas (HQH) in green (for estimation details, see formula in Methods).

Map of records (image): Fig. 6

Map of records (Google Earth): Suppl. material 2

Basis of EOO and AOO: Species Distribution Model

Basis (narrative): A species distribution modelling has been performed to predict its potential range. See methods for details.

Min Elevation/Depth (m): 310

Max Elevation/Depth (m): 2290

Range description: *Brachypelma albiceps* can be found in southern Mexico State, Morelos State, northern and eastern region of Guerrero State (to Chilpancingo area) and western Puebla State (Pickard-Cambridge 1897, Smith 1995, Locht et al. 1999, Estrada-Alvarez 2014, CEC 2017, iNaturalist 2018, unpublished data). Given current data, there is a possibility to find some subpopulations in Michoacán State. *Brachypelma albiceps* is sympatric in Guerrero State with other *Brachypelma* species such as *B. auratum* (at the westernmost known extent of *B. albiceps*) and *B. verdezi* (at the southernmost known limits of *B. albiceps*).

#### New occurrences

#### Extent of occurrence

EOO (km2): 26533

Trend: Decline (inferred)

Justification for trend: Deforestation due to human activities, such as urbanisation, agricultural activities and roads, is causing a decline in EOO. The overharvesting of natural subpopulations to meet the illegal trade can also be causing a decline in EOO if some of the marginal subpopulations are lost.

Causes ceased?: No

Causes understood?: Yes

Causes reversible?: No

Extreme fluctuations?: No

#### Area of occupancy

Trend: Decline (inferred)

Justification for trend: Deforestation due to human activities, such as urbanisation, agricultural activities and roads is causing a decline in AOO. The overharvesting of natural subpopulations to meet the illegal pet trade may also be causing a decline in AOO if some of these subpopulations are lost.

Causes ceased?: No

Causes understood?: Yes

Causes reversible?: No

Extreme fluctuations?: No

AOO (km2): 15996

#### Locations

Number of locations: Unknown

Justification for number of locations: Given the large range, the number of locations is much above that of any category thresholds.

Trend: Unknown

Extreme fluctuations?: No

#### Population

Number of individuals: Unknown

Trend: Decline (inferred)

Justification for trend: The population size is declining due to decrease in AOO and quality of the habitat. Many subpopulations are also being depleted by smugglers to meet the illegal pet trade.

Basis for decline: (c) a decline in area of occupancy, extent of occurrence and/or quality of habitat(d) actual or potential levels of exploitation

Causes ceased?: No

Causes understood?: Yes

Causes reversible?: No

Extreme fluctuations?: No

Population Information (Narrative): Despite the population size being unknown, it is possible to infer its decline since both the AOO and EOO are decreasing, affecting many subpopulations. The overharvesting to meet the illegal trade is also a reason for the population decline in some places of Morelos and Puebla States.

#### Subpopulations

Number of subpopulations: Unknown

Trend: Decline (inferred)

Justification for trend: Deforestation due to human activities, such as urbanisation, agricultural activities and roads, is possibly leading to a decline in the number of subpopulations. The overharvesting of natural subpopulations to meet the illegal pet trade can also be causing some of these to be lost.

Extreme fluctuations?: No

Severe fragmentation?: Yes

Justification for fragmentation: Based on the analysis of the relative abundance of each species in low quality habitat (RA_LQH_) and the Species Distribution Model, 92.48% of the population should be in subpopulations that are non-viable and without possibility of rescue effects due to fragmentation.

#### Habitat

System: Terrestrial

Habitat specialist: No

**Figure 7.**
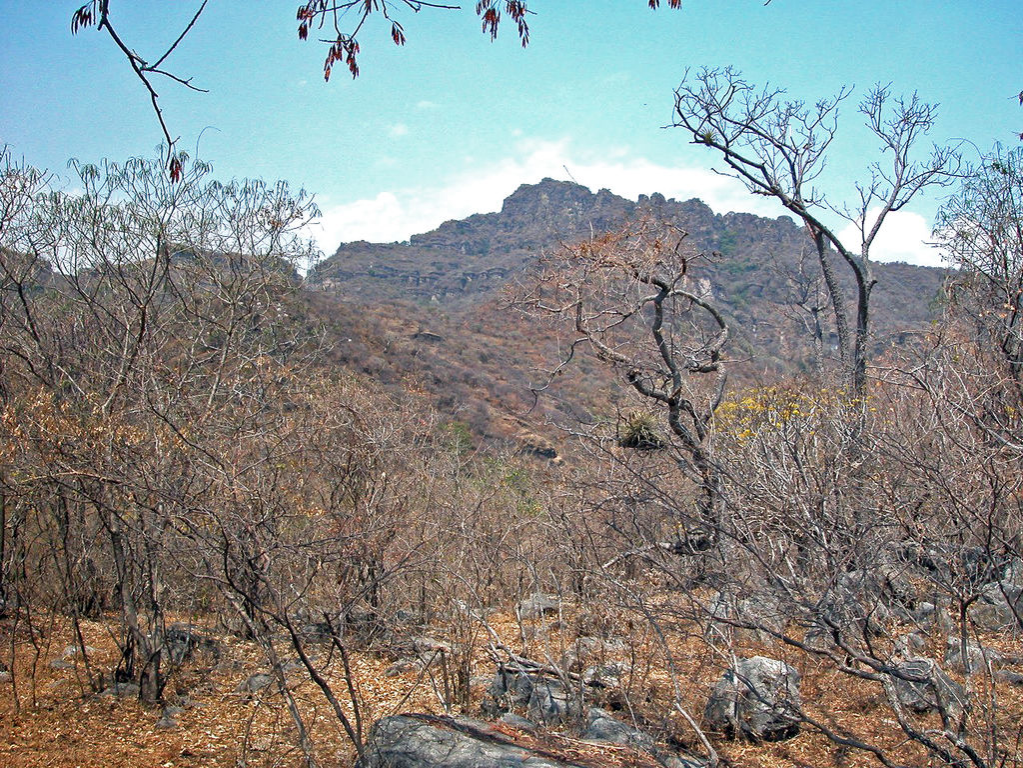
*Brachypelma albiceps* habitat, dry forest, near Tepoztlán, Morelos state, Mexico. Photo: Rick C. West.

Habitat (narrative): *Brachypelma albiceps* primarily inhabits the Balsas dry forest on the inland side of the Sierra Madre del Sur, an area known as Balsas Depression (Fig. 7). Compared to the outer Pacific coast, the inland thorn and dry deciduous forest habitat of the Balsas is seasonally much hotter and drier for several months of the year (Hijmensen 2018). The species can be found up to an altitude of 2,300 metres (above sea level), but prefers low and medium altitudes with a warmer climate. The species can also be found in pine-oak forests, plus sporadically at lower densities around the edges of cultivated areas or in more xeric natural regions.

Trend in extent, area or quality?: Decline (inferred)

Justification for trend: The habitat of this species is inferred to be declining in area, extent and quality (Global Forest Watch 2019), due to human development in particular urbanisation, but also agricultural activities and roads.

Figure(s) or Photo(s): Fig. 7

##### Habitat

Habitat importance: Major Importance

Habitats: 1.5. Forest - Subtropical/Tropical Dry

##### Habitat

Habitat importance: Marginal

Habitats: 14.3. Artificial/Terrestrial - Plantations

#### Habitat

Habitat importance: Major Importance

Habitats: 1.5. Forest - Subtropical/Tropical Dry

#### Habitat

Habitat importance: Marginal

Habitats: 14.3. Artificial/Terrestrial - Plantations

#### Ecology

Size: 70 mm (female); 50 mm (male).

Generation length (yr): 9

Dependency of single sp?: No

Ecology and traits (narrative): *Brachypelma albiceps* is a fossorial species that modifies previously excavated burrows or can excavate their own unaided, either by altering a small natural cavity or by producing a well-defined burrow-like retreat under debris, rocks or large roots in thickets of subtropical dry forest, pine-oak forest or around edges of cultivated fields. In more open areas, they can often make retreats under the rocks supporting fence posts. Burrows mostly do not have any silk around the entrance, giving no clear indication there is a spider inside. The interior can often be multi-tunnelled between 30-40 centimetres in length and ending in a large chamber (West 2005). These spiders are nocturnal predators that wait near the entrance of their refuge from dusk and into the night to feed primarily on ground-dwelling arthropods (insects, other arachnids and some myriapods) or even small vertebrates. Prey remains, discarded around or in burrows, consisted of beetles, millipedes and scorpions. The mating season occurs during the last part of the rainy and first part of dry seasons (August to January) when mature males wander in the open to search for females. The males are likely most active at night, cooler daylight hours and throughout overcast days. Adult females typically moult once per year, just prior to the onset of the annual male emergence. Females will produce cocoons (large silken egg sacs) during the drier winter months with young emerging about two months later, with most young dispersing in the late spring or summer, just before the onset of the early summer rains.

#### Threats

Justification for threats: Urbanisation and the construction of highways are threats to the species as they cause habitat loss and fragmentation. The northern extent of the range across Morelos State appears to be the most severely impacted by human development, particularly massive expansions of urban areas that exterminate multiple subpopulations. Similarly in this zone, throughout Morelos and into adjacent states, increasing agriculture has converted large areas of their former range into various modified land uses (Global Forest Watch 2019) that are completely unsuitable for the species (Turner et al. 2018). Additionally, harvesting to meet trafficking demand is also of concern and the construction of roads facilitates this illegal activity.

##### Threats

Threat type: Ongoing

Threats: 1.1. Residential & commercial development - Housing & urban areas2.2.1. Agriculture & aquaculture - Wood & pulp plantations - Small-holder plantations2.2.2. Agriculture & aquaculture - Wood & pulp plantations - Agro-industry plantations2.3.2. Agriculture & aquaculture - Livestock farming & ranching - Small-holder grazing, ranching or farming2.3.3. Agriculture & aquaculture - Livestock farming & ranching - Agro-industry grazing, ranching or farming4.1. Transportation & service corridors - Roads & railroads5.1.1. Biological resource use - Hunting & trapping terrestrial animals - Intentional use (species is the target)

#### Threats

Threat type: Ongoing

Threats: 1.1. Residential & commercial development - Housing & urban areas2.2.1. Agriculture & aquaculture - Wood & pulp plantations - Small-holder plantations2.2.2. Agriculture & aquaculture - Wood & pulp plantations - Agro-industry plantations2.3.2. Agriculture & aquaculture - Livestock farming & ranching - Small-holder grazing, ranching or farming2.3.3. Agriculture & aquaculture - Livestock farming & ranching - Agro-industry grazing, ranching or farming4.1. Transportation & service corridors - Roads & railroads5.1.1. Biological resource use - Hunting & trapping terrestrial animals - Intentional use (species is the target)

#### Conservation

Justification for conservation actions: There is a critical lack of environmentally protected lands in Guerrero State, which comprises the majority of the range for *Brachypelma albiceps*. The few protected areas of this state are either outside the species range or relatively small and/or of unsuitable habitat. The two exceptions may be the Parque Nacional Grutas de Cacahuamilpa and Biosfera Sierra de Huautla where the occurrence of this species remains to be evaluated, but may be ideal for developing conservation initiatives. Further protected areas need to be established, especially in the lowlands. Important additional conservation actions for *B. albiceps* include establishing management plans and conducting systematic monitoring to inform population recovery and species re-introduction programmes. In order to avoid international trade incompatible with its survival, this species is currently listed on CITES Appendix II, along with *Brachypelma ruhnaui* Schmidt, 1997 (CITES 2019), which was posteriorly considered a junior synonym of *B. albiceps* (Locht et al. 2005). *Brachypelma albiceps* is being reared in captivity in Mexican Units for Management and Sustainable Exploitation of Wildlife (Unidades de Manejo para la Conservación de la Vida Silvestre - UMAs) and sold legally. However, demand in the pet trade still appears to be far higher than numbers currently available through legal trade. As this species is still in the illegal pet trade, both within Mexico and internationally, in comparatively high numbers (relative to legal trade), it is necessary to develop better enforcement actions to curb trafficking, as well as to establish tax advantages for legal dealers in order to make their prices more competitive with the ones in the black market. Finally, regarding public outreach programmes, this species is being reared in captivity in a Mexican Unit for Management (UMA) that also promotes educational and awareness activities about tarantula spiders aimed at students and the general public. Adding to this, *B. albiceps* can be used as a flagship (perhaps most notably for Morelos State) since it is a very attractive species.

##### Conservation actions

Conservation action type: In Place

Conservation actions: 1.1. Land/water protection - Site/area protection3.4.1. Species management - Ex-situ conservation - Captive breeding/artificial propagation5.1.1. Law & policy - Legislation - International level

##### Conservation actions

Conservation action type: Needed

Conservation actions: 1.1. Land/water protection - Site/area protection1.2. Land/water protection - Resource & habitat protection3.1.1. Species management - Species management - Harvest management3.1.2. Species management - Species management - Trade management3.2. Species management - Species recovery3.3.1. Species management - Species re-introduction - Reintroduction5.3. Law & policy - Private sector standards & codes5.4.1. Law & policy - Compliance and enforcement - International level5.4.2. Law & policy - Compliance and enforcement - National level5.4.3. Law & policy - Compliance and enforcement - Sub-national level6.4. Livelihood, economic & other incentives - Conservation payments4.3. Education & awareness - Awareness & communications

#### Conservation actions

Conservation action type: In Place

Conservation actions: 1.1. Land/water protection - Site/area protection3.4.1. Species management - Ex-situ conservation - Captive breeding/artificial propagation5.1.1. Law & policy - Legislation - International level

#### Conservation actions

Conservation action type: Needed

Conservation actions: 1.1. Land/water protection - Site/area protection1.2. Land/water protection - Resource & habitat protection3.1.1. Species management - Species management - Harvest management3.1.2. Species management - Species management - Trade management3.2. Species management - Species recovery3.3.1. Species management - Species re-introduction - Reintroduction5.3. Law & policy - Private sector standards & codes5.4.1. Law & policy - Compliance and enforcement - International level5.4.2. Law & policy - Compliance and enforcement - National level5.4.3. Law & policy - Compliance and enforcement - Sub-national level6.4. Livelihood, economic & other incentives - Conservation payments4.3. Education & awareness - Awareness & communications

#### Other

Justification for use and trade: *Brachypelma albiceps* is being reared in captivity in Mexican Units for Management (UMAs) and sold legally. This species is currently listed on CITES Appendix II and thus its international trade is regulated by an international agreement (CITES 2019). A legal trade of captive-bred live specimens of *B. albiceps* is recorded, but an unquantified amount of wild caught animals illegally traded is also known. Data from 2006-2016 indicates that captive-bred juveniles are legally sold from US$20–$60 in Canada and the United States, for US$9 in the EU and for US$4 in Mexico; adult males are sold for approximately US$50 in Canada and adult females approximately for US$250 in the United States and for US$47 in the EU (CEC 2017). Data from the United Nations Environment Programme World Conservation Monitoring Centre (UNEP-WCMC) showed that, during 2006–2016, about 897 specimens were traded, but this number could be higher, possibly up to 1,219, constituting a common species considering the availability in the international trade (Cooper et al. 2019). All specimens of *B. albiceps* were traded live and recorded as *Aphonopelma albiceps*; none of them was declared as wild-caught and most specimens were traded for commercial purposes (Cooper et al. 2019).

##### Use and trade

Use type: International

Use and trade: 13. Pets/display animals, horticulture16. Establishing ex-situ production *

##### Ecosystem services

Ecosystem service type: Less important

##### Research needed

Research needed: 2.3. Conservation Planning - Harvest & Trade Management Plan3.1. Monitoring - Population trends3.2. Monitoring - Harvest level trends3.3. Monitoring - Trade trends

Justification for research needed: The development of a system for certifying the origin of specimens used in breeding programmes in the Mexican Units for Management (UMAs) should be done in collaboration with the government of Mexico and Mexican tarantula breeders. Systematic monitoring could be undertaken within known subpopulations. Despite healthy subpopulations in southern Morelos State, cursory field surveys indicate that other subpopulations are likely in decline elsewhere; however, there is no rigorous evidence to support this and further studies are needed. Trade trends and how these affect harvest levels must be further studied.

#### Use and trade

Use type: International

Use and trade: 13. Pets/display animals, horticulture16. Establishing ex-situ production *

#### Ecosystem services

Ecosystem service type: Less important

#### Research needed

Research needed: 2.3. Conservation Planning - Harvest & Trade Management Plan3.1. Monitoring - Population trends3.2. Monitoring - Harvest level trends3.3. Monitoring - Trade trends

Justification for research needed: The development of a system for certifying the origin of specimens used in breeding programmes in the Mexican Units for Management (UMAs) should be done in collaboration with the government of Mexico and Mexican tarantula breeders. Systematic monitoring could be undertaken within known subpopulations. Despite healthy subpopulations in southern Morelos State, cursory field surveys indicate that other subpopulations are likely in decline elsewhere; however, there is no rigorous evidence to support this and further studies are needed. Trade trends and how these affect harvest levels must be further studied.

#### Viability analysis

### Brachypelma albopilosum

#### Species information

Scientific name: Brachypelma albopilosum

Species authority: Valerio, 1980

Synonyms: *Brachypelma albopilosa* Valerio, 1980; *Euathlus albopilosus* (Valerio, 1980).

Common names: Curlyhair, Honduran curlyhair (see taxonomic notes).

Kingdom: Animalia

Phylum: Arthropoda

Class: Arachnida

Order: Araneae

Family: Theraphosidae

Taxonomic notes: The known species range comprises two disjunct areas, one in the Caribbean coast of Honduras and another in southern Nicaragua and northern Costa Rica. However, the Honduran subpopulations are currently of unclear taxonomic status and deserve further taxonomic studies, not considered here. Mendonza and Francke (in press) are publishing a taxonomic revision of *Brachypelma* and this species will be accommodated in a new genus.

**Figure 8.**
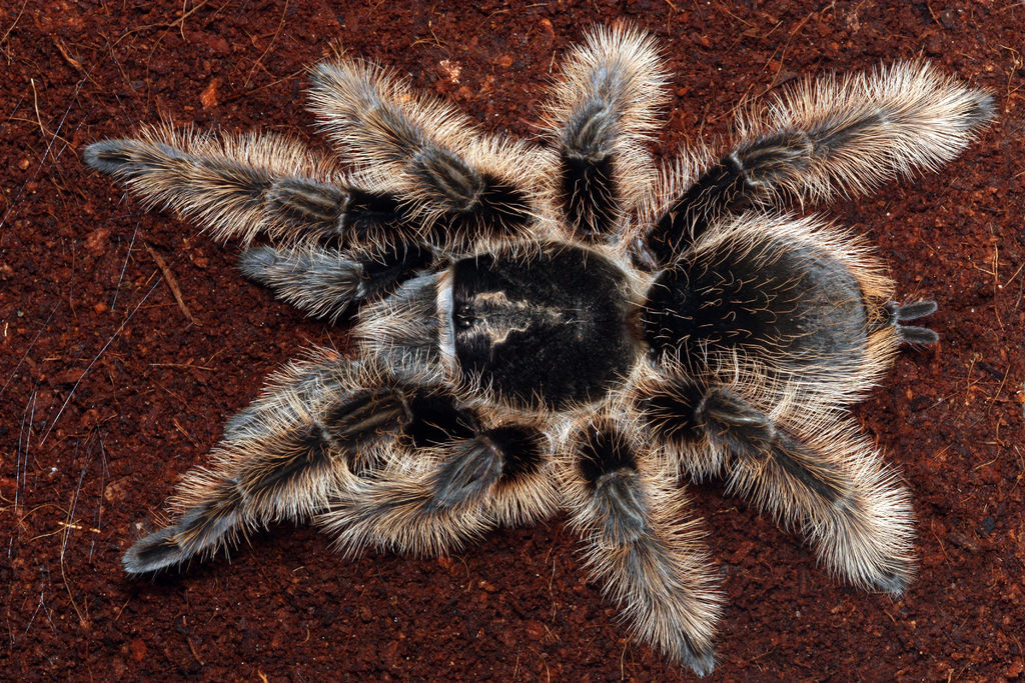
*Brachypelma albopilosum* Valerio, 1980 female from Nicaragua. Photo: Stuart J. Longhorn.

**Figure 9.**
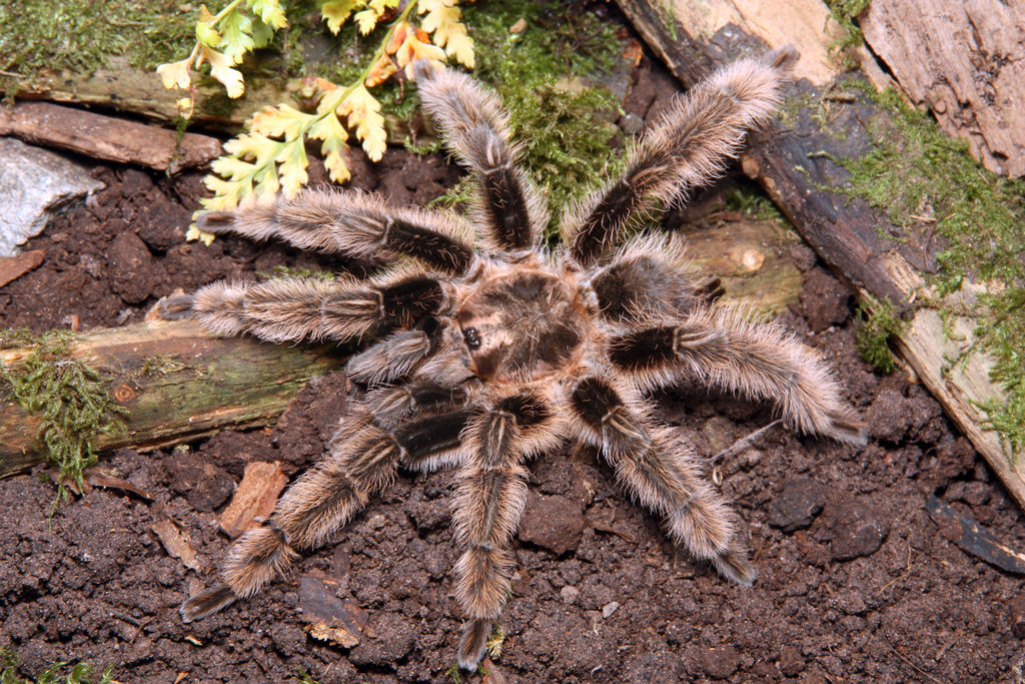
*Brachypelma albopilosum* Valerio, 1980 male from Southern Nicaragua. Photo: Stuart J. Longhorn.

Figure(s) or Photo(s): Figs 8, 9

Region for assessment: Global

#### Geographic range

Biogeographic realm: Neotropical

Countries: Costa RicaNicaragua

Map of records (Google Earth): Suppl. material 3

Basis of EOO and AOO: Species Distribution Model

Basis (narrative): Despite few collection sites recorded for this species, it was possible to perform species distribution modelling to predict its potential range. See methods for details.

Min Elevation/Depth (m): 0

Max Elevation/Depth (m): 1770

Range description: *Brachypelma albopilosum* can be found in northern Costa Rica (Valerio 1980) across the northern part of Province of Alajuela and into south-eastern Nicaragua throughout the Department Río San Juan and into Región Autónoma Costa Caribe Sur (Maes et al. 1989, Maes 1992, unpublished data).

#### New occurrences

#### Extent of occurrence

EOO (km2): 29633

Trend: Decline (inferred)

Justification for trend: A decline in EOO is inferred from habitat loss due to deforestation, urbanisation and agricultural activities.

Causes ceased?: No

Causes understood?: Yes

Causes reversible?: No

Extreme fluctuations?: Unknown

#### Area of occupancy

Trend: Decline (inferred)

Justification for trend: A decline in AOO is inferred from habitat loss due to deforestation, urbanisation and agricultural activities.

Causes ceased?: No

Causes understood?: Yes

Causes reversible?: No

Extreme fluctuations?: Unknown

AOO (km2): 13256

#### Locations

Number of locations: Unknown

Trend: Unknown

Justification for trend: Given the large range, the number of locations is much above that of any category thresholds.

Extreme fluctuations?: No

#### Population

Number of individuals: Unknown

Trend: Decline (inferred)

Justification for trend: A decline in population size is inferred from possible loss of AOO and EOO due to deforestation (Global Forest Watch 2019), urbanisation and agricultural activities within its range. Additionally, the population is harvested for the pet trade in Nicaragua.

Basis for decline: (c) a decline in area of occupancy, extent of occurrence and/or quality of habitat(d) actual or potential levels of exploitation

Causes ceased?: No

Causes understood?: Yes

Causes reversible?: No

Extreme fluctuations?: Unknown

Population Information (Narrative): A decline in population size is inferred from habitat loss due to deforestation (Global Forest Watch 2019), urbanisation and agricultural activities. Additionally, the population is harvested for the pet trade in Nicaragua.

#### Subpopulations

Number of subpopulations: Unknown

Trend: Decline (inferred)

Justification for trend: A decline in number of subpopulations is inferred from habitat loss due to deforestation (Global Forest Watch 2019), urbanisation and agricultural activities. Additionally, the population is harvested for the pet trade in Nicaragua which might cause some subpopulations to be entirely depleted.

Extreme fluctuations?: No

Severe fragmentation?: No

Justification for fragmentation: To our knowledge, the species is not subject to severe fragmentation.

#### Habitat

System: Terrestrial

Habitat specialist: No

Habitat (narrative): *Brachypelma albopilosum* inhabits moist tropical broadleaf forests of Central America, which have been described as “lush, tall tropical evergreen forest of huge, buttressed canopy trees reaching 40m in height and an extremely rich epiphyte flora” (Hijmensen 2018). The species can be found in an altitudinal range from 0-1770 metres (above sea level), but prefers lower altitudes and a warmer climate. It also presents some tolerance to semi-disturbed areas, for example around edges of small towns.

Trend in extent, area or quality?: Decline (inferred)

Justification for trend: The habitat of this species is inferred to be declining in area, extent and quality due to deforestation (Global Forest Watch 2019), mainly due to agricultural activities.

##### Habitat

Habitat importance: Major Importance

Habitats: 1.6. Forest - Subtropical/Tropical Moist Lowland

##### Habitat

Habitat importance: Marginal

Habitats: 14.2. Artificial/Terrestrial - Pastureland14.6. Artificial/Terrestrial - Subtropical/Tropical Heavily Degraded Former Forest

#### Habitat

Habitat importance: Major Importance

Habitats: 1.6. Forest - Subtropical/Tropical Moist Lowland

#### Habitat

Habitat importance: Marginal

Habitats: 14.2. Artificial/Terrestrial - Pastureland14.6. Artificial/Terrestrial - Subtropical/Tropical Heavily Degraded Former Forest

#### Ecology

Size: 70 mm (female); 60 mm (male).

Generation length (yr): 7

Dependency of single sp?: No

Ecology and traits (narrative): *Brachypelma albopilosum* is largely a fossorial species that modifies previously excavated burrows or can excavate their own unaided, sometimes on minor alterations to natural small cavities under debris such as large rocks and tree roots in dense vegetation of moist tropical forest. Others, however, can be found in more clearly defined obligate burrow-like retreats under similar debris or be somewhat adaptable to disturbance and found amongst fallen wood, between rocks or even associated with cavities higher up in trees (Hijmensen 2018). Burrows can have either very little or decent amounts of silk at the burrow entrance to transmit the vibrations of prey movement (if present) and can be sealed with a further thin layer of silk across the diameter during daylight that may deter predators (e.g. ants, wasps etc.) and/or help maintain humidity inside the retreat. The burrows of adults can be steeply inclined by the entrance, but elbowed below to become more horizontal and relatively narrow throughout except for a larger terminal chamber or chambers. These spiders are nocturnal predators that wait near the entrance of their refuge from dusk and into the night to feed primarily on ground-dwelling arthropods (insects, other arachnids and some myriapods) or even small vertebrates. The mating season occurs during the rainy season (June to October) and may continue into the dry season (after December) when mature males wander in the open to search for females. The males are likely most active at night, cooler daylight hours and throughout overcast days. Adult females typically moult once per year, just prior to the onset of the annual male emergence. Females will produce cocoons (large silken egg sacs) during the driest spring months with young emerging about two months later. Most young disperse in the late spring (March-April) or summer, just before the onset of the early summer rains.

#### Threats

Justification for threats: As for the majority of *Brachypelma* species, the reduction in habitat size due to human modification of land use is an important threat. Added to this, within just the last 5 years, *B. albopilosum* has begun to be intensely harvested from the wild in Nicaragua for the pet trade, in large part for registered exports to fill the large demand of major chain pet stores, but also to meet the illegal market.

##### Threats

Threat type: Ongoing

Threats: 1.1. Residential & commercial development - Housing & urban areas2.2.1. Agriculture & aquaculture - Wood & pulp plantations - Small-holder plantations2.2.2. Agriculture & aquaculture - Wood & pulp plantations - Agro-industry plantations2.3.2. Agriculture & aquaculture - Livestock farming & ranching - Small-holder grazing, ranching or farming2.3.3. Agriculture & aquaculture - Livestock farming & ranching - Agro-industry grazing, ranching or farming5.1.1. Biological resource use - Hunting & trapping terrestrial animals - Intentional use (species is the target)

#### Threats

Threat type: Ongoing

Threats: 1.1. Residential & commercial development - Housing & urban areas2.2.1. Agriculture & aquaculture - Wood & pulp plantations - Small-holder plantations2.2.2. Agriculture & aquaculture - Wood & pulp plantations - Agro-industry plantations2.3.2. Agriculture & aquaculture - Livestock farming & ranching - Small-holder grazing, ranching or farming2.3.3. Agriculture & aquaculture - Livestock farming & ranching - Agro-industry grazing, ranching or farming5.1.1. Biological resource use - Hunting & trapping terrestrial animals - Intentional use (species is the target)

#### Conservation

Justification for conservation actions: Important conservation actions include the protection of the natural habitat of *B. albopilosum*, establishing management plans and conducting systematic monitoring to provide information for population recovery and species re-introduction programmes. Although the real occurrence of this species remains to be evaluated, some protected areas such as Refugio Nacional de La Vida Silvestre Caño Negro, Rincón de la Vieja Natural Park, Guanacaste Natural Park and Tenório Volcano Natural Park are into the probable range of the species and may be suitable for developing conservation initiatives. In order to avoid international trade, incompatible with its survival, this species is currently listed on CITES Appendix II, along with all the other species of the genus *Brachypelma* (CITES 2019). Breeding and trade are not allowed in Costa Rica, one of the countries where this species occurs and just a single Unit for Management (UMA) in Mexico is currently breeding and legally selling true *B. albopilosum* in the market. As *B. albopilosum* is a species with a high demand in the trade and easy to breed, legal breeding should be encouraged, as well as developing a system for certifying the origin of specimens that come from legal breeding programmes. It is necessary to develop better enforcement actions to curb illegal pet trade, as well as establish tax advantages for legal dealers, in order to make their prices more competitive with the ones in the black market.

##### Conservation actions

Conservation action type: In Place

Conservation actions: 1.1. Land/water protection - Site/area protection3.4.1. Species management - Ex-situ conservation - Captive breeding/artificial propagation5.1.1. Law & policy - Legislation - International level

##### Conservation actions

Conservation action type: Needed

Conservation actions: 1.2. Land/water protection - Resource & habitat protection3.1.1. Species management - Species management - Harvest management3.1.2. Species management - Species management - Trade management3.2. Species management - Species recovery3.3.1. Species management - Species re-introduction - Reintroduction3.4.1. Species management - Ex-situ conservation - Captive breeding/artificial propagation5.3. Law & policy - Private sector standards & codes5.4.1. Law & policy - Compliance and enforcement - International level5.4.2. Law & policy - Compliance and enforcement - National level6.3. Livelihood, economic & other incentives - Market forces6.4. Livelihood, economic & other incentives - Conservation payments

#### Conservation actions

Conservation action type: In Place

Conservation actions: 1.1. Land/water protection - Site/area protection3.4.1. Species management - Ex-situ conservation - Captive breeding/artificial propagation5.1.1. Law & policy - Legislation - International level

#### Conservation actions

Conservation action type: Needed

Conservation actions: 1.2. Land/water protection - Resource & habitat protection3.1.1. Species management - Species management - Harvest management3.1.2. Species management - Species management - Trade management3.2. Species management - Species recovery3.3.1. Species management - Species re-introduction - Reintroduction3.4.1. Species management - Ex-situ conservation - Captive breeding/artificial propagation5.3. Law & policy - Private sector standards & codes5.4.1. Law & policy - Compliance and enforcement - International level5.4.2. Law & policy - Compliance and enforcement - National level6.3. Livelihood, economic & other incentives - Market forces6.4. Livelihood, economic & other incentives - Conservation payments

#### Other

Justification for use and trade: Just a single Unit for Management (UMA) in Mexico is currently breeding and legally selling true *B. albopilosum* in the market. This species is currently listed on CITES Appendix II and thus its international trade is regulated by an international agreement (CITES 2019). *Brachypelma albopilosum* is a species with a recent high demand in the pet trade and its population is declining due to relatively intensive extraction for these markets. During 2006-2016, *B. albopilosum* was considered a very common species in the trade, with between 3,101–4,784 live specimens traded internationally, none of them being declared as wild-caught and most declared as being for commercial purposes (Cooper et al. 2019). Anecdotal information suggests that many of the specimens exported from Nicaragua were adult or subadult specimens, clearly indicating these would be wild-caught animals. These were seen to contain a large percentage of either pregnant females or those in breeding age, the removal of which could severely negatively impact on the ability of affected subpopulations to recover from harvesting.

##### Use and trade

Use type: International

Use and trade: 13. Pets/display animals, horticulture16. Establishing ex-situ production *

##### Ecosystem services

Ecosystem service type: Less important

##### Research needed

Research needed: 1.1. Research - Taxonomy1.2. Research - Population size, distribution & trends1.3. Research - Life history & ecology1.5. Research - Threats

Justification for research needed: Taxonomic studies are needed to clarify the status of the Honduran subpopulations. Prioritisation and support for basic research on ecology, traits, population status and distribution of the species, since there is little data about *B. albopilosum*, should also be a priority.

#### Use and trade

Use type: International

Use and trade: 13. Pets/display animals, horticulture16. Establishing ex-situ production *

#### Ecosystem services

Ecosystem service type: Less important

#### Research needed

Research needed: 1.1. Research - Taxonomy1.2. Research - Population size, distribution & trends1.3. Research - Life history & ecology1.5. Research - Threats

Justification for research needed: Taxonomic studies are needed to clarify the status of the Honduran subpopulations. Prioritisation and support for basic research on ecology, traits, population status and distribution of the species, since there is little data about *B. albopilosum*, should also be a priority.

#### Viability analysis

### Brachypelma andrewi

#### Species information

Scientific name: Brachypelma andrewi

Species authority: Schmidt, 1992

Synonyms: None.

Common names: None.

Kingdom: Animalia

Phylum: Arthropoda

Class: Arachnida

Order: Araneae

Family: Theraphosidae

Taxonomic notes: *B. andrewi* is known from a single male, supposedly from the island of Cuba (Smith 1992). However, the holotype is lost and there is no other information about the species. Mendonza and Francke (in press) are publishing a taxonomic revision of *Brachypelma* and this species will be considered a *nomen dubium*.

Region for assessment: Global

#### Geographic range

Biogeographic realm: Neotropical

Countries: Cuba

Map of records (Google Earth): Suppl. material 4

Basis of EOO and AOO: Unknown

Basis (narrative): This species EOO and AOO are unkown.

Min Elevation/Depth (m): 0

Max Elevation/Depth (m): 0

Range description: The species is known from a single male, supposedly from Cuba (Smith 1992). However, the holotype is lost and there is no other information about it.

#### New occurrences

#### Extent of occurrence

EOO (km2): Unknown

Trend: Unknown

Causes ceased?: Unknown

Causes understood?: Unknown

Causes reversible?: Unknown

Extreme fluctuations?: Unknown

#### Area of occupancy

Trend: Unknown

Causes ceased?: Unknown

Causes understood?: Unknown

Causes reversible?: Unknown

Extreme fluctuations?: Unknown

AOO (km2): Unknown

#### Locations

Number of locations: Unknown

Trend: Unknown

Extreme fluctuations?: Unknown

Justification for extreme fluctuations: The single specimen known is the holotype, thus there is no ecological data.

#### Population

Number of individuals: Unknown

Trend: Unknown

Causes ceased?: Unknown

Causes understood?: Unknown

Causes reversible?: Unknown

Extreme fluctuations?: Unknown

#### Subpopulations

Number of subpopulations: Unknown

Trend: Unknown

Extreme fluctuations?: Unknown

Severe fragmentation?: Unknown

#### Habitat

System: Terrestrial

Habitat specialist: Unknown

Habitat (narrative): Unknown habitat type.

Trend in extent, area or quality?: Unknown

##### Habitat

Habitat importance: Major Importance

Habitats: 18. Unknown

#### Habitat

Habitat importance: Major Importance

Habitats: 18. Unknown

#### Ecology

Size: unknown female, 55 mm (male).

Generation length (yr): 7

Dependency of single sp?: No

Ecology and traits (narrative): *Brachypelma andrewi* is only known from the male holotype and there is no information about its ecology.

#### Threats

Justification for threats: Since there is virtually no data about this species, threats are unknown.

##### Threats

Threat type: Past

Threats: 12. Other options - Other threat

#### Threats

Threat type: Past

Threats: 12. Other options - Other threat

#### Conservation

Justification for conservation actions: Despite the lack of basic data, *B. andrewi* is currently listed on CITES Appendix II, along with all the other species of the genus *Brachypelma* (CITES 2019).

##### Conservation actions

Conservation action type: In Place

Conservation actions: 5.1.1. Law & policy - Legislation - International level

#### Conservation actions

Conservation action type: In Place

Conservation actions: 5.1.1. Law & policy - Legislation - International level

#### Other

Justification for use and trade: *Brachypelma andrewi* is currently listed on CITES Appendix II and thus its international trade is regulated by an international agreement (CITES 2019). According to Cooper et al. (2019), a few specimens of *B. andrewi* were traded internationally during 2006–2016 from Chile to the USA, all as live specimens for commercial purposes. Since the identity of *B. andrewi* is unclear, all these specimens traded as *B. andrewi* presumably were misidentified.

##### Use and trade

Use type: International

Use and trade: 13. Pets/display animals, horticulture

##### Ecosystem services

Ecosystem service type: Less important

##### Research needed

Research needed: 1.1. Research - Taxonomy1.2. Research - Population size, distribution & trends1.3. Research - Life history & ecology

Justification for research needed: Virtually nothing is known about *B. andrewi*, including its taxonomic validity. Thus, basic research on taxonomy, ecology and distribution has to be supported as the first step to any other studies and actions.

#### Use and trade

Use type: International

Use and trade: 13. Pets/display animals, horticulture

#### Ecosystem services

Ecosystem service type: Less important

#### Research needed

Research needed: 1.1. Research - Taxonomy1.2. Research - Population size, distribution & trends1.3. Research - Life history & ecology

Justification for research needed: Virtually nothing is known about *B. andrewi*, including its taxonomic validity. Thus, basic research on taxonomy, ecology and distribution has to be supported as the first step to any other studies and actions.

#### Viability analysis

### Brachypelma auratum

#### Species information

Scientific name: Brachypelma auratum

Species authority: Schmidt, 1992

Common names: Mexican flameknee, tarántula rodillas de fuego, tarántula mexicana rodilla de llama, tarántula rodillas rojas, mygale à genoux de feu du Mexique.

Kingdom: Animalia

Phylum: Arthropoda

Class: Arachnida

Order: Araneae

Family: Theraphosidae

**Figure 10.**
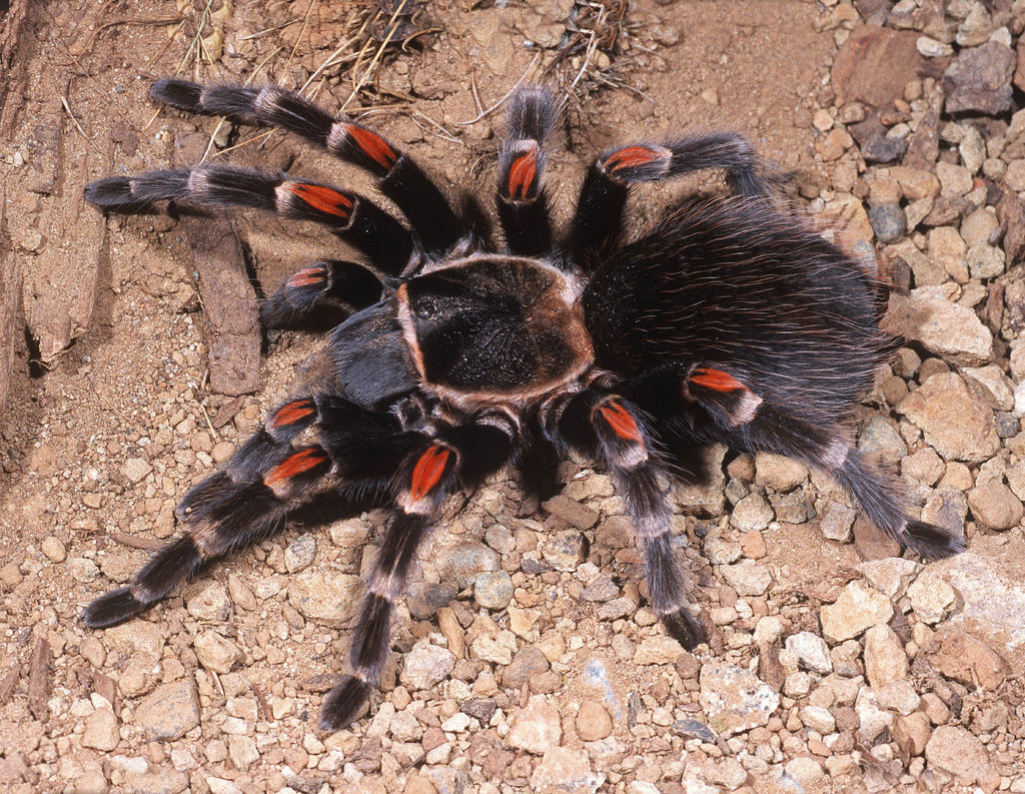
*Brachypelma auratum* Schmidt, 1992 female near Ciudad Altamirano, Guerrero state, Mexico. Photo: Rick C. West.

**Figure 11.**
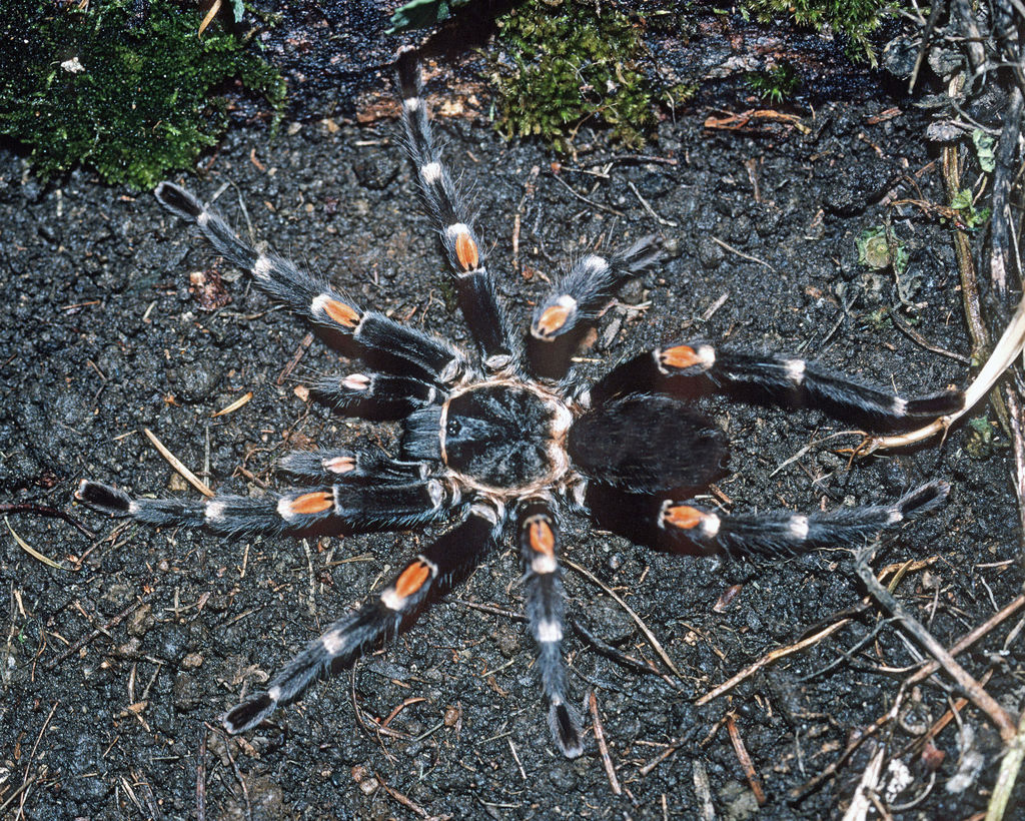
*Brachypelma auratum* Schmidt, 1992 male near Ciudad Altamirano, Guerrero state, Mexico. Photo: Rick C. West.

Figure(s) or Photo(s): Figs 10, 11

Region for assessment: Global

#### Geographic range

Biogeographic realm: Neotropical

Countries: Mexico

**Figure 12.**
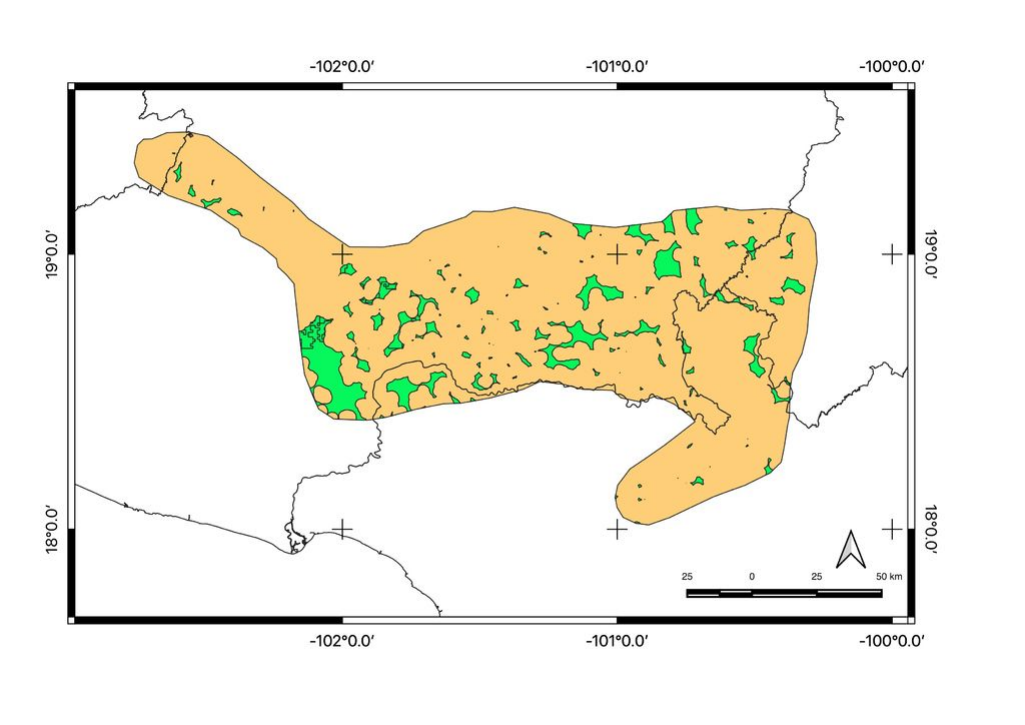
Habitat quality across the distribution of *Brachypelma auratum* Schmidt, 1992. Low quality habitat areas (LQH) in orange and high quality habitat areas (HQH) in green (for estimation details, see formula in Methods).

Map of records (image): Fig. 12

Map of records (Google Earth): Suppl. material 5

Basis of EOO and AOO: Species Distribution Model

Basis (narrative): A species distribution modelling has been performed to predict its potential range. See methods for details.

Min Elevation/Depth (m): 130

Max Elevation/Depth (m): 2030

Range description: *Brachypelma auratum* is endemic to Mexico and occurs mainly north of the Sierra Madre del Sur and south of the Transverse Neovolcanic Ranges, mainly in the Balsas River Basin, from eastern Jalisco State, through northern and central Michoacán State and north-western Guerrero State, as well as in the south-western Mexico State (Schmidt 1992, Locht et al. 1999, Gabriel and Longhorn 2015, Mendoza and Francke 2017, CEC 2017, iNaturalist 2018, unpublished data). This species does not occur in coastal lowlands since the mountain range acts as a natural barrier. *Brachypelma auratum* is sympatric with *B. albiceps* in Guerrero State, only at the easternmost known extent of *B. auratum*.

#### New occurrences

#### Extent of occurrence

EOO (km2): 24735

Trend: Decline (inferred)

Justification for trend: There is a possible loss of EOO due to agriculture activities with consequent deforestation.

Causes ceased?: No

Causes understood?: Yes

Causes reversible?: Yes

Extreme fluctuations?: No

#### Area of occupancy

Trend: Decline (inferred)

Justification for trend: There is a possible loss of AOO due to agriculture activities with consequent deforestation.

Causes ceased?: No

Causes understood?: Yes

Causes reversible?: Yes

Extreme fluctuations?: No

AOO (km2): 11616

#### Locations

Number of locations: Unknown

Justification for number of locations: Unknown number of locations that is nevertheless higher than any thresholds.

Trend: Unknown

Extreme fluctuations?: No

#### Population

Number of individuals: Unknown

Trend: Decline (inferred)

Justification for trend: Despite a lack of formal data about population reduction, the number of individuals is likely to be declining due to a loss in AOO and EOO owing to increasingly intense agricultural activities, combined with intense harvesting for the illegal pet trade.

Basis for decline: (c) a decline in area of occupancy, extent of occurrence and/or quality of habitat(d) actual or potential levels of exploitation

Causes ceased?: No

Causes understood?: Yes

Causes reversible?: Yes

Extreme fluctuations?: No

Population Information (Narrative): Despite a lack of formal data about population reduction, the number of individuals is likely to be declining due to a loss in AOO and EOO owing to increasingly intense agricultural activities, combined with intense harvesting for the illegal pet trade.

#### Subpopulations

Number of subpopulations: Unknown

Trend: Decline (inferred)

Justification for trend: The number of subpopulations is expected to be declining given the strong human pressure and severe fragmentation.

Extreme fluctuations?: No

Severe fragmentation?: Yes

Justification for fragmentation: Based on the analysis of the relative abundance of each species in low quality habitat (RA_LQH_) and the Species Distribution Model, 90.5% of the population should be in subpopulations that are non-viable and without possibility of rescue effects due to fragmentation.

#### Habitat

System: Terrestrial

Habitat specialist: Yes

Habitat (narrative): *Brachypelma auratum* primarily inhabits the Balsas dry forest on the inland side of the Sierra Madre del Sur. Compared to the outer Pacific coast, the inland thorn and dry deciduous forest habitat of the Balsas is seasonally much hotter and drier for several months of the year (Hijmensen 2018). The species can be found in an altitudinal range up to 2000 m a.s.l., but prefers low and medium altitudes and warmer climate.

Trend in extent, area or quality?: Decline (inferred)

Justification for trend: The habitat of this species is inferred to be declining in area, extent and quality due to deforestation (Global Forest Watch 2019), in particular through expanding agricultural activities.

**Figure 13.**
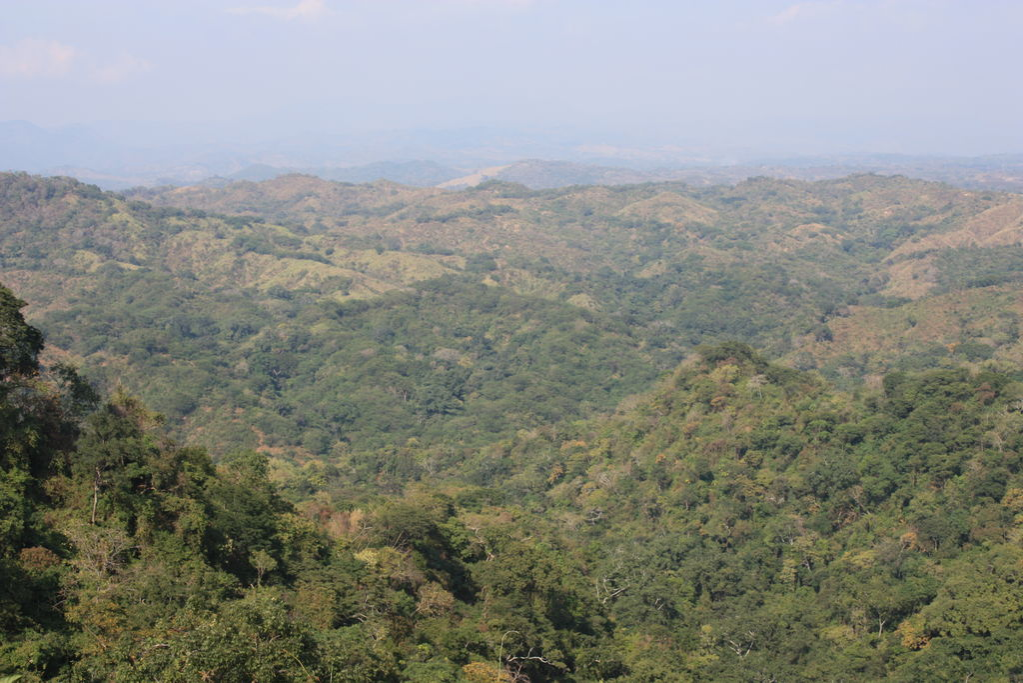
*Brachypelma auratum* habitat, dry forest, near Ciudad Altamirano, Guerrero state, Mexico. Photo: Stuart J. Longhorn.

Figure(s) or Photo(s): Fig. 13

##### Habitat

Habitat importance: Major Importance

Habitats: 1.5. Forest - Subtropical/Tropical Dry

#### Habitat

Habitat importance: Major Importance

Habitats: 1.5. Forest - Subtropical/Tropical Dry

#### Ecology

Size: 70 mm (females); 60 mm (males).

Generation length (yr): 9

Dependency of single sp?: No

Ecology and traits (narrative): *Brachypelma auratum* is a fossorial species that modifies previously excavated burrows or can excavate their own unaided, often only minor alterations being made to natural small cavities under debris, such as large rocks and tree roots in thorny brush and subtropical dry forest. Juveniles are often instead found in scrapes under stones (see also Hijmensen 2018). Burrows mostly do not have any silk around the entrance, giving no clear indication there is a spider inside and can be rather horizontal and broad. These can have the same appearance as those dug by ground squirrels, which have also been seen at some locations alongside *B. auratum*. It may be speculated that the co-existence of these mammals may be beneficial to *B. auratum* by providing them with potential new burrows. These spiders are nocturnal predators that wait near the entrance of their refuge from dusk and into the night to feed primarily on ground-dwelling arthropods (insects, other arachnids and some myriapods) or even small vertebrates. The mating season occurs during the last part of the rainy and first part of dry seasons (August to January) when mature males wander in the open to search for females. The males are likely most active at night, cooler daylight hours and throughout overcast days. Adult females typically moult once per year, just prior to the onset of the annual male emergence. Females produce cocoons (large silken egg sacs) during the drier winter months with young emerging about two months later, with most young dispersing in the late spring or summer, just before the onset of the early summer rains.

#### Threats

Justification for threats: There has been extensive human development of much of the former habitat for this species, including substantial deforestation and degradation of the remaining habitat. This has been mainly through poorly practised non-industrial agricultural activities of small-holders (various cash-crop vegetables, sugar-cane and some fruits) but also by large-scale agricultural production of key crops (particularly corn, avocadoes and sorghum). There is also substantial livestock farming, partly through extensive ranching but also several areas with more intensive and destructive practices (particularly dairy and domestic fowl). This species, along with the others of the red leg complex, are more fragile and does not adapt well or quickly to alterations in the environment caused by human activities (Turner et al. 2018). The major threats are habitat loss due to agriculture and intense trafficking compared to most other species. There is some legal trade of captive-bred live specimens, but also an unknown amount of trafficking of live animals, although likely to frequently be of significant harm to wild populations. Roads facilitate the access of smugglers to wild populations and also are a source *per se* of habitat degradation. The high demand by the pet market can be considered as a significant threat since the legally regulated captivity production is comparably low. Illegal specimens have been regularly extracted and sold in several local Mexican markets as *B. smithi*, undercutting potential legal and sustainable trade of both species.

##### Threats

Threat type: Ongoing

Threats: 2.1.2. Agriculture & aquaculture - Annual & perennial non-timber crops - Small-holder farming2.1.3. Agriculture & aquaculture - Annual & perennial non-timber crops - Agro-industry farming2.3.2. Agriculture & aquaculture - Livestock farming & ranching - Small-holder grazing, ranching or farming2.3.3. Agriculture & aquaculture - Livestock farming & ranching - Agro-industry grazing, ranching or farming4.1. Transportation & service corridors - Roads & railroads5.1.1. Biological resource use - Hunting & trapping terrestrial animals - Intentional use (species is the target)

#### Threats

Threat type: Ongoing

Threats: 2.1.2. Agriculture & aquaculture - Annual & perennial non-timber crops - Small-holder farming2.1.3. Agriculture & aquaculture - Annual & perennial non-timber crops - Agro-industry farming2.3.2. Agriculture & aquaculture - Livestock farming & ranching - Small-holder grazing, ranching or farming2.3.3. Agriculture & aquaculture - Livestock farming & ranching - Agro-industry grazing, ranching or farming4.1. Transportation & service corridors - Roads & railroads5.1.1. Biological resource use - Hunting & trapping terrestrial animals - Intentional use (species is the target)

#### Conservation

Justification for conservation actions: Important conservation actions include protecting the natural habitat of *B. auratum*, establishing management plans and conducting systematic monitoring to provide information for population recovery and species reintroduction programmes. Although the real occurrence of this species remains to be evaluated, a protected area called Zicuirán Infiernillo Biosphere Reserve is inside the range of the species and may be suitable for developing conservation initiatives. In order to avoid international trade incompatible with its survival, this species is listed on CITES Appendix II along with all the other species of the genus *Brachypelma* (CITES 2019). *Brachypelma auratum* is being reared in captivity in Mexican Units for Management (UMAs) and sold legally. However, there is still a dominant illegal pet trade of this species. Thus, the government of Mexico should collaborate with Mexican tarantula breeders to develop a system for certifying the origin of specimens used in breeding programmes of the Units for Management and increase surveillance and control of specimens collected and traded. Tax advantages for legal dealers would make their prices more competitive with the ones in the black market. Currently, this species is being reared in captivity in a Mexican Unit for Management that also promotes educational and awareness activities regarding tarantula spiders aimed at students and the general public.

##### Conservation actions

Conservation action type: In Place

Conservation actions: 1.1. Land/water protection - Site/area protection3.4.1. Species management - Ex-situ conservation - Captive breeding/artificial propagation5.1.1. Law & policy - Legislation - International level4.3. Education & awareness - Awareness & communications

##### Conservation actions

Conservation action type: Needed

Conservation actions: 1.1. Land/water protection - Site/area protection1.2. Land/water protection - Resource & habitat protection3.1.1. Species management - Species management - Harvest management3.1.2. Species management - Species management - Trade management3.2. Species management - Species recovery3.3.1. Species management - Species re-introduction - Reintroduction5.3. Law & policy - Private sector standards & codes5.4.1. Law & policy - Compliance and enforcement - International level5.4.2. Law & policy - Compliance and enforcement - National level5.4.3. Law & policy - Compliance and enforcement - Sub-national level6.4. Livelihood, economic & other incentives - Conservation payments

#### Conservation actions

Conservation action type: In Place

Conservation actions: 1.1. Land/water protection - Site/area protection3.4.1. Species management - Ex-situ conservation - Captive breeding/artificial propagation5.1.1. Law & policy - Legislation - International level4.3. Education & awareness - Awareness & communications

#### Conservation actions

Conservation action type: Needed

Conservation actions: 1.1. Land/water protection - Site/area protection1.2. Land/water protection - Resource & habitat protection3.1.1. Species management - Species management - Harvest management3.1.2. Species management - Species management - Trade management3.2. Species management - Species recovery3.3.1. Species management - Species re-introduction - Reintroduction5.3. Law & policy - Private sector standards & codes5.4.1. Law & policy - Compliance and enforcement - International level5.4.2. Law & policy - Compliance and enforcement - National level5.4.3. Law & policy - Compliance and enforcement - Sub-national level6.4. Livelihood, economic & other incentives - Conservation payments

#### Other

Justification for use and trade: *Brachypelma auratum* is being reared in captivity in Mexican Unit for Management (UMA) and sold legally. This species is currently listed on CITES Appendix II and thus its international trade is regulated by an international agreement (CITES 2019). However, there is relatively intense trafficking of this species. The high demand on the pet market can be considered as an important threat since the legal captive production is lower than demand. Captive-bred juveniles are sold for approximately US$25–$125 in Canada and the United States, for US$10 in Mexico and for US$4 in the EU; adult females are sold at approximately US$400 in Canada and US$54 in the EU (CEC 2017). Traders offer money for local people to collect *B. auratum* specimens and thus it is the most illegally traded species in Mexico, being regularly sold in markets of Mexico City (CEC 2017). It is also one of the most requested species internationally, for example in European and USA pet markets. As the species is also reared outside Mexico (mainly in Germany), the Mexican number of produced and exported specimens do not reflect the real number of specimens in the trade (CEC 2017). Between 1,008–1,059 live specimens of *B. auratum* were traded internationally during 2006–2016, none declared as wild-caught and mainly traded for commercial purposes (Cooper et al. 2019). However, more importantly, regarding illegal pet trade, the largest part of trafficking specimens can be comprised of mature and even pregnant adult females, which may produce a far higher number of young in captivity which are then sold for additional profit, rather than supporting recovery of natural populations.

##### Use and trade

Use type: International

Use and trade: 13. Pets/display animals, horticulture16. Establishing ex-situ production *

##### Ecosystem services

Ecosystem service type: Less important

##### Research needed

Research needed: 2.3. Conservation Planning - Harvest & Trade Management Plan3.1. Monitoring - Population trends3.2. Monitoring - Harvest level trends3.3. Monitoring - Trade trends

Justification for research needed: The government of Mexico should collaborate with Mexican tarantula breeders to develop a system for certifying the origin of specimens used in the breeding programmes of the Units for Management (UMA). Additionally, systematic monitoring and protection could be undertaken in known subpopulations. Further studies are needed to confirm population, harvest and trade trends and how the latter affect harvest levels.

#### Use and trade

Use type: International

Use and trade: 13. Pets/display animals, horticulture16. Establishing ex-situ production *

#### Ecosystem services

Ecosystem service type: Less important

#### Research needed

Research needed: 2.3. Conservation Planning - Harvest & Trade Management Plan3.1. Monitoring - Population trends3.2. Monitoring - Harvest level trends3.3. Monitoring - Trade trends

Justification for research needed: The government of Mexico should collaborate with Mexican tarantula breeders to develop a system for certifying the origin of specimens used in the breeding programmes of the Units for Management (UMA). Additionally, systematic monitoring and protection could be undertaken in known subpopulations. Further studies are needed to confirm population, harvest and trade trends and how the latter affect harvest levels.

#### Viability analysis

### Brachypelma aureoceps

#### Species information

Scientific name: Brachypelma aureoceps

Species authority: (Chamberlin, 1917)

Synonyms: *Eurypelma aureoceps* Chamberlin, 1917; *Avicularia aureoceps* (Chamberlin, 1917).

Common names: Florida golden chestnut, tarántula dorada de Florida, mygale châtaigne dorée de Floride.

Kingdom: Animalia

Phylum: Arthropoda

Class: Arachnida

Order: Araneae

Family: Theraphosidae

Taxonomic notes: This species is known from a single female, described in 1917 and reportedly from the Dry Tortugas islands, Florida, USA (Chamberlin 1917). Mendonza and Francke (in press) are publishing a taxonomic revision of *Brachypelma* and this species will be considered a *nomen dubium*.

Region for assessment: Global

#### Geographic range

Biogeographic realm: Neotropical

Countries: United States

Map of records (Google Earth): Suppl. material 6

Basis of EOO and AOO: Unknown

Basis (narrative): This species EOO and AOO are unknown.

Range description: This species is known from a single female, described in 1917 and reportedly from the Dry Tortugas islands, Florida, USA (Chamberlin 1917). To date, no further specimens have been found from this area. Furthermore, Dry Tortugas are outside the range of all known *Brachypelma* species. The type locality, as the species itself, is considered dubious.

#### New occurrences

#### Extent of occurrence

EOO (km2): Unknown

Trend: Unknown

Justification for trend: 

Causes ceased?: Unknown

Causes understood?: Unknown

Causes reversible?: Unknown

Extreme fluctuations?: Unknown

#### Area of occupancy

Trend: Unknown

Causes ceased?: Unknown

Causes understood?: Unknown

Causes reversible?: Unknown

Extreme fluctuations?: Unknown

AOO (km2): Unknown

#### Locations

Number of locations: Unknown

Trend: Unknown

Extreme fluctuations?: Unknown

#### Population

Number of individuals: Unknown

Trend: Unknown

Causes ceased?: Unknown

Causes understood?: Unknown

Causes reversible?: Unknown

Extreme fluctuations?: Unknown

Population Information (Narrative): No population information is available.

#### Subpopulations

Number of subpopulations: Unknown

Trend: Unknown

Extreme fluctuations?: Unknown

Severe fragmentation?: Unknown

#### Habitat

System: Terrestrial

Habitat specialist: Unknown

Habitat (narrative): *Brachypelma aureoceps* is from an unknown habitat. The single specimen known is the holotype, thus there are no population and ecological data. Adding to this, the type locality is Dry Tortugas (Chamberlin 1917), that is outside the range of all known *Brachypelma* species, making any assumptions about habitat type dubious.

Trend in extent, area or quality?: Unknown

##### Habitat

Habitat importance: Major Importance

Habitats: 18. Unknown

#### Habitat

Habitat importance: Major Importance

Habitats: 18. Unknown

#### Ecology

Size: 44 mm (female holotype), unknown male.

Generation length (yr): 7

Dependency of single sp?: Unknown

Ecology and traits (narrative): *Brachypelma aureoceps* are only known from the female holotype and there is no information about its ecology.

#### Threats

Justification for threats: There is no research in basic ecological features, so it is not possible to identify threats to this species.

##### Threats

Threat type: Past

Threats: 12. Other options - Other threat

#### Threats

Threat type: Past

Threats: 12. Other options - Other threat

#### Conservation

Justification for conservation actions: No conservation actions can be suggested before basic taxonomic and ecological research is done. Despite the current available data being scarce, this species is listed on CITES Appendix II along with all the other *Brachypelma* species (CITES 2019).

##### Conservation actions

Conservation action type: In Place

Conservation actions: 5.1.1. Law & policy - Legislation - International level

#### Conservation actions

Conservation action type: In Place

Conservation actions: 5.1.1. Law & policy - Legislation - International level

#### Other

Justification for use and trade: This species is currently listed on CITES Appendix II and thus is protected by an international agreement (CITES 2019) and just a single specimen of *B. aureoceps* was reportedly internationally traded during 2006–2016.

##### Use and trade

Use type: International

Use and trade: 13. Pets/display animals, horticulture

##### Ecosystem services

Ecosystem service type: Less important

##### Research needed

Research needed: 1.1. Research - Taxonomy1.2. Research - Population size, distribution & trends1.3. Research - Life history & ecology

Justification for research needed: Virtually nothing is known about this species, including its validity. Thus, basic research on taxonomy, ecology and distribution has to be supported as the first step to any other studies and actions.

#### Use and trade

Use type: International

Use and trade: 13. Pets/display animals, horticulture

#### Ecosystem services

Ecosystem service type: Less important

#### Research needed

Research needed: 1.1. Research - Taxonomy1.2. Research - Population size, distribution & trends1.3. Research - Life history & ecology

Justification for research needed: Virtually nothing is known about this species, including its validity. Thus, basic research on taxonomy, ecology and distribution has to be supported as the first step to any other studies and actions.

#### Viability analysis

### Brachypelma baumgarteni

#### Species information

Scientific name: Brachypelma baumgarteni

Species authority: Smith, 1993

Common names: Mexican orangebeauty, tarántula anaranjada, tarántula mexicana naranja, mygale orange du Mexique.

Kingdom: Animalia

Phylum: Arthropoda

Class: Arachnida

Order: Araneae

Family: Theraphosidae

**Figure 14.**
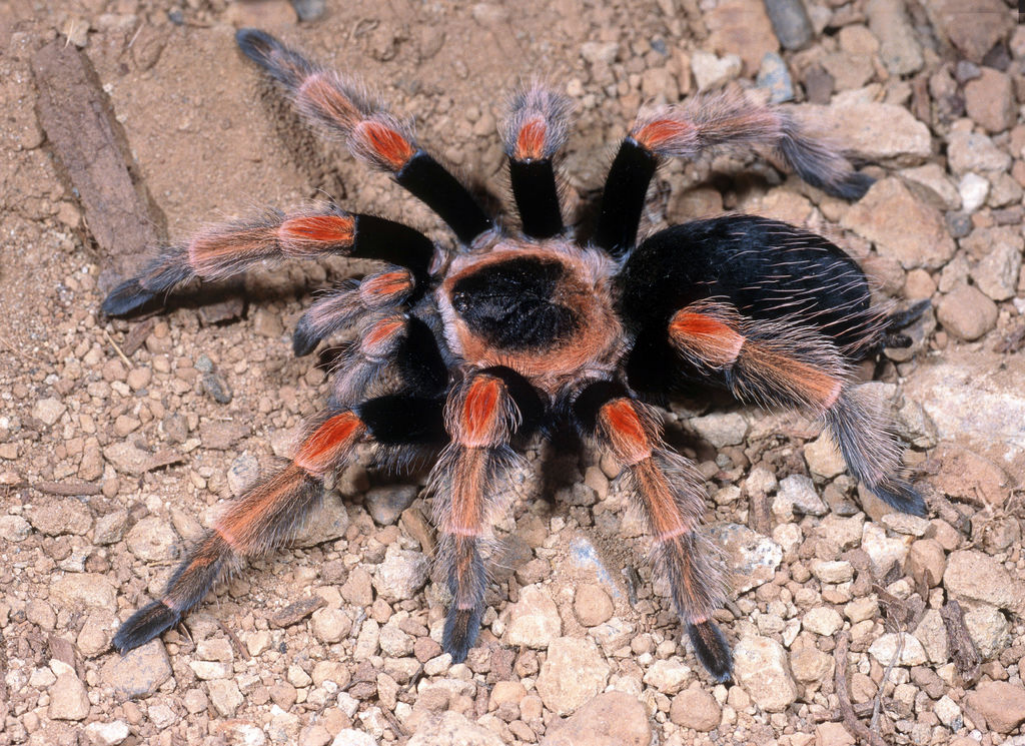
*Brachypelma baumgarteni* Smith, 1993 female from Michoacán state, Mexico. Photo: Rick C. West.

**Figure 15.**
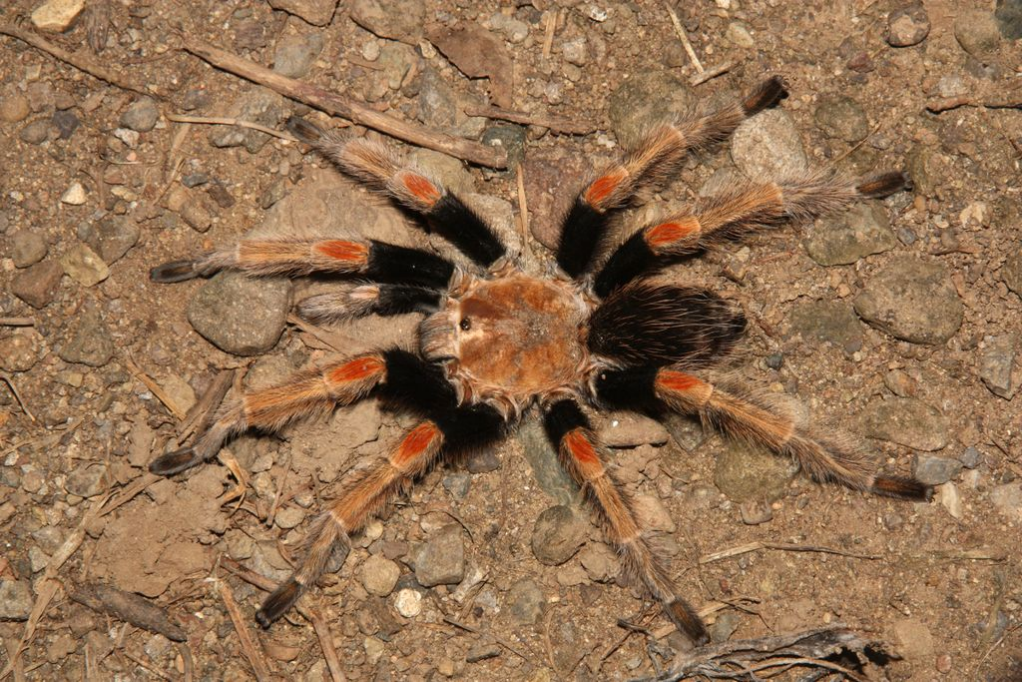
*Brachypelma baumgarteni* Smith, 1993 male near Lázaro Cárdenas, Michoacán state, Mexico. Photo: Stuart J. Longhorn.

Figure(s) or Photo(s): Figs 14, 15

Region for assessment: Global

#### Geographic range

Biogeographic realm: Neotropical

Countries: Mexico

**Figure 16.**
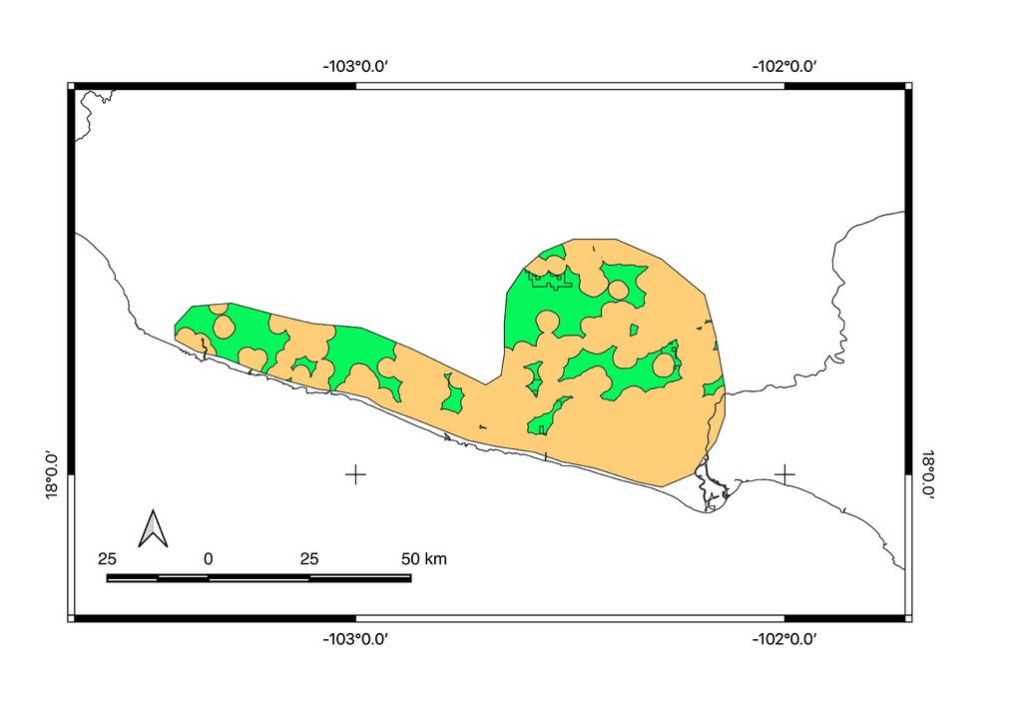
Habitat quality across the distribution of *Brachypelma baumgarteni* Smith, 1993. Low quality habitat areas (LQH) in orange and high quality habitat areas (HQH) in green (for estimation details, see formula in Methods).

Map of records (image): Fig. 16

Map of records (Google Earth): Suppl. material 7

Basis of EOO and AOO: Species Distribution Model

Basis (narrative): A species distribution modelling has been performed to predict its potential range. See methods for details.

Min Elevation/Depth (m): 110

Max Elevation/Depth (m): 1360

Range description: *Brachypelma baumgarteni* is endemic to Mexico and found in the coastal region of the Sierra Madre del Sur, west of the Balsas River Basin, in south-eastern Michoacán State (Smith 1993, Smith 1995, CEC 2017, Mendoza and Francke 2017, iNaturalist 2018 unpublished data). It is sympatric with *B. hamorii* in the north-western part of Michoacán State.

#### New occurrences

#### Extent of occurrence

EOO (km2): 2727

Trend: Decline (inferred)

Justification for trend: Human pressure, such as urbanisation and agriculture, are affecting the extent of occurrence, as the species seems to be dependent on well-preserved forests.

Causes ceased?: No

Causes understood?: Yes

Causes reversible?: No

Extreme fluctuations?: No

#### Area of occupancy

Trend: Decline (inferred)

Justification for trend: Human pressure, such as urbanisation and agriculture are affecting the area of occupancy, as the species seems to be dependent on well-preserved forests.

Causes ceased?: No

Causes understood?: Yes

Causes reversible?: No

Extreme fluctuations?: No

AOO (km2): 2248

#### Locations

Number of locations: 100

Justification for number of locations: Derived from the approximate number of human populations above 100 inhabitants needed to cover the entire range of the species. We assume each locality covers a mean radius of 2.5 km.

Trend: Decline (inferred)

Justification for trend: We assume the number of locations to be decreasing, given the generalised loss of subpopulations of this species.

Extreme fluctuations?: No

#### Population

Number of individuals: Unknown

Trend: Decline (inferred)

Justification for trend: Despite the population size being unknown, it is possible to infer its decline since both the AOO and the quality of habitat are decreasing (Global Forest Watch 2019). There is also increasing harvesting pressure on the few subpopulations remaining. Human pressure, such as changing land use through urbanisation and agriculture, are the main causes of the inferred decline since the species seems to be forest-dependent and does not appear to adapt well to disturbed habitats. In addition, some subpopulations are known to have been devastated by hurricanes. Since it is a desirable species in the pet trade and legal breeding in captivity is too low to meet the demands, harvesting pressure on wild populations is increasing. Finally, like other *Brachypelma* species which occur in ranges around the north-south coastal Pacific highway, many males of *B. baumgarteni* are run over while crossing roads during the mating season.

Basis for decline: (c) a decline in area of occupancy, extent of occurrence and/or quality of habitat(d) actual or potential levels of exploitation

Causes ceased?: No

Causes understood?: Yes

Causes reversible?: No

Extreme fluctuations?: No

Population Information (Narrative): Despite the population size being unknown, it is possible to infer its decline since AOO, EOO and the quality of habitat are decreasing (Global Forest Watch 2019). There is also increasing harvesting pressure on the few subpopulations remaining. Human pressure, such as changing land use through urbanisation and agriculture, are the main causes of the inferred decline since the species seems to be forest-dependent and does not appear to adapt well to disturbed habitats. In addition, some subpopulations are known to have been devastated by hurricanes. Since it is a desirable species in the pet trade and legal breeding in captivity is too low to meet the demands, harvesting pressure on wild populations is increasing and is facilitated by construction of roads. Finally, like other *Brachypelma* species which occur in ranges around the north-south coastal Pacific highway, many males of *B. baumgarteni* are run over while crossing roads during the mating season.

#### Subpopulations

Number of subpopulations: Unknown

Trend: Decline (inferred)

Justification for trend: Subpopulations are expected to be declining given the strong human pressure and severe fragmentation.

Extreme fluctuations?: No

Severe fragmentation?: Yes

Justification for fragmentation: Based on the analysis of the relative abundance of each species in low quality habitat (RA_LQH_) and the Species Distribution Model, 84.5% of the population should be in subpopulations that are non-viable and without the possibility of rescue effects due to fragmentation.

#### Habitat

System: Terrestrial

Habitat specialist: Yes

Habitat (narrative): *Brachypelma baumgarteni* inhabits the subtropical dry forest in the foothills of the Sierra Madre del Sur mountains, primarily in the lowlands near the Pacific coast. This species appears to prefer shady forested areas and healthy populations are not found outside this type of habitat. However, it has been previously reported that burrows can be found on grass hillsides and are approximately 30-40 cm deep (Smith 1995), which can be the case, although more recent data indicates this appears to be a rather unfavourable habitat.

Trend in extent, area or quality?: Decline (inferred)

Justification for trend: The habitat of this species is inferred to be declining in area, extent and quality due to expansion of urbanisation and agriculture, both of which appear as important factors massively reducing the remaining habitat of this species, which does not adapt well to disturbance (appearing to be a forest-specialist). Large areas of forest have already been cleared in this zone, to which the species does not adapt. The construction and expansion of roads is also destroying habitat and for the remaining subpopulations adjacent to them, the major pacific transit routes in this region provide both a barrier to dispersal and cause of mortality as males, in particular, appear to be often run over by traffic. Natural phenomena, such as hurricanes, have also likely caused a decrease in the quality of its coastal habitat.

**Figure 17.**
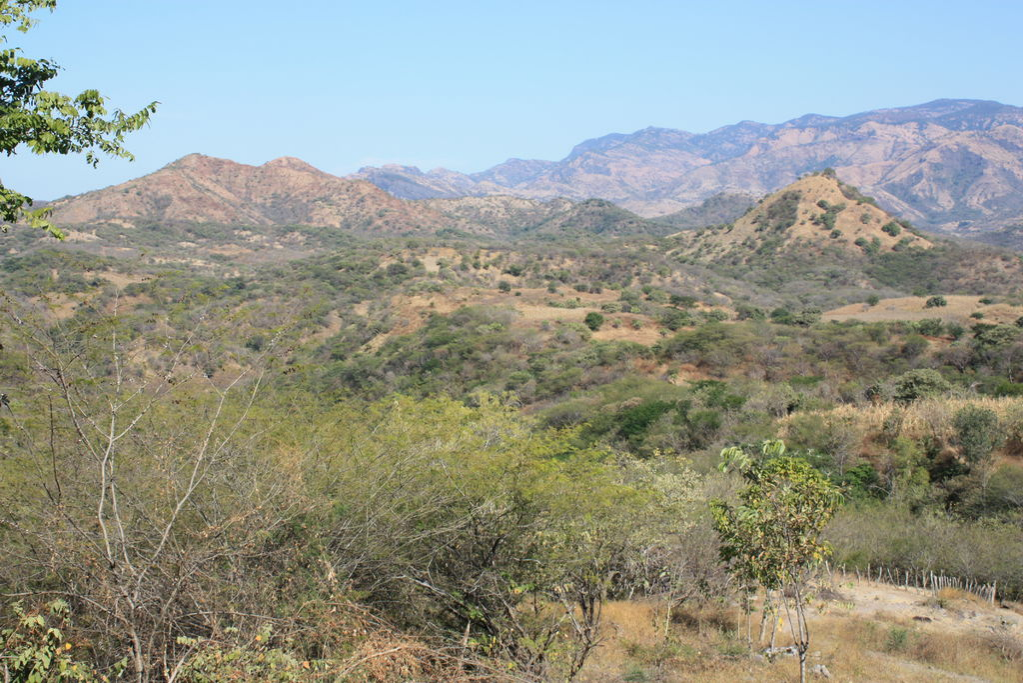
*Brachypelma baumgarteni* habitat, subtropical dry forest, near Lázaro Cárdenas, Michoacán state, Mexico. Photo: Stuart J. Longhorn.

Figure(s) or Photo(s): Fig. 17

##### Habitat

Habitat importance: Major Importance

Habitats: 1.5. Forest - Subtropical/Tropical Dry

#### Habitat

Habitat importance: Major Importance

Habitats: 1.5. Forest - Subtropical/Tropical Dry

#### Ecology

Size: 75 mm (female); 65 mm (male).

Generation length (yr): 7

Dependency of single sp?: No

Ecology and traits (narrative): *Brachypelma baumgarteni* is a fossorial species that modifies previously excavated burrows or can excavate their own unaided, often with only minor alterations to natural small cavities under debris, such as large rocks and tree roots in dense vegetation of dry forests. These can be found on both sloped and level ground. Burrows mostly do not have any silk around the entrance, giving no clear indication there is a spider inside. These spiders are nocturnal predators that wait near the entrance of their refuge from dusk and into the night to feed primarily on ground-dwelling arthropods (insects, other arachnids and some myriapods) or even small vertebrates. The mating season occurs during the last part of the rainy and first part of dry seasons (August to January) when mature males wander in the open to search for females. The males are likely most active at night, cooler daylight hours and throughout overcast days. Adult females typically moult once per year, just prior to the onset of the annual male emergence. Females will produce cocoons (large silken egg sacs) during the drier winter months with young emerging about two months later. Most young disperse in the late spring or summer, just before the onset of the early summer rains.

#### Threats

Justification for threats: Until five years ago, *B. baumgarteni* was commonly found in the wild. However, about 3 years ago, specimens became difficult to find in the places they used to live. Since *B. baumgarteni* seems to be forest-dependent, human pressure, such as urbanisation and agriculture, could be the main cause of the species disappearance, but local information indicates that some subpopulations were devastated by hurricanes. Along with the others of the red leg complex, this species is more fragile and does not adapt well or quickly to alterations in the environment caused by human activities (Turner et al. 2018). Adding to this, red leg species are more popular as pets than the red abdomen ones. As breeding in captivity is too low to meet the market demands and because there has been a recent rise in this number, the harvesting pressure on wild populations is increasing. Roads facilitate the access of smugglers to wild populations and also are a source *per se* of habitat degradation. Adding to this, likewise the other *Brachypelma* species that occur along the north-south coastal Pacific highway, males of *B. baumgarteni* are killed by cars while crossing roads during the mating season.

##### Threats

Threat type: Ongoing

Threats: 1.1. Residential & commercial development - Housing & urban areas2.1.2. Agriculture & aquaculture - Annual & perennial non-timber crops - Small-holder farming2.1.3. Agriculture & aquaculture - Annual & perennial non-timber crops - Agro-industry farming4.1. Transportation & service corridors - Roads & railroads5.1.1. Biological resource use - Hunting & trapping terrestrial animals - Intentional use (species is the target)11.4. Climate change & severe weather - Storms & flooding

#### Threats

Threat type: Ongoing

Threats: 1.1. Residential & commercial development - Housing & urban areas2.1.2. Agriculture & aquaculture - Annual & perennial non-timber crops - Small-holder farming2.1.3. Agriculture & aquaculture - Annual & perennial non-timber crops - Agro-industry farming4.1. Transportation & service corridors - Roads & railroads5.1.1. Biological resource use - Hunting & trapping terrestrial animals - Intentional use (species is the target)11.4. Climate change & severe weather - Storms & flooding

#### Conservation

Justification for conservation actions: Important conservation actions include protecting the natural habitat of *Brachypelma baumgarteni*, establishing management plans and conducting systematic monitoring to provide information for population recovery and species reintroduction programmes. As there is no official protected area in its range of occurrence, it is highly recommended to establish one in north Michoacán State in order to help in conservation, recovery and protection, not only of *B. baumgarteni*, but of *B. hamorii* as well, since they are sympatric in that area. In order to avoid international trade incompatible with its survival, this species is currently listed on CITES Appendix II along with all the other species of the genus *Brachypelma* (CITES 2019). *Brachypelma baumgarteni* is being reared in captivity in a single Mexican Unit for Management (UMA) and sold legally. However, there is still a dominant illegal pet trade of this species. Thus, the government of Mexico should collaborate with Mexican tarantula breeders to develop a system for certifying the origin of specimens used in the breeding programmes of Unit for Management and increase surveillance and control of specimens collected and traded. Tax advantages for legal dealers would make their prices more competitive with the ones in the black market. Captive breeding and specimens re-introductions into the wild, such as those being planned by Aracneé Unit for Management, should also be encouraged by authorities.

##### Conservation actions

Conservation action type: In Place

Conservation actions: 3.4. Species management - Ex-situ conservation5.1.1. Law & policy - Legislation - International level

##### Conservation actions

Conservation action type: Needed

Conservation actions: 1.1. Land/water protection - Site/area protection1.2. Land/water protection - Resource & habitat protection3.1.1. Species management - Species management - Harvest management3.1.2. Species management - Species management - Trade management3.2. Species management - Species recovery3.3.1. Species management - Species re-introduction - Reintroduction5.3. Law & policy - Private sector standards & codes5.4.1. Law & policy - Compliance and enforcement - International level5.4.2. Law & policy - Compliance and enforcement - National level5.4.3. Law & policy - Compliance and enforcement - Sub-national level6.4. Livelihood, economic & other incentives - Conservation payments

#### Conservation actions

Conservation action type: In Place

Conservation actions: 3.4. Species management - Ex-situ conservation5.1.1. Law & policy - Legislation - International level

#### Conservation actions

Conservation action type: Needed

Conservation actions: 1.1. Land/water protection - Site/area protection1.2. Land/water protection - Resource & habitat protection3.1.1. Species management - Species management - Harvest management3.1.2. Species management - Species management - Trade management3.2. Species management - Species recovery3.3.1. Species management - Species re-introduction - Reintroduction5.3. Law & policy - Private sector standards & codes5.4.1. Law & policy - Compliance and enforcement - International level5.4.2. Law & policy - Compliance and enforcement - National level5.4.3. Law & policy - Compliance and enforcement - Sub-national level6.4. Livelihood, economic & other incentives - Conservation payments

#### Other

Justification for use and trade: *Brachypelma baumgarteni* is being reared in captivity in a single Unit for Management (UMA) and sold legally. This species is currently listed on CITES Appendix II and thus its international trade is regulated by an international agreement (CITES 2019). This species is being reared in captivity in a single Unit for Management (UMA) and sold legally. Between 2006-2016, about 960–1,108 *B. baumgarteni* specimens were traded internationally and it is considered a common species in the market (Cooper et al. 2019). All *B. baumgarteni* in trade were live specimens and none was declared as wild-caught, mostly being traded for commercial purposes (Cooper et al. 2019).

##### Use and trade

Use type: International

Use and trade: 13. Pets/display animals, horticulture16. Establishing ex-situ production *

##### Ecosystem services

Ecosystem service type: Less important

##### Research needed

Research needed: 2.3. Conservation Planning - Harvest & Trade Management Plan3.1. Monitoring - Population trends3.2. Monitoring - Harvest level trends3.3. Monitoring - Trade trends

Justification for research needed: The Government of Mexico should collaborate with Mexican tarantula breeders to develop a system for certifying the origin of specimens used in the breeding programmes of Units for Management (UMAs). Additionally, systematic monitoring and protection could be undertaken in known subpopulations. Further studies are needed to confirm population, harvest and trade trends and how the latter affect harvest levels.

#### Use and trade

Use type: International

Use and trade: 13. Pets/display animals, horticulture16. Establishing ex-situ production *

#### Ecosystem services

Ecosystem service type: Less important

#### Research needed

Research needed: 2.3. Conservation Planning - Harvest & Trade Management Plan3.1. Monitoring - Population trends3.2. Monitoring - Harvest level trends3.3. Monitoring - Trade trends

Justification for research needed: The Government of Mexico should collaborate with Mexican tarantula breeders to develop a system for certifying the origin of specimens used in the breeding programmes of Units for Management (UMAs). Additionally, systematic monitoring and protection could be undertaken in known subpopulations. Further studies are needed to confirm population, harvest and trade trends and how the latter affect harvest levels.

#### Viability analysis

### Brachypelma boehmei

#### Species information

Scientific name: Brachypelma boehmei

Species authority: Schmidt & Klaas, 1993

Common names: Mexican fireleg, Mexican rustleg, tarántula de piernas oxidadas, tarántula mexicana pierna naranja oscuro, mygale du Mexique à pattes rouille.

Kingdom: Animalia

Phylum: Arthropoda

Class: Arachnida

Order: Araneae

Family: Theraphosidae

**Figure 18.**
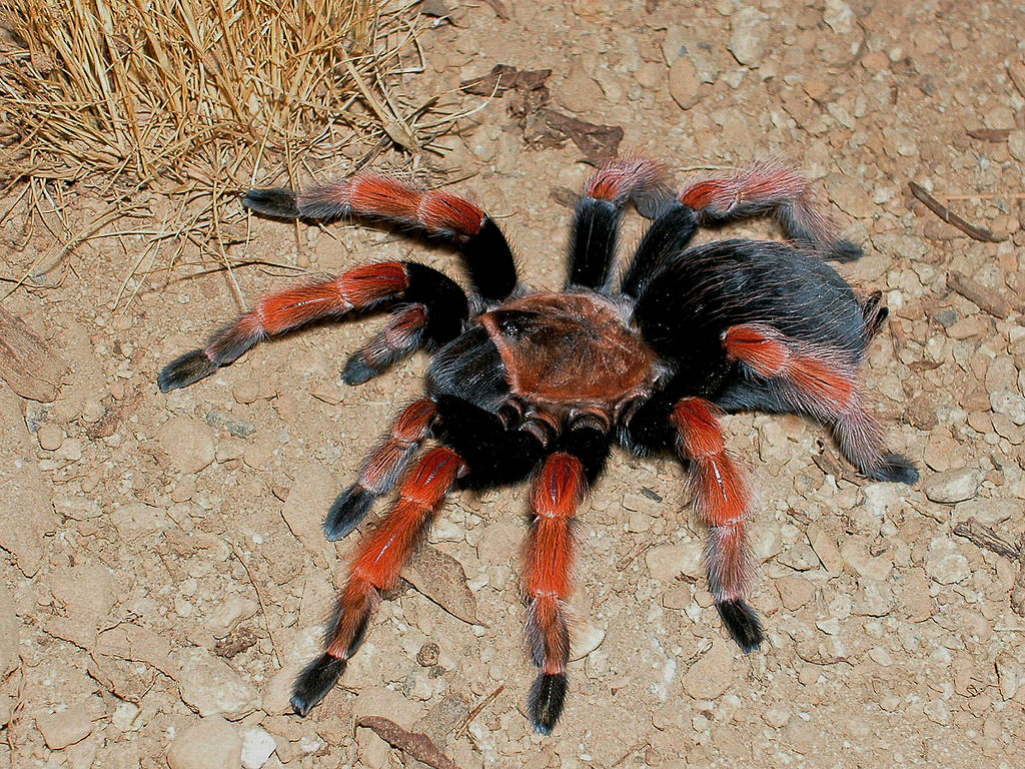
*Brachypelma boehmei* Schmidt & Kraus 1993, female from north of Ixtapa, Guerrero state, Mexico. Photo: Rick C. West.

**Figure 19.**
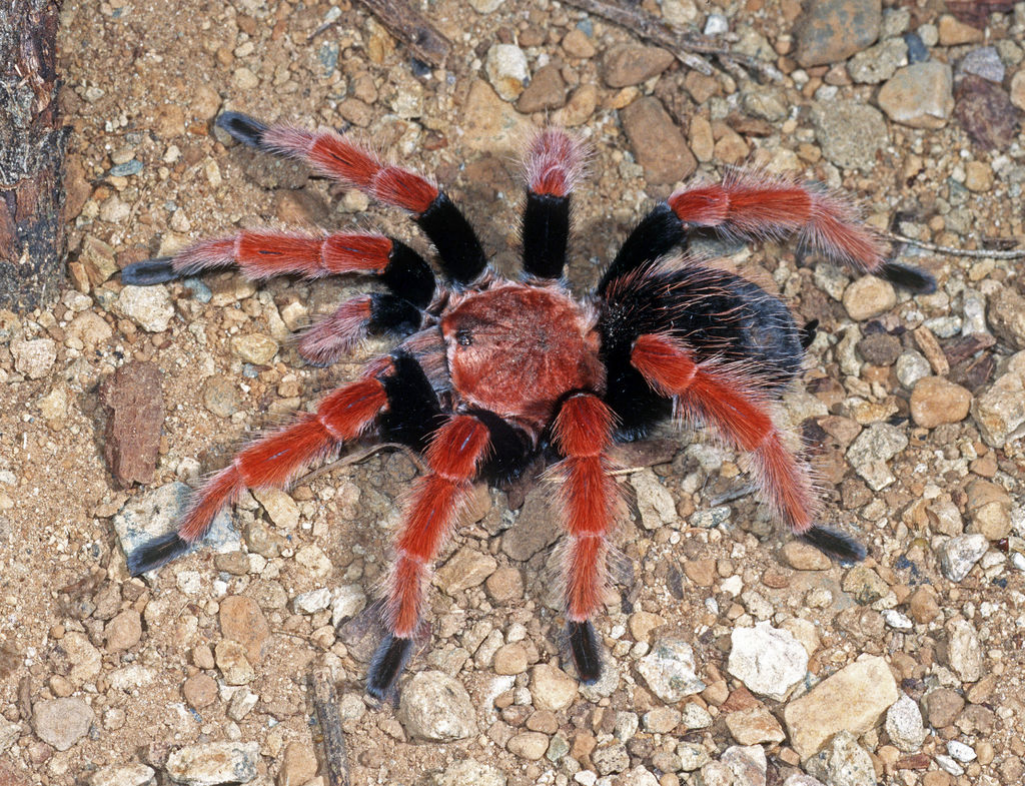
*Brachypelma boehmei* Schmidt & Klaas, 1993 male from north of Ixtapa, Guerrero state, Mexico. Photo: Rick C. West.

Figure(s) or Photo(s): Figs 18, 19

Region for assessment: Global

#### Geographic range

Biogeographic realm: Neotropical

Countries: Mexico

**Figure 20.**
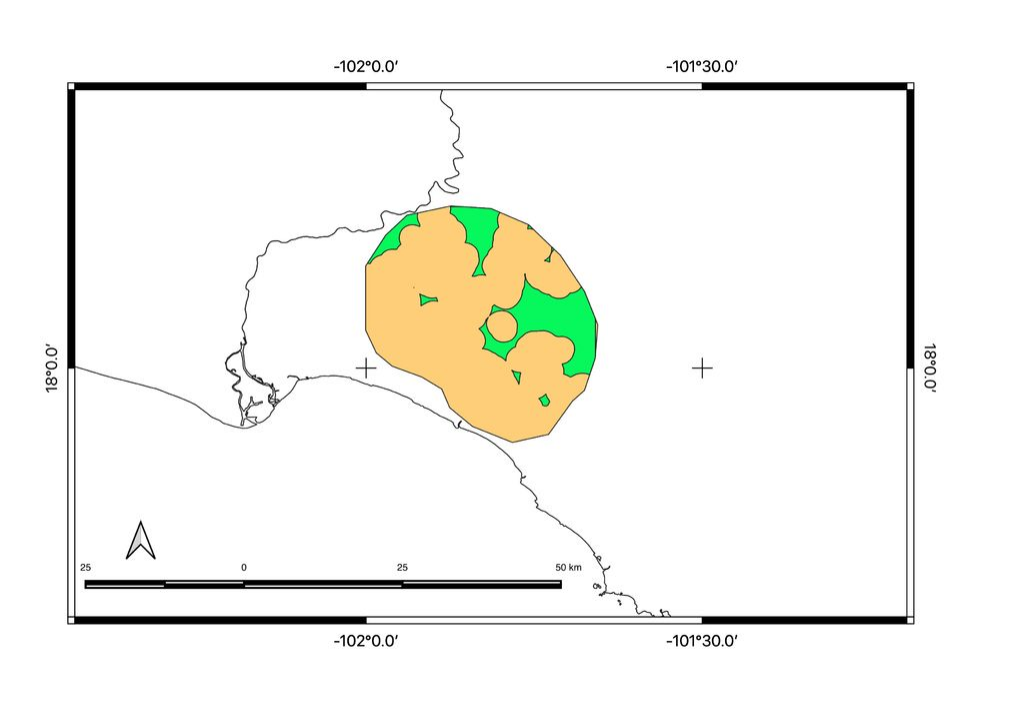
Habitat quality across the distribution of *Brachypelma boehmei* Schmidt & Klaas, 1993. Low quality habitat areas (LQH) in orange and high quality habitat areas (HQH) in green (for estimation details, see formula in Methods).

Map of records (image): Fig. 20

Map of records (Google Earth): Suppl. material 8

Basis of EOO and AOO: Species Distribution Model

Basis (narrative): Despite few collection sites recorded for this species, it was possible to perform species distribution modelling to predict its potential range. See methods for details.

Min Elevation/Depth (m): 100

Max Elevation/Depth (m): 320

Range description: *Brachypelma boehmei* is endemic to Mexico and can be found in a small coastal region of the Sierra Madre del Sur, east of the Balsas River Basin in western Guerrero State (Schmidt and Klaas 1993, Mendoza and Francke 2017, CEC 2017, iNaturalist 2018, unpublished data). The western border of distribution is the wide Balsas river. This species follows the Pacific coast until areas where *Brachypelma smithi* occurs. In the North, the high Sierra Madre del Sur mountain range separates *Brachypelma boehmei* from *Brachypelma auratum* further inland (Hijmensen 2018).

#### New occurrences

#### Extent of occurrence

EOO (km2): 396

Trend: Decline (inferred)

Justification for trend: Human pressure, such as urbanisation and agriculture, are affecting the extent of occurrence, as the species seems to be dependent on well-preserved forests.

Causes ceased?: No

Causes understood?: Yes

Causes reversible?: No

Extreme fluctuations?: No

#### Area of occupancy

Trend: Decline (inferred)

Justification for trend: Human pressure, such as urbanisation and agriculture, are affecting the area of occupancy, as the species seems to be dependent on well-preserved forests.

Causes ceased?: No

Causes understood?: Yes

Causes reversible?: No

Extreme fluctuations?: No

AOO (km2): 264

#### Locations

Number of locations: 14

Justification for number of locations: Derived from the approximate number of human populations above 100 inhabitants needed to cover the entire range of the species. We assume each locality covers a mean radius of 2.5 km.

Trend: Decline (inferred)

Justification for trend: We assume the number of locations to be decreasing, given the generalised loss of subpopulations of this species. Experts attending the Tarantula Trinational Trade and Enforcement Workshop (Jalisco, México, 2018), identified smuggling as a continuous threat over this species range. Based on their experience and field observations, some smugglers travel from 1 to 5 km from their towns in order to catch this species in the wild for the black market. Taking into consideration this fact, we mapped and quantified the number of villages within tarantula’s distribution using GIS, a database of Mexican villages (CONABIO 2014) and a buffer area of 2.5 km around villages. Considering that agglomerations of villages are acting as single threats (because contiguous urban areas have no physical barriers between them), the number of locations for this species is 14.

Extreme fluctuations?: No

#### Population

Number of individuals: Unknown

Trend: Decline (inferred)

Justification for trend: Despite the population size being unknown, it is possible to infer its decline since the AOO (Global Forest Watch 2019), EOO and the quality of habitat are decreasing. There is also increasing harvesting pressure to the few subpopulations remaining. Human pressure, such as changing land use through urbanisation and agriculture, are the main causes of the inferred decline since the species seems to be forest-dependent and does not appear to adapt well to disturbed habitats. In addition, some subpopulations are known to have been devastated by hurricanes. Since it is a desirable species in the pet trade and legal breeding in captivity is too low to meet demands, illegal collection of wild specimens is increasing, the activity being facilitated by road constructions. Finally, like other *Brachypelma* species which occur in ranges around the north-south coastal Pacific highway, many males of *B. boehmei* are run over while crossing roads during the mating season.

Basis for decline: (c) a decline in area of occupancy, extent of occurrence and/or quality of habitat(d) actual or potential levels of exploitation

Causes ceased?: No

Causes understood?: Yes

Causes reversible?: No

Extreme fluctuations?: No

Population Information (Narrative): Despite the population size being unknown, it is possible to infer its decline since both the AOO and the quality of habitat are decreasing (Global Forest Watch 2019). There is also increasing harvesting pressure to the few subpopulations remaining. Human pressure, such as changing land use through urbanisation and agriculture, are the main causes of the inferred decline since the species seems to be forest-dependent and does not appear to adapt well to disturbed habitats. In addition, some subpopulations are known to have been devastated by hurricanes. Since it is a desirable species in the pet trade and legal breeding in captivity is too low to meet the demands, harvesting pressure on wild populations is increasing. Finally, like other *Brachypelma* species which occur in ranges around the north-south coastal Pacific highway, many males of *B. boehmei* are run over while crossing roads during the mating season.

#### Subpopulations

Number of subpopulations: Unknown

Trend: Decline (inferred)

Justification for trend: Subpopulations are expected to be declining given the strong human pressure and severe fragmentation.

Extreme fluctuations?: No

Severe fragmentation?: Yes

Justification for fragmentation: Based on the analysis of the relative abundance of each species in low quality habitat (RA_LQH_) and the Species Distribution Model, 81.01% of the population should be in subpopulations that are non-viable and without the possibility of rescue effects due to fragmentation.

#### Habitat

System: Terrestrial

Habitat specialist: Yes

Habitat (narrative): *Brachypelma boehmei* inhabits the subtropical dry forest (xeric area with thorn forest) in the foothills of the Sierra Madre del Sur mountains, primarily in the lowlands near the Pacific coast. This species appears to prefer shady forested areas and healthy populations are not found outside this type of habitat.

Trend in extent, area or quality?: Decline (inferred)

Justification for trend: The habitat of this species is inferred to be declining in area, extent and quality due to expansion of urbanisation and agriculture, both of which appear as important factors reducing remaining habitat of this species, which does not adapt well to disturbance (appearing to be a forest-specialist). The construction and expansion of roads are also destroying habitat and for remaining subpopulations adjacent them, the major pacific transit routes in this region provide both a barrier to dispersal and a cause of mortality as males, in particular, appear to often be run over by traffic. Adding to this, the construction of roads also facilitates the access of poachers to some areas, leading to a declining of wild populations. Natural phenomena, such as hurricanes, have also likely caused a decrease in the quality of its coastal habitat.

**Figure 21.**
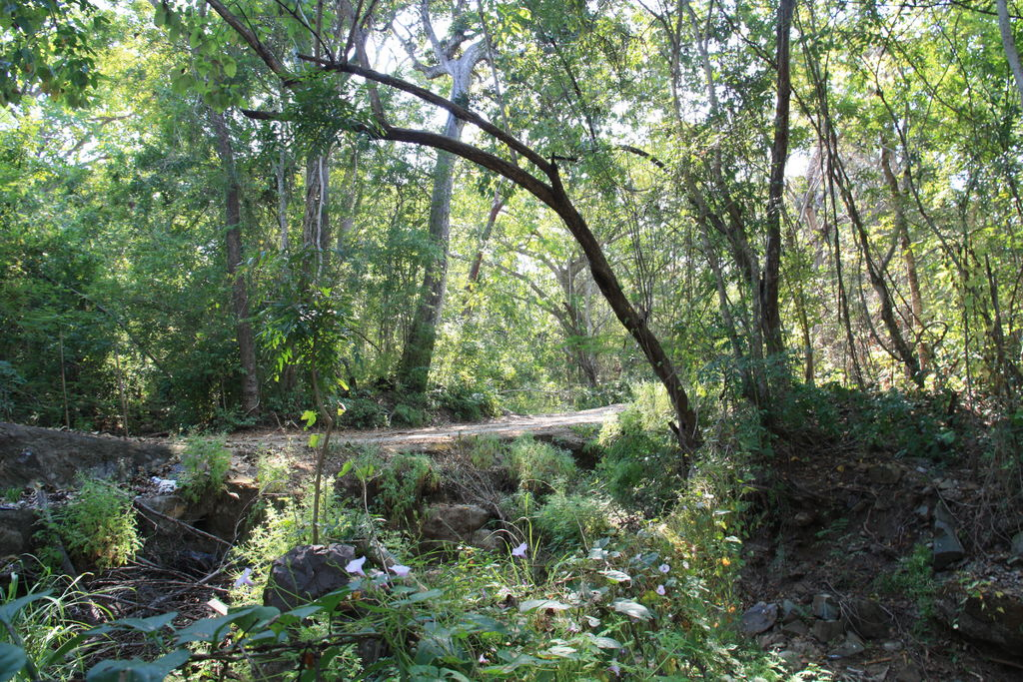
*Brachypelma boehmei* habitat, subtropical dry forest and thorn brush, north of Ixtapa, Guerrero state, Guerrero state, Mexico. Photo: Stuart J. Longhorn.

Figure(s) or Photo(s): Fig. 21

##### Habitat

Habitat importance: Major Importance

Habitats: 1.5. Forest - Subtropical/Tropical Dry

#### Habitat

Habitat importance: Major Importance

Habitats: 1.5. Forest - Subtropical/Tropical Dry

#### Ecology

Size: 75 mm (female); 65 mm (male).

Generation length (yr): 7

Dependency of single sp?: No

Ecology and traits (narrative): *Brachypelma boehmei* is a fossorial species that modifies previously excavated burrows or can excavate their own unaided, often only making minor alterations to natural small cavities under debris, such as large rocks and tree roots in dense vegetation of dry forests and thorny brush. They can be found on both sloped and level ground. Burrows mostly do not have any silk around the entrance, giving no clear indication that there is a spider inside. These spiders are nocturnal predators that wait near the entrance of their refuge from dusk and into the night to feed primarily on ground-dwelling arthropods (insects, other arachnids and some myriapods) or even small vertebrates. Prey remains, found in burrows, have consisted mostly of beetles and millipedes. (West 2005; pers. obs.). Juvenile specimens were additionally found under fallen rotting wood lying on the ground. One female specimen was found cohabiting with a 30 centimetres long Balsas ground snake (*Sonora michoacanensis* Duges, 1884). The mating season occurs during the last part of the rainy and first part of dry seasons (August to January) when mature males wander in the open to search for females. The males are likely most active at night, cooler daylight hours and throughout overcast days. Adult females typically moult once per year, just prior to the onset of the annual male emergence. Females will produce cocoons (large silken egg sacs) during the drier winter months with young emerging about two months later, with most young dispersing in the late spring or summer, just before the onset of the early summer rains.

#### Threats

Justification for threats: Red leg species are more popular as pets than the red abdomen ones. There is a legal trade of captive-bred live specimens, but also an unknown amount of trafficking of live animals. Urbanisation and agricultural impacts also threaten this species, since they cause a decline in area and quality of habitat, as well as leading to severe fragmentation of the population of *B. boehmei*. This species, along with the others of the red leg complex, are more fragile and do not adapt well or quickly to alterations in the environment caused by human activities (Turner et al. 2018). Local information indicates that some subpopulations were devastated by hurricanes. Vehicular traffic on highways can also be considered a threat to *B. boehmei* males, since similar to the other *Brachypelma* species that occur along the north-south coastal Pacific highway, they are run over by cars while crossing roads during mating season. Adding to this, roads facilitate the access of smugglers to wild populations and also are a source *per se* of habitat degradation.

##### Threats

Threat type: Ongoing

Threats: 1.1. Residential & commercial development - Housing & urban areas2.1.2. Agriculture & aquaculture - Annual & perennial non-timber crops - Small-holder farming2.1.3. Agriculture & aquaculture - Annual & perennial non-timber crops - Agro-industry farming4.1. Transportation & service corridors - Roads & railroads5.1.1. Biological resource use - Hunting & trapping terrestrial animals - Intentional use (species is the target)11.4. Climate change & severe weather - Storms & flooding

#### Threats

Threat type: Ongoing

Threats: 1.1. Residential & commercial development - Housing & urban areas2.1.2. Agriculture & aquaculture - Annual & perennial non-timber crops - Small-holder farming2.1.3. Agriculture & aquaculture - Annual & perennial non-timber crops - Agro-industry farming4.1. Transportation & service corridors - Roads & railroads5.1.1. Biological resource use - Hunting & trapping terrestrial animals - Intentional use (species is the target)11.4. Climate change & severe weather - Storms & flooding

#### Conservation

Justification for conservation actions: As there is no official protected area on the range of *B. boehmei*, the creation of a conservation unit in the north part of Guerrero State would be an effective action to preserve subpopulations of *B. boehme*i together with *B. smithi*. It is also necessary to establish management plans and conduct systematic monitoring to provide information for population recovery and to develop more programmes of species re-introduction, such as the one that is being outlined by a Mexican Unit for Management (UMA). In order to avoid international trade incompatible with its survival, this species is currently listed on CITES Appendix II, along with all the other species of the genus *Brachypelma* (CITES 2019). This species is being reared in captivity in Mexican Units for Managements (UMAs) and sold legally. However, as *B. boehmei* is part of the illegal pet trade, it is necessary to develop better enforcement actions to curb it, as well as to establish tax advantages for legal dealers in order to make their prices more competitive with the ones in the black market. Currently, this species is being reared in captivity in a Mexican Unit for Management (UMA) that also promotes educational and awareness activities regarding tarantula spiders aimed at students and the general public. Adding to this, *B. boehmei* could be used as a flagship species to motivate public involvement in tarantula conservational efforts in the Pacific coastal area since it is a very attractive species.

##### Conservation actions

Conservation action type: In Place

Conservation actions: 3.3.1. Species management - Species re-introduction - Reintroduction3.4.1. Species management - Ex-situ conservation - Captive breeding/artificial propagation5.1.1. Law & policy - Legislation - International level4.3. Education & awareness - Awareness & communications

##### Conservation actions

Conservation action type: Needed

Conservation actions: 1.1. Land/water protection - Site/area protection1.2. Land/water protection - Resource & habitat protection3.1.1. Species management - Species management - Harvest management3.1.2. Species management - Species management - Trade management3.2. Species management - Species recovery5.4.1. Law & policy - Compliance and enforcement - International level5.4.2. Law & policy - Compliance and enforcement - National level5.4.3. Law & policy - Compliance and enforcement - Sub-national level6.4. Livelihood, economic & other incentives - Conservation payments

#### Conservation actions

Conservation action type: In Place

Conservation actions: 3.3.1. Species management - Species re-introduction - Reintroduction3.4.1. Species management - Ex-situ conservation - Captive breeding/artificial propagation5.1.1. Law & policy - Legislation - International level4.3. Education & awareness - Awareness & communications

#### Conservation actions

Conservation action type: Needed

Conservation actions: 1.1. Land/water protection - Site/area protection1.2. Land/water protection - Resource & habitat protection3.1.1. Species management - Species management - Harvest management3.1.2. Species management - Species management - Trade management3.2. Species management - Species recovery5.4.1. Law & policy - Compliance and enforcement - International level5.4.2. Law & policy - Compliance and enforcement - National level5.4.3. Law & policy - Compliance and enforcement - Sub-national level6.4. Livelihood, economic & other incentives - Conservation payments

#### Other

Justification for use and trade: *Brachypelma boehmei* is being reared in captivity in Mexican Units for Management (UMA) and sold legally. This species is currently listed on CITES Appendix II and thus its international trade is regulated by an international agreement (CITES 2019). There is legal trade of captive-bred live specimens, but also an unknown amount of trafficking of live animals, including wild-caught gravid females. These are occasionally bred in Europe, but available numbers do not appear to satisfy demand. Stocks for some Mexican breeders came from a German seizure and were repatriated in 2005 (CEC 2017). Captive-bred juveniles are sold for approximately US$30–$45 in Canada and the United States, for US$10 in Mexico and for US$8 in the EU; adult males are sold for US$90 in the United States and adult females for approximately US$100–$250 in Canada and the United States and for US$67 in the EU (CEC 2017). It is considered a very common species in the international trade since between 7,810–10,021 specimens of *B. boehmei* were traded during 2006–2016, all as live specimens for commercial purposes, none declared as wild-caught (Cooper et al. 2019).

##### Use and trade

Use type: International

Use and trade: 13. Pets/display animals, horticulture16. Establishing ex-situ production *

##### Ecosystem services

Ecosystem service type: Less important

##### Research needed

Research needed: 2.3. Conservation Planning - Harvest & Trade Management Plan3.1. Monitoring - Population trends3.2. Monitoring - Harvest level trends3.3. Monitoring - Trade trends

Justification for research needed: The Government of Mexico could collaborate with Mexican tarantula breeders to develop a system for certifying the origin of specimens used in the breeding programmes of the Units for Management (UMA). Additionally, systematic monitoring and protection could be undertaken in known subpopulations. Further studies are needed to confirm population, harvest and trade trends and how the latter affect harvest levels.

#### Use and trade

Use type: International

Use and trade: 13. Pets/display animals, horticulture16. Establishing ex-situ production *

#### Ecosystem services

Ecosystem service type: Less important

#### Research needed

Research needed: 2.3. Conservation Planning - Harvest & Trade Management Plan3.1. Monitoring - Population trends3.2. Monitoring - Harvest level trends3.3. Monitoring - Trade trends

Justification for research needed: The Government of Mexico could collaborate with Mexican tarantula breeders to develop a system for certifying the origin of specimens used in the breeding programmes of the Units for Management (UMA). Additionally, systematic monitoring and protection could be undertaken in known subpopulations. Further studies are needed to confirm population, harvest and trade trends and how the latter affect harvest levels.

#### Viability analysis

### Brachypelma emilia

#### Species information

Scientific name: Brachypelma emilia

Species authority: (White, 1856)

Synonyms: *Mygale emilia* White, 1856; *Eurypelma emilia* (White, 1856); *Brachypelma aemilia* (White, 1856); *Euathlus emilia* (White, 1856).

Common names: Mexican redleg, tarántula mexicana de piernas rojas, tarántula mexicana pierna roja, mygale du Mexique à pattes rouges.

Kingdom: Animalia

Phylum: Arthropoda

Class: Arachnida

Order: Araneae

Family: Theraphosidae

**Figure 22.**
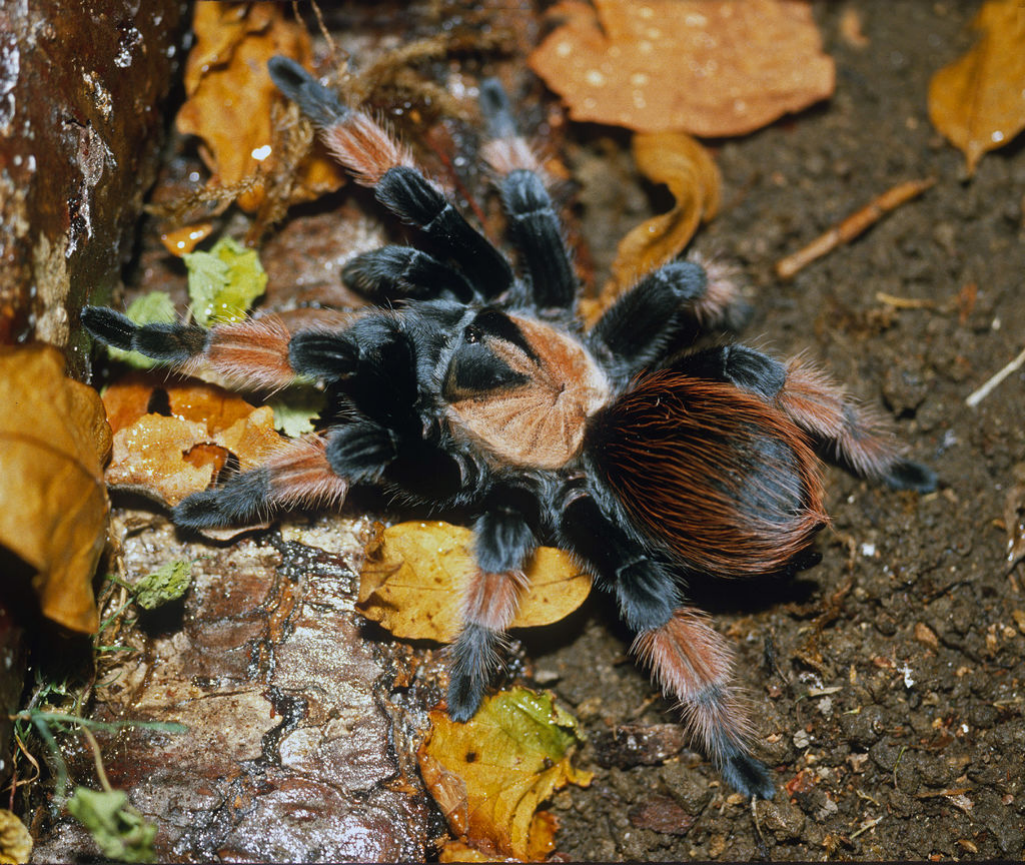
*Brachypelma emilia* (White, 1856) female near San Blas, Nayarit state, Mexico. Photo: Rick C. West.

**Figure 23.**
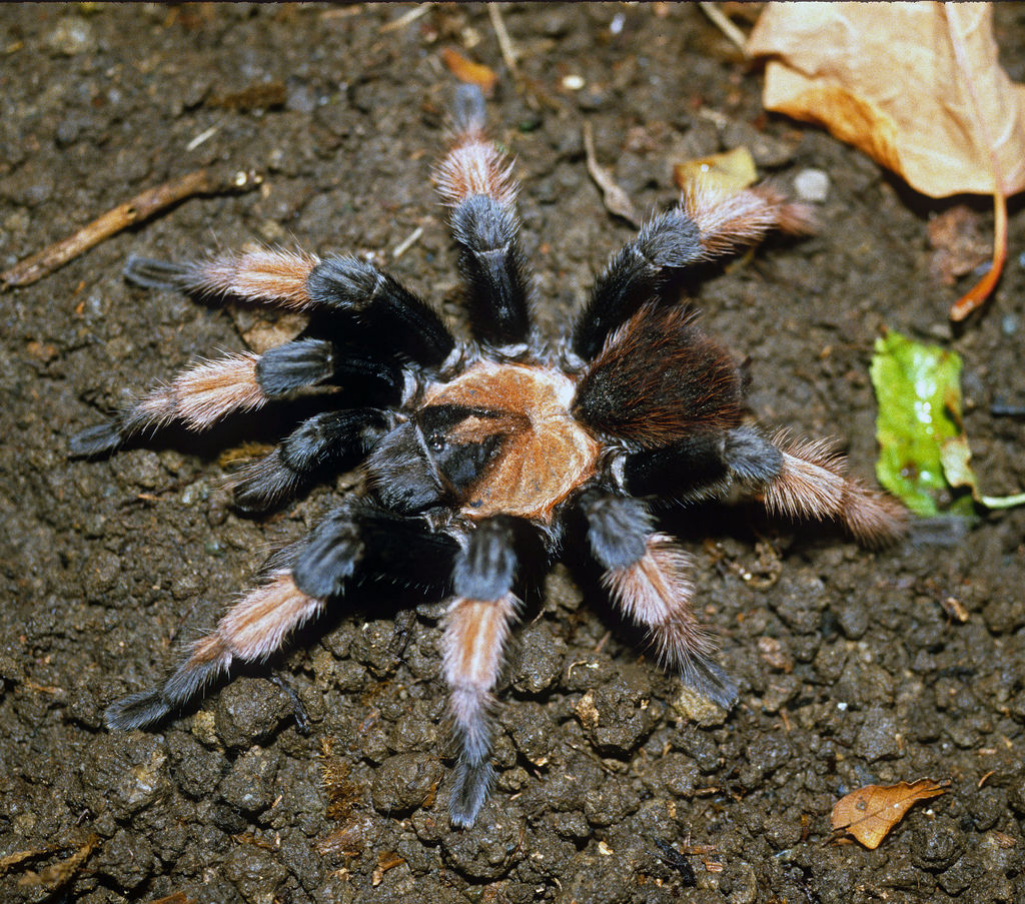
*Brachypelma emilia* (White, 1856) male near Las Varas, Nayarit state, Mexico. Photo: Rick C. West.

Figure(s) or Photo(s): Figs 22, 23

Region for assessment: Global

#### Geographic range

Biogeographic realm: Neotropical

Countries: Mexico

**Figure 24.**
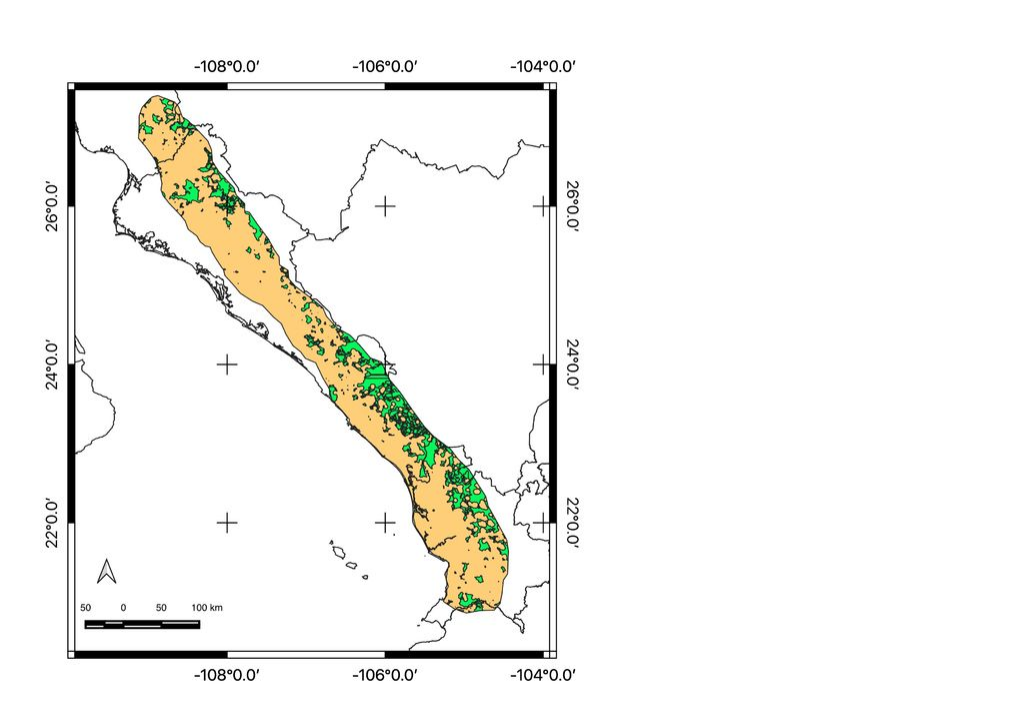
Habitat quality across the distribution of *Brachypelma emilia* (White, 1856). Low quality habitat areas (LQH) in orange and high quality habitat areas (HQH) in green (for estimation details, see formula in Methods).

Map of records (image): Fig. 24

Map of records (Google Earth): Suppl. material 9

Basis of EOO and AOO: Species Distribution Model

Basis (narrative): A species distribution modelling has been performed to predict its potential range. See methods for details.

Min Elevation/Depth (m): 0

Max Elevation/Depth (m): 2030

Range description: *Brachypelma emilia* is endemic to Mexico and occurs on the coastal plain, west of the Sierra Madre Occidental, from southern Sonora State, in Sinaloa State, south-western Nayarit State and inland to western Durango State (White 1856, Pickard-Cambridge 1897, Smith 1986, CEC 2017, iNaturalist 2018 unpublished data). Recently a small population of *B. klaasi* was found in Nayarit, resulting in sympatry with *B. emilia* in this area. Considering the range of all *Brachypelma* species, *B. emilia* has the most northern range (Hijmensen 2018), not reaching the north-western part of Jalisco.

#### New occurrences

#### Extent of occurrence

EOO (km2): 75867

Trend: Decline (inferred)

Justification for trend: The species habitat is being lost due to urbanisation, possibly affecting the EOO.

Causes ceased?: No

Causes understood?: Yes

Causes reversible?: No

Extreme fluctuations?: No

#### Area of occupancy

Trend: Decline (inferred)

Justification for trend: The species habitat is being lost due to urbanisation, affecting the AOO.

Causes ceased?: No

Causes understood?: Yes

Causes reversible?: No

Extreme fluctuations?: No

AOO (km2): 34236

#### Locations

Number of locations: Unknown

Justification for number of locations: Given the large range, the number of locations is much above that of any category thresholds.

Trend: Unknown

Extreme fluctuations?: No

#### Population

Number of individuals: Unknown

Trend: Decline (inferred)

Justification for trend: A decline in population size is inferred since it is being overharvested for trade and also subject to decline in AOO and EOO quality of habitat due to human activities, such as urbanisation.

Basis for decline: (c) a decline in area of occupancy, extent of occurrence and/or quality of habitat(d) actual or potential levels of exploitation

Causes ceased?: No

Causes understood?: Yes

Causes reversible?: No

Extreme fluctuations?: No

Population Information (Narrative): Unknown population size. A decline in population size is inferred since it is being overharvested for trade and also subject to decline in AOO and EOO due to human activities, such as urbanisation.

#### Subpopulations

Number of subpopulations: Unknown

Trend: Decline (inferred)

Justification for trend: A decline in number of subpopulations is inferred since it is being overharvested for trade and also subject to decline in AOO and quality of habitat due to human activities, such as urbanisation.

Extreme fluctuations?: No

Severe fragmentation?: Yes

Justification for fragmentation: Based on the analysis of the relative abundance of each species in low quality habitat (RA_LQH_) and the Species Distribution Model, 83.29% of the population should be in subpopulations that are non-viable and without the possibility of rescue effects due to fragmentation.

#### Habitat

System: Terrestrial

Habitat specialist: No

Habitat (narrative): *Brachypelma emilia* inhabits the semi-arid lowlands and the deciduous dry forest slopes of the Sierra Madre Occidental mountains (West 2005). It can also sometimes be found in more human disturbed areas such as avocado groves and edges of pastures and croplands of maize, particularly under large piles of soil and rocks, even occasionally, for example, on the sides of roads.

Trend in extent, area or quality?: Decline (inferred)

Justification for trend: The habitat of this species is inferred to be declining in area, extent and quality due to human activity, especially on more lowland coastal areas. The development of roads are one of the main threats to this species since it occurs within many regions of intense traffic and males are frequently found run over on the highways.

**Figure 25.**
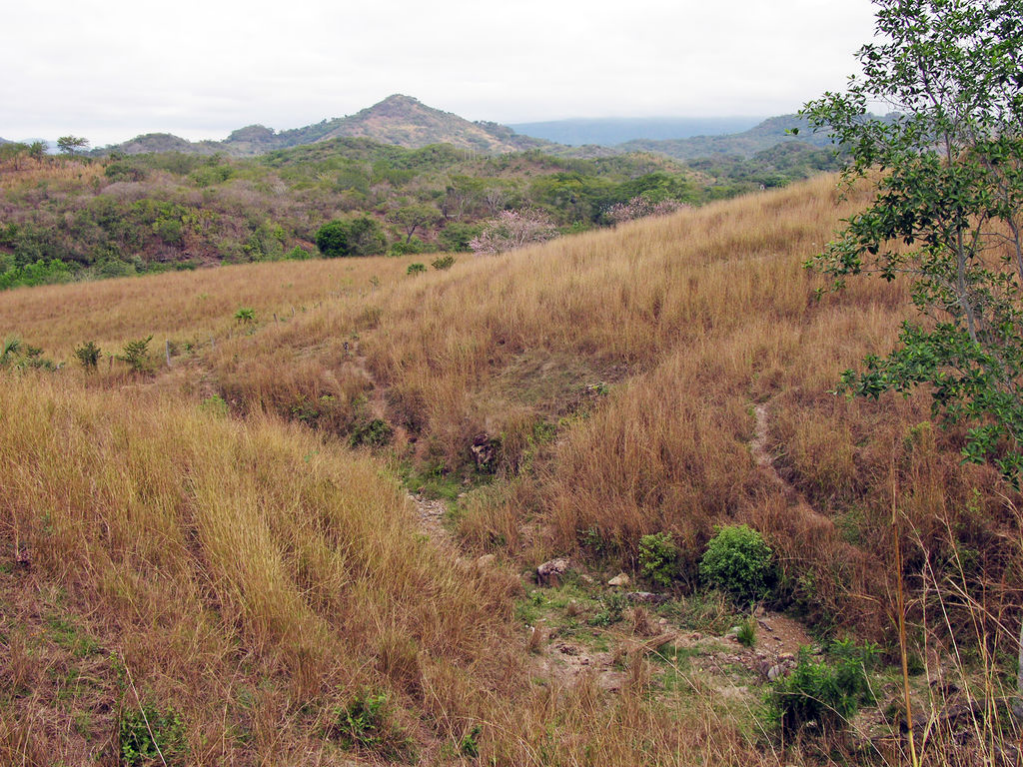
*Brachypelma emilia* habitat near Las Varas, Nayarit state, Mexico. Photo: Rick C. West.

Figure(s) or Photo(s): Fig. 25

##### Habitat

Habitat importance: Major Importance

Habitats: 1.5. Forest - Subtropical/Tropical Dry

##### Habitat

Habitat importance: Marginal

Habitats: 14.2. Artificial/Terrestrial - Pastureland14.3. Artificial/Terrestrial - Plantations

#### Habitat

Habitat importance: Major Importance

Habitats: 1.5. Forest - Subtropical/Tropical Dry

#### Habitat

Habitat importance: Marginal

Habitats: 14.2. Artificial/Terrestrial - Pastureland14.3. Artificial/Terrestrial - Plantations

#### Ecology

Size: 70 mm (female); 60 mm (male).

Generation length (yr): 7

Dependency of single sp?: No

Ecology and traits (narrative): *Brachypelma emilia* is a fossorial species that modifies previously excavated burrows or can excavate their own unaided, often clearly defined obligate burrow-like retreats under debris, such as large rocks and tree roots in subtropical dry forest and occasionally in moderately disturbed areas on either level ground or sloped ground. Burrows can sometimes be found in avocado groves, edges of pastures and maize croplands or amongst large piles of soil and rocks with some vegetation cover on the sides of roads (West 2005, Hijmensen 2018). Some can be also found very close to houses and other human structures (West 2005). Burrows mostly do not have any silk around the entrance, giving no clear indication that there is a spider inside (West 2005). The interior can often be multi-tunnelled between 20-100 centimetres in length and ending in a large chamber. These spiders are nocturnal predators that wait near the entrance of their refuge from dusk and into the night to feed primarily on ground-dwelling arthropods (insects, other arachnids and some myriapods) or even small vertebrates. One female *B. emilia* from the southernmost part of the range in Nayarit State was observed to catch and start eating an adult lizard *Ameiva undulata* (Wiegmann, 1834). The mating season occurs during the last part of the rainy and first part of dry seasons (August to January) when mature males wander in the open to search for females. The males are likely most active at night, cooler daylight hours and throughout overcast days. Adult females typically moult once per year, just prior to the onset of the annual male emergence. Females will produce cocoons (large silken egg sacs) during the drier winter months with young emerging about two months later, with most young dispersing in the late spring or summer, just before the onset of the early summer rains.

#### Threats

Justification for threats: The major threat to *B. emilia* is habitat loss, especially in coastal areas, due to human construction. This species, along with the others of the red leg complex, are more fragile and do not adapt well or quickly to alterations on the environment caused by human activities (Turner et al. 2018). Roads are also relevant threats to this species since *B. emilia* occurs near many roads with intense traffic and males are frequently found run over on them during the mating season. Finally, considering that the red leg species are more popular as pets than the red abdomen ones, illegal harvest and trade are also important factors across its range and it is also facilitated by construction of roads since they make the access to wild populations easier.

##### Threats

Threat type: Ongoing

Threats: 1.1. Residential & commercial development - Housing & urban areas4.1. Transportation & service corridors - Roads & railroads5.1.1. Biological resource use - Hunting & trapping terrestrial animals - Intentional use (species is the target)

#### Threats

Threat type: Ongoing

Threats: 1.1. Residential & commercial development - Housing & urban areas4.1. Transportation & service corridors - Roads & railroads5.1.1. Biological resource use - Hunting & trapping terrestrial animals - Intentional use (species is the target)

#### Conservation

Justification for conservation actions: The Pacific coast of Mexico is one of the most human-disturbed areas of the country and encompasses several different species of *Brachypelma*, including *B. emilia*. Thus it is a priority area regarding conservation planning and actions for the genus. Although the real occurrence of this species remains to be evaluated, some protected areas, such as Sierra de Vallejo State Biosphere Reserve and Sierra de Álamos Natural Protection Area, are inside the range of the species and may be suitable for developing conservation initiatives. Systematic monitoring and re-introduction of specimens in depleted areas should be undertaken, especially around Jalisco, where *B. emilia* has been harvested by local people in large numbers. In order to avoid international trade incompatible with its survival, this species is currently listed on CITES Appendix II, along with all other species of the genus *Brachypelma* (CITES 2019). *Brachypelma emilia* is already being reared in captivity in Mexican Units for Management (UMAs) and sold legally. However, better enforcement actions to curb trafficking are necessary, as well as to establish tax advantages for legal dealers in order to make their prices more competitive with the ones in the black market. Furthermore, the government of Mexico could collaborate with Mexican tarantula breeders to develop a system for certifying the origin of specimens used in the breeding programmes of UMAs. Currently, this species is being reared in captivity in a UMA that also promotes educational and awareness activities regarding tarantula spiders aimed at students and the general public.

##### Conservation actions

Conservation action type: In Place

Conservation actions: 1.1. Land/water protection - Site/area protection3.4.1. Species management - Ex-situ conservation - Captive breeding/artificial propagation5.1.1. Law & policy - Legislation - International level4.3. Education & awareness - Awareness & communications

##### Conservation actions

Conservation action type: Needed

Conservation actions: 1.1. Land/water protection - Site/area protection1.2. Land/water protection - Resource & habitat protection2.3. Land/water management - Habitat & natural process restoration3.1. Species management - Species management5.3. Law & policy - Private sector standards & codes5.4.1. Law & policy - Compliance and enforcement - International level5.4.2. Law & policy - Compliance and enforcement - National level5.4.3. Law & policy - Compliance and enforcement - Sub-national level6.4. Livelihood, economic & other incentives - Conservation payments

#### Conservation actions

Conservation action type: In Place

Conservation actions: 1.1. Land/water protection - Site/area protection3.4.1. Species management - Ex-situ conservation - Captive breeding/artificial propagation5.1.1. Law & policy - Legislation - International level4.3. Education & awareness - Awareness & communications

#### Conservation actions

Conservation action type: Needed

Conservation actions: 1.1. Land/water protection - Site/area protection1.2. Land/water protection - Resource & habitat protection2.3. Land/water management - Habitat & natural process restoration3.1. Species management - Species management5.3. Law & policy - Private sector standards & codes5.4.1. Law & policy - Compliance and enforcement - International level5.4.2. Law & policy - Compliance and enforcement - National level5.4.3. Law & policy - Compliance and enforcement - Sub-national level6.4. Livelihood, economic & other incentives - Conservation payments

#### Other

Justification for use and trade: *Brachypelma emilia* is being reared in captivity in Mexican Units for Management (UMAs) and sold legally. This species is currently listed on CITES Appendix II and thus its international trade is regulated by an international agreement (CITES 2019). There is a legal trade of captive-bred live specimens, but also an unknown amount of trafficking of live animals. Specimens are known to be collected at least around Jalisco region by local people. Captive-bred juveniles are sold for approximately US$30–$35 in Canada and the United States, for US$10 in Mexico and for US$13 in the EU; adult females are sold for approximately US$300 in the United States and for US$40 in the EU (CEC 2017). This species, along with *B. auratum*, are replacing *B. smithi* in the market, thus it is common to find it in the black market. Specimens of 5 cm are the most common size sold and adults are not commonly traded. This species is considered very common in the international trade since between 6,133–7,496 *B. emilia* were traded internationally during 2006–2016, all as live specimens and none declared as wild-caught, mostly being traded for commercial purposes (Cooper et al. 2019).

##### Use and trade

Use type: International

Use and trade: 13. Pets/display animals, horticulture16. Establishing ex-situ production *

##### Ecosystem services

Ecosystem service type: Less important

##### Research needed

Research needed: 2.3. Conservation Planning - Harvest & Trade Management Plan3.1. Monitoring - Population trends3.2. Monitoring - Harvest level trends3.3. Monitoring - Trade trends

Justification for research needed: The Government of Mexico could collaborate with Mexican tarantula breeders to develop a system for certifying the origin of specimens used in the breeding programmes of the Units for Management (UMA). Systematic monitoring and protection could be undertaken within known subpopulations. Trade trends and how these affect harvest levels must be further studied.

#### Use and trade

Use type: International

Use and trade: 13. Pets/display animals, horticulture16. Establishing ex-situ production *

#### Ecosystem services

Ecosystem service type: Less important

#### Research needed

Research needed: 2.3. Conservation Planning - Harvest & Trade Management Plan3.1. Monitoring - Population trends3.2. Monitoring - Harvest level trends3.3. Monitoring - Trade trends

Justification for research needed: The Government of Mexico could collaborate with Mexican tarantula breeders to develop a system for certifying the origin of specimens used in the breeding programmes of the Units for Management (UMA). Systematic monitoring and protection could be undertaken within known subpopulations. Trade trends and how these affect harvest levels must be further studied.

#### Viability analysis

### Brachypelma epicureanum

#### Species information

Scientific name: Brachypelma epicureanum

Species authority: (Chamberlin, 1925)

Synonyms: *Eurypelma epicureana* Chamberlin, 1925; *Dugesiella epicureana* (Chamberlin, 1925); *Rhechostica epicureana* (Chamberlin, 1925); *Avicularia epicureana* (Chamberlin, 1925); *Aphonopelma epicureanum* (Chamberlin, 1925).

Common names: Yucatán rustrump, tarántula trasero oxidado de Yucatán, mygale à abdomen rouille du Yucatán.

Kingdom: Animalia

Phylum: Arthropoda

Class: Arachnida

Order: Araneae

Family: Theraphosidae

Taxonomic notes: Mendonza and Francke (in press) are publishing a taxonomic revision of *Brachypelma* and this species will be accommodated in a new genus.

**Figure 26.**
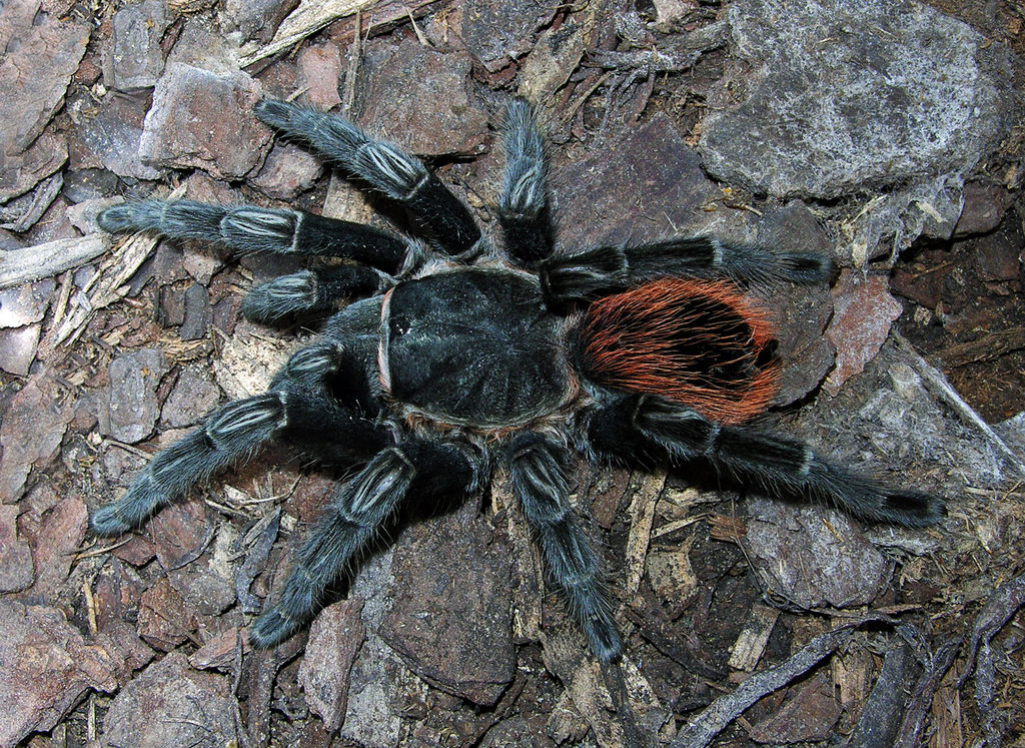
*Brachypelma epicureanum* (Chamberlin, 1925) female from Mérida, Yucatán, Mexico. Photo: Rick C. West.

**Figure 27.**
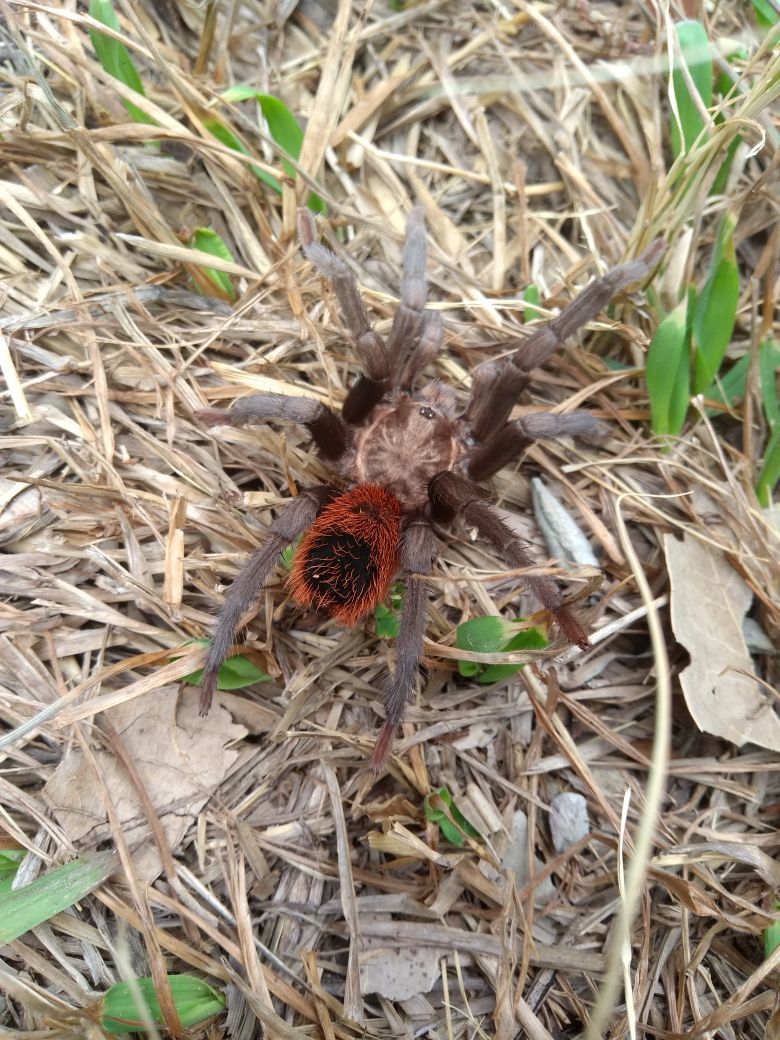
*Brachypelma epicureanum* (Chamberlin, 1925) male from Mexico. Photo: Rodrigo Orozco.

Figure(s) or Photo(s): Figs 26, 27

Region for assessment: Global

#### Geographic range

Biogeographic realm: Neotropical

Countries: Mexico

**Figure 28.**
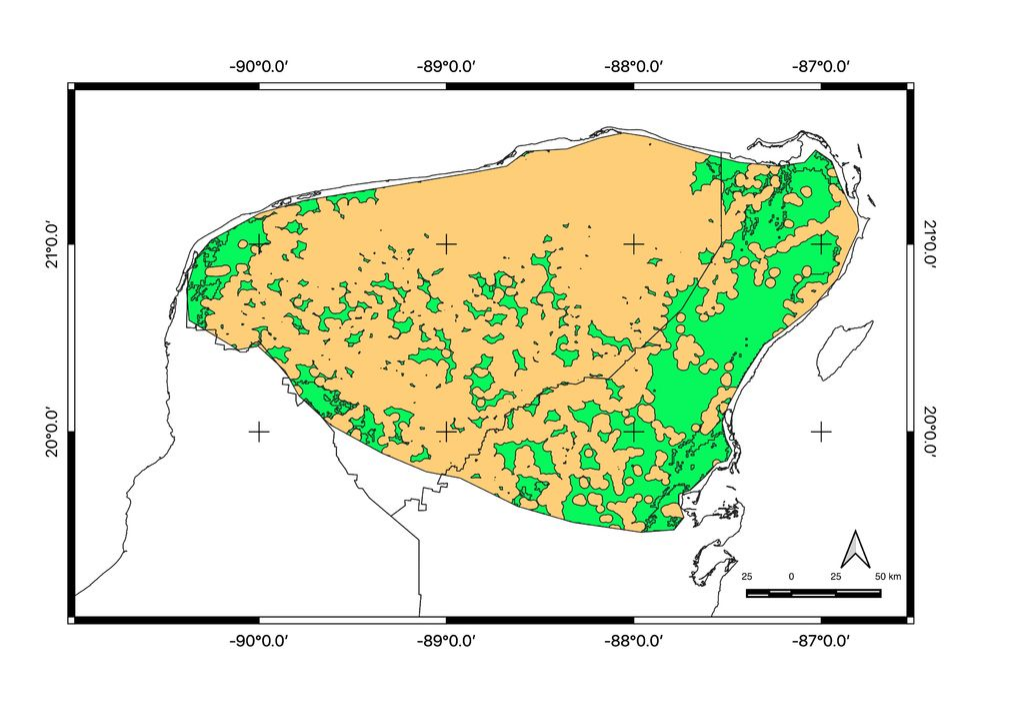
Habitat quality across the distribution of *Brachypelma epicureanum* (Chamberlin, 1925). Low quality habitat areas (LQH) in orange and high quality habitat areas (HQH) in green (for estimation details, see formula in Methods).

Map of records (image): Fig. 28

Map of records (Google Earth): Suppl. material 10

Basis of EOO and AOO: Species Distribution Model

Basis (narrative): A species distribution modelling has been performed to predict its potential range. See methods for details.

Min Elevation/Depth (m): 0

Max Elevation/Depth (m): 190

Range description: *Brachypelma epicureanum* is endemic to Mexico and occurs in Yucatán State and the north half of Quintana Roo State (Chamberlin 1925, Arisqueta-Chablé et al. 2009, CEC 2017, iNaturalist 2018, Hijmensen 2018, unpublished data)

#### New occurrences

#### Extent of occurrence

EOO (km2): 59225

Trend: Decline (inferred)

Justification for trend: Despite a lack of systematic research, increasingly intense urbanisation and touristic activities on the coastal area of Quintana Roo State have likely led to the extinction of some subpopulations in the region, causing a reduction in EOO. Many subpopulations have also recently been negatively affected by more frequent rising water (flooding) and hurricanes. The area where this species occurs is being used intensively for agriculture. Farmers regularly burn their corn fields to make the land fertile again (Hijmensen 2018), an act which can affect the subpopulations.

Causes ceased?: No

Causes understood?: Yes

Causes reversible?: No

Extreme fluctuations?: No

#### Area of occupancy

Trend: Decline (inferred)

Justification for trend: Despite a lack of systematic research, increasingly intense urbanisation and touristic activities on the coastal area of Quintana Roo State have likely led to the extinction of some subpopulations in the region, causing a reduction in AOO. Many subpopulations have also recently been negatively affected by more frequent rising water (flooding) and hurricanes. The area where this species occurs is being used intensively for agriculture. Farmers regularly burn their corn fields to make the land fertile again (Hijmensen 2018), an act which can affect the subpopulations.

Causes ceased?: No

Causes understood?: Yes

Causes reversible?: No

Extreme fluctuations?: No

AOO (km2): 57096

#### Locations

Number of locations: Unknown

Justification for number of locations: The number of locations is far above any thresholds.

Trend: Decline (inferred)

Justification for trend: Inferred from a decrease in the number of subpopulations.

Extreme fluctuations?: No

#### Population

Number of individuals: Unknown

Trend: Decline (inferred)

Justification for trend: Despite a lack of systematic research, increasingly intense urbanisation and touristic activities on the coastal area of Quintana Roo State have likely led to the extinction of some subpopulations in the region, causing a reduction in AOO, EOO and, consequently, population size. Harvesting adds to this reduction, since there is an unknown amount of trafficking of wild-caught animals. Many subpopulations have also recently been negatively affected by loss of quality of habitat due to more frequent rising water (flooding) and hurricanes. The area where this species occurs is being used intensively for agriculture. Farmers regularly burn their corn fields to make the land fertile again (Hijmensen 2018), an act which can affect the subpopulations.

Basis for decline: (c) a decline in area of occupancy, extent of occurrence and/or quality of habitat(d) actual or potential levels of exploitation

Causes ceased?: No

Causes understood?: Yes

Causes reversible?: No

Extreme fluctuations?: No

Population Information (Narrative): Despite a lack of systematic research, increasingly intense urbanisation and touristic activities on the coastal area of Quintana Roo State have likely led to the extinction of some subpopulations in the region, causing a reduction in AOO and, consequently, population size. Harvesting adds to this reduction, since there is an unknown amount of trafficking of wild-caught animals. Many subpopulations have also recently been negatively affected by more frequent rising water (flooding) and hurricanes. The area where this species occurs is being used intensively for agriculture. Farmers regularly burn their corn fields to make the land fertile again (Hijmensen 2018), an act which can affect the subpopulations.

#### Subpopulations

Number of subpopulations: Unknown

Trend: Decline (inferred)

Justification for trend: Despite a lack of systematic research, increasingly intense urbanisation and touristic activities on the coastal area of Quintana Roo State have likely led to the extinction of some subpopulations in the region. Many subpopulations have also recently been negatively affected by more frequent rising water (flooding) and hurricanes. The area, where this species occurs, is being used intensively for agriculture. Farmers regularly burn their corn fields to make the land fertile again (Hijmensen 2018), an act which can affect the subpopulations.

Extreme fluctuations?: No

Severe fragmentation?: Yes

Justification for fragmentation: Based on the analysis of the relative abundance of each species in low quality habitat (RA_LQH_) and the Species Distribution Model, 71.06% of the population should be in subpopulations that are non-viable and without the possibility of rescue effects due to fragmentation.

#### Habitat

System: Terrestrial

Habitat specialist: No

Habitat (narrative): *Brachypelma epicureanum* inhabits the Yucatán dry forest (Hijmensen 2018), which consists of lower thorn brush vegetation with an upper canopy of deciduous trees and some palms. The species also can adapt to some slightly modified habitats disturbed by human activities. The area, where this species occurs, is being used intensively for agriculture. Farmers regularly burn their corn fields to make the land fertile again (Hijmensen 2018), an act which can affect the subpopulations.

Trend in extent, area or quality?: Decline (inferred)

Justification for trend: The habitat of this species is inferred to be declining in area, extent and quality due to huge human disturbance (urbanisation and tourism), reducing much of the natural habitat. Many subpopulations have been negatively affected both by rising water (flooding) and hurricanes. The area, where this species occurs, is being used intensively for agriculture. Farmers regularly burn their corn fields to make the land fertile again (Hijmensen 2018), an act which can affect the subpopulations.

**Figure 29.**
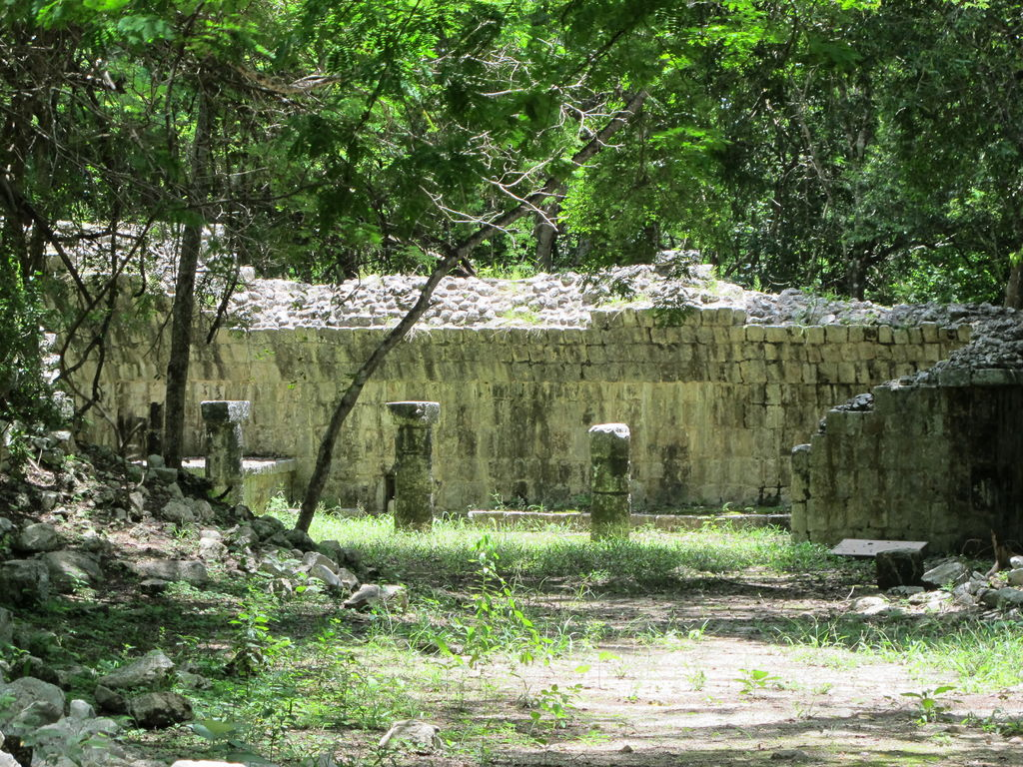
*Brachypelma epicureanum* habitat with archaeological ruins, Mexico. Photo: Jorge Mendoza.

Figure(s) or Photo(s): Fig. 29

##### Habitat

Habitat importance: Major Importance

Habitats: 1.5. Forest - Subtropical/Tropical Dry

##### Habitat

Habitat importance: Marginal

Habitats: 14.2. Artificial/Terrestrial - Pastureland14.3. Artificial/Terrestrial - Plantations14.6. Artificial/Terrestrial - Subtropical/Tropical Heavily Degraded Former Forest

#### Habitat

Habitat importance: Major Importance

Habitats: 1.5. Forest - Subtropical/Tropical Dry

#### Habitat

Habitat importance: Marginal

Habitats: 14.2. Artificial/Terrestrial - Pastureland14.3. Artificial/Terrestrial - Plantations14.6. Artificial/Terrestrial - Subtropical/Tropical Heavily Degraded Former Forest

#### Ecology

Size: 70 mm (female); 60 mm (male).

Generation length (yr): 7

Dependency of single sp?: No

Ecology and traits (narrative): *Brachypelma epicureanum* is a fossorial species that constructs or modifies burrows, often clearly defined obligate burrow-like retreat under debris, such as large rocks and tree roots in subtropical dry forest. The burrows typically slope at a 45-degree angle for about 30 centimetres, ending in a chamber (Hijmensen 2018). Burrows will typically have a layer of silk around the entrance and can be sealed with a further thin layer of silk across the diameter during daylight that may deter predators (e.g. ants, wasps etc.) and/or help maintain humidity inside the retreat. Studies on this species in its natural habitat have identified that, unlike the superficially similar *B. vagans*, *B. epicureanum* can be uncommon, which can indicate that it is a demographically rare species (Arisqueta-Chablé et al. 2009). These spiders are nocturnal predators that wait near the entrance of their refuge from dusk and into the night to feed primarily on ground-dwelling arthropods (insects, other arachnids and some myriapods) or even small vertebrates. The mating season occurs towards the end of the year (August to January) when mature males wander in the open to search for females. The peak has been recorded as being around October, during the rainy season, as most adult males were collected in the field during this period (Arisqueta-Chablé et al. 2009). The males are likely most active at night, cooler daylight hours and throughout overcast days. Adult females typically moult once per year, just prior to the onset of the annual male emergence. Females will produce cocoons (large silken egg sacs) during the drier winter months with young emerging about two months later, with most young dispersing in the late spring or early summer, just before the onset of the early summer rains.

#### Threats

Justification for threats: Despite a lack of systematic research, increasingly intense urbanisation and consequently deforestation on the coastal area of Quintana Roo State in the last years (Global Forest Watch 2019) have likely led to the extinction of some subpopulations in the region, causing a reduction in EOO, AOO and consequently population size. The strong touristic activity in all Yucatán Peninsula is also negatively affecting subpopulations of *B. epicureanum*. Harvesting adds to this reduction, since there is an unknown amount of trafficking of wild-caught animals. Roads facilitate the access of smugglers to wild populations and are also a source *per se* of habitat degradation. Many subpopulations have also recently been negatively affected both by natural phenomena, such as rising water (flooding) and hurricanes or by human interferences, such as fire on crops (Hijmensen 2018).

##### Threats

Threat type: Ongoing

Threats: 1.1. Residential & commercial development - Housing & urban areas1.3. Residential & commercial development - Tourism & recreation areas2.1.2. Agriculture & aquaculture - Annual & perennial non-timber crops - Small-holder farming2.1.3. Agriculture & aquaculture - Annual & perennial non-timber crops - Agro-industry farming4.1. Transportation & service corridors - Roads & railroads5.1.1. Biological resource use - Hunting & trapping terrestrial animals - Intentional use (species is the target)7.1.1. Natural system modifications - Fire & fire suppression - Increase in fire frequency/intensity11.4. Climate change & severe weather - Storms & flooding

#### Threats

Threat type: Ongoing

Threats: 1.1. Residential & commercial development - Housing & urban areas1.3. Residential & commercial development - Tourism & recreation areas2.1.2. Agriculture & aquaculture - Annual & perennial non-timber crops - Small-holder farming2.1.3. Agriculture & aquaculture - Annual & perennial non-timber crops - Agro-industry farming4.1. Transportation & service corridors - Roads & railroads5.1.1. Biological resource use - Hunting & trapping terrestrial animals - Intentional use (species is the target)7.1.1. Natural system modifications - Fire & fire suppression - Increase in fire frequency/intensity11.4. Climate change & severe weather - Storms & flooding

#### Conservation

Justification for conservation actions: This species occurs in the Biosphere Reserve of Celestun, National Park of Ría Lagartos, National Park San Felipe and Ecological Reserves of Cuxtal and Sian Kaan, so some subpopulations are under ecological protection. The northern section of the species distribution, in particular, is the one that presents greater risk of survival as it is affected by increasing frequency of flooding and hurricanes, deserving special attention regarding conservation actions. As *B. epicureanum* occurs in an area with intense touristic activities (the type locality, for example, is the very famous archaeological site of Chichén Itza), management and species recovery plans are needed. It would be interesting to take advantage of the fact that the species occurs in a highly touristic area to promote educational activities to the visitors in order to help *in-situ* conservation and to curb trafficking as well. The species can also be used as a flagship for motivating conservational efforts in Yucatán peninsula. This species is currently listed on CITES Appendix II, along with all other *Brachypelma* species (CITES 2019). *Brachypelma epicureanum* is being reared in captivity in some Mexican Units for Management (UMAs) and sold legally. Although not common in the trade, this species is traded in both the Mexican and international pet market. Thus, it is necessary to develop better enforcement actions to combat illegal pet trade, as well as establish tax advantages for legal dealers in order to make their prices more competitive with the ones in the black market. Currently, this species is being reared in captivity in a UMA that also promotes educational and awareness activities regarding tarantula spiders aimed at students and the general public.

##### Conservation actions

Conservation action type: In Place

Conservation actions: 1.1. Land/water protection - Site/area protection3.4.1. Species management - Ex-situ conservation - Captive breeding/artificial propagation5.1.1. Law & policy - Legislation - International level4.3. Education & awareness - Awareness & communications

##### Conservation actions

Conservation action type: Needed

Conservation actions: 1.1. Land/water protection - Site/area protection1.2. Land/water protection - Resource & habitat protection2.1. Land/water management - Site/area management3.2. Species management - Species recovery5.4.1. Law & policy - Compliance and enforcement - International level5.4.2. Law & policy - Compliance and enforcement - National level5.4.3. Law & policy - Compliance and enforcement - Sub-national level6.4. Livelihood, economic & other incentives - Conservation payments4.3. Education & awareness - Awareness & communications

#### Conservation actions

Conservation action type: In Place

Conservation actions: 1.1. Land/water protection - Site/area protection3.4.1. Species management - Ex-situ conservation - Captive breeding/artificial propagation5.1.1. Law & policy - Legislation - International level4.3. Education & awareness - Awareness & communications

#### Conservation actions

Conservation action type: Needed

Conservation actions: 1.1. Land/water protection - Site/area protection1.2. Land/water protection - Resource & habitat protection2.1. Land/water management - Site/area management3.2. Species management - Species recovery5.4.1. Law & policy - Compliance and enforcement - International level5.4.2. Law & policy - Compliance and enforcement - National level5.4.3. Law & policy - Compliance and enforcement - Sub-national level6.4. Livelihood, economic & other incentives - Conservation payments4.3. Education & awareness - Awareness & communications

#### Other

Justification for use and trade: *Brachypelma epicureanum* is being reared in captivity in some Mexican Units for Management (UMAs) and sold legally. This species is currently listed on CITES Appendix II and thus its international trade is regulated by an international agreement (CITES 2019). *Brachypelma epicureanum* is not common in the pet trade within Mexico and only in relatively small numbers in the international pet trade. Compared with other *Brachypelma* species, the trade of *B. epicureanum* is not intense, consisting of just 52 declared specimens traded internationally during 2006–2016 for commercial purposes (Cooper et al. 2019).

##### Use and trade

Use type: International

Use and trade: 13. Pets/display animals, horticulture16. Establishing ex-situ production *

##### Ecosystem services

Ecosystem service type: Less important

##### Research needed

Research needed: 1.2. Research - Population size, distribution & trends1.5. Research - Threats2.1. Conservation Planning - Species Action/Recovery Plan3.1. Monitoring - Population trends3.4. Monitoring - Habitat trends

Justification for research needed: Due to the occurrence in an area that is experiencing a high level of human activity, such as urbanisation and tourism, it is necessary to prioritise and support research on the impact of changes in land use and habitat degradation and in the population viability of *B. epicureanum*. This will help in conservation decisions, especially for subpopulations in the northern section of the species distribution which are subject to the most extreme risks to survival through increasing frequency of deleterious natural processes (hurricanes, flooding etc). These subpopulations and area, in particular, need to be carefully monitored.

#### Use and trade

Use type: International

Use and trade: 13. Pets/display animals, horticulture16. Establishing ex-situ production *

#### Ecosystem services

Ecosystem service type: Less important

#### Research needed

Research needed: 1.2. Research - Population size, distribution & trends1.5. Research - Threats2.1. Conservation Planning - Species Action/Recovery Plan3.1. Monitoring - Population trends3.4. Monitoring - Habitat trends

Justification for research needed: Due to the occurrence in an area that is experiencing a high level of human activity, such as urbanisation and tourism, it is necessary to prioritise and support research on the impact of changes in land use and habitat degradation and in the population viability of *B. epicureanum*. This will help in conservation decisions, especially for subpopulations in the northern section of the species distribution which are subject to the most extreme risks to survival through increasing frequency of deleterious natural processes (hurricanes, flooding etc). These subpopulations and area, in particular, need to be carefully monitored.

#### Viability analysis

### Brachypelma hamorii

#### Species information

Scientific name: Brachypelma hamorii

Species authority: Tesmoingst, Cleton & Verdez, 1997

Common names: Mexican orangeknee, tarántula mexicana de rodillas anaranjadas, mygale mexicaine à genoux orange.

Kingdom: Animalia

Phylum: Arthropoda

Class: Arachnida

Order: Araneae

Family: Theraphosidae

**Figure 30.**
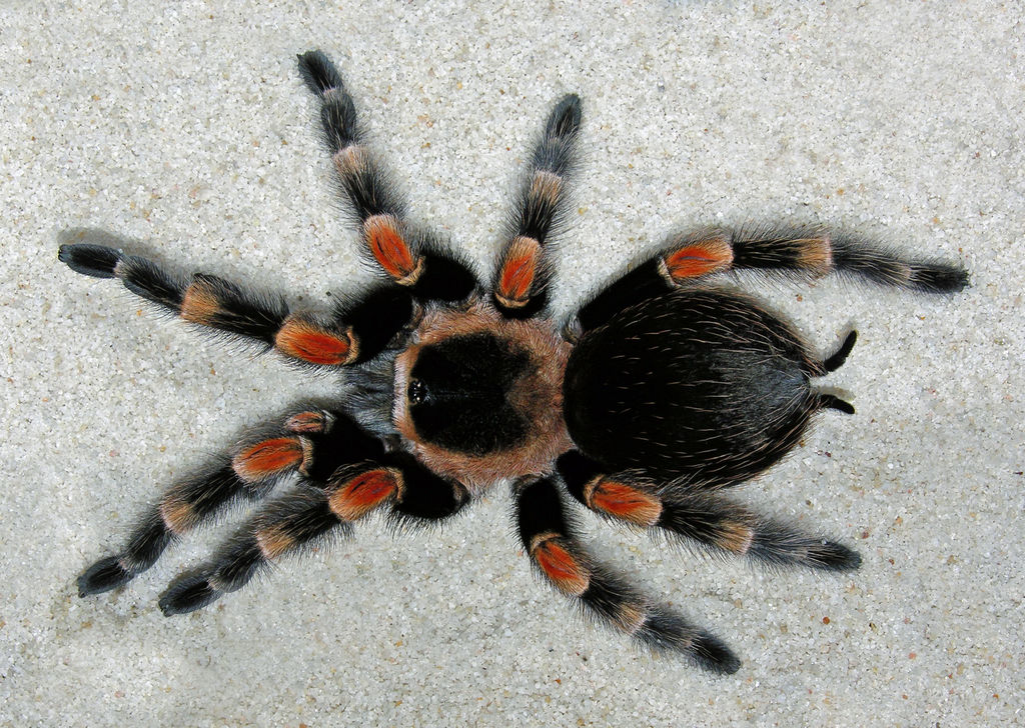
*Brachypelma hamorii* Tesmoignt, Cleton & Verdez, 1997 female near Manzanillo, Colima state, Mexico. Photo: Rick C. West.

**Figure 31.**
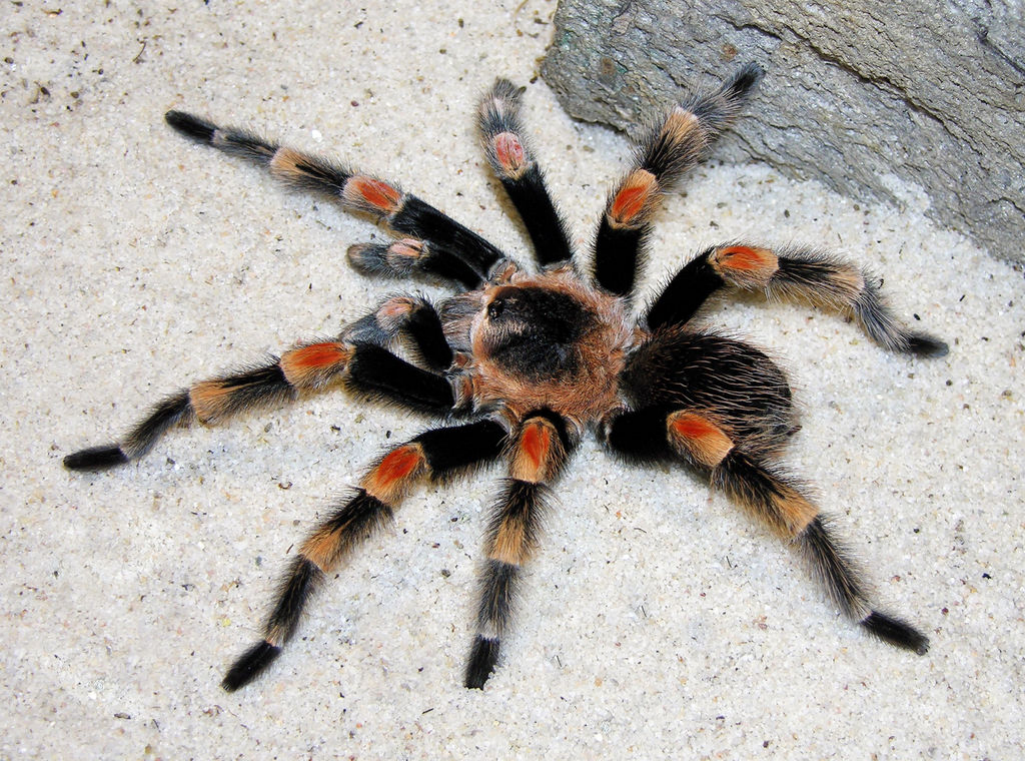
*Brachypelma hamorii* Tesmoignt, Cleton & Verdez, 1997 male from Colima state, Mexico. Photo: Rick C. West.

Figure(s) or Photo(s): Figs 30, 31

Region for assessment: Global

#### Geographic range

Biogeographic realm: Neotropical

Countries: Mexico

**Figure 32.**
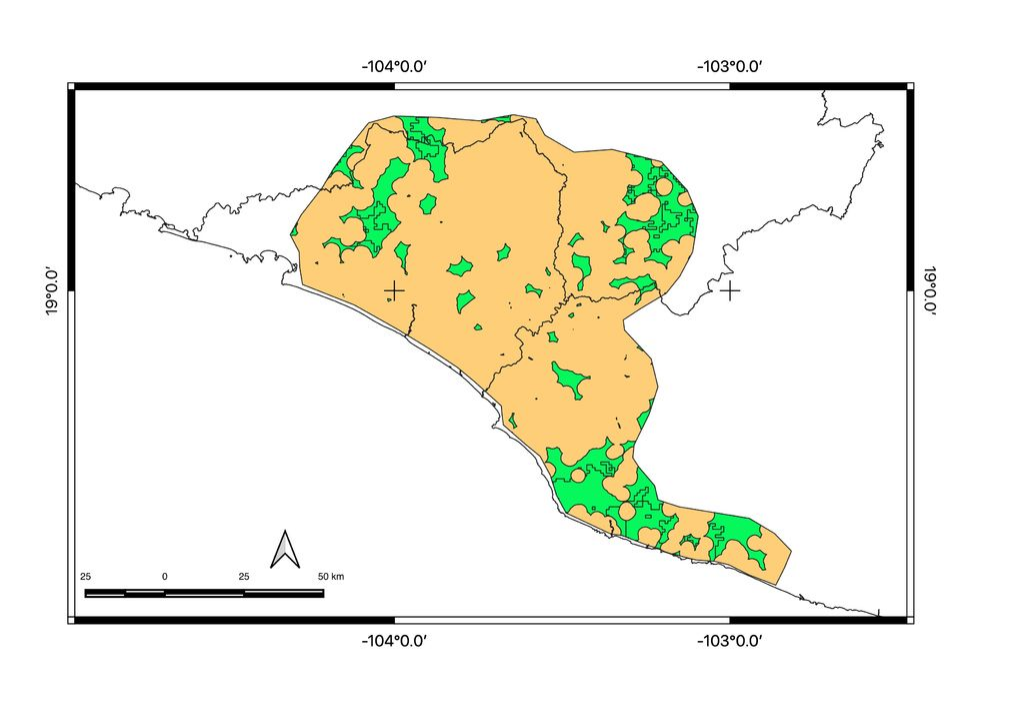
Habitat quality across the distribution of *Brachypelma hamorii* Tesmoingt, Cleton & Verdez, 1997. Low quality habitat areas (LQH) in orange and high quality habitat areas (HQH) in green (for estimation details, see formula in Methods).

Map of records (image): Fig. 32

Map of records (Google Earth): Suppl. material 11

Basis of EOO and AOO: Species Distribution Model

Basis (narrative): A species distribution modelling has been performed to predict its potential range. See methods for details.

Min Elevation/Depth (m): 0

Max Elevation/Depth (m): 2110

Range description: *Brachypelma hamorii* is endemic to Mexico and occurs across much of the small Colima State and in the north-western coast of Michoacán State, plus into fringes of southern Jalisco State (Tesmoingt et al. 1997, Mendoza and Francke 2017, iNaturalist 2018, unpublished data). *Brachypelma hamorii* occurs in the lowland coastal zone, but ranges slightly further inland than neighbouring species. It is sympatric with *B. baumgarteni* in the north-western part of Michoacán State and with subpopulations of *B. klaasi* in the north-western limits of Colima State.

#### New occurrences

#### Extent of occurrence

EOO (km2): 13030

Trend: Decline (inferred)

Justification for trend: A decrease in EOO is inferred since there is habitat loss due to human activities, such as urbanisation, agriculture and roads.

Causes ceased?: No

Causes understood?: Yes

Causes reversible?: No

Extreme fluctuations?: No

#### Area of occupancy

Trend: Decline (inferred)

Justification for trend: A decrease in EOO is inferred since there is habitat loss due to human activities, such as urbanisation, agriculture and roads.

Causes ceased?: No

Causes understood?: Yes

Causes reversible?: No

Extreme fluctuations?: No

AOO (km2): 8452

#### Locations

Number of locations: Unknown

Justification for number of locations: The number of locations far exceeds any thresholds.

Trend: Decline (inferred)

Justification for trend: Inferred from the decrease in the number of subpopulations.

Extreme fluctuations?: No

#### Population

Number of individuals: Unknown

Trend: Decline (inferred)

Justification for trend: A decline in population size is inferred since there is a decrease in AOO and quality of habitat due to human activity, such as urbanisation, agricultural activities and roads. It has also been intensively collected for the pet trade, likely leading to the extinction of some subpopulations.

Basis for decline: (c) a decline in area of occupancy, extent of occurrence and/or quality of habitat(d) actual or potential levels of exploitation

Causes ceased?: No

Causes understood?: Yes

Causes reversible?: No

Extreme fluctuations?: No

Population Information (Narrative): Habitat loss due to human activity (urbanisation, agricultural activities) is leading to decline in the population throughout Colima State in Mexico due to major recent expansion of human development, especially in low-lying coastal areas. The natural habitat of *B. hamorii* is subdivided by roads such as Federal Highway 200, an important road that connects several large cities along the Pacific coast of Mexico and which, combined with other roads, appears to be both a significant set of barriers to dispersal and a cause of mortality for males during the mating season. Adding to this, the construction of roads also facilitates the access of smugglers to some areas, leading to a declining of wild populations. Additionally, regarding Colima State, a harvesting pressure in populations of this area has been recorded.

#### Subpopulations

Number of subpopulations: Unknown

Trend: Decline (inferred)

Justification for trend: A decrease in the number of subpopulations is inferred since there is habitat loss due to human activities, such as urbanisation, agriculture and roads. Harvesting of wild animals is also leading to the disappearance of some subpopulations.

Extreme fluctuations?: No

Severe fragmentation?: Yes

Justification for fragmentation: Based on the analysis of the relative abundance of each species in low quality habitat (RA_LQH_) and the Species Distribution Model, 86.18% of the population should be in subpopulations that are non-viable and without the possibility of rescue effects due to fragmentation.

#### Habitat

System: Terrestrial

Habitat specialist: Yes

Habitat (narrative): *Brachypelma hamorii* inhabits the subtropical dry forest in the foothills of the Sierra Madre del Sur mountains in Mexico, from the lowlands near the Pacific coast into slightly higher elevations further inland. This region is mostly covered by thorn and deciduous secondary forests (Mendoza and Francke 2017). They appear to prefer undisturbed areas shaded by trees or bushes and can favour areas near to seasonal watercourses.

Trend in extent, area or quality?: Decline (inferred)

Justification for trend: The habitat of this species is inferred to be declining in area, extent and quality due to deforestation caused by urbanisation and agricultural activities. The construction and expansion of roads is also destroying habitat and for remaining subpopulations adjacent to them, the major pacific transit routes in this region provide both a barrier to dispersal and cause of mortality, as males in particular appear to often be run over by traffic.

**Figure 33.**
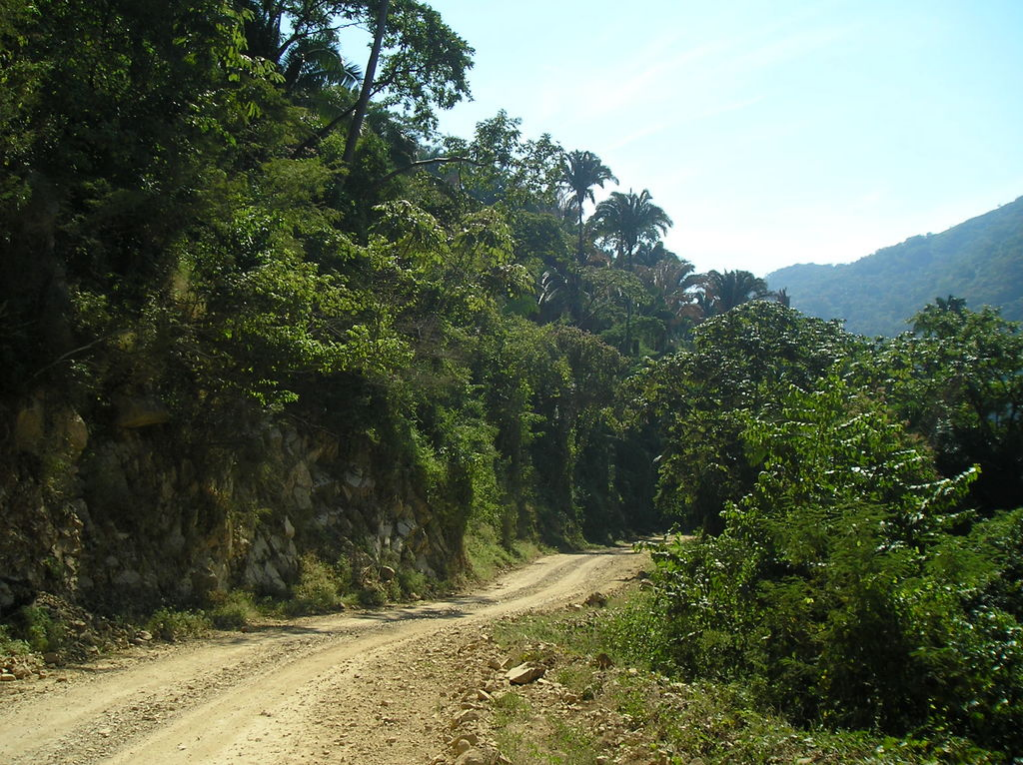
*Brachypelma hamorii* habitat, subtropical dry forest, near Manzanillo, Colima state, Mexico. Photo: Stuart J. Longhorn.

Figure(s) or Photo(s): Fig. 33

##### Habitat

Habitat importance: Major Importance

Habitats: 1.5. Forest - Subtropical/Tropical Dry

#### Habitat

Habitat importance: Major Importance

Habitats: 1.5. Forest - Subtropical/Tropical Dry

#### Ecology

Size: 70 mm (female); 60 mm (male).

Generation length (yr): 7

Dependency of single sp?: No

Ecology and traits (narrative): *Brachypelma hamorii* is a fossorial species that modifies previously excavated burrows or can excavate their own unaided, often only making minor alteration to natural small cavities under debris such as fallen logs, large rocks and large tree roots amongst thorny brush or tall grass thickets (after Mendoza and Francke 2017) in subtropical dry forest. The burrows have no traces of silk around the entrance (Mendoza and Francke 2017), giving no clear indication that there is a spider inside. These spiders are nocturnal predators that wait near the entrance of their refuge from dusk and into the night to feed primarily on ground-dwelling arthropods (insects, other arachnids and some myriapods) or even small vertebrates. The mating season occurs during the last part of the rainy and first part of dry seasons (August to January) when mature males wander in the open to search for females. The males are likely most active at night, cooler daylight hours and throughout overcast days. Adult females typically moult once per year, just prior to the onset of the annual male emergence. Females will produce cocoons (large silken egg sacs) during the drier winter months with young emerging about two months later, with most young dispersing in the late spring or summer, just before the onset of the early summer rains.

#### Threats

Justification for threats: Habitat loss due to human activities, such as urbanisation and agriculture, threaten the species. Many urban areas are rapidly expanding, often related to extensive agriculture through both small-holders and agro-industry, especially fruit growing (particularly lime, avocado, mangoes and melon), plus maize, cocoa, sugar-cane, cotton etc. These are united by a network of roads and rail, linking them to other cities in Mexico and export via the international port of Manzanillo. This species, along with the others of the red leg complex, are more fragile and do not adapt well or quickly to alterations on the environment caused by human activities (Turner et al. 2018). Roads can be a significant threat as both a barrier to dispersal and a cause of mortality, since males are frequently found run over during the mating season. Adding to this, roads facilitate the access of smugglers to wild populations and also are a source *per se* of habitat degradation. In addition, red leg species are more popular as pets than the red abdomen ones. Thus, harvesting pressure on several subpopulations of *B. hamorii* around Colima can also be considered as a threat to the species, as illegal collection for the pet trade appears to be ongoing.

##### Threats

Threat type: Ongoing

Threats: 1.1. Residential & commercial development - Housing & urban areas2.1.2. Agriculture & aquaculture - Annual & perennial non-timber crops - Small-holder farming2.1.3. Agriculture & aquaculture - Annual & perennial non-timber crops - Agro-industry farming4.1. Transportation & service corridors - Roads & railroads5.1.1. Biological resource use - Hunting & trapping terrestrial animals - Intentional use (species is the target)

#### Threats

Threat type: Ongoing

Threats: 1.1. Residential & commercial development - Housing & urban areas2.1.2. Agriculture & aquaculture - Annual & perennial non-timber crops - Small-holder farming2.1.3. Agriculture & aquaculture - Annual & perennial non-timber crops - Agro-industry farming4.1. Transportation & service corridors - Roads & railroads5.1.1. Biological resource use - Hunting & trapping terrestrial animals - Intentional use (species is the target)

#### Conservation

Justification for conservation actions: Although the real occurrence of this species remains to be evaluated, some protected areas such as Volcán Nevado de Colima National Park and Manantlán Biosphere Reserve are inside the range of the species and may be suitable for developing conservation initiatives. In order to avoid international trade incompatible with its survival, this species is currently listed on CITES Appendix II, along with all other *Brachypelma* species (CITES 2019). *Brachypelma hamorii* is being reared in captivity in Mexican Units for Management (UMAs) and sold legally. However, an illegal pet trade is likely an ongoing concern since adults of unknown origin have frequently been traded in both Mexican and international markets. A single subpopulation in Colima seems to provide most of the specimens traded, impacted by a high harvesting pressure, thus it requires protection and efforts to repopulate it. Important conservation actions include developing new initiatives to protect the natural habitat of *B. hamorii*, creating management plans and conducting systematic monitoring to provide information about the recovery of subpopulations, since there are no conservation units in the occurrence area of the species. As this region of the Pacific coast is one of the most populous areas of Mexico with several different species of *Brachypelma*, it should therefore be a priority area regarding the establishment of new conservation areas. It is also necessary to improve enforcement actions to curb trafficking, as well as establishing tax advantages for legal dealers in order to make their prices more competitive than others in the black market. Currently, this species is being reared in captivity in a Mexican Unit for Management (UMA) that also promotes educational and awareness activities regarding tarantula spiders aimed at students and the general public.

##### Conservation actions

Conservation action type: In Place

Conservation actions: 1.1. Land/water protection - Site/area protection3.4.1. Species management - Ex-situ conservation - Captive breeding/artificial propagation5.1.1. Law & policy - Legislation - International level4.3. Education & awareness - Awareness & communications

##### Conservation actions

Conservation action type: Needed

Conservation actions: 1.1. Land/water protection - Site/area protection3.2. Species management - Species recovery3.3.1. Species management - Species re-introduction - Reintroduction5.4.1. Law & policy - Compliance and enforcement - International level5.4.2. Law & policy - Compliance and enforcement - National level5.4.3. Law & policy - Compliance and enforcement - Sub-national level6.4. Livelihood, economic & other incentives - Conservation payments

#### Conservation actions

Conservation action type: In Place

Conservation actions: 1.1. Land/water protection - Site/area protection3.4.1. Species management - Ex-situ conservation - Captive breeding/artificial propagation5.1.1. Law & policy - Legislation - International level4.3. Education & awareness - Awareness & communications

#### Conservation actions

Conservation action type: Needed

Conservation actions: 1.1. Land/water protection - Site/area protection3.2. Species management - Species recovery3.3.1. Species management - Species re-introduction - Reintroduction5.4.1. Law & policy - Compliance and enforcement - International level5.4.2. Law & policy - Compliance and enforcement - National level5.4.3. Law & policy - Compliance and enforcement - Sub-national level6.4. Livelihood, economic & other incentives - Conservation payments

#### Other

Justification for use and trade: *Brachypelma hamorii* is being reared in captivity in Mexican Unit for Management (UMAs) and sold legally. This species is currently listed on CITES Appendix II and thus is protected by an international agreement (CITES 2019). No legal trade in specimens of *B. hamorii* was recorded in the UNEP-WCMC CITES Trade Database during 2006–2016 (Cooper et al. 2019). However, this should be analysed carefully since the taxonomic review of the genus was not published until 2017 (Mendoza and Francke 2017). As it is known now that the specimens from Colima traded as *B. smithi* were actually specimens of *B. hamorii*, the participation of *B. hamorii* in international trade up to 2017 needs to be reviewed. Currently, an illegal pet trade is being carried out since the presence of adults on the market was detected, which captive breeding cannot account for. It is known that *B. hamorii* specimens are also traded in the black market, misidentified as *B. smithi*.

##### Use and trade

Use type: International

Use and trade: 13. Pets/display animals, horticulture16. Establishing ex-situ production *

##### Ecosystem services

Ecosystem service type: Less important

##### Research needed

Research needed: 1.2. Research - Population size, distribution & trends1.4. Research - Harvest, use & livelihoods1.5. Research - Threats2.1. Conservation Planning - Species Action/Recovery Plan2.2. Conservation Planning - Area-based Management Plan2.3. Conservation Planning - Harvest & Trade Management Plan

Justification for research needed: It is necessary to prioritise and support research on the population trends and distribution, as well as on the impact of land use and habitat degradation, mainly around the Colima area, where the most threatened subpopulations are found. As said, the Pacific coast is one of the most human-disturbed areas of Mexico and encompasses several different species of *Brachypelma*. Therefore, it is a priority area regarding conservation planning and actions for the genus. Despite *B. hamorii* not being officially on record for recent trade (although see *B. smithi*), it is necessary to research its national and international use, demand for and trade, since illegal commerce is being carried out.

#### Use and trade

Use type: International

Use and trade: 13. Pets/display animals, horticulture16. Establishing ex-situ production *

#### Ecosystem services

Ecosystem service type: Less important

#### Research needed

Research needed: 1.2. Research - Population size, distribution & trends1.4. Research - Harvest, use & livelihoods1.5. Research - Threats2.1. Conservation Planning - Species Action/Recovery Plan2.2. Conservation Planning - Area-based Management Plan2.3. Conservation Planning - Harvest & Trade Management Plan

Justification for research needed: It is necessary to prioritise and support research on the population trends and distribution, as well as on the impact of land use and habitat degradation, mainly around the Colima area, where the most threatened subpopulations are found. As said, the Pacific coast is one of the most human-disturbed areas of Mexico and encompasses several different species of *Brachypelma*. Therefore, it is a priority area regarding conservation planning and actions for the genus. Despite *B. hamorii* not being officially on record for recent trade (although see *B. smithi*), it is necessary to research its national and international use, demand for and trade, since illegal commerce is being carried out.

#### Viability analysis

### Brachypelma kahlenbergi

#### Species information

Scientific name: Brachypelma kahlenbergi

Species authority: Rudloff, 2008

Common names: New Mexican tarantula, nueva tarántula mexicana, nouvelle mygale du Mexique.

Kingdom: Animalia

Phylum: Arthropoda

Class: Arachnida

Order: Araneae

Family: Theraphosidae

Taxonomic notes: Mendonza and Francke (in press) are publishing a taxonomic revision of *Brachypelma* and this species will be accommodated in a new genus.

**Figure 34.**
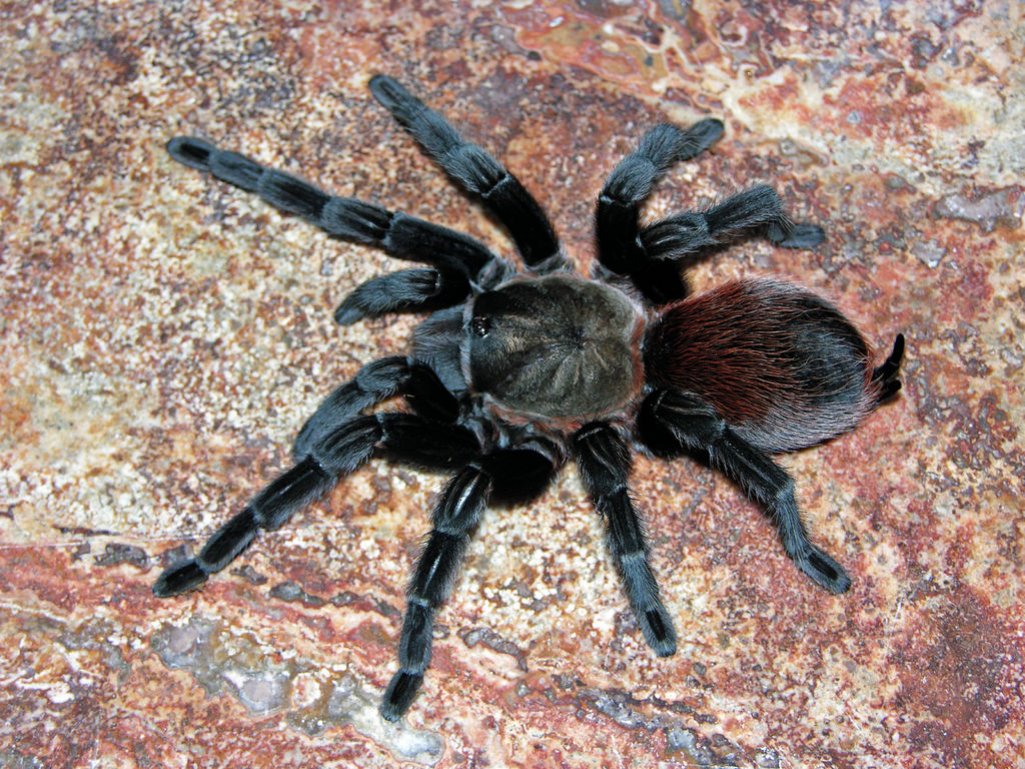
*Brachypelma kahlenbergi* Rudloff, 2008 female near Veracruz, Veracruz state, Mexico. Photo: Rick C. West.

**Figure 35.**
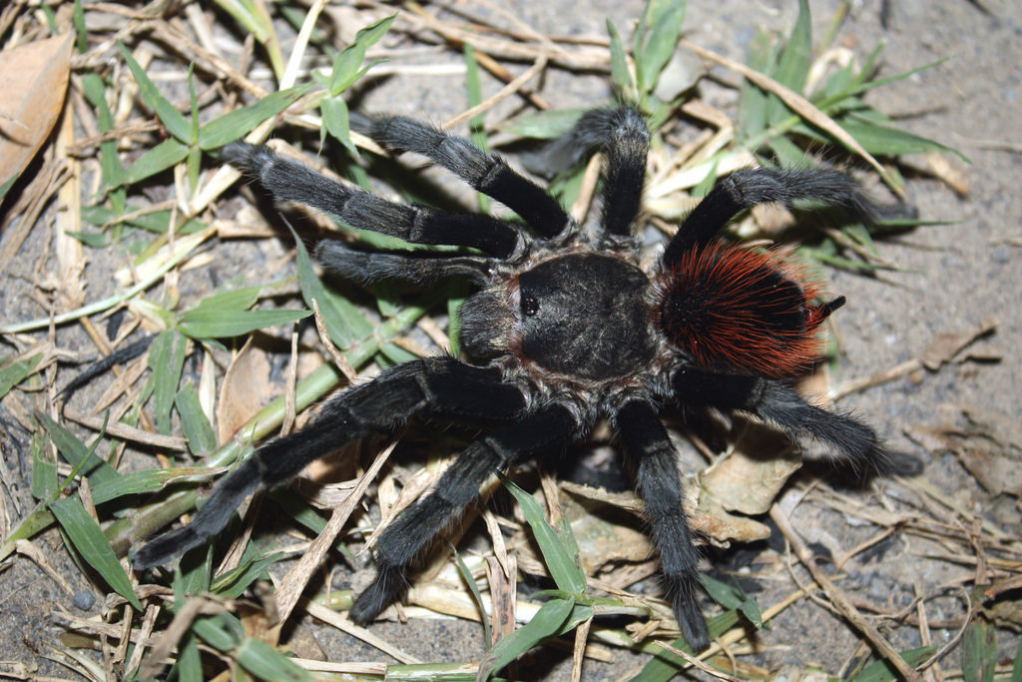
*Brachypelma kahlenbergi* Rudloff, 2008 male from near Veracruz, Veracruz state, Mexico. Photo: Stuart J. Longhorn.

Figure(s) or Photo(s): Figs 34, 35

Region for assessment: Global

#### Geographic range

Biogeographic realm: Neotropical

Countries: Mexico

**Figure 36.**
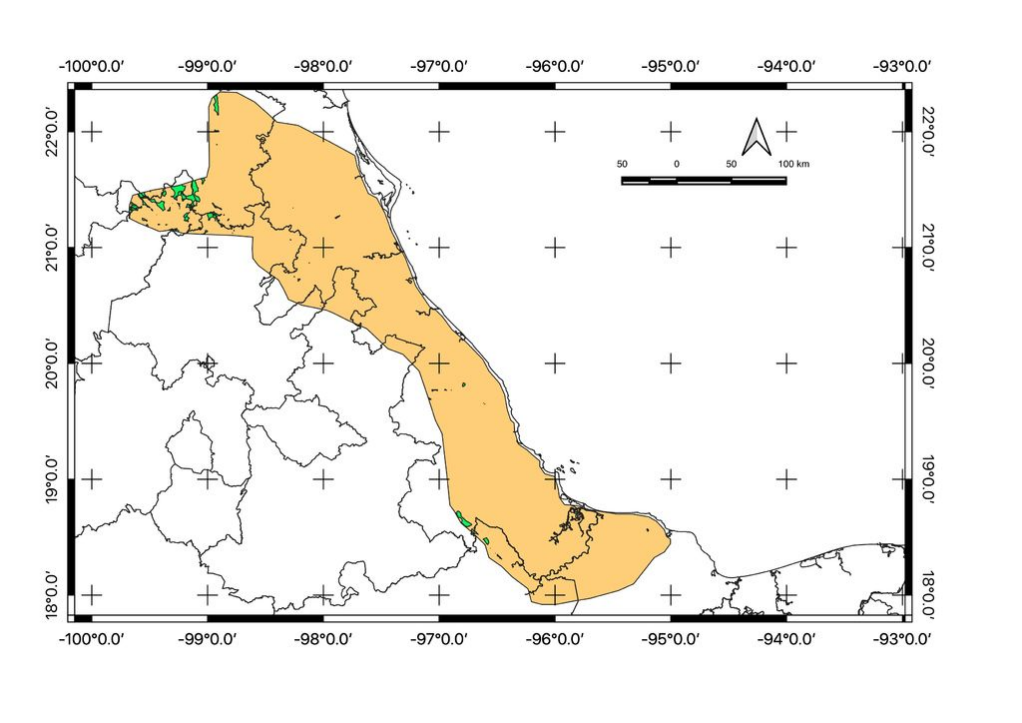
Habitat quality across the distribution of *Brachypelma kahlenberghi* Rudloff, 2008. Low quality habitat areas (LQH) in orange and high quality habitat areas (HQH) in green (for estimation details, see formula in Methods).

Map of records (image): Fig. 36

Map of records (Google Earth): Suppl. material 12

Basis of EOO and AOO: Species Distribution Model

Basis (narrative): A species distribution modelling has been performed to predict its potential range. See methods for details.

Min Elevation/Depth (m): 0

Max Elevation/Depth (m): 2600

Range description: *Brachypelma kahlenbergi* is endemic to Mexico and occurs east of the Sierra Madre Oriental in Veracruz State (Rudloff 2008, CEC 2017) and ranges into eastern San Luis Potosi, Querétaro and Hidalgo States and north-eastern Puebla and Oaxaca States (iNaturalist 2018, unpublished data)

#### New occurrences

#### Extent of occurrence

EOO (km2): 89494

Trend: Decline (inferred)

Justification for trend: A decline in EOO is inferred from habitat loss due to deforestation for urbanisation, agriculture and roads. Increasing frequency of deleterious natural disaster episodes, such as hurricanes within its range, is also a concern.

Causes ceased?: No

Causes understood?: Yes

Causes reversible?: No

Extreme fluctuations?: No

#### Area of occupancy

Trend: Decline (inferred)

Justification for trend: A decline in AOO is inferred from habitat loss due to deforestation for urbanisation, agriculture and roads. Increasing frequency of deleterious natural disaster episodes, such as hurricanes within its range, is also a concern.

Causes ceased?: No

Causes understood?: Yes

Causes reversible?: No

Extreme fluctuations?: No

AOO (km2): 51036

#### Locations

Number of locations: Unknown

Justification for number of locations: The number of locations far exceeds any thresholds.

Trend: Decline (inferred)

Justification for trend: Inferred from decline in number of subpopulations.

Extreme fluctuations?: No

#### Population

Number of individuals: Unknown

Trend: Decline (inferred)

Justification for trend: A decreasing number of individuals is inferred due to a decrease in AOO, especially due to deforestation (Global Forest Watch 2019) since the area where *B. kahlenbergi* can be found, mainly Veracruz State, is one of the most populous in Mexico, with a significant use for agriculture and cattle raising (SEMARNAT 2012). Adding to this, there is an increase of natural disaster episodes, such as hurricanes and tropical storms and the depletion of many subpopulations by smugglers to meet the illegal pet trade.

Basis for decline: (c) a decline in area of occupancy, extent of occurrence and/or quality of habitat(d) actual or potential levels of exploitation

Causes ceased?: No

Causes understood?: Yes

Causes reversible?: No

Extreme fluctuations?: No

Population Information (Narrative): The population size of *B. kahlenbergi* is unknown and there is no systematic research about population trends. However, the area where *B. kahlenbergi* can be found, mainly Veracruz State, is one of the most populous in Mexico, with a significant use for agriculture and cattle raising (SEMARNAT 2012), which can affect subpopulations of this species. It is known that hurricanes and tropical storms, common along coastal Veracruz State (SEMARNAT 2008), have depleted some subpopulations. Adding to this, overharvesting to meet the illegal pet trade is also affecting subpopulations of *B. kahlenbergi*.

#### Subpopulations

Number of subpopulations: Unknown

Trend: Decline (inferred)

Justification for trend: Decreasing number of subpopulations is inferred due to habitat loss.

Extreme fluctuations?: No

Severe fragmentation?: Yes

Justification for fragmentation: Based on the analysis of the relative abundance of each species in low quality habitat (RA_LQH_) and the Species Distribution Model, 99.45% of the species lives in low quality and fragmented habitat.

#### Habitat

System: Terrestrial

Habitat specialist: No

Habitat (narrative): *Brachypelma kahlenbergi* inhabits tropical and subtropical moist broadleaf forests of the Atlantic lowlands. The species can be found from low altitude, close to sea level, to higher elevations inland. They appear to be relatively tolerant of human-disturbance and can adapt to more open areas around livestock pasture and areas of non-intensive crops (without intense use of chemical pesticides).

Trend in extent, area or quality?: Decline (inferred)

Justification for trend: The habitat of this species is inferred to be declining in area, extent and quality due to human development in particular agriculture through both relatively small-scale farming, such as seasonal cash crops (e.g. crops like corn and beans) plus livestock pasture, but also extensive industrial agricultural activities (in particular coffee, vanilla, sugar-cane, rice and citrus fruits), alongside the linked development of urbanisation plus roads and rail.

**Figure 37.**
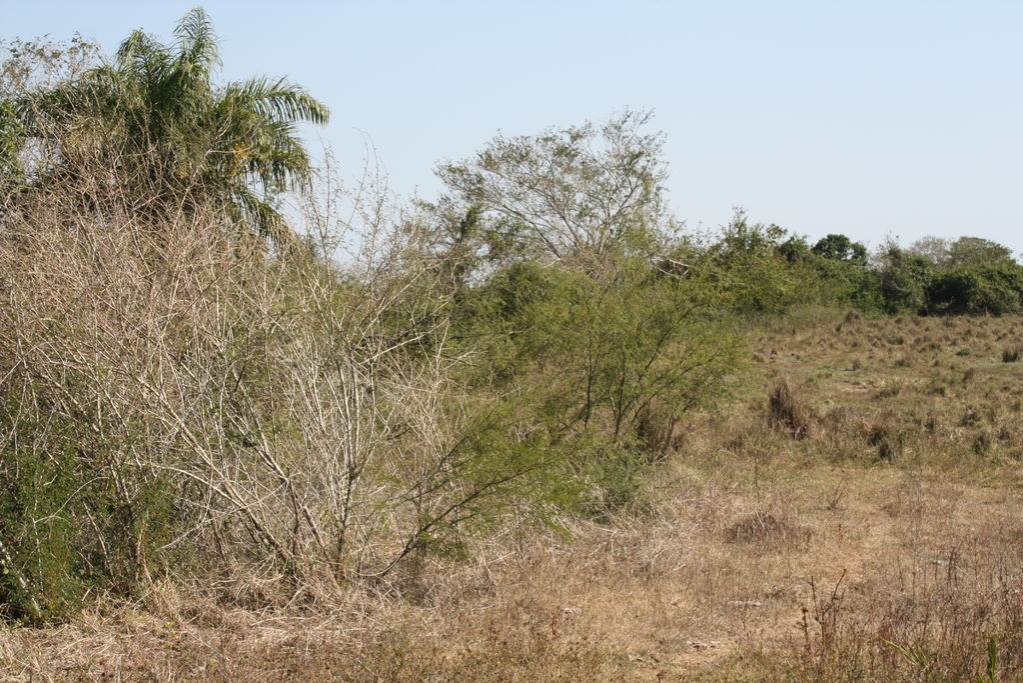
*Brachypelma kahlenbergi* habitat, human-disturbed scrub and pasture near Veracruz, Veracruz state, Mexico. Photo: Stuart J. Longhorn.

Figure(s) or Photo(s): Fig. 37

##### Habitat

Habitat importance: Major Importance

Habitats: 1.6. Forest - Subtropical/Tropical Moist Lowland

##### Habitat

Habitat importance: Suitable

Habitats: 14.2. Artificial/Terrestrial - Pastureland14.3. Artificial/Terrestrial - Plantations14.6. Artificial/Terrestrial - Subtropical/Tropical Heavily Degraded Former Forest

#### Habitat

Habitat importance: Major Importance

Habitats: 1.6. Forest - Subtropical/Tropical Moist Lowland

#### Habitat

Habitat importance: Suitable

Habitats: 14.2. Artificial/Terrestrial - Pastureland14.3. Artificial/Terrestrial - Plantations14.6. Artificial/Terrestrial - Subtropical/Tropical Heavily Degraded Former Forest

#### Ecology

Size: 70 mm (female), 60 mm (male).

Generation length (yr): 9

Dependency of single sp?: No

Ecology and traits (narrative): *Brachypelma kahlenbergi* is a fossorial species that constructs or modifies burrows, often clearly defined obligate burrow-like retreats under debris such as large rocks and tree roots in subtropical moist lowland forest, as well as scrubland and pasture. The burrows slope steeply for several centimetres, ending in a chamber. Burrows will typically have a layer of silk around the entrance to transmit the vibrations of prey movement and can be sealed with a further thin layer of silk across the diameter during daylight that may deter predators (*e.g.* ants, wasps etc.) and/or help maintain humidity inside the retreat. These spiders are nocturnal predators that wait near the entrance of their refuge from dusk and into the night to feed primarily on ground-dwelling arthropods (insects, other arachnids and some myriapods) or even small vertebrates. The presence of the frog *Engystomops pustulosus* (Cope, 1864), living in a same burrow with *Brachypelma kahlenbergi*, was reported by Hijmensen (2018). The mating season occurs during the last part of the rainy and first part of dry seasons (August to January) when mature males wander in the open to search for females. The males are likely most active at night, cooler daylight hours and throughout overcast days. Adult females typically moult once per year, just prior to the onset of the annual male emergence. Females will produce cocoons (large silken egg sacs) during the drier winter months with young emerging about two months later, with most young dispersing in the late spring or early summer, just before the onset of the early summer rains.

#### Threats

Justification for threats: Despite a lack of systematic research, increasingly intense agriculture and related urbanisation and infrastructure are likely posing significant threats. The area where *B. kahlenbergi* can be found, mainly Veracruz State, is one of the most populous in Mexico, with a significant use for agriculture and cattle raising (SEMARNAT 2012). This occurs both through small-holders (particularly corn and beans) and larger agro-industry (especially coffee, vanilla, sugar-cane, rice and citrus fruits), plus livestock (with cattle ranching predominant). These agricultural activities imply increased urbanisation and are united by a network of roads and rail, linking them both to other cities in Mexico and export via the international port of Veracruz. Roads can also be considered a threat, since they disturb the natural habitat and increase the risk of males being run over during the mating season, as well as facilitate the access of smugglers to wild populations. There is a legal trade of captive-bred live specimens, but also an unknown amount of illegal pet trade.

##### Threats

Threat type: Ongoing

Threats: 1.1. Residential & commercial development - Housing & urban areas2.1.2. Agriculture & aquaculture - Annual & perennial non-timber crops - Small-holder farming2.1.3. Agriculture & aquaculture - Annual & perennial non-timber crops - Agro-industry farming2.3.2. Agriculture & aquaculture - Livestock farming & ranching - Small-holder grazing, ranching or farming2.3.3. Agriculture & aquaculture - Livestock farming & ranching - Agro-industry grazing, ranching or farming4.1. Transportation & service corridors - Roads & railroads5.1.1. Biological resource use - Hunting & trapping terrestrial animals - Intentional use (species is the target)

#### Threats

Threat type: Ongoing

Threats: 1.1. Residential & commercial development - Housing & urban areas2.1.2. Agriculture & aquaculture - Annual & perennial non-timber crops - Small-holder farming2.1.3. Agriculture & aquaculture - Annual & perennial non-timber crops - Agro-industry farming2.3.2. Agriculture & aquaculture - Livestock farming & ranching - Small-holder grazing, ranching or farming2.3.3. Agriculture & aquaculture - Livestock farming & ranching - Agro-industry grazing, ranching or farming4.1. Transportation & service corridors - Roads & railroads5.1.1. Biological resource use - Hunting & trapping terrestrial animals - Intentional use (species is the target)

#### Conservation

Justification for conservation actions: *Brachypelma kahlenbergi* was recorded in Los Tuxtlas Biosphere Reserve and in the Biosphere Reserve of Sierra Gorda, but it is likely to occur also in other conservation units in Mexico. Important conservation actions include protecting the natural habitat by creating management plans, conducting systematic monitoring to provide information on the recovery of populations and re-introduce the species in some areas. In order to avoid international trade incompatible with its survival, this species is currently listed on CITES Appendix II, along with all other species of the genus *Brachypelma* (CITES 2019). *Brachypelma kahlenbergi* is being reared in captivity in Mexican Units for Management (UMAs) and sold legally. There is legal trade of captive-bred live specimens, but also an unknown amount of illegal pet trade of live animals, which needs to be curbed. It is also necessary to establish tax advantages for legal dealers in order to make their prices more competitive with the ones in the black market.

##### Conservation actions

Conservation action type: In Place

Conservation actions: 1.1. Land/water protection - Site/area protection3.4.1. Species management - Ex-situ conservation - Captive breeding/artificial propagation5.1.1. Law & policy - Legislation - International level

##### Conservation actions

Conservation action type: Needed

Conservation actions: 1.2. Land/water protection - Resource & habitat protection2.3. Land/water management - Habitat & natural process restoration3.2. Species management - Species recovery3.3. Species management - Species re-introduction5.4.1. Law & policy - Compliance and enforcement - International level5.4.2. Law & policy - Compliance and enforcement - National level5.4.3. Law & policy - Compliance and enforcement - Sub-national level6.4. Livelihood, economic & other incentives - Conservation payments

#### Conservation actions

Conservation action type: In Place

Conservation actions: 1.1. Land/water protection - Site/area protection3.4.1. Species management - Ex-situ conservation - Captive breeding/artificial propagation5.1.1. Law & policy - Legislation - International level

#### Conservation actions

Conservation action type: Needed

Conservation actions: 1.2. Land/water protection - Resource & habitat protection2.3. Land/water management - Habitat & natural process restoration3.2. Species management - Species recovery3.3. Species management - Species re-introduction5.4.1. Law & policy - Compliance and enforcement - International level5.4.2. Law & policy - Compliance and enforcement - National level5.4.3. Law & policy - Compliance and enforcement - Sub-national level6.4. Livelihood, economic & other incentives - Conservation payments

#### Other

Justification for use and trade: *Brachypelma kahlenbergi* is being reared in captivity in Mexican Units for Management (UMAs) and sold legally. This species is currently listed on CITES Appendix II and thus its international trade is regulated by an international agreement (CITES 2019). A legal trade of captive-bred live specimens is recorded, but also an unknown amount of illegal pet trade of live animals. Captive-bred juveniles are sold for approximately US$65–$80 in Canada and the United States, for US$20 in Mexico and for US$5 in the EU; adult females are sold for approximately US$80 in the EU (CEC 2017). This species does not have a high market demand and most trade is done by specialised dealers. Additionally, *B. kahlenbergi* has often been misidentified in the pet trade with other species of *Brachypelma* with red abdomen, such as *B. vagans* and *B. sabulosum*. As just 25–35 specimens of *B. kahlenbergi* were traded internationally during 2006–2016, all alive, for commercial purposes and none declared as wild-caught, it is considered an uncommon species in international trade (Cooper et al. 2019).

##### Use and trade

Use type: International

Use and trade: 13. Pets/display animals, horticulture16. Establishing ex-situ production *

##### Ecosystem services

Ecosystem service type: Less important

##### Research needed

Research needed: 1.5. Research - Threats3.1. Monitoring - Population trends3.4. Monitoring - Habitat trends

Justification for research needed: Some protected areas can be found in the range of *Brachypelma kahlenbergi*. However, there is no systematic monitoring of wild populations or their habitat. As this species occurs in some readily accessible conservation units such as Los Tuxtlas Biosphere Reserve, it is desirable to initiate monitoring of subpopulations, habitats and their trends. Research about the impact of habitat degradation on the population viability should be conducted and, consequently, solutions to promote sustainability could be proposed.

#### Use and trade

Use type: International

Use and trade: 13. Pets/display animals, horticulture16. Establishing ex-situ production *

#### Ecosystem services

Ecosystem service type: Less important

#### Research needed

Research needed: 1.5. Research - Threats3.1. Monitoring - Population trends3.4. Monitoring - Habitat trends

Justification for research needed: Some protected areas can be found in the range of *Brachypelma kahlenbergi*. However, there is no systematic monitoring of wild populations or their habitat. As this species occurs in some readily accessible conservation units such as Los Tuxtlas Biosphere Reserve, it is desirable to initiate monitoring of subpopulations, habitats and their trends. Research about the impact of habitat degradation on the population viability should be conducted and, consequently, solutions to promote sustainability could be proposed.

#### Viability analysis

### Brachypelma klaasi

#### Species information

Scientific name: Brachypelma klaasi

Species authority: (Schmidt & Krause, 1994)

Synonyms: *Brachypelmides klaasi* Schmidt & Krause, 1994

Common names: Mexican pink beauty, Mexican pink tarantula, tarántula mexicana rosada, mygale rose mexicaine.

Kingdom: Animalia

Phylum: Arthropoda

Class: Arachnida

Order: Araneae

Family: Theraphosidae

**Figure 38.**
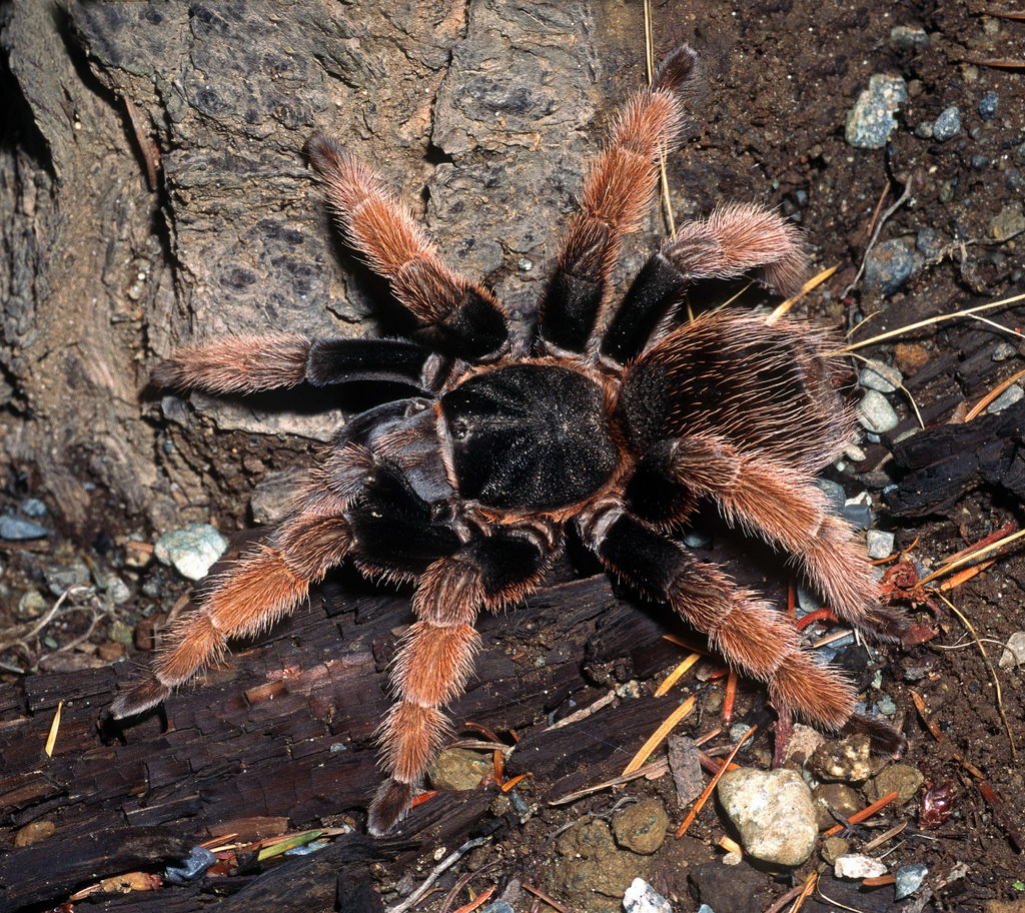
*Brachypelma klaasi* (Schmidt & Krause, 1994) female near Chamela, Jalisco state, Mexico. Photo: Rick C. West.

**Figure 39.**
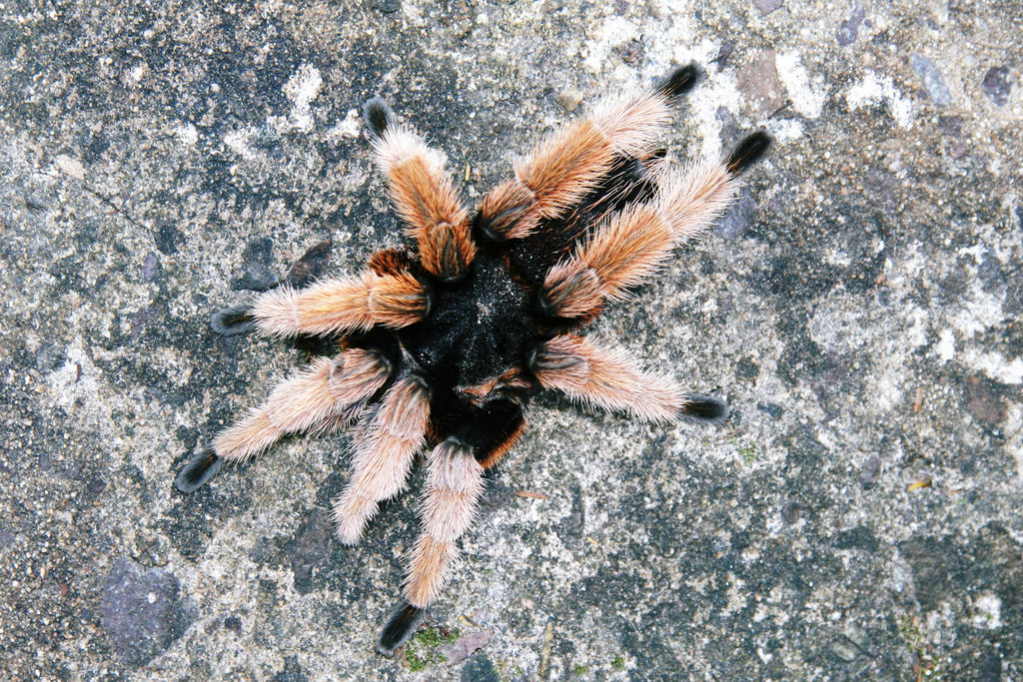
*Brachypelma klaasi* (Schmidt & Krause, 1994) male near Chamela, Jalisco state, Mexico. Photo: Andrew M. Smith.

Figure(s) or Photo(s): Figs 38, 39

Region for assessment: Global

#### Geographic range

Biogeographic realm: Neotropical

Countries: Mexico

**Figure 40.**
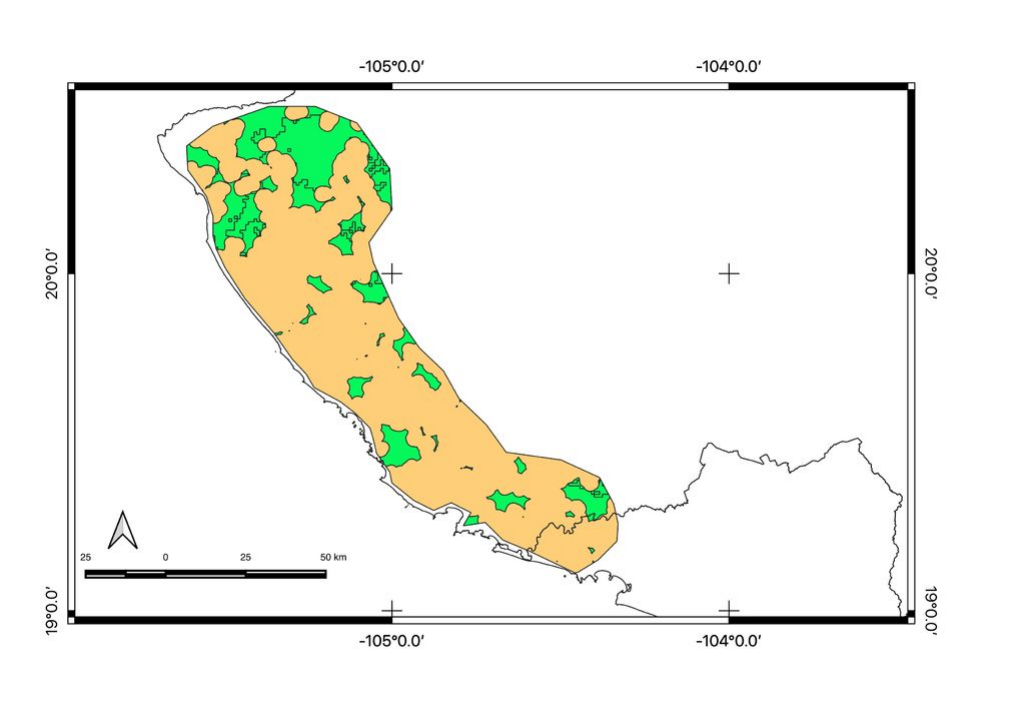
Habitat quality across the distribution of *Brachypelma klaasi* (Schmidt & Krause, 1994). Low quality habitat areas (LQH) in orange and high quality habitat areas (HQH) in green (for estimation details, see formula in Methods).

Map of records (image): Fig. 40

Map of records (Google Earth): Suppl. material 13

Basis of EOO and AOO: Species Distribution Model

Basis (narrative): A species distribution modelling has been performed to predict its potential range. See methods for details.

Min Elevation/Depth (m): 0

Max Elevation/Depth (m): 1000

Range description: *Brachypelma klaasi* is endemic to Mexico and occurs on the Pacific coastal side of the Sierra Madre Occidental range, in south-western Jalisco State into north-western Colima State where it is sympatric (overlapping in range) with *Brachypelma hamorii* in a relatively small area ([Bibr B4347927][Bibr B4347927], CEC 2017, iNaturalist 2018, unpublished data). Most northernmost records extend to south of Puerto Vallarta in Jalisco State, but recently a small population was found in Nayarit (unpublished data), resulting in sympatry with *B. emilia*. Yáñez and Floater (2000) stated that the species can be found at elevations of 300-1,400 m a.s.l. on the extreme western slopes of Sierra Madre Occidental and some areas on the western limits of the Transverse Volcanic Belt in Jalisco and Nayarit States.

#### New occurrences

#### Extent of occurrence

EOO (km2): 6438

Trend: Decline (inferred)

Justification for trend: A decline in EOO can be inferred, since there is habitat loss due to human activity, such as urbanisation, livestock and agricultural activities and roads.

Causes ceased?: No

Causes understood?: Yes

Causes reversible?: No

Extreme fluctuations?: No

#### Area of occupancy

Trend: Decline (inferred)

Justification for trend: There is a decrease in AOO, since there is habitat loss due to human activity, such as urbanisation, livestock and agricultural activities and roads.

Causes ceased?: No

Causes understood?: Yes

Causes reversible?: No

Extreme fluctuations?: No

AOO (km2): 2576

#### Locations

Number of locations: 100

Justification for number of locations: Derived from the approximate number of human populations above 100 inhabitants needed to cover the entire range of the species. We assume each locality covers a mean radius of 2.5 km.

Trend: Decline (inferred)

Extreme fluctuations?: Unknown

#### Population

Number of individuals: Unknown

Trend: Decline (inferred)

Justification for trend: A decline in population size is inferred from a possible decline in AOO and a loss of habitat due to human activity, such as urbanisation, livestock and agricultural activities and roads. In recent years, specimens are hard to find in some sites where they used to be abundant. Harvesting to feed the illegal pet trade can also be considered a cause of decline in population size, although typically not as intense as experienced by some other *Brachypelma* species.

Basis for decline: (c) a decline in area of occupancy, extent of occurrence and/or quality of habitat(d) actual or potential levels of exploitation

Causes ceased?: No

Causes understood?: Yes

Causes reversible?: No

Extreme fluctuations?: No

Population Information (Narrative): A decline in population size is inferred from a possible decline in AOO and a loss of habitat due to human activities, such as urbanisation, livestock and agricultural activities and roads. In recent years, specimens have been hard to find in some sites where they used to be abundant. Harvesting to feed the illegal pet trade can also be considered a cause of decline in population size, although typically not as intense as experienced by some other *Brachypelma* species.

#### Subpopulations

Trend: Decline (inferred)

Justification for trend: A decrease in number of subpopulations of *B. klaasi* is inferred from a possible decline in AOO, since there is habitat loss due to human activity, such as urbanisation, livestock and agricultural activities and roads. Harvesting of wild animals is also leading to the disappearance of some subpopulations of this species.

Extreme fluctuations?: No

Severe fragmentation?: Yes

Justification for fragmentation: Based on the analysis of the relative abundance of each species in low quality habitat (RA_LQH_) and the Species Distribution Model, 77.05% of the population should be in subpopulations that are non-viable and without the possibility of rescue effects due to fragmentation.

#### Habitat

System: Terrestrial

Habitat specialist: Yes

Habitat (narrative): *Brachypelma klaasi* inhabits the subtropical dry forest in the foothills of the Sierra Madre del Sur mountains, in the coastal Pacific of Mexico. It can be found in thorn forest and dry deciduous forest in coastal lowlands and up into pine-oak forest habitats at higher elevations. The species can be found from a low altitude almost at sea level, above the dunes, up to 1,000 m, at the Sierra El Tuito range.

Trend in extent, area or quality?: Decline (inferred)

Justification for trend: The habitat of this species is inferred to be declining in area, extent and quality due to intense human activities in particular urbanisation and agricultural expansion. At higher altitudes, pines and oak trees are being increasingly felled for timber and the increase in agriculture (in particular corn fields) are increasingly reducing species habitat.

**Figure 41.**
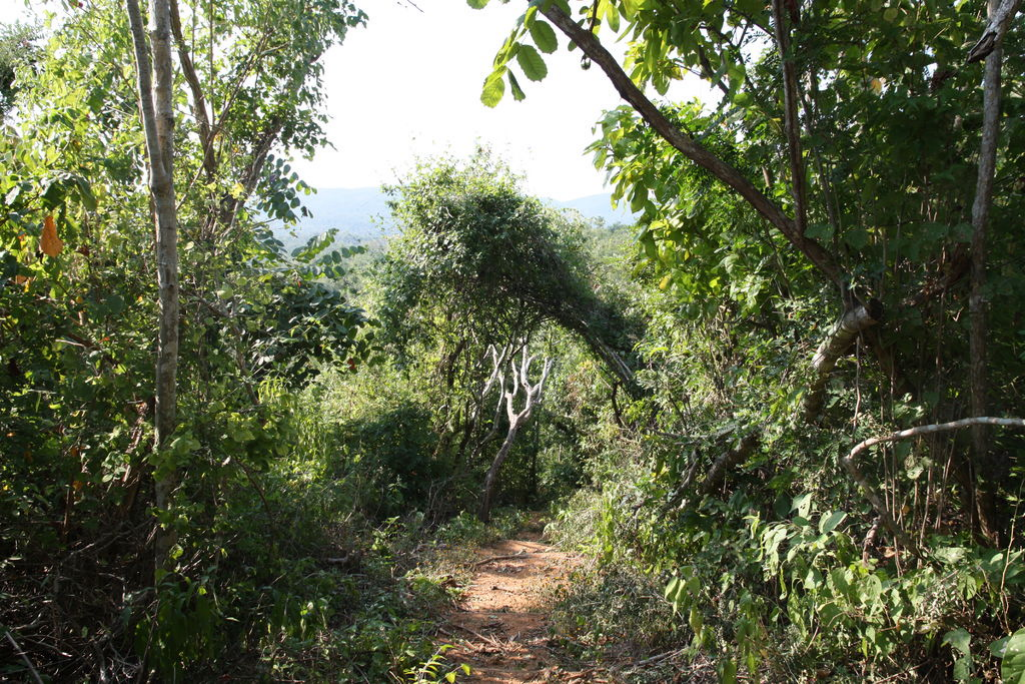
*Brachypelma klaasi* habitat, subtropical deciduous forest, near Chamela, Jalisco state, Mexico. Photo: Stuart J. Longhorn.

Figure(s) or Photo(s): Fig. 41

##### Habitat

Habitat importance: Major Importance

Habitats: 1.5. Forest - Subtropical/Tropical Dry

#### Habitat

Habitat importance: Major Importance

Habitats: 1.5. Forest - Subtropical/Tropical Dry

#### Ecology

Size: 50-75 mm (Yánez et al. 1999).

Generation length (yr): 9

Dependency of single sp?: No

Ecology and traits (narrative): This species, along with *Brachypelma vagans*, is one of the better-known for its ecological traits amongst *Brachypelma* species. *Brachypelma klaasi* are a fossorial species that modifies previously excavated burrows or can excavate their own unaided, often with significant alterations to natural small cavities or more defined burrow-like retreats under debris, such as large rocks and tree roots in dense vegetation of thorn and dry deciduous forest or pine-oak forests. Burrows have been recorded amongst dense thorny thickets, in rotting fallen pine trees and old rotten logged pine stumps or in dense tall grasses (West 2005). It has been suggested that immature spiders transiently exploit various refuges until they find a suitable site for a permanent burrow that they can then inhabit for many years (Yáñez and Floater 2000). The burrow varies in length from 0.15 to 2 m, depending on the site and the age of the spider (Yáñez et al. 1999) and consists of a horizontal tunnel leading from the burrow entrance to a primary chamber where moulting usually takes place and a larger, secondary chamber where the spider rests during the night and where prey is consumed, both connected by an inclined tunnel (Yáñez et al. 1999). The burrows (of older individuals) have no traces of silk at the entrance giving no clear indication that there is a spider inside. These spiders are nocturnal predators that wait near the entrance of their refuge from dusk and into the night to feed primarily on ground-dwelling arthropods (insects, other arachnids and some myriapods) or even small vertebrates. Known prey items of *B. klaasi* which have been recorded are large insects, such as Orthoptera and Blattodea, as well as small lizards and frogs (Yáñez 1998). The species is diurnal and is most active in the early morning and early evening (Yáñez and Floater 2000). The mating season occurs during the last part of rainy and first part of dry seasons (August to January) when mature males wander in the open to search for females. Adult males of the species do not appear to have fixed home ranges and move frequently in search of females, that remain within or close to their permanent burrow (Yáñez and Floater 2000). The males are likely most active at night, cooler daylight hours and throughout overcast days. Adult females typically moult once per year, just prior to the onset of the annual male emergence. After copulation, they produce cocoons (large silken egg sacs) containing 400–800 eggs in their burrows in April–May, immediately before the first rains of the season and the spiderlings only emerge in June–July (Yáñez and Floater 2000). The spiderlings disperse in July–August and are prone to predation for other animals such as ants (Yáñez and Floater 2000). Females mature between 7-9 years and live up to 30 years in captivity while males probably mature earlier (between six and eight years) and live four to six months more after maturity (Yáñez et al. 1999).

#### Threats

Justification for threats: There are some factors that threaten populations of *B. klaasi*, especially related to human activities, such as urbanisation and agricultural expansion. This species, along with the others of the red leg complex, are more fragile and do not adapt well or quickly to alterations in the environment caused by human activities (Turner et al. 2018). Vehicular traffic on roads can also be considered as a threat to this species, since male specimens can be hit by cars while dispersing during the mating season. Adding to this, roads facilitate the access of smugglers to wild populations and also are a source *per se* of habitat degradation. Red leg species are more popular as pets than the red abdomen ones. There is legal trade of captive-bred live specimens, but also an unknown amount of illegal pet trade of live animals, which can also be considered as a threat.

##### Threats

Threat type: Ongoing

Threats: 1.1. Residential & commercial development - Housing & urban areas2.1.2. Agriculture & aquaculture - Annual & perennial non-timber crops - Small-holder farming2.1.3. Agriculture & aquaculture - Annual & perennial non-timber crops - Agro-industry farming2.2.1. Agriculture & aquaculture - Wood & pulp plantations - Small-holder plantations2.3.2. Agriculture & aquaculture - Livestock farming & ranching - Small-holder grazing, ranching or farming2.3.3. Agriculture & aquaculture - Livestock farming & ranching - Agro-industry grazing, ranching or farming4.1. Transportation & service corridors - Roads & railroads5.1.1. Biological resource use - Hunting & trapping terrestrial animals - Intentional use (species is the target)

#### Threats

Threat type: Ongoing

Threats: 1.1. Residential & commercial development - Housing & urban areas2.1.2. Agriculture & aquaculture - Annual & perennial non-timber crops - Small-holder farming2.1.3. Agriculture & aquaculture - Annual & perennial non-timber crops - Agro-industry farming2.2.1. Agriculture & aquaculture - Wood & pulp plantations - Small-holder plantations2.3.2. Agriculture & aquaculture - Livestock farming & ranching - Small-holder grazing, ranching or farming2.3.3. Agriculture & aquaculture - Livestock farming & ranching - Agro-industry grazing, ranching or farming4.1. Transportation & service corridors - Roads & railroads5.1.1. Biological resource use - Hunting & trapping terrestrial animals - Intentional use (species is the target)

#### Conservation

Justification for conservation actions: Although some stable populations of this species can be found in Chamela-Cuixmala Biosphere Reserve, important conservation actions include protecting other areas of the natural habitat of *B. klaasi*, creating management plans and conducting systematic monitoring to provide information about the recovery of populations that are in decline due to human activities. Several studies suggest that mating *B. klaasi* in captivity is not difficult and the production of egg sacs in the laboratory can be successful (Yáñez et al. 1999). Thus, since captive breeding and re-introduction of *B. klaasi* is an important means of sustaining natural populations (Yáñez et al. 1999), some efforts for stimulating these activities should be undertaken. In order to avoid international trade incompatible with its survival, this species is currently listed on CITES Appendix II, along with all other *Brachypelma* species (CITES 2019). *Brachypelma klaasi* is being reared in captivity in Mexican Units for Management (UMAs) and sold legally. Despite the legal trade of captive-bred live specimens, there is also an unknown amount of trafficking of live animals that demands better border enforcement. Thus it is necessary to develop better enforcement actions to curb trafficking, as well as to establish tax advantages for legal dealers in order to make their prices more competitive with the ones in the black market. Currently, this species is being reared in captivity in a UMA that also promotes educational and awareness activities regarding tarantula spiders aimed at students and the general public.

##### Conservation actions

Conservation action type: In Place

Conservation actions: 1.1. Land/water protection - Site/area protection3.4.1. Species management - Ex-situ conservation - Captive breeding/artificial propagation5.1.1. Law & policy - Legislation - International level4.3. Education & awareness - Awareness & communications

##### Conservation actions

Conservation action type: Needed

Conservation actions: 1.1. Land/water protection - Site/area protection3.2. Species management - Species recovery3.3.1. Species management - Species re-introduction - Reintroduction5.4.1. Law & policy - Compliance and enforcement - International level5.4.2. Law & policy - Compliance and enforcement - National level5.4.3. Law & policy - Compliance and enforcement - Sub-national level6.4. Livelihood, economic & other incentives - Conservation payments

#### Conservation actions

Conservation action type: In Place

Conservation actions: 1.1. Land/water protection - Site/area protection3.4.1. Species management - Ex-situ conservation - Captive breeding/artificial propagation5.1.1. Law & policy - Legislation - International level4.3. Education & awareness - Awareness & communications

#### Conservation actions

Conservation action type: Needed

Conservation actions: 1.1. Land/water protection - Site/area protection3.2. Species management - Species recovery3.3.1. Species management - Species re-introduction - Reintroduction5.4.1. Law & policy - Compliance and enforcement - International level5.4.2. Law & policy - Compliance and enforcement - National level5.4.3. Law & policy - Compliance and enforcement - Sub-national level6.4. Livelihood, economic & other incentives - Conservation payments

#### Other

Justification for use and trade: *Brachypelma klaasi* is being reared in captivity in Mexican Units for Management (UMAs) and sold legally. This species is currently listed on CITES Appendix II and thus is protected by an international agreement (CITES 2019). A legal trade in captive-bred live specimens is recorded, but an unquantified amount of wild-caught animals illegally traded is also known. It seems to be rare, but adults appear at times from unknown sources, increasing demand when available. Captive-bred juveniles are sold for approximately US$70–$75 (up to US$100) in Canada and the United States, for US$14 in Mexico and for US$7 in the EU; adult males are sold for approximately US$33 and adult females for approximately US$80 in the EU (CEC 2017). It has the highest price amongst all *Brachypelma* species and it is sold very quickly. *Brachypelma klaasi* is considered a common species in the international trade, since between 492–512 specimens were traded during 2006–2016, all live, none declared as wild-caught and mainly for commercial purposes (Cooper et al. 2019).

##### Use and trade

Use type: International

Use and trade: 13. Pets/display animals, horticulture16. Establishing ex-situ production *

##### Ecosystem services

Ecosystem service type: Less important

##### Research needed

Research needed: 2.3. Conservation Planning - Harvest & Trade Management Plan3.1. Monitoring - Population trends3.4. Monitoring - Habitat trends

Justification for research needed: Due to previous studies (Yáñez 1998, Yáñez and Locht 1998, Yáñez et al. 1999), *B. klaasi* can be considered one of the *Brachypelma* species with the best-known ecological features. Therefore, it would be important to concentrate research about the impact of land use and habitat degradation in the population viability of *B. klaasi* in order to help in conservation decisions, as well monitoring the population and habitat trends.

#### Use and trade

Use type: International

Use and trade: 13. Pets/display animals, horticulture16. Establishing ex-situ production *

#### Ecosystem services

Ecosystem service type: Less important

#### Research needed

Research needed: 2.3. Conservation Planning - Harvest & Trade Management Plan3.1. Monitoring - Population trends3.4. Monitoring - Habitat trends

Justification for research needed: Due to previous studies (Yáñez 1998, Yáñez and Locht 1998, Yáñez et al. 1999), *B. klaasi* can be considered one of the *Brachypelma* species with the best-known ecological features. Therefore, it would be important to concentrate research about the impact of land use and habitat degradation in the population viability of *B. klaasi* in order to help in conservation decisions, as well monitoring the population and habitat trends.

#### Viability analysis

### Brachypelma sabulosum

#### Species information

Scientific name: Brachypelma sabulosum

Species authority: (F. O. Pickard-Cambridge, 1897)

Synonyms: *Eurypelma sabulosum* F. O. Pickard-Cambridge, 1897; *Delopelma sabulosum* (F. O. Pickard-Cambridge, 1897); *Rhechostica sabulosa* (F. O. Pickard-Cambridge, 1897); *Avicularia sabulosa* (F. O. Pickard-Cambridge, 1897).

Common names: Guatemalan redrump.

Kingdom: Animalia

Phylum: Arthropoda

Class: Arachnida

Order: Araneae

Family: Theraphosidae

Taxonomic notes: Mendonza and Francke (in press) are publishing a taxonomic revision of *Brachypelma* and this species will be accommodated in a new genus.

**Figure 42.**
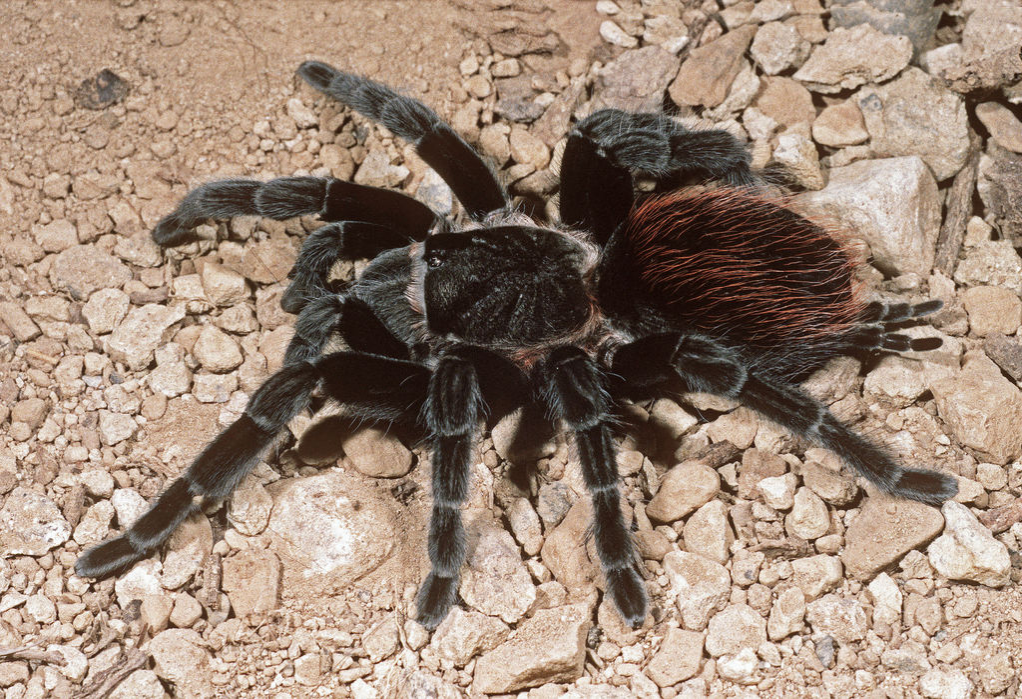
*Brachypelma sabulosum* (F. O. P.-Cambridge, 1897) female from Tikal, Guatemala. Photo: Rick C. West.

**Figure 43.**
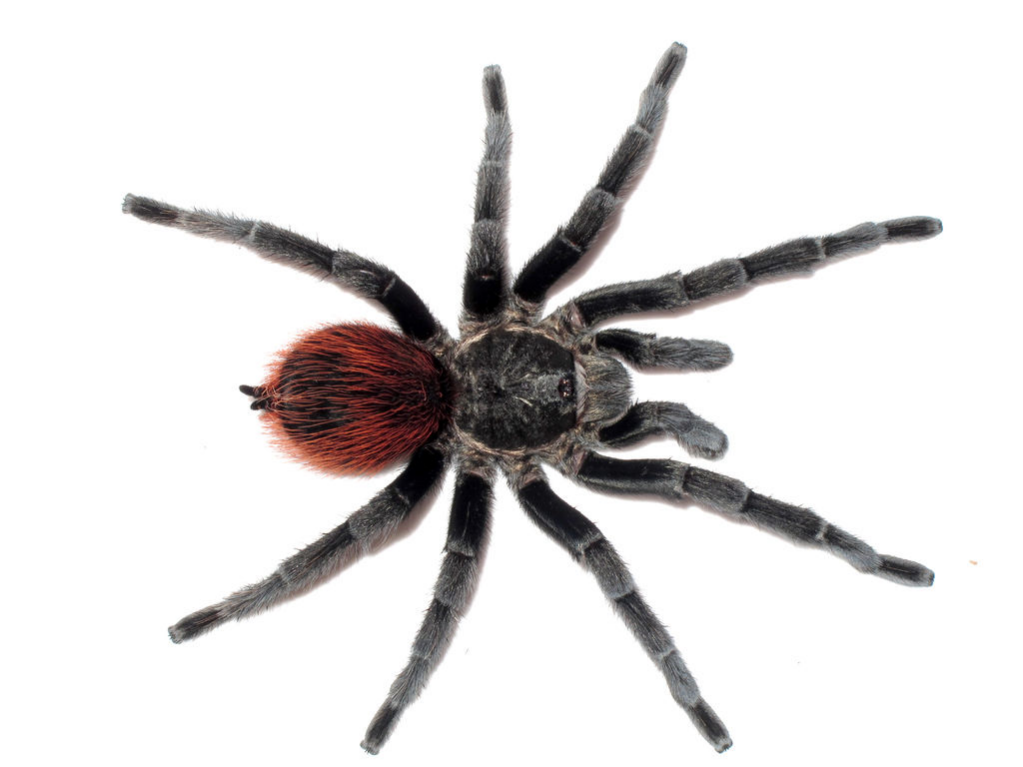
*Brachypelma sabulosum* (F. O. P.-Cambridge, 1897) male. Photo: Jorge Mendoza.

Figure(s) or Photo(s): Figs 42, 43

Region for assessment: Global

#### Geographic range

Biogeographic realm: Neotropical

Countries: GuatemalaMexico

Map of records (Google Earth): Suppl. material 14

Basis of EOO and AOO: Unknown

Basis (narrative): This species EOO and AOO are unknown.

Range description: *Brachypelma sabulosum* is only found in north-eastern Chiapas State and north and north-western Guatemala, probably across Petén, most of Alta Verapaz and to the southeast parts of Izabel and southwest to Quiché and Huehuetenango departments (Pickard-Cambridge 1897, CEC 2017, iNaturalist 2018, unpublished data). Yet, a scarcity of data prevents us from having any confidence in its existing knowledge.

#### New occurrences

#### Extent of occurrence

EOO (km2): Unknown

Trend: Unknown

Causes ceased?: Unknown

Causes understood?: Unknown

Causes reversible?: Unknown

Extreme fluctuations?: Unknown

#### Area of occupancy

Trend: Decline (inferred)

Justification for trend: Although AOO is unknown, a decline is inferred, since there is loss of habitat in its range due to high amounts of deforestation (Global Forest Watch 2019) caused by human interferences, such as urbanisation and agricultural activities within the range of this species.

Causes ceased?: No

Causes understood?: Yes

Causes reversible?: No

Extreme fluctuations?: Unknown

AOO (km2): Unknown

#### Locations

Number of locations: Unknown

Trend: Unknown

Extreme fluctuations?: No

#### Population

Number of individuals: Unknown

Trend: Decline (inferred)

Justification for trend: Population declining due to an inferred AOO reduction caused by deforestation, especially for agricultural purposes.

Basis for decline: (c) a decline in area of occupancy, extent of occurrence and/or quality of habitat

Causes ceased?: No

Causes understood?: Yes

Causes reversible?: No

Extreme fluctuations?: No

Population Information (Narrative): Population is declining due to an inferred AOO reduction caused by deforestation (Global Forest Watch 2019), especially for agricultural purposes.

#### Subpopulations

Number of subpopulations: Unknown.

Trend: Decline (inferred)

Justification for trend: The number of subpopulations is probably declining due to an inferred AOO reduction caused by deforestation (Global Forest Watch 2019), especially for agricultural purposes.

Extreme fluctuations?: No

Severe fragmentation?: No

Justification for fragmentation: To our knowledge, the species is not subject to severe fragmentation.

#### Habitat

System: Terrestrial

Habitat specialist: No

Habitat (narrative): *Brachypelma sabulosum* inhabits tropical and subtropical moist broadleaf forests of Guatemala and Mexico, in south-eastern Chiapas State. They appear to be tolerant of human-disturbance and appear to be able to adapt to open areas of pasture or non-intensive crops (without intense use of chemical pesticides).

Trend in extent, area or quality?: Decline (inferred)

Justification for trend: *Brachypelma sabulosum* inhabits tropical and subtropical moist broadleaf forests of Guatemala and Mexico, in south-eastern Chiapas State. They appear to be tolerant of human-disturbance and appear to be able to adapt to open areas of pasture or non-intensive crops (without intense use of chemical pesticides).

**Figure 44.**
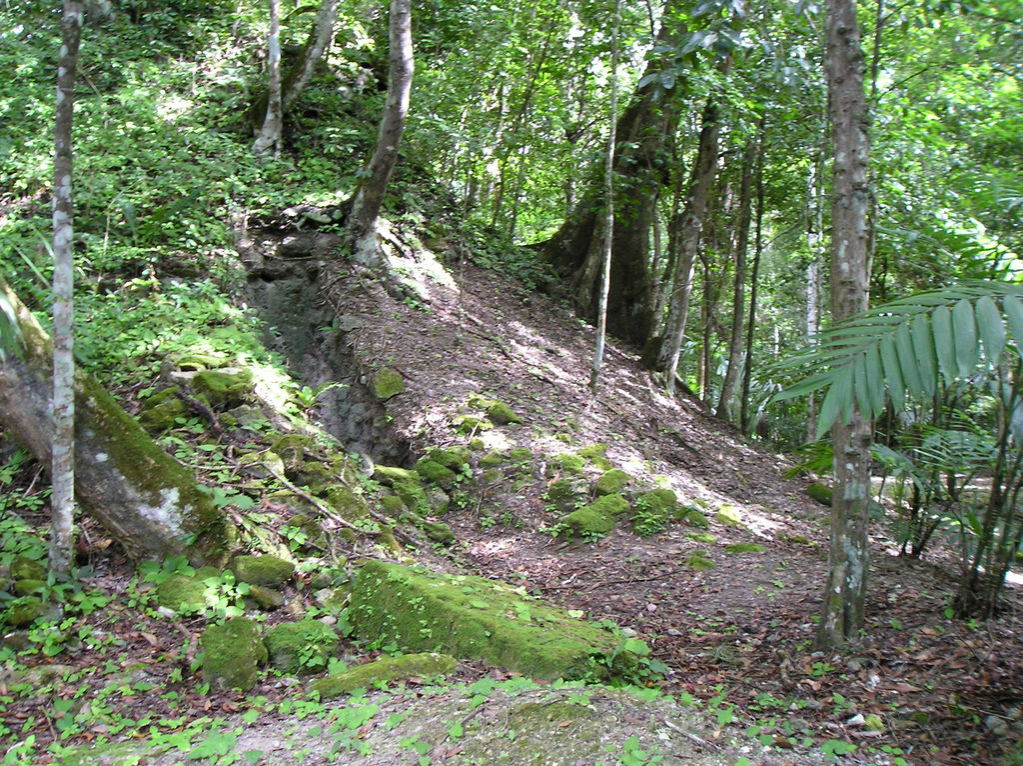
*Brachypelma sabulosum* habitat, tropical moist forest, north of of Tikal, Petén department, Guatemala. Photo: Stuart J. Longhorn.

Figure(s) or Photo(s): Fig. 44

##### Habitat

Habitat importance: Major Importance

Habitats: 1.6. Forest - Subtropical/Tropical Moist Lowland

##### Habitat

Habitat importance: Marginal

Habitats: 14.2. Artificial/Terrestrial - Pastureland14.3. Artificial/Terrestrial - Plantations14.6. Artificial/Terrestrial - Subtropical/Tropical Heavily Degraded Former Forest

#### Habitat

Habitat importance: Major Importance

Habitats: 1.6. Forest - Subtropical/Tropical Moist Lowland

#### Habitat

Habitat importance: Marginal

Habitats: 14.2. Artificial/Terrestrial - Pastureland14.3. Artificial/Terrestrial - Plantations14.6. Artificial/Terrestrial - Subtropical/Tropical Heavily Degraded Former Forest

#### Ecology

Size: 48 mm (female); 35 mm (male)

Generation length (yr): 7

Dependency of single sp?: No

Ecology and traits (narrative): *Brachypelma sabulosum* is a fossorial species that constructs or modifies burrows, often clearly defined obligate burrow-like retreats under debris in particular large rocks in tropical moist forests. The burrows slope steeply for several centimetres, ending in a chamber. Burrows will typically have some silk around the entrance to transmit the vibrations of prey movement and can be sealed further with a thin layer of silk across the diameter during daylight that may deter predators (e.g. ants, wasps etc.) and/or help maintain humidity inside the retreat. These spiders are nocturnal predators that wait near the entrance of their refuge from dusk and into the night to feed primarily on ground-dwelling arthropods (insects, other arachnids and some myriapods) or even small vertebrates. The mating season occurs towards the end of the year (August to January) when mature males wander in the open to search for females. The males are likely most active at night, cooler daylight hours and throughout overcast days. Adult females typically moult once per year, just prior to the onset of the annual male emergence. Females will produce cocoons (large silken egg sacs) during the drier winter months with young emerging about two months later, with most young dispersing in the late spring or early summer, just before the onset of the early summer rains.

#### Threats

Justification for threats: Despite a lack of systematic research, increasingly intense agriculture and related urbanisation and infrastructure are likely posing significant threats. The area where *B. sabulosum* can be found, mainly Petén department in Guatemala and Chiapas State in Mexico, is experiencing significant deforestation in recent years, coming from a rapidly growing human population along with poorly practised ranching, agricultural activities invading the forest and large amounts of timber extraction (The Nature Conservancy 2018), hence habitat loss due to deforestation can be considered a serious threat. This occurs through both small holders clearing forest for crops (especially corn, beans, potatoes, tomatoes) as well as livestock (with cattle ranching predominant).

##### Threats

Threat type: Ongoing

Threats: 1.1. Residential & commercial development - Housing & urban areas2.1.2. Agriculture & aquaculture - Annual & perennial non-timber crops - Small-holder farming2.3.2. Agriculture & aquaculture - Livestock farming & ranching - Small-holder grazing, ranching or farming

#### Threats

Threat type: Ongoing

Threats: 1.1. Residential & commercial development - Housing & urban areas2.1.2. Agriculture & aquaculture - Annual & perennial non-timber crops - Small-holder farming2.3.2. Agriculture & aquaculture - Livestock farming & ranching - Small-holder grazing, ranching or farming

#### Conservation

Justification for conservation actions: Little is known about *B. sabulosum*. However, it is known that a huge area where the species occurs, the one around Sierra del Lacadón National Park, is suffering significant pressure due to a growing human population along with poorly practised ranching, agricultural activities encroaching on the forest and timber extraction (The Nature Conservancy 2018). The Maya forest, the New World's largest remaining block of tropical rainforest outside of the Amazon basin (The Nature Conservancy 2018), covers most of the poorly known occurrence area of *B. sabulosum*, but the species has high potential to be found also in large areas to the South of this, outside any protected areas. Thus, conservation actions that integrate both Mayan and non-indigenous communities that live and explore Sierra del Lacandón and intend to improve their economic well-being and at the same time that preserve the Maya Forest, are highly desirable. It is clear that restoration, management and protection of the remaining areas is imperative for conservation, not only of *B. sabulosum*, but of this unique and rich ecosystem. The species was recorded in Tikal National Park and, despite not being officially detected, it probably occurs at Sierra del Lacadón National Park areas that can be suitable for developing conservation initiatives. Despite little information about the trade and the population trends of *B. sabulosum*, this species is currently listed on CITES Appendix II, along with all other species of the genus (CITES 2019).

##### Conservation actions

Conservation action type: In Place

Conservation actions: 1.1. Land/water protection - Site/area protection5.1.1. Law & policy - Legislation - International level

##### Conservation actions

Conservation action type: Needed

Conservation actions: 1.1. Land/water protection - Site/area protection1.2. Land/water protection - Resource & habitat protection2.1. Land/water management - Site/area management2.3. Land/water management - Habitat & natural process restoration6.1. Livelihood, economic & other incentives - Linked enterprises & livelihood alternatives

#### Conservation actions

Conservation action type: In Place

Conservation actions: 1.1. Land/water protection - Site/area protection5.1.1. Law & policy - Legislation - International level

#### Conservation actions

Conservation action type: Needed

Conservation actions: 1.1. Land/water protection - Site/area protection1.2. Land/water protection - Resource & habitat protection2.1. Land/water management - Site/area management2.3. Land/water management - Habitat & natural process restoration6.1. Livelihood, economic & other incentives - Linked enterprises & livelihood alternatives

#### Other

Justification for use and trade: This species is currently listed on CITES Appendix II and thus its international trade is regulated by an international agreement (CITES 2019). A single live specimen of *B. sabulosum* was reportedly exported from Germany to Switzerland in 2015 during the period between 2006–2016 (Cooper et al. 2019). The specimen was captive-bred and traded for a circus/travelling exhibition, reported by the exporting country (Germany) but not the importing country (Switzerland) (Cooper et al. 2019). Illegal pet trade of *B. sabulosum* does not seem to be too common. Advertisements selling *B. sabulosum* juveniles (25–30 mm in body size) for €25 can be found in web pages, but nothing is known about the legality of their origin.

##### Use and trade

Use type: International

Use and trade: 13. Pets/display animals, horticulture

##### Ecosystem services

Ecosystem service type: Less important

##### Research needed

Research needed: 1.1. Research - Taxonomy1.2. Research - Population size, distribution & trends1.3. Research - Life history & ecology

Justification for research needed: Since there are just a few records of *B. sabulosum*, the identity of the species is poorly known and it is necessary to prioritise and support basic research on taxonomy, ecology and distribution of the species.

#### Use and trade

Use type: International

Use and trade: 13. Pets/display animals, horticulture

#### Ecosystem services

Ecosystem service type: Less important

#### Research needed

Research needed: 1.1. Research - Taxonomy1.2. Research - Population size, distribution & trends1.3. Research - Life history & ecology

Justification for research needed: Since there are just a few records of *B. sabulosum*, the identity of the species is poorly known and it is necessary to prioritise and support basic research on taxonomy, ecology and distribution of the species.

#### Viability analysis

### Brachypelma schroederi

#### Species information

Scientific name: Brachypelma schroederi

Species authority: Rudloff, 2003

Common names: Mexican blackvelvet, tarántula mexicana de terciopelo negro, mygale de velours noir mexicaine.

Kingdom: Animalia

Phylum: Arthropoda

Class: Arachnida

Order: Araneae

Family: Theraphosidae

Taxonomic notes: Mendonza and Francke (in press) are publishing a taxonomic revision of *Brachypelma* and this species will be accommodated in a new genus.

**Figure 45.**
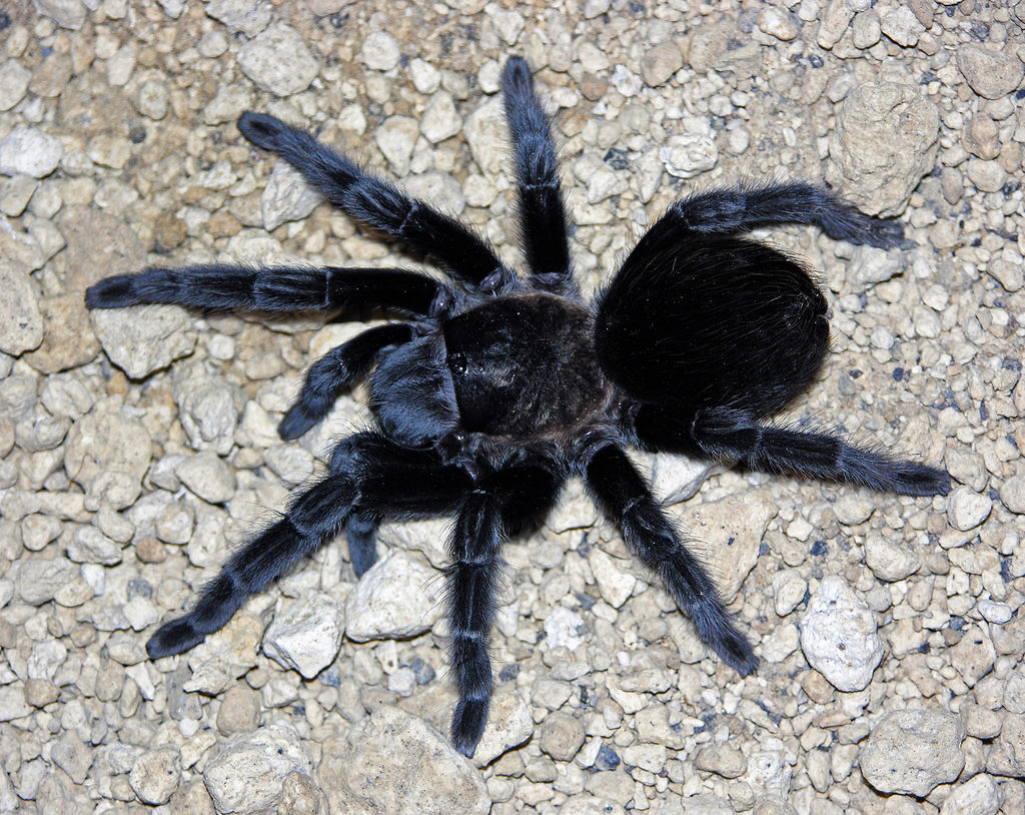
*Brachypelma schroederi* Rudloff, 2003 female from Mitla, Oaxaca state, Mexico. Photo: Rick C. West.

**Figure 46.**
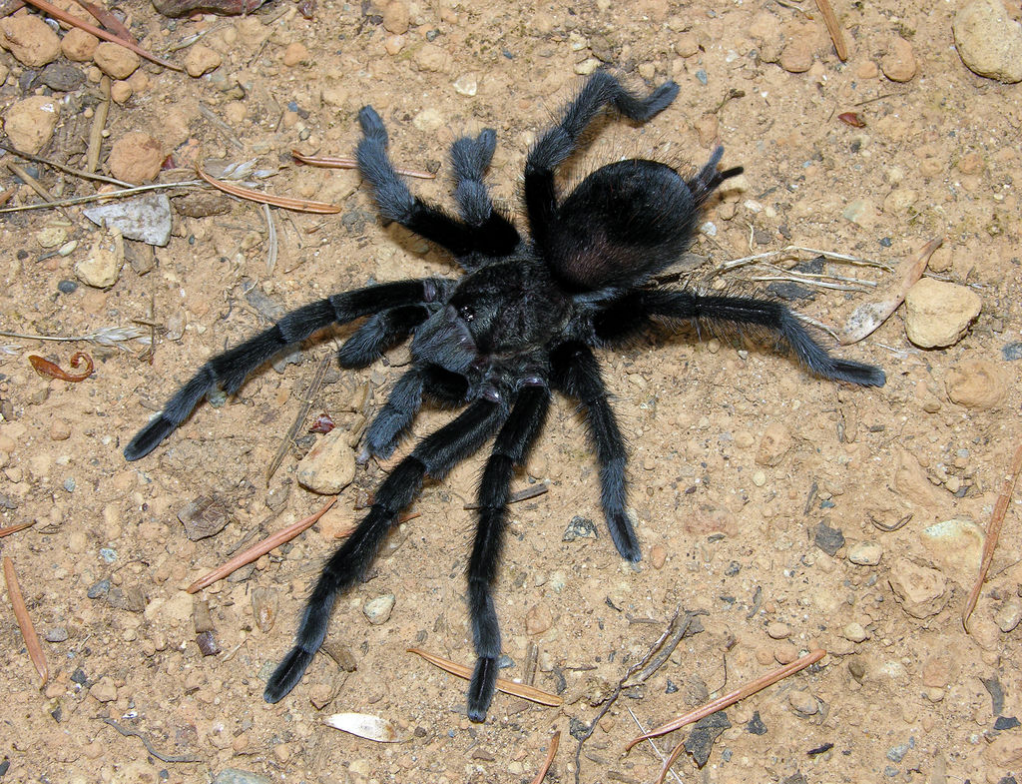
*Brachypelma schroederi* Rudloff, 2003 male from Mitla, Oaxaca State, Mexico. Photo: Rick C. West.

Figure(s) or Photo(s): Figs 45, 46

Region for assessment: Global

#### Geographic range

Biogeographic realm: Nearctic

Countries: Mexico

**Figure 47.**
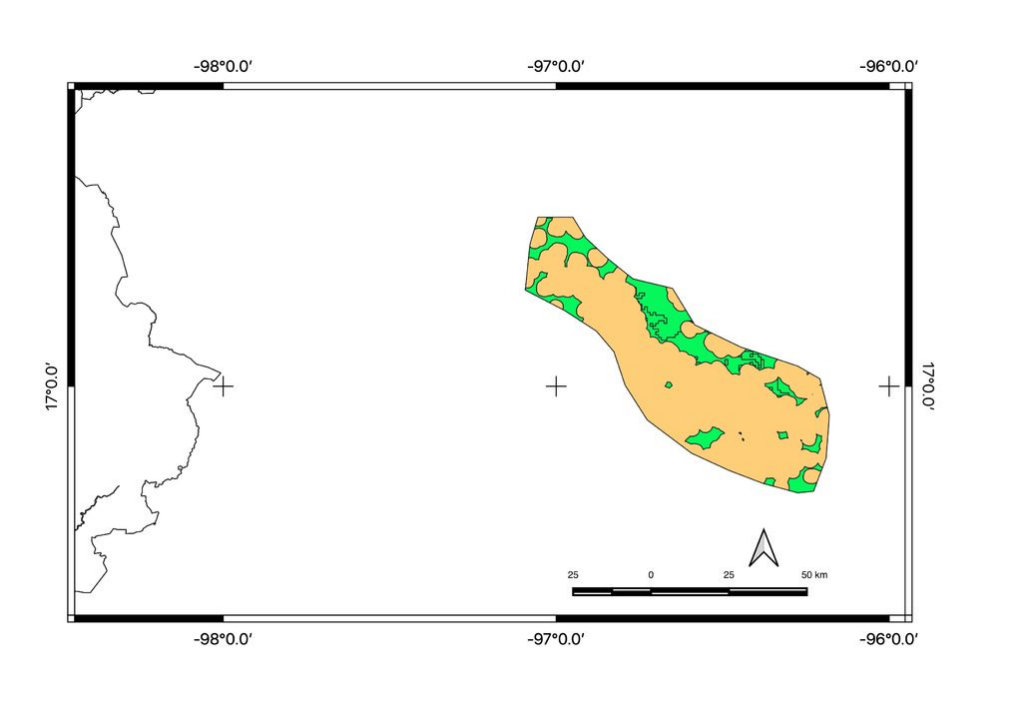
Habitat quality across the distribution of *Brachypelma schroederi* Rudloff, 2008. Low quality habitat areas (LQH) in orange and high quality habitat areas (HQH) in green (for estimation details, see formula in Methods).

Map of records (image): Fig. 47

Map of records (Google Earth): Suppl. material 15

Basis of EOO and AOO: Species Distribution Model

Basis (narrative): Despite few collection sites recorded for this species, it was possible to perform species distribution modelling to predict its potential range. See methods for details.

Min Elevation/Depth (m): 1040

Max Elevation/Depth (m): 3000

Range description: *Brachypelma schroederi* is endemic to Mexico and occurs in the Central Valley region of Oaxaca State (Rudloff 2003, CEC 2017, iNaturalist 2018, unpublished data).

#### New occurrences

#### Extent of occurrence

EOO (km2): 3775

Trend: Decline (inferred)

Justification for trend: The EOO is decreasing, since there is habitat loss due to human activities, such as urbanisation and agriculture.

Causes ceased?: No

Causes understood?: Yes

Causes reversible?: No

Extreme fluctuations?: No

#### Area of occupancy

Trend: Decline (inferred)

Justification for trend: The AOO is decreasing, since there is habitat loss due to human activities, such as urbanisation and agriculture.

Causes ceased?: No

Causes understood?: Yes

Causes reversible?: No

Extreme fluctuations?: No

AOO (km2): 2688

#### Locations

Number of locations: 100

Justification for number of locations: Derived from the approximate number of human populations above 100 inhabitants needed to cover the entire range of the species. We assume each locality covers a mean radius of 2.5 km.

Trend: Decline (inferred)

Justification for trend: We assume the number of locations to be decreasing given the generalised loss of subpopulations of this species.

Extreme fluctuations?: No

#### Population

Number of individuals: Unknown

Trend: Decline (inferred)

Justification for trend: A decline in population size is inferred through decline in AOO due to human activities, such as urbanisation and agriculture, as well as overharvesting of specimens for the trade.

Basis for decline: (c) a decline in area of occupancy, extent of occurrence and/or quality of habitat(d) actual or potential levels of exploitation

Causes ceased?: No

Causes understood?: Yes

Causes reversible?: No

Extreme fluctuations?: No

Population Information (Narrative): A decline in population size is inferred through decline in AOO due to human activities, such as urbanisation and agriculture, as well as overharvesting of specimens for the trade.

#### Subpopulations

Number of subpopulations: Unknown

Trend: Decline (inferred)

Justification for trend: Subpopulations are expected to be declining given the strong human pressure and severe fragmentation.

Extreme fluctuations?: No

Severe fragmentation?: Yes

Justification for fragmentation: Based on the analysis of the relative abundance of each species in low quality habitat (RA_LQH_) and the Species Distribution Model, 79.68% of the population should be in subpopulations that are non-viable and without the possibility of rescue effects due to fragmentation.

#### Habitat

System: Terrestrial

Habitat specialist: No

Habitat (narrative): *Brachypelma schroederi* inhabits dry deciduous forest and hillside scrub on the lower slopes of the Tlacolula valley (Valles Centrales) in subtropical central Oaxaca, enclosed by the Mixe Mountain Range (Sierra Norte, Sierra Sur etc.) and pine-oak forests at higher elevations (unpublished data).

Trend in extent, area or quality?: Decline (inferred)

Justification for trend: The habitat of this species is inferred to be declining in area, extent and quality due in particular to an increase in urbanisation, as well as non-industrial agriculture (e.g. corns, beans, sorghum, peanuts), as well as large-scale agricultural production of key crops in both the valley (especially wheat and peanuts) and hillsides (especially agave) which has destroyed the majority of the potential habitat.

**Figure 48.**
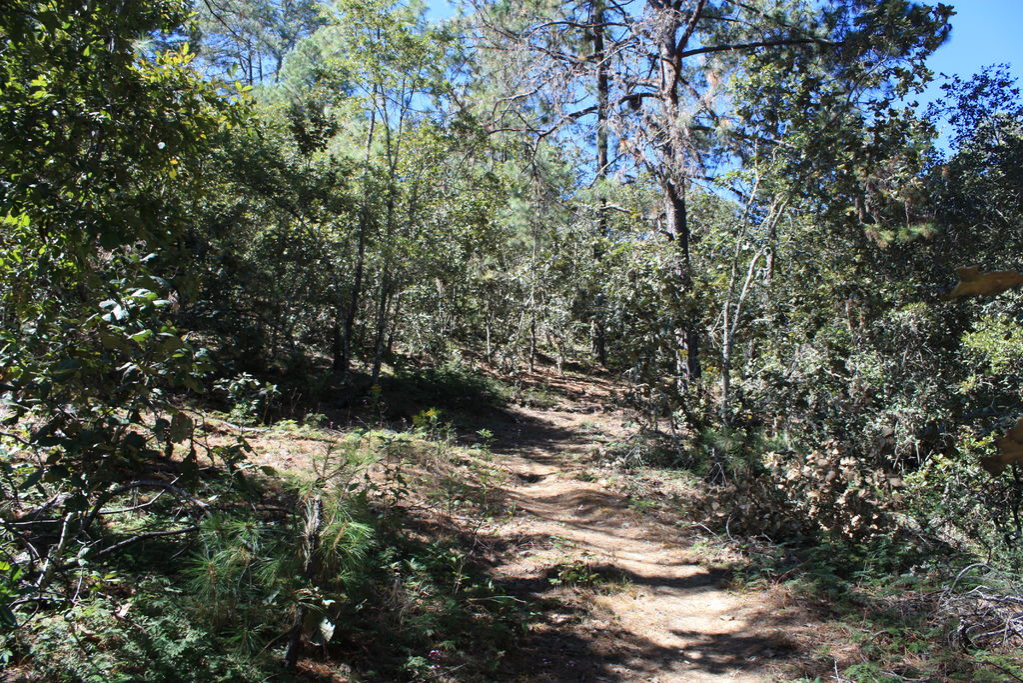
*Brachypelma schroederi* habitat, pine-oak forest, near Mitla, Oaxaca State, Mexico. Photo: Stuart J. Longhorn.

Figure(s) or Photo(s): Fig. 48

##### Habitat

Habitat importance: Major Importance

Habitats: 1.5. Forest - Subtropical/Tropical Dry

##### Habitat

Habitat importance: Marginal

Habitats: 3.5. Shrubland - Subtropical/Tropical Dry14.6. Artificial/Terrestrial - Subtropical/Tropical Heavily Degraded Former Forest

#### Habitat

Habitat importance: Major Importance

Habitats: 1.5. Forest - Subtropical/Tropical Dry

#### Habitat

Habitat importance: Marginal

Habitats: 3.5. Shrubland - Subtropical/Tropical Dry14.6. Artificial/Terrestrial - Subtropical/Tropical Heavily Degraded Former Forest

#### Ecology

Size: 65 mm (female); 55 mm (male)

Generation length (yr): 7

Dependency of single sp?: No

Ecology and traits (narrative): *Brachypelma schroederi* is a fossorial species that constructs or modifies burrows, often clearly defined obligate burrow-like retreats under debris, in particular, large rocks in various subtropical dry forests and scrub. The burrows slope steeply for several centimetres, ending in a chamber. Burrows will typically have some silk around the entrance to transmit the vibrations of prey movement and can be sealed further with a thin layer of silk across the diameter during daylight that may deter predators (e.g. ants, wasps etc.) and/or help maintain humidity inside the retreat. These spiders are nocturnal predators that wait near the entrance of their refuge from dusk and into the night to feed primarily on ground-dwelling arthropods (insects, other arachnids and some myriapods) or even small vertebrates. The mating season occurs during the last part of the rainy and first part of dry seasons (August to January) when mature males wander in the open to search for females. The males are likely most active at night, cooler daylight hours and throughout overcast days. Adult females typically moult once per year, just prior to the onset of the annual male emergence. Females will produce cocoons (large silken egg sacs) during the drier winter months with young emerging about two months later, with most young dispersing in the late spring or early summer, just before the onset of the early summer rains.

#### Threats

Justification for threats: There has been extensive human development of much of the former habitat for this species, including substantial deforestation and degradation of the remaining habitat. Urbanisation, livestock farming, as well as non-industrial agriculture and large-scale agricultural production of key crops in both the valley and hillsides, can be considered the major threats because they are the most probable cause of the severe fragmentation of subpopulations. Vehicular traffic on roads also jeopardise subpopulations, since males are killed by cars while crossing them during the mating season. Adding to this, the roads *per se* cause degradation of the natural habitat, but they also facilitate the access of smugglers to some areas, leading to a declining of wild populations. There is legal trade of captive-bred live specimens, but also an unknown amount of illegal trade of live animals, constituting a threat for subpopulations of *B. schroederi*. Adding to this, the population is declining due to human occupation of its natural habitat.

##### Threats

Threat type: Ongoing

Threats: 1.1. Residential & commercial development - Housing & urban areas2.2.1. Agriculture & aquaculture - Wood & pulp plantations - Small-holder plantations2.3.2. Agriculture & aquaculture - Livestock farming & ranching - Small-holder grazing, ranching or farming4.1. Transportation & service corridors - Roads & railroads5.1.1. Biological resource use - Hunting & trapping terrestrial animals - Intentional use (species is the target)

#### Threats

Threat type: Ongoing

Threats: 1.1. Residential & commercial development - Housing & urban areas2.2.1. Agriculture & aquaculture - Wood & pulp plantations - Small-holder plantations2.3.2. Agriculture & aquaculture - Livestock farming & ranching - Small-holder grazing, ranching or farming4.1. Transportation & service corridors - Roads & railroads5.1.1. Biological resource use - Hunting & trapping terrestrial animals - Intentional use (species is the target)

#### Conservation

Justification for conservation actions: Important conservation actions include protecting the natural habitat of *B. schroederi*, creating management plans and conducting systematic monitoring to provide information about the recovery of subpopulations. In Oaxaca State, conservation actions involving indigenous forestry organisations has proven to be an effective way of use and conservation of the natural habitat (Velázquez et al. 2003) and could be applied to *B. schroederi* management and recovery plan. Although the real occurrence of this species remains to be evaluated in it, Benito Juaréz National Park occurs into the range of the species and may be suitable for developing conservation initiatives. *Brachypelma schroederi* is being reared in captivity in Mexican Units for Management (UMAs) and sold legally. In order to avoid international trade incompatible with its survival, this species is currently listed on CITES Appendix II, along with all other species of the genus *Brachypelma* (CITES 2019). However, since there is an unknown amount of illegal pet trade of live animals, it is necessary to develop better enforcement actions to curb the illegal pet trade, as well as to establish tax advantages for legal dealers to rear this species in captivity and make their prices more competitive against the ones in the black market.

##### Conservation actions

Conservation action type: Needed

Conservation actions: 1.1. Land/water protection - Site/area protection3.4.1. Species management - Ex-situ conservation - Captive breeding/artificial propagation5.1.1. Law & policy - Legislation - International level

##### Conservation actions

Conservation action type: In Place

Conservation actions: 1.1. Land/water protection - Site/area protection1.2. Land/water protection - Resource & habitat protection3.1.1. Species management - Species management - Harvest management3.1.2. Species management - Species management - Trade management3.2. Species management - Species recovery5.4.1. Law & policy - Compliance and enforcement - International level5.4.2. Law & policy - Compliance and enforcement - National level5.4.3. Law & policy - Compliance and enforcement - Sub-national level6.4. Livelihood, economic & other incentives - Conservation payments

#### Conservation actions

Conservation action type: Needed

Conservation actions: 1.1. Land/water protection - Site/area protection3.4.1. Species management - Ex-situ conservation - Captive breeding/artificial propagation5.1.1. Law & policy - Legislation - International level

#### Conservation actions

Conservation action type: In Place

Conservation actions: 1.1. Land/water protection - Site/area protection1.2. Land/water protection - Resource & habitat protection3.1.1. Species management - Species management - Harvest management3.1.2. Species management - Species management - Trade management3.2. Species management - Species recovery5.4.1. Law & policy - Compliance and enforcement - International level5.4.2. Law & policy - Compliance and enforcement - National level5.4.3. Law & policy - Compliance and enforcement - Sub-national level6.4. Livelihood, economic & other incentives - Conservation payments

#### Other

Justification for use and trade: *Brachypelma schroederi* is being reared in captivity in Mexican Units for Management (UMAs) and sold legally. This species is currently listed on CITES Appendix II and thus its international trade is regulated by an international agreement (CITES 2019). A legal trade of captive-bred live specimens is recorded, but an unquantified amount of wild-caught animals illegally traded is also known. Captive-bred juveniles are sold for approximately US$30 in Canada and the United States, for US$17 in Mexico and for US$27 in the EU; adult males are sold for approximately US$250 in the United States and for US$28 in the EU; adult females are sold for approximately US$400 in the United States and for US$87 in the EU (CEC 2017). It is rare in trade but has a high demand. During 2006-2016, as only between 36–42 specimens of *B. schroederi* were traded internationally as live specimens traded for commercial purposes and none was declared as wild-caught, the species is considered uncommon in the international trade (Cooper et al. 2019).

##### Use and trade

Use type: International

Use and trade: 13. Pets/display animals, horticulture16. Establishing ex-situ production *

##### Ecosystem services

Ecosystem service type: Less important

##### Research needed

Research needed: 2.3. Conservation Planning - Harvest & Trade Management Plan3.1. Monitoring - Population trends3.2. Monitoring - Harvest level trends3.3. Monitoring - Trade trends

Justification for research needed: The government of Mexico should collaborate with Mexican tarantula breeders to develop a system for certifying the origin of specimens used in the breeding programmes of the Units for Management (UMAs). Additionally, systematic monitoring and protection could be undertaken in known subpopulations. Further studies are needed to confirm population, harvest and trade trends and how the latter affect harvest levels.

#### Use and trade

Use type: International

Use and trade: 13. Pets/display animals, horticulture16. Establishing ex-situ production *

#### Ecosystem services

Ecosystem service type: Less important

#### Research needed

Research needed: 2.3. Conservation Planning - Harvest & Trade Management Plan3.1. Monitoring - Population trends3.2. Monitoring - Harvest level trends3.3. Monitoring - Trade trends

Justification for research needed: The government of Mexico should collaborate with Mexican tarantula breeders to develop a system for certifying the origin of specimens used in the breeding programmes of the Units for Management (UMAs). Additionally, systematic monitoring and protection could be undertaken in known subpopulations. Further studies are needed to confirm population, harvest and trade trends and how the latter affect harvest levels.

#### Viability analysis

### Brachypelma smithi

#### Species information

Scientific name: Brachypelma smithi

Species authority: (F. O. Pickard-Cambridge, 1897)

Synonyms: *Eurypelma smithi* F. O. Pickard-Cambridge, 1897; *Euathlus smithi* (F. O. Pickard-Cambridge, 1897); *Avicularia smithi* (F. O. Pickard-Cambridge, 1897); *Brachypelma annitha* Tesmoingt, Cleton & Verdez, 1997.

Common names: Mexican redknee, tarántula mexicana de rodillas rojas, tarántula de anillos rojos, mygale à genoux rouges du Mexique.

Kingdom: Animalia

Phylum: Arthropoda

Class: Arachnida

Order: Araneae

Family: Theraphosidae

Taxonomic notes: CITES still records the synonym *Brachypelma annitha* as a valid species.

**Figure 49.**
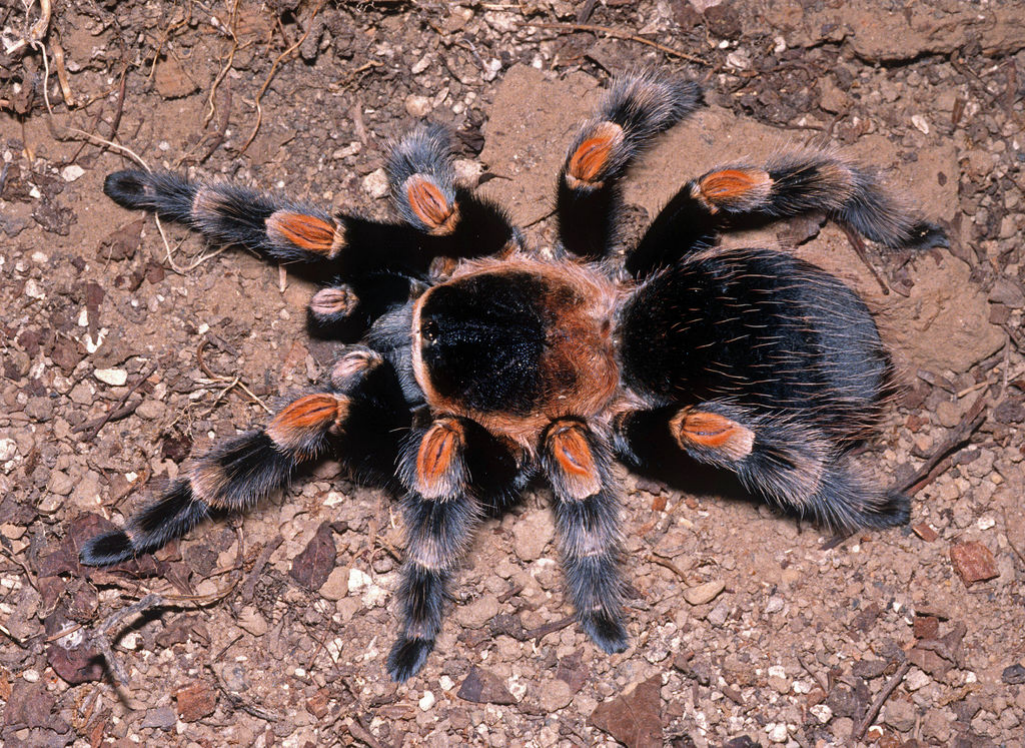
*Brachypelma smithi* (F. O. P.-Cambridge, 1897) female dark morphotype near Ixtapa, Guerrero state, Mexico. Photo: Rick C. West.

**Figure 50.**
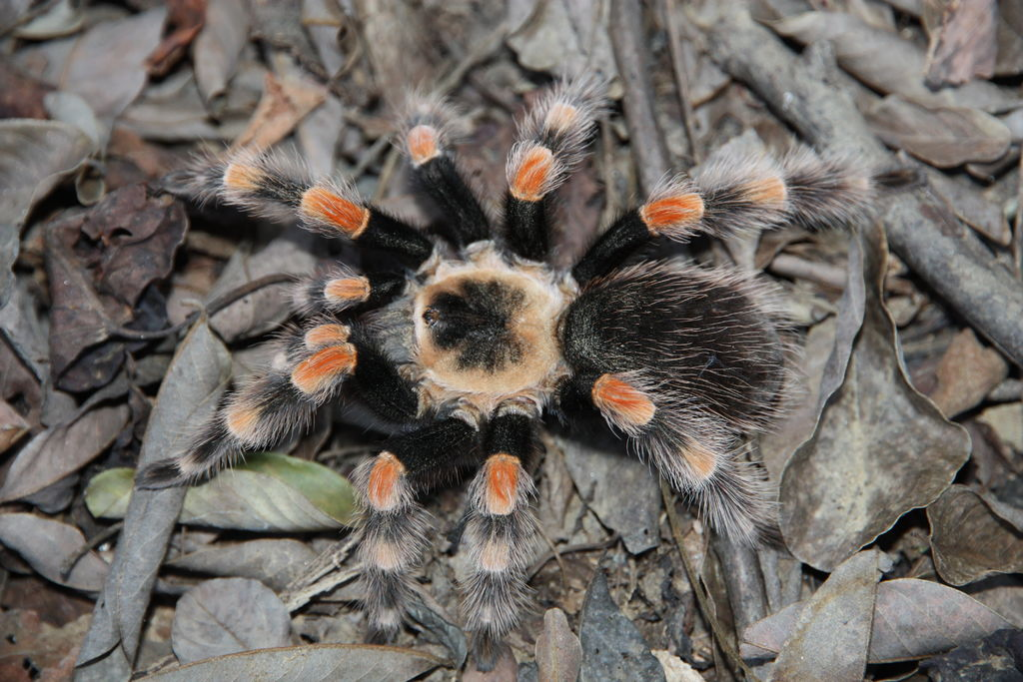
*Brachypelma smithi* (F. O. P.-Cambridge, 1897) female of morphotype at type locality from Dos Arroyos, Guerrero state, Mexico. Photo: Stuart J. Longhorn.

**Figure 51.**
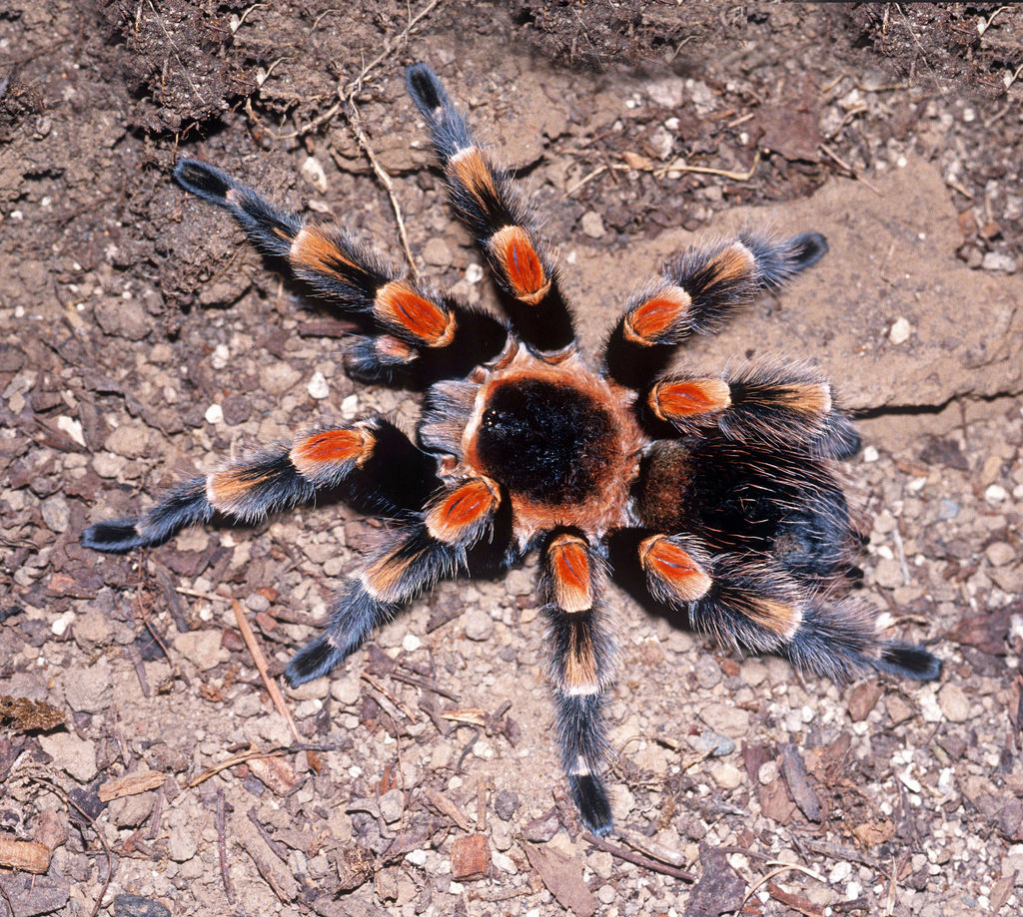
*Brachypelma smithi* (F.O.P.-Cambridge, 1987) male dark morphotype from north of Ixtapa, Guerrero state, Mexico. Photo: Rick C. West.

**Figure 52.**
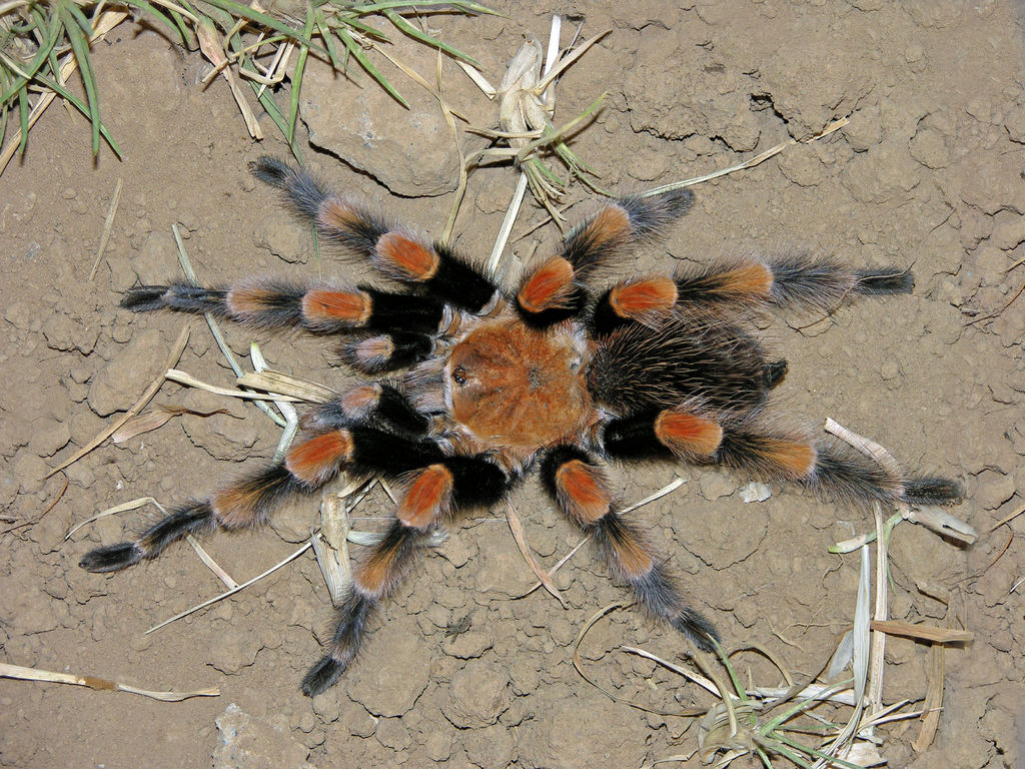
*Brachypelma smithi* (F. O. P.-Cambridge, 1897) male from Guerrero state, Mexico. Photo: Rick C. West.

Figure(s) or Photo(s): Figs 49, 50, 51, 52

Region for assessment: Global

#### Geographic range

Biogeographic realm: Neotropical

Countries: Mexico

**Figure 53.**
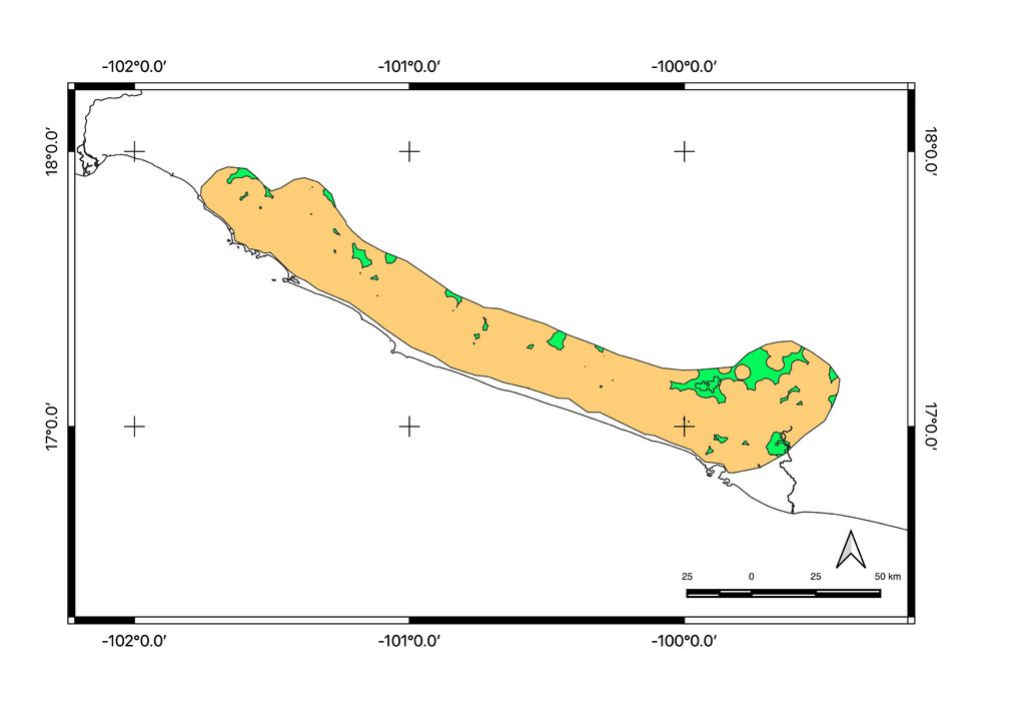
Habitat quality across the distribution of *Brachypelma smithi* (F. O. Pickard-Cambridge, 1897) Low quality habitat areas (LQH) in orange and high quality habitat areas (HQH) in green (for estimation details, see formula in Methods).

Map of records (image): Fig. 53

Map of records (Google Earth): Suppl. material 16

Basis of EOO and AOO: Species Distribution Model

Basis (narrative): A species distribution modelling has been performed to predict its potential range. See methods for details.

Min Elevation/Depth (m): 0

Max Elevation/Depth (m): 1500

Range description: *Brachypelma smithi* is endemic to Mexico and occurs along the Pacific coastal side of the Sierra Madre del Sur, east of the Balsas River Basin, to the Acapulco region in Guerrero State, being sympatric with *B. verdezi* in central Guerrero State, for much of the eastern range of *B. smithi* (Pickard-Cambridge 1897, Pocock 1903, Locht et al. 1999, CEC 2017, Mendoza and Francke 2017, iNaturalist 2018, unpublished data).

#### New occurrences

#### Extent of occurrence

EOO (km2): 11572

Trend: Decline (inferred)

Justification for trend: A decline in EOO is inferred, since there is loss of habitat area and quality due to human activities, such as urbanisation, agriculture and roads.

Causes ceased?: No

Causes understood?: Yes

Causes reversible?: No

Extreme fluctuations?: No

#### Area of occupancy

Trend: Decline (inferred)

Justification for trend: A decline in AOO is inferred, since there is loss of habitat area and quality due to human activities, such as urbanisation, agriculture and roads.

Causes ceased?: No

Causes understood?: Yes

Causes reversible?: No

Extreme fluctuations?: No

AOO (km2): 4692

#### Locations

Number of locations: 200

Justification for number of locations: Derived from the approximate number of human populations above 100 inhabitants needed to cover the entire range of the species. We assume each locality covers a mean radius of 2.5 km.

Trend: Decline (inferred)

Justification for trend: We assume the number of locations to be decreasing given the generalised loss of subpopulations of this species.

Extreme fluctuations?: No

#### Population

Number of individuals: Unknown

Trend: Decline (inferred)

Justification for trend: Despite no systematic research on populations of *B. smithi*, it is known that there is a loss of habitat, area and quality (Global Forest Watch 2019) due to human activities, such as urbanisation and agriculture. Adding to this, the species is suffering from overharvesting due to relatively intense trafficking (George Odell and Alejandro Alagon pers. comm.), especially in the Guerrero coastal area. Some populations in the highlands are depleted by locals and coastal populations are threatened by being run over by cars while crossing highways, since their range follows the main Pacific coastal highway. In the Acapulco vicinities, *B. smithi* has been collected to extinction. Populations are easily accessed by smugglers and there have already been cases of hundreds of specimens caught with a single smuggler. Adding to this, extensive flooding of the Papagayo River nearly caused the complete destruction of two *B. smithi* populations (Mendoza and Francke 2017).

Basis for decline: (c) a decline in area of occupancy, extent of occurrence and/or quality of habitat(d) actual or potential levels of exploitation

Causes ceased?: No

Causes understood?: Yes

Causes reversible?: Yes

Extreme fluctuations?: No

Population Information (Narrative): Despite no systematic research on populations of *B. smithi*, it is known that there is a loss of habitat, area and quality (Global Forest Watch 2019) due to human activities, such as urbanisation and agriculture. This species, along with the others of the red leg complex, are more fragile and do not adapt well or quickly to alterations in the environment caused by human activities. Adding to this, the species is suffering from overharvesting due to relatively intense trafficking, as well as for use in traditional medicine (George Odell and Alejandro Alagon pers. comm.), especially in the Guerrero coastal area. Some populations in the highlands are depleted by locals and coastal populations are threatened by being run over by cars while crossing highways, since their range follows the main Pacific coastal highway. In the Acapulco vicinities, *B. smithi* has been collected to extinction. Populations are easily accessed by smugglers and there have already been cases of hundreds of specimens caught with a single smuggler. Adding to this, extensive flooding of the Papagayo River nearly caused the complete destruction of two *B. smithi* populations (Mendoza and Francke 2017).

#### Subpopulations

Trend: Decline (inferred)

Justification for trend: Subpopulations are expected to be declining given the strong human pressure.

Extreme fluctuations?: No

Severe fragmentation?: Yes

Justification for fragmentation: Based on the analysis of the relative abundance of each species in low quality habitat (RA_LQH_) and the Species Distribution Model, 96.37% of the species lives in low quality and fragmented habitat.

#### Habitat

System: Terrestrial

Habitat specialist: Yes

Habitat (narrative): *Brachypelma smithi* inhabits the subtropical dry forest in the foothills of the Sierra Madre del Sur mountains, from the lowlands near the Pacific coast into slightly higher elevations further inland. This region is comprised mostly of thorn and deciduous secondary forest (Mendoza and Francke 2017), previously described as wooded shrubland and grassland, with some areas with intensive cultivation of maize (after Smith 1995). They appear to prefer undisturbed areas shaded by trees or bushes and can favour areas near seasonal watercourses.

Trend in extent, area or quality?: Decline (inferred)

Justification for trend: The habitat of this species is inferred to be declining in area, extent and quality due to human activities, such as urbanisation, agriculture and roads. Urban centres are rapidly expanding in much of its coastal zone, including rapid development of small coastal towns into major tourism centres. This has included major development of an extensive road infrastructure.

**Figure 54.**
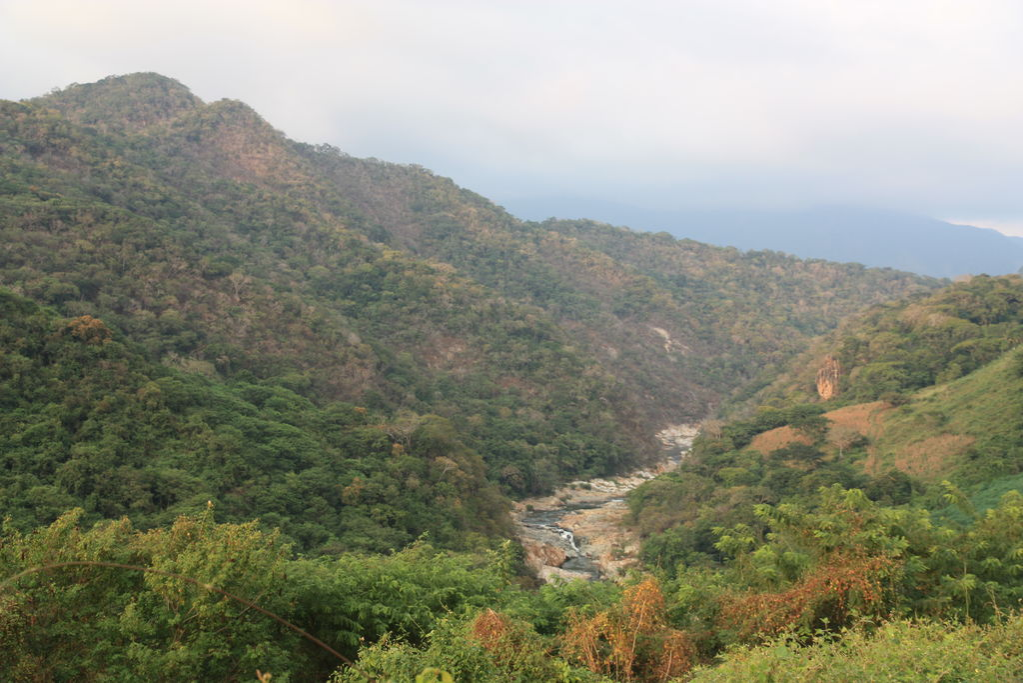
*Brachypelma smithi* habitat, subtropical deciduous forest, near Ixtapa, Guerrero state, Mexico. Photo: Stuart J. Longhorn

Figure(s) or Photo(s): Fig. 54

##### Habitat

Habitat importance: Major Importance

Habitats: 1.5. Forest - Subtropical/Tropical Dry

##### Habitat

Habitat importance: Suitable

Habitats: 14.3. Artificial/Terrestrial - Plantations

#### Habitat

Habitat importance: Major Importance

Habitats: 1.5. Forest - Subtropical/Tropical Dry

#### Habitat

Habitat importance: Suitable

Habitats: 14.3. Artificial/Terrestrial - Plantations

#### Ecology

Size: 70 mm (female); 60 mm (male).

Generation length (yr): 7

Dependency of single sp?: No

Ecology and traits (narrative): *Brachypelma smithi* is a fossorial species that modifies previously excavated burrows or can excavate their own unaided, often only making minor alterations to natural small cavities under debris, such as large rocks and tree roots in dense thickets or vegetation of dry thorn forests and deciduous forests (Mendoza and Francke 2017). The burrows have no traces of silk at the entrance giving no clear indication that there is a spider inside and the interior is often multi-tunnelled (Mendoza and Francke 2017). These spiders are nocturnal predators that wait near the entrance of their refuge from dusk and into the night to feed primarily on ground-dwelling arthropods (insects, other arachnids and some myriapods) or even small vertebrates. The mating season occurs during the last part of the rainy and first part of dry seasons (September to January) when mature males wander in the open to search for females. The males are likely most active at night, cooler daylight hours and throughout overcast days. Adult females typically moult once per year, just prior to the onset of the annual male emergence. Females will produce cocoons (large silken egg sacs) during the drier winter months with young emerging about two months later, with most young dispersing in the late spring (or early summer), just before the onset of the early summer rains (Mendoza and Francke 2017).

#### Threats

Justification for threats: Urbanisation and related transport infrastructure are rapidly expanding in the southern coastal region of the species range and either threaten or have destroyed several subpopulations. In particular, the port city of Acapulco is continuing to expand, developing new urban areas in the foothills, as well as expansion of several linked coastal towns often depending on tourism. *Brachypelma smithi*, along with the others of the red leg complex, are more fragile and do not adapt well or quickly to alterations in the environment caused by human activities (Turner et al. 2018). This species is also one of the most affected by overharvesting, due to the relatively intense trafficking, especially in the Pacific coastal area of Guerrero State (George Odell and Alejandro Alagon pers. comm.). Some populations in the highlands are depleted by locals and others are easily accessed by smugglers for the pet trade. There have already been cases of hundreds of specimens caught with a single smuggler. Vehicular traffic on roads can be considered another threat for populations along the north-south coastal Pacific highway, since males are killed by cars while crossing it during the mating season. Adding to this, roads facilitate the access of smugglers to wild populations and are also a source *per se* of natural habitat degradation. The increase in frequency of deleterious natural phenomena can also be considered as a threat since the coastal area of Guerrero State is subject to severe weather as well as flooding, such as the one that happened after the hurricane season of 2012-2013 and that almost totally wiped out some subpopulations of *B. smithi* (Mendoza and Francke 2017).

##### Threats

Threat type: Ongoing

Threats: 1.1. Residential & commercial development - Housing & urban areas2.1.2. Agriculture & aquaculture - Annual & perennial non-timber crops - Small-holder farming2.1.3. Agriculture & aquaculture - Annual & perennial non-timber crops - Agro-industry farming4.1. Transportation & service corridors - Roads & railroads5.1.1. Biological resource use - Hunting & trapping terrestrial animals - Intentional use (species is the target)11.4. Climate change & severe weather - Storms & flooding

#### Threats

Threat type: Ongoing

Threats: 1.1. Residential & commercial development - Housing & urban areas2.1.2. Agriculture & aquaculture - Annual & perennial non-timber crops - Small-holder farming2.1.3. Agriculture & aquaculture - Annual & perennial non-timber crops - Agro-industry farming4.1. Transportation & service corridors - Roads & railroads5.1.1. Biological resource use - Hunting & trapping terrestrial animals - Intentional use (species is the target)11.4. Climate change & severe weather - Storms & flooding

#### Conservation

Justification for conservation actions: In order to avoid international trade incompatible with its survival, this species is currently listed on CITES Appendix II, along with all other species of the genus *Brachypelma*, including its junior synonym *B. annitha*, mistakenly considered valid in CITES (CITES 2019). Adding to this, *Brachypelma smithi* is listed as a Threatened Species in Mexico. Despite this species being reared in captivity in Mexican Units for Management (UMAs) and sold legally, the illegal market has a high demand and the species is being overharvested mostly in the Guerrero coastal area. Thus, some areas are in need of protection, especially in the vicinities of Acapulco, where several subpopulations were collected to extinction. Allied to this, it is necessary to educate local populations not to collect these animals and also to protect them from smugglers. It is necessary to develop better enforcement actions to curb the illegal pet trade, as well as to establish tax advantages for legal dealers in order to make their prices more competitive with the ones in the black market. A Mexican Unit for Management is planning to release some specimens in the wild and re-introduce them in some areas where populations were severely depleted. Currently, this species is being reared in captivity in another UMA that promotes educational and awareness activities with tarantula spiders aimed at students and the general public. Adding to this, *B. smithi* can be used as a flagship species to motivate tarantula conservational efforts in Mexico, since it is a widely recognisable, very attractive species.

##### Conservation actions

Conservation action type: In Place

Conservation actions: 3.3.1. Species management - Species re-introduction - Reintroduction3.4.1. Species management - Ex-situ conservation - Captive breeding/artificial propagation5.1.1. Law & policy - Legislation - International level4.3. Education & awareness - Awareness & communications

##### Conservation actions

Conservation action type: Needed

Conservation actions: 1.1. Land/water protection - Site/area protection1.2. Land/water protection - Resource & habitat protection5.4.1. Law & policy - Compliance and enforcement - International level5.4.2. Law & policy - Compliance and enforcement - National level5.4.3. Law & policy - Compliance and enforcement - Sub-national level6.4. Livelihood, economic & other incentives - Conservation payments4.3. Education & awareness - Awareness & communications

#### Conservation actions

Conservation action type: In Place

Conservation actions: 3.3.1. Species management - Species re-introduction - Reintroduction3.4.1. Species management - Ex-situ conservation - Captive breeding/artificial propagation5.1.1. Law & policy - Legislation - International level4.3. Education & awareness - Awareness & communications

#### Conservation actions

Conservation action type: Needed

Conservation actions: 1.1. Land/water protection - Site/area protection1.2. Land/water protection - Resource & habitat protection5.4.1. Law & policy - Compliance and enforcement - International level5.4.2. Law & policy - Compliance and enforcement - National level5.4.3. Law & policy - Compliance and enforcement - Sub-national level6.4. Livelihood, economic & other incentives - Conservation payments4.3. Education & awareness - Awareness & communications

#### Other

Justification for use and trade: *Brachypelma smithi* is being reared in captivity in Mexican Units for Management (UMAs) and sold legally. This species is currently listed on CITES Appendix II (as well as its junior synonym *B. annitha*, that is still recognised by CITES as a valid species) and thus its international trade is regulated by an international agreement (CITES 2019). A legal trade of captive-bred live specimens is recorded, but an unquantified amount of wild-caught animals illegally traded is also known. Many specimens were taken from the wild due to the demand of what collectors call as ʺpure blood linesʺ. Unfortunately, the populations are easily accessed by smugglers, which increases overharvesting. Many specimens were sold under the name of *B. annitha.* Captive-bred juveniles have been sold for approximately US$40–$75 in Canada and the United States, for US$16 in Mexico and for US$8 in the EU (CEC 2017). Under the name *B. smithi*, captive-bred juveniles have been sold for approximately US$30–$35 in Canada and the United States, for US$10 in Mexico and for US$5 in the EU (CEC 2017). Adult males have been sold for approximately US$95 in Canada and for US$60 in the United States; adult females for approximately US$250 in Canada and the United States and for US$60 in the EU (CEC 2017). There is also an illegal pet trade to Asia, mostly to Japan and Hong Kong, but this species is present in large markets in Acapulco (Mexico), sold for use in traditional medicine (CEC 2017). Officially, *B. smithi* is a very common species on international trade: during 2006-2016, between 21,198–25,482 live specimens were traded internationally (Cooper et al. 2019). One hundred specimens exported from the USA to Canada were reported as being wild-caught and originating in Mexico, all others being reported as being captive-bred or captive-born, mostly for commercial purposes (Cooper et al. 2019). However, it is important to stress that it is likely that a significant number of these specimens were actually *B. hamorii* because, prior to the early 1980s, most (if not all) specimens called B. smithi were collected and exported from Colima State until that trade was restricted by the Mexican government. As discussed in the *B. hamorii* section, specimens from Colima State are now known to be *B. hamorii*. Thus, the values related to the trade of *B. smithi* should be viewed cautiously.

##### Use and trade

Use type: International

Use and trade: 3. Medicine - human & veterinary13. Pets/display animals, horticulture16. Establishing ex-situ production *

##### Ecosystem services

Ecosystem service type: Less important

##### Research needed

Research needed: 2.3. Conservation Planning - Harvest & Trade Management Plan3.1. Monitoring - Population trends3.2. Monitoring - Harvest level trends3.3. Monitoring - Trade trends

Justification for research needed: The government of Mexico should collaborate with Mexican tarantula breeders to develop a system for certifying the origin of specimens used in the breeding programmes of the Units for Management (UMAs). Additionally, systematic monitoring and protection could be undertaken in known subpopulations. Further studies are needed to confirm population, harvest and trade trends and how the latter affect harvest levels.

#### Use and trade

Use type: International

Use and trade: 3. Medicine - human & veterinary13. Pets/display animals, horticulture16. Establishing ex-situ production *

#### Ecosystem services

Ecosystem service type: Less important

#### Research needed

Research needed: 2.3. Conservation Planning - Harvest & Trade Management Plan3.1. Monitoring - Population trends3.2. Monitoring - Harvest level trends3.3. Monitoring - Trade trends

Justification for research needed: The government of Mexico should collaborate with Mexican tarantula breeders to develop a system for certifying the origin of specimens used in the breeding programmes of the Units for Management (UMAs). Additionally, systematic monitoring and protection could be undertaken in known subpopulations. Further studies are needed to confirm population, harvest and trade trends and how the latter affect harvest levels.

#### Viability analysis

### Brachypelma vagans

#### Species information

Scientific name: Brachypelma vagans

Species authority: (Ausserer, 1875)

Synonyms: *Eurypelma vagans* Ausserer, 1875; *Eurypelma dupontii* Becker, 1879; *Euathlus vagans* (Ausserer, 1875); *Avicularia vagans* (Ausserer, 1875).

Common names: Mexican redrump, tarántula mexicana de cadera roja, tarántula de terciopelo, tarántula de trasero rojo, mygale à croupion rouge du Mexique.

Kingdom: Animalia

Phylum: Arthropoda

Class: Arachnida

Order: Araneae

Family: Theraphosidae

Taxonomic notes: Mendonza and Francke (in press) are publishing a taxonomic revision of *Brachypelma* and this species will be accommodated in a new genus.

**Figure 55.**
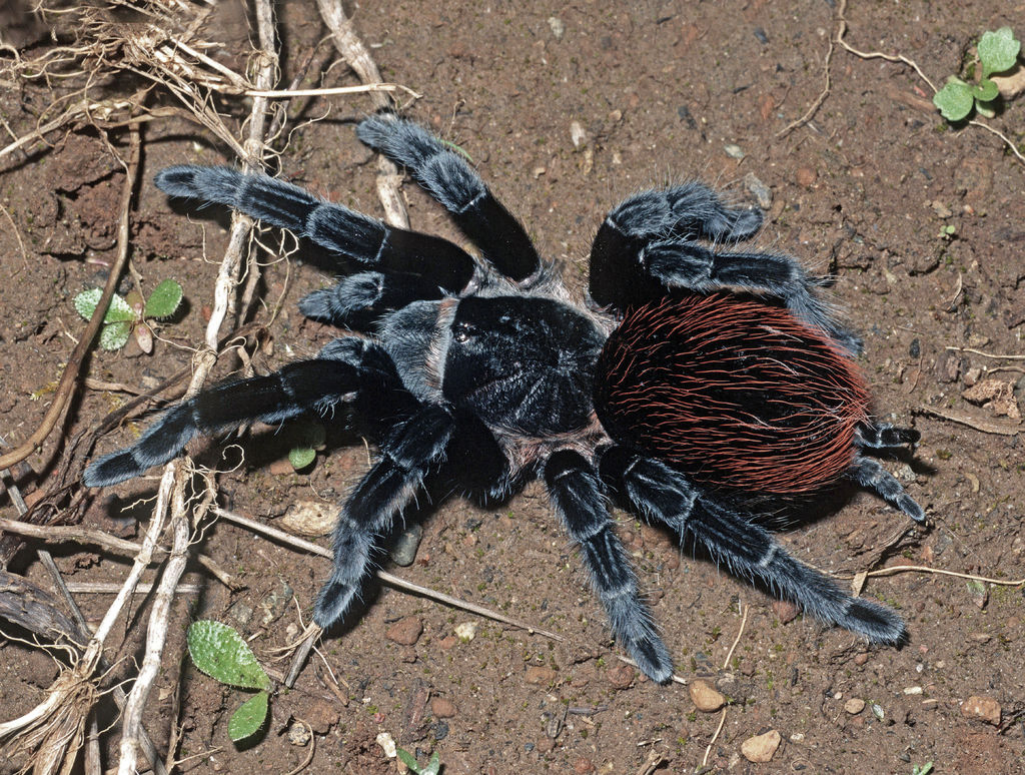
*Brachypelma vagans* (Ausserer, 1875) female from Orange Walk district, Belize. Photo: Rick C. West.

**Figure 56.**
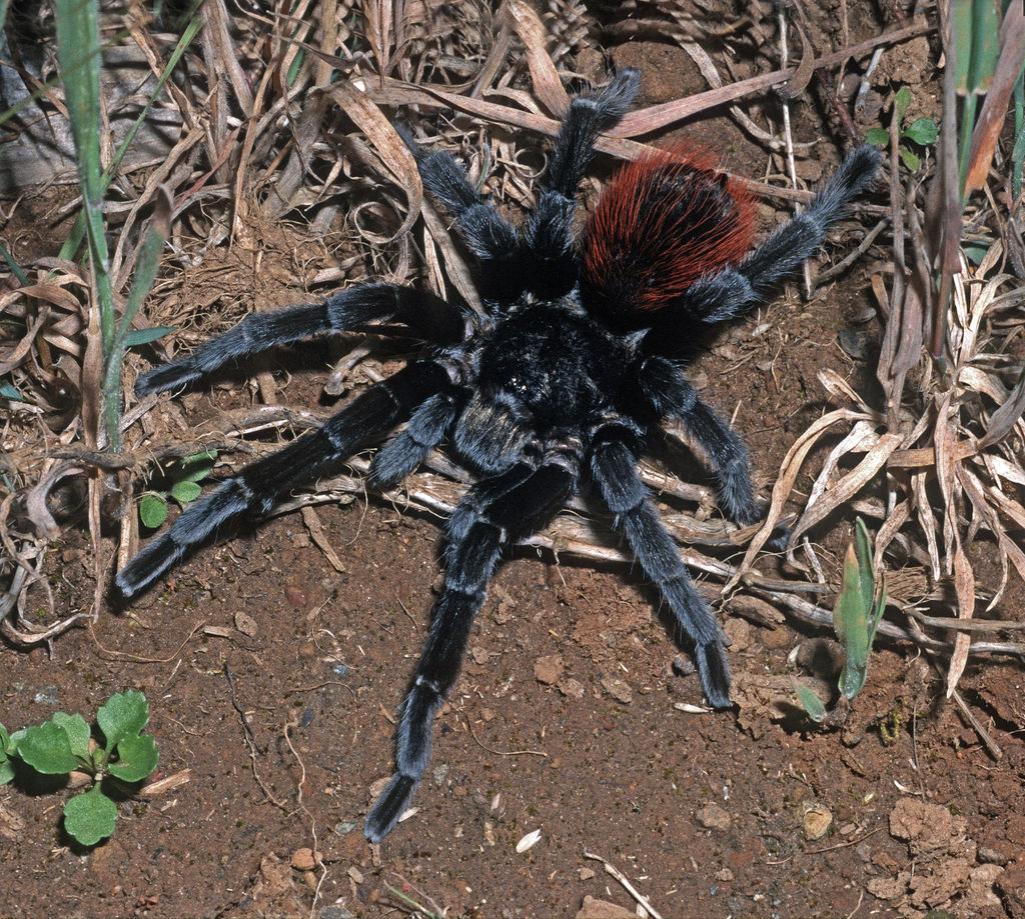
*Brachypelma vagans* (Ausserer, 1875) male from Orange Walk District, Belize. Photo: Rick C. West.

Figure(s) or Photo(s): Figs 55, 56

Region for assessment: Global

#### Geographic range

Biogeographic realm: Neotropical

Countries: GuatemalaBelizeMexico

**Figure 57.**
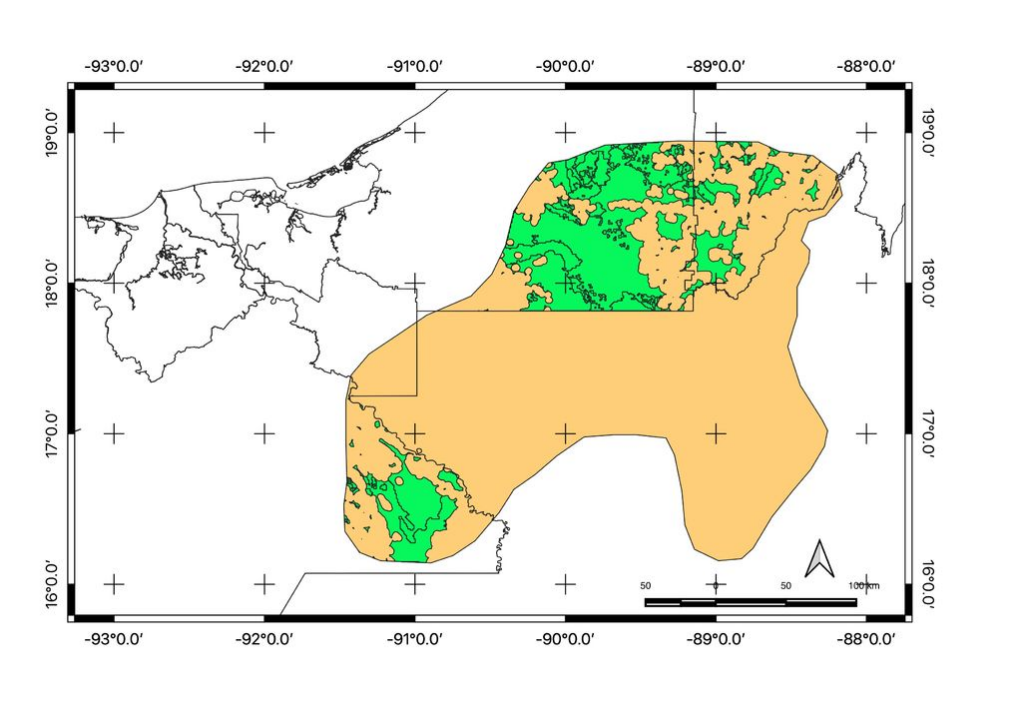
Habitat quality across the distribution of *Brachypelma vagans* (Ausserer, 1875). Low quality habitat areas (LQH) in orange and high quality habitat areas (HQH) in green (for estimation details, see formula in Methods).

Map of records (image): Fig. 57

Map of records (Google Earth): Suppl. material 17

Basis of EOO and AOO: Species Distribution Model

Basis (narrative): A species distribution modelling has been performed to predict its potential range. See methods for details.

Min Elevation/Depth (m): 0

Max Elevation/Depth (m): 1460

Range description: This species occurs in Belize, Guatemala and Mexico (Ausserer 1875, Pickard-Cambridge 1897, Locht et al. 1999, Yáñez and Floater 2000, Locht et al. 2005, Dor et al. 2008, Machkour-M’Rabet et al. 2012, CEC 2017, iNaturalist 2018, unpublished data). In Mexico, *B. vagans* occurs in southern Yucatán State, north-eastern Chiapas State and Quintana Roo State. In addition, there is a fairly widespread population of introduced *B. vagans* established in the St. Lucie County area of Florida, USA (Edwards and Hibbard 1999).

#### New occurrences

#### Extent of occurrence

EOO (km2): 121939

Trend: Decline (inferred)

Justification for trend: A decline in EOO is inferred, since there is habitat loss due to urbanisation and consequent deforestation.

Causes ceased?: No

Causes understood?: Yes

Causes reversible?: No

Extreme fluctuations?: No

#### Area of occupancy

Trend: Decline (inferred)

Justification for trend: A decline in AOO is inferred, since there is habitat loss due to urbanisation and consequent deforestation. Increasing frequency of flooding events might also be responsible for the recent loss in AOO.

Causes ceased?: No

Causes understood?: Yes

Causes reversible?: No

Extreme fluctuations?: No

AOO (km2): 73076

#### Locations

Number of locations: Unknown

Justification for number of locations: Given the large range, the number of locations is much above that of any category thresholds.

Trend: Unknown

Extreme fluctuations?: No

#### Population

Number of individuals: Unknown

Trend: Decline (inferred)

Justification for trend: The population size is inferred to be declining due to reduction in AOO and exploitation for the pet trade.

Basis for decline: (c) a decline in area of occupancy, extent of occurrence and/or quality of habitat(d) actual or potential levels of exploitation

Causes ceased?: No

Causes understood?: Yes

Causes reversible?: No

Extreme fluctuations?: No

Population Information (Narrative): *Brachypelma vagans* demonstrates invasive potential and has been reported in two invasive events: the first, taking place in Florida (Edwards and Hibbard 1999) and the second occurred on Cozumel Island in the Mexican Caribbean, as first reported by Machkour-M’Rabet et al. (2012). While this species is quite tolerant to some human changes in land use, some subpopulations are suffering with the illegal pet trade demand, others were negatively affected by extended periods of flooding which appears to have decimated several subpopulations.

#### Subpopulations

Number of subpopulations: Unknown

Trend: Decline (inferred)

Justification for trend: A decline in number of subpopulations is inferred, since it is being overharvested for the pet trade and traditional medicine and also subject to decline in AOO and quality of habitat due to human activities, such as urbanisation and increasing frequency of flooding events. The subpopulation situation has changed drastically in the last 15 years, especially in the Yucatan peninsula in Mexico, since males are now very rare and not spotted as numerously as before, probably due to more and larger roads and an explosion of human activity.

Extreme fluctuations?: No

Severe fragmentation?: Yes

Justification for fragmentation: Based on the analysis of the relative abundance of each species in low quality habitat (RA_LQH_) and the Species Distribution Model, 53% of the population should be in subpopulations that are non-viable and without the possibility of rescue effects due to fragmentation.

#### Habitat

System: Terrestrial

Habitat specialist: No

Habitat (narrative): *Brachypelma vagans* inhabits tropical and subtropical moist broadleaf forests of Mexico and adjacent countries. Some observations were made in the Yucatán peninsula, where a high density of their burrows can be associated with traditional human communities (Dor and Hénaut 2012). It was found that moderate levels of human disturbance can create favourable microhabitats for *B. vagans* (M'rabet et al. 2005). A reason is that these places correspond to areas of deep clay soil and absence of vegetation-roots, making it favourable for *B. vagans* specimens to excavate their burrows (M'rabet et al. 2005). At the sites studied in detail, M'rabet et al. (2005) also showed that different life stages occupied different microhabitats: females and spiderlings occupied backyards, whereas pre-adults occupied the edges of more exposed locations, such as football fields. They can also occupy open areas of pasture or non-intensive crops (without intense use of chemical pesticides).

Trend in extent, area or quality?: Decline (inferred)

Justification for trend: The habitat of this species is inferred to be declining in area, extent and quality due to human development through urbanisation.

**Figure 58.**
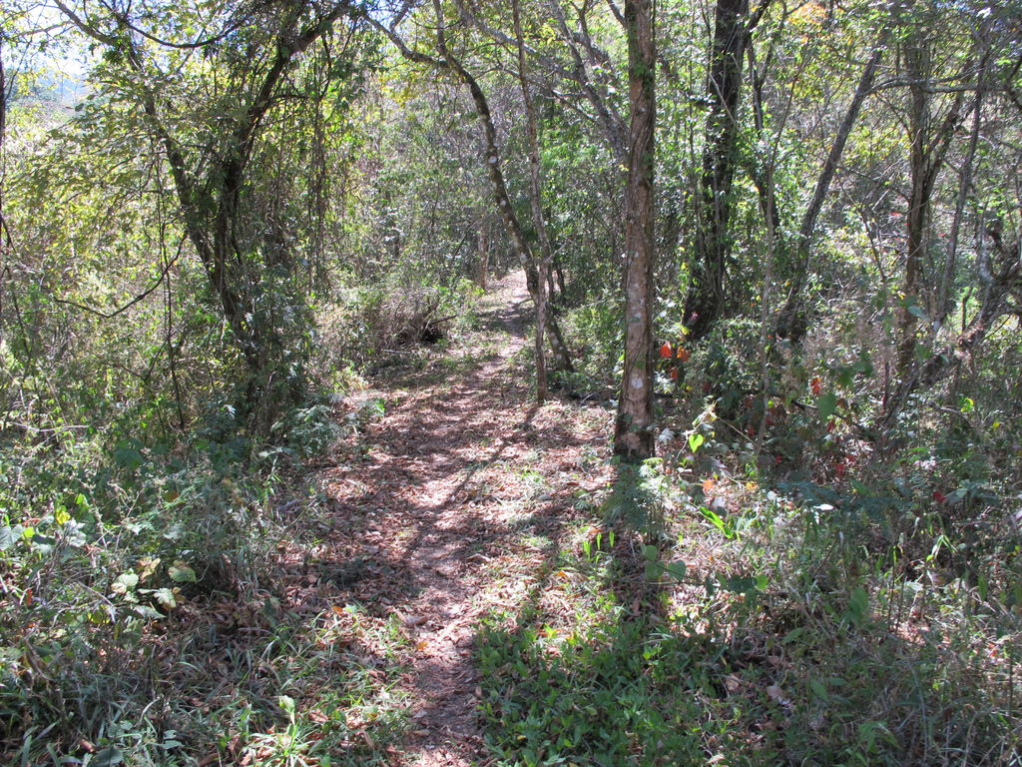
*Brachypelma vagans* habitat, Mexico. Photo: Jorge Mendoza.

Figure(s) or Photo(s): Fig. 58

##### Habitat

Habitat importance: Major Importance

Habitats: 1.6. Forest - Subtropical/Tropical Moist Lowland

##### Habitat

Habitat importance: Suitable

Habitats: 14.2. Artificial/Terrestrial - Pastureland14.3. Artificial/Terrestrial - Plantations14.6. Artificial/Terrestrial - Subtropical/Tropical Heavily Degraded Former Forest

#### Habitat

Habitat importance: Major Importance

Habitats: 1.6. Forest - Subtropical/Tropical Moist Lowland

#### Habitat

Habitat importance: Suitable

Habitats: 14.2. Artificial/Terrestrial - Pastureland14.3. Artificial/Terrestrial - Plantations14.6. Artificial/Terrestrial - Subtropical/Tropical Heavily Degraded Former Forest

#### Ecology

Size: 70 mm (female); 55 mm (male).

Generation length (yr): 7

Dependency of single sp?: No

Ecology and traits (narrative): This species along with *Brachypelma klaasi* is one of the better known for its ecological traits amongst *Brachypelma* species. *Brachypelma vagans* are a fossorial species that constructs or modifies burrows, often clearly defined obligate burrow-like retreats under debris, such as large rocks and tree roots in various tropical and subtropical habitats. The burrows slope steeply for several centimetres, ending in a chamber. Burrows will typically have a layer of silk around the entrance to transmit the vibrations of prey movement (Yáñez and Floater 2000) and can be sealed with a further thin layer of silk across the diameter during daylight that may deter predators (e.g. ants, wasps etc.) and/or help maintain humidity inside the retreat. These spiders are nocturnal predators that wait near the entrance of their refuge from dusk and into the night to feed primarily on ground-dwelling arthropods (insects, other arachnids and some myriapods) or even small vertebrates (Marshall 1996). The mating season is towards the end of the year, when mature males wander in the open to search for females. In Belize, this appearance of wandering adult males mostly occurs during August to October, but by December a few stragglers and late emergers can be found (Reichling 2003). The males are most active at night but may also be found roaming during the morning or late afternoon or at any time during overcast days (Reichling 2003). Adult females moult once per year, just prior to the onset of the annual male emergence. *Brachypelma vagans* females lay their eggs in April, but in southern Belize, some females have made cocoons (large silken egg sacs) by the middle of February. Most adult females. observed during April and May. are tending cocoons, which can be 4–5 centimetres in diameter containing around 300 young and spiderlings stay with the mother for several weeks before they disperse (Moore 1994). Spiderlings may form a line during the dispersion, walking as ants in a column (Reichling 2000, Reichling 2003). Regarding interactions of *B. vagans* populations with human activities, it was found that the density of tarantulas decreased with increasing human activity (M'rabet et al. 2005). Surprisingly, it was found that *B. vagans* was absent both in mature and secondary forests in areas where it originally occurred, suggesting that, in natural conditions, this species probably uses clearings in the forest and that it can be present in high densities in rural villages with low levels of anthropogenic disturbance close to medium semi-evergreen forests (M'rabet et al. 2005). Specimens of *Brachypelma vagans* probably from the pet trade have also colonised central Florida, an area with soil type, vegetation type and climate very similar to its natural habitat in the Yucatán Peninsula in Mexico, indicating the high adaptability of this species (Edwards and Hibbard 1999). Furthermore, this species is considered a potential predator of *Centruroides* spp., i.e. scorpions that live in peri-domestic areas in Yucatán, where they represent a real health problem (Dor et al. 2011) and also a known predator the cockroach *Periplaneta americana* (Linnaeus, 1758), a synanthropic cosmopolitan urban pest. The association between tarantulas and the human community in Yucatán is not only ecological but also cultural, with the use of those spiders in traditional medicine (Machkour-M’Rabet et al. 2011). Another peculiarity about this species is that there is a cultural association with the Ch’ol, a Mayan ethnic group from Chiapas and Campeche States, since they use specimens of *B. vagans* in traditional medicine (Machkour-M’Rabet et al. 2011).

#### Threats

Justification for threats: The natural habitat of *B. vagans* has undergone one of the world’s highest deforestation rates during the past 20 years (Mittermeier RA et al. 1999), creating a landscape mosaic of secondary vegetation, agriculture and urban or rural areas (M'rabet et al. 2005). The habitat loss due to deforestation and extended periods of flooding decimated populations in some areas. Low genetic diversity in *B. vagans* was also detected due to the reduced number of individuals moving between different populations (Machkour-M’Rabet et al. 2012). There is some trade and specimens are harvested by local children.

##### Threats

Threat type: Ongoing

Threats: 1.1. Residential & commercial development - Housing & urban areas2.1.2. Agriculture & aquaculture - Annual & perennial non-timber crops - Small-holder farming2.1.3. Agriculture & aquaculture - Annual & perennial non-timber crops - Agro-industry farming5.1.1. Biological resource use - Hunting & trapping terrestrial animals - Intentional use (species is the target)11.4. Climate change & severe weather - Storms & flooding

#### Threats

Threat type: Ongoing

Threats: 1.1. Residential & commercial development - Housing & urban areas2.1.2. Agriculture & aquaculture - Annual & perennial non-timber crops - Small-holder farming2.1.3. Agriculture & aquaculture - Annual & perennial non-timber crops - Agro-industry farming5.1.1. Biological resource use - Hunting & trapping terrestrial animals - Intentional use (species is the target)11.4. Climate change & severe weather - Storms & flooding

#### Conservation

Justification for conservation actions: The species occurs in some protected areas, such as the Mexican Calakmul Biosphere Reserve and the Belize Chiquibul National Park, areas that can be suitable for developing conservation initiatives. As *B. vagans* may rapidly become extinct in states where their numbers are so low (Hénaut et al. 2015), there is a clear need for this species to receive adequate protection and management plans. The large populations in Campeche need to be provided with special protection as they represent important centres of dispersal and resilience for *B. vagans* (Hénaut et al. 2015). The higher number of specimens may be due to the proximity to a large protected area and further research may help us understand the importance of these areas for the conservation of the Mexican redrump tarantula (Hénaut et al. 2015). The fact that these animals can be considered as predator of a peri-domestic scorpion that causes problems to human health, as well of an urban pest can be used to stimulate the establishment of management plans for *B. vagans*. In order to avoid international trade incompatible with its survival, this species is currently listed on CITES Appendix II, along with all other species of the genus *Brachypelma* (CITES 2019). *Brachypelma vagans* is being reared in captivity in Mexican Units for Management (UMAs) and sold legally. However, as this species is in the illegal trade, it is necessary to develop better enforcement actions to curb trafficking, as well as tax advantages for legal dealers to make their prices more competitive with the ones in the black market. Currently, this species is being reared in captivity in a UMA that also promotes educational and awareness activities regarding tarantula spiders aimed at students and the general public.

##### Conservation actions

Conservation action type: In Place

Conservation actions: 1.1. Land/water protection - Site/area protection3.4.1. Species management - Ex-situ conservation - Captive breeding/artificial propagation5.1.1. Law & policy - Legislation - International level4.3. Education & awareness - Awareness & communications

##### Conservation actions

Conservation action type: Needed

Conservation actions: 1.2. Land/water protection - Resource & habitat protection2.3. Land/water management - Habitat & natural process restoration5.4.1. Law & policy - Compliance and enforcement - International level5.4.2. Law & policy - Compliance and enforcement - National level5.4.3. Law & policy - Compliance and enforcement - Sub-national level6.4. Livelihood, economic & other incentives - Conservation payments4.3. Education & awareness - Awareness & communications

#### Conservation actions

Conservation action type: In Place

Conservation actions: 1.1. Land/water protection - Site/area protection3.4.1. Species management - Ex-situ conservation - Captive breeding/artificial propagation5.1.1. Law & policy - Legislation - International level4.3. Education & awareness - Awareness & communications

#### Conservation actions

Conservation action type: Needed

Conservation actions: 1.2. Land/water protection - Resource & habitat protection2.3. Land/water management - Habitat & natural process restoration5.4.1. Law & policy - Compliance and enforcement - International level5.4.2. Law & policy - Compliance and enforcement - National level5.4.3. Law & policy - Compliance and enforcement - Sub-national level6.4. Livelihood, economic & other incentives - Conservation payments4.3. Education & awareness - Awareness & communications

#### Other

Justification for use and trade: *Brachypelma vagans* is being reared in captivity in Mexican Units for Management (UMA) and sold legally. This species is currently listed on CITES Appendix II and thus its international trade is regulated by an international agreement (CITES 2019). A legal trade of captive-bred live animals has been recorded, but an unquantified amount of wild-caught animals illegally traded is also known. However, this seems to be of limited extent and stable for now due, in part, to the relative ease of breeding this species in captivity by hobbyists. Captive-bred juveniles are currently sold for approximately US$10 in Canada and the United States, for US$10 in Mexico and for US$5 in the EU (CEC 2017). Adult males are sold for approximately US$50 in Canada and for US$35 in the United States; adult females for approximately US$100–$130 in Canada and the United States and for US$60 in the EU (CEC 2017). During 2006-2016, between 875–1,072 live specimens of *B. vagans* were traded internationally, all but two reported as being captive-bred or captive-born, mostly for commercial purposes, being considered a common species in the international trade (Cooper et al. 2019). An additional 210 wild-caught specimens of *B. vagans* were exported from Mexico to France for scientific purposes, although this trade was not recorded by the importing country.

##### Use and trade

Use type: International

Use and trade: 3. Medicine - human & veterinary13. Pets/display animals, horticulture16. Establishing ex-situ production *

##### Ecosystem services

Ecosystem service type: Less important

##### Research needed

Research needed: 2.3. Conservation Planning - Harvest & Trade Management Plan3.1. Monitoring - Population trends3.2. Monitoring - Harvest level trends3.3. Monitoring - Trade trends

Justification for research needed: *Brachypelma vagans* has possibly the best known ecological traits of any *Brachypelma* species. Therefore, more advanced studies are necessary at this point, such as investigations about genetic profile and flow amongst different populations. The government of Mexico could collaborate with Mexican tarantula breeders to develop a system for certifying the origin of specimens used in the breeding programmes of the Units for Management (UMAs). Systematic monitoring and protection could be undertaken within known subpopulations. Trade trends and how these affect harvest levels must be further studied.

#### Use and trade

Use type: International

Use and trade: 3. Medicine - human & veterinary13. Pets/display animals, horticulture16. Establishing ex-situ production *

#### Ecosystem services

Ecosystem service type: Less important

#### Research needed

Research needed: 2.3. Conservation Planning - Harvest & Trade Management Plan3.1. Monitoring - Population trends3.2. Monitoring - Harvest level trends3.3. Monitoring - Trade trends

Justification for research needed: *Brachypelma vagans* has possibly the best known ecological traits of any *Brachypelma* species. Therefore, more advanced studies are necessary at this point, such as investigations about genetic profile and flow amongst different populations. The government of Mexico could collaborate with Mexican tarantula breeders to develop a system for certifying the origin of specimens used in the breeding programmes of the Units for Management (UMAs). Systematic monitoring and protection could be undertaken within known subpopulations. Trade trends and how these affect harvest levels must be further studied.

#### Viability analysis

### Brachypelma verdezi

#### Species information

Scientific name: Brachypelma verdezi

Species authority: Schmidt, 2003

Common names: Mexican rosegrey, tarántula mexicana rosa-gris, mygale rose-gris mexicaine.

Kingdom: Animalia

Phylum: Arthropoda

Class: Arachnida

Order: Araneae

Family: Theraphosidae

Taxonomic notes: Mendonza and Francke (in press) are publishing a taxonomic revision of *Brachypelma* and this species will be accommodated in a new genus.

**Figure 59.**
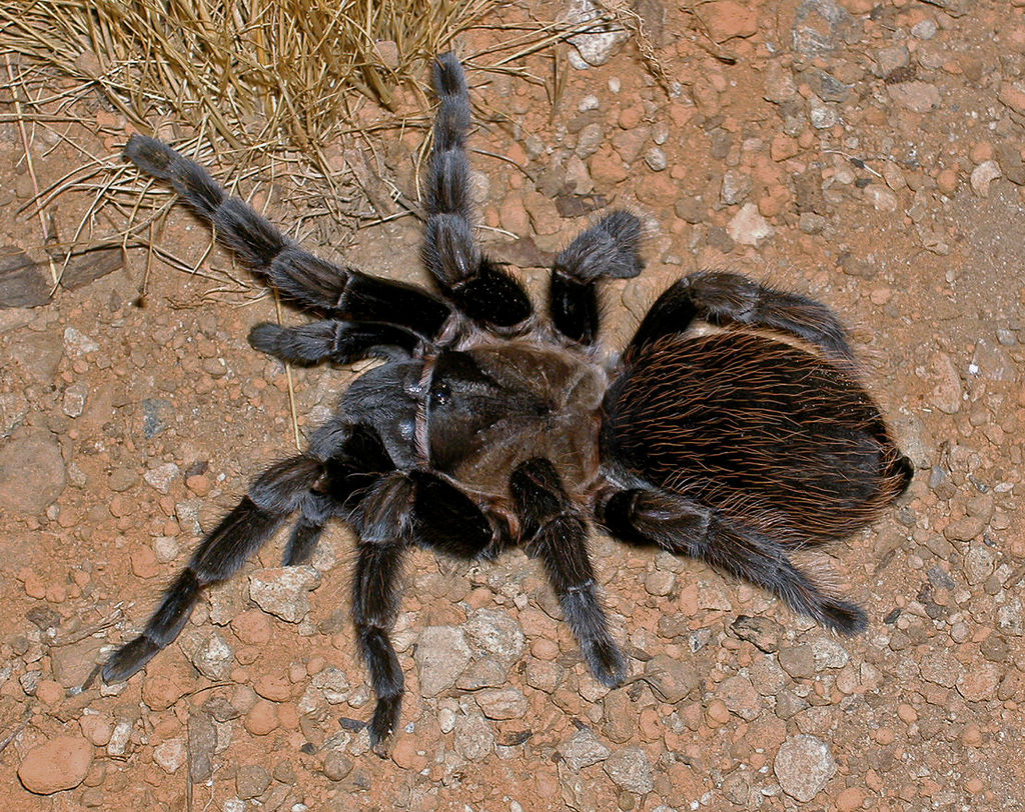
*Brachypelma verdezi* Schmidt, 2003 female from Chilpancingo, Guerrero state, Mexico. Photo: Rick C. West.

**Figure 60.**
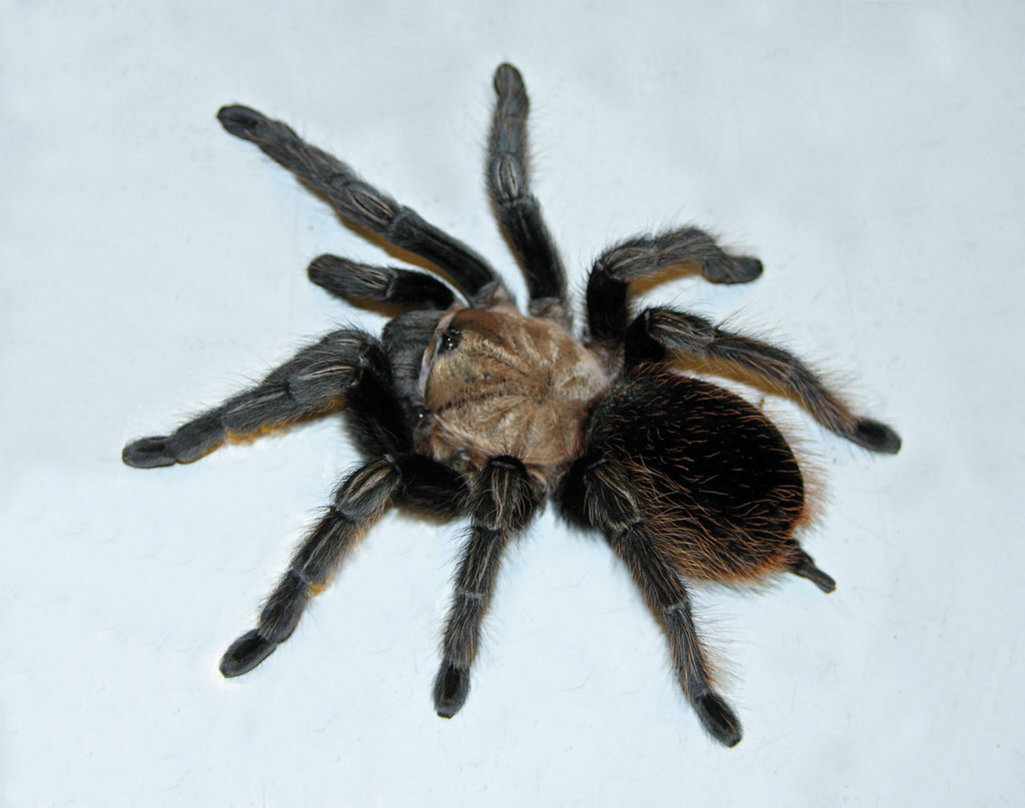
*Brachypelma verdezi* Schmidt, 2003 female, light morphotype from near Chilpancingo, Guerrero state, Mexico. Photo: Rick C. West.

**Figure 61.**
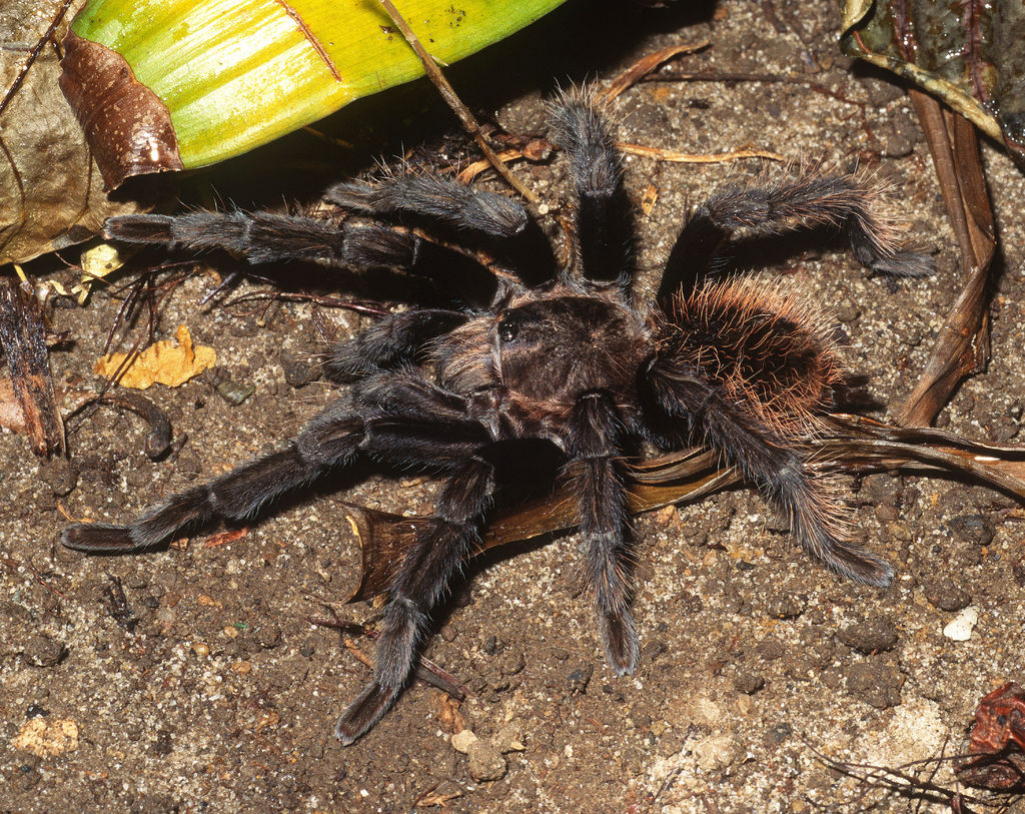
*Brachypelma verdezi* Schmidt, 2003 male from Chilpancingo area, Guerrero state, Mexico. Photo: Rick C. West.

Figure(s) or Photo(s): Figs 59, 60, 61

Region for assessment: Global

#### Geographic range

Biogeographic realm: Neotropical

Countries: Mexico

**Figure 62.**
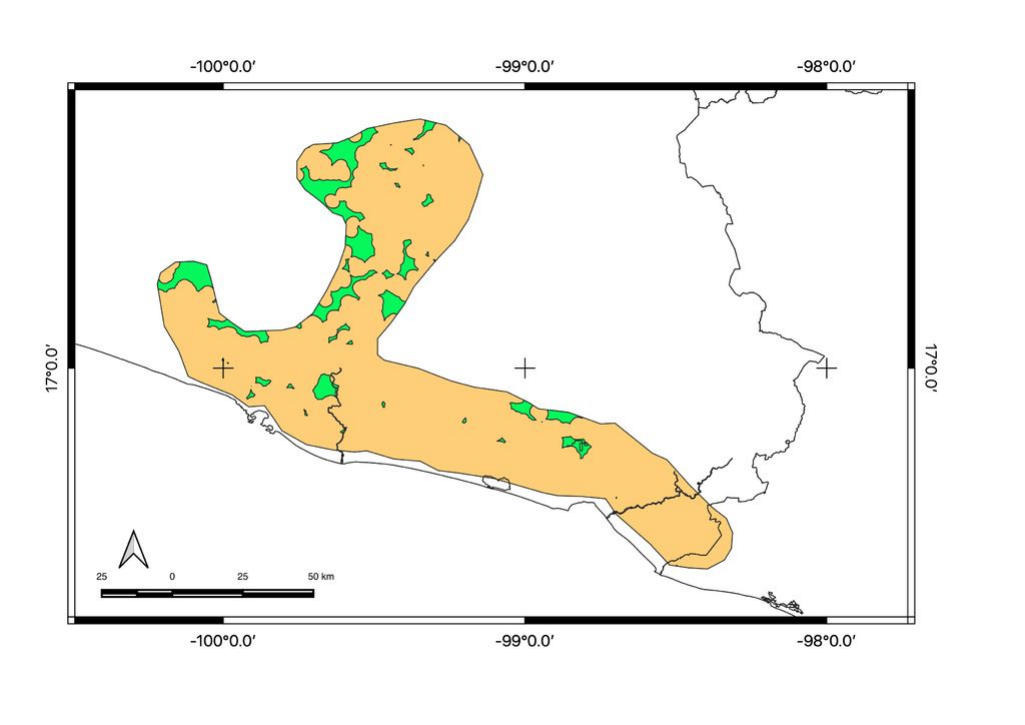
Habitat quality across the distribution of *Brachypelma verdezi* Schmidt, 2003. Low quality habitat areas (LQH) in orange and high quality habitat areas (HQH) in green (for estimation details, see formula in Methods).

Map of records (image): Fig. 62

Map of records (Google Earth): Suppl. material 18

Basis of EOO and AOO: Species Distribution Model

Basis (narrative): Despite few collection sites recorded for this species, it was possible to perform species distribution modelling to predict its potential range. See methods for details.

Min Elevation/Depth (m): 0

Max Elevation/Depth (m): 2050

Range description: *Brachypelma verdezi* is endemic to Mexico and primarily occurs in the Sierra Madre del Sur region around Chilpancingo, Guerrero State. *B. verdezi* is sympatric in Guerrero State with other *Brachypelma* species such as *B. smithi* (in Central Guerrero State, for much of the westernmost extent of *B. verdezi*) and *B. albiceps* (in Guerrero State, only inland at the northernmost known limits of *B. verdezi*) ([Bibr B4348193][Bibr B4348203], CEC 2017, iNaturalist 2018, unpublished data).

#### New occurrences

#### Extent of occurrence

EOO (km2): 15312

Trend: Decline (inferred)

Justification for trend: The species habitat is being lost due to urbanisation and agriculture, possibly affecting the EOO.

Causes ceased?: No

Causes understood?: Yes

Causes reversible?: No

Extreme fluctuations?: No

#### Area of occupancy

Trend: Decline (inferred)

Justification for trend: The species habitat is being lost due to urbanisation and agriculture, affecting the AOO.

Causes ceased?: No

Causes understood?: Yes

Causes reversible?: No

Extreme fluctuations?: No

AOO (km2): 7488

#### Locations

Number of locations: Unknown

Justification for number of locations: The number of locations is far above any thresholds.

Trend: Decline (inferred)

Justification for trend: Inferred from a decrease in the number of subpopulations.

Extreme fluctuations?: No

#### Population

Number of individuals: Unknown

Trend: Decline (inferred)

Justification for trend: A decline in population size is inferred, given the decrease in AOO. Harvesting adds to this reduction, since there is an unknown amount of trafficking of wild-caught animals.

Basis for decline: (c) a decline in area of occupancy, extent of occurrence and/or quality of habitat(d) actual or potential levels of exploitation

Causes ceased?: No

Causes understood?: Yes

Causes reversible?: No

Extreme fluctuations?: No

Population Information (Narrative): A decline in population size is inferred, given the decrease in AOO. Harvesting adds to this reduction, since there is an unknown amount of illegal pet trade of wild-caught animals.

#### Subpopulations

Number of subpopulations: Unknown

Trend: Decline (inferred)

Justification for trend: The number of subpopulations is expected to be declining, given the strong human pressure.

Extreme fluctuations?: No

Severe fragmentation?: Yes

Justification for fragmentation: Based on the analysis of the relative abundance of each species in low quality habitat (RA_LQH_) and the Species Distribution Model, 94.2% of the population should be in subpopulations that are non-viable and without the possibility of rescue effects due to fragmentation.

#### Habitat

System: Terrestrial

Habitat specialist: No

Habitat (narrative): *Brachypelma verdezi* inhabits the subtropical dry forest in the foothills of the Sierra Madre del Sur mountains, from the lowlands near the Pacific coast into higher elevations further inland. This region is mostly covered by thorn and deciduous secondary forests, scrubland and grassland, with some areas with intensive cultivation of maize. They appear to be relatively tolerant of human-disturbance and can adapt to either forming burrows or a slightly more vagrant lifestyle under rocks or other debris in more open areas around livestock pasture and areas of non-intensive crops (without intense use of chemical pesticides).

Trend in extent, area or quality?: Decline (inferred)

Justification for trend: The habitat of this species is inferred to be declining in area, extent and quality due to human activities, such as urbanisation and agriculture. Urban centres are rapidly expanding in much of its coastal zone, including rapid development of small coastal towns into major tourism centres.

**Figure 63.**
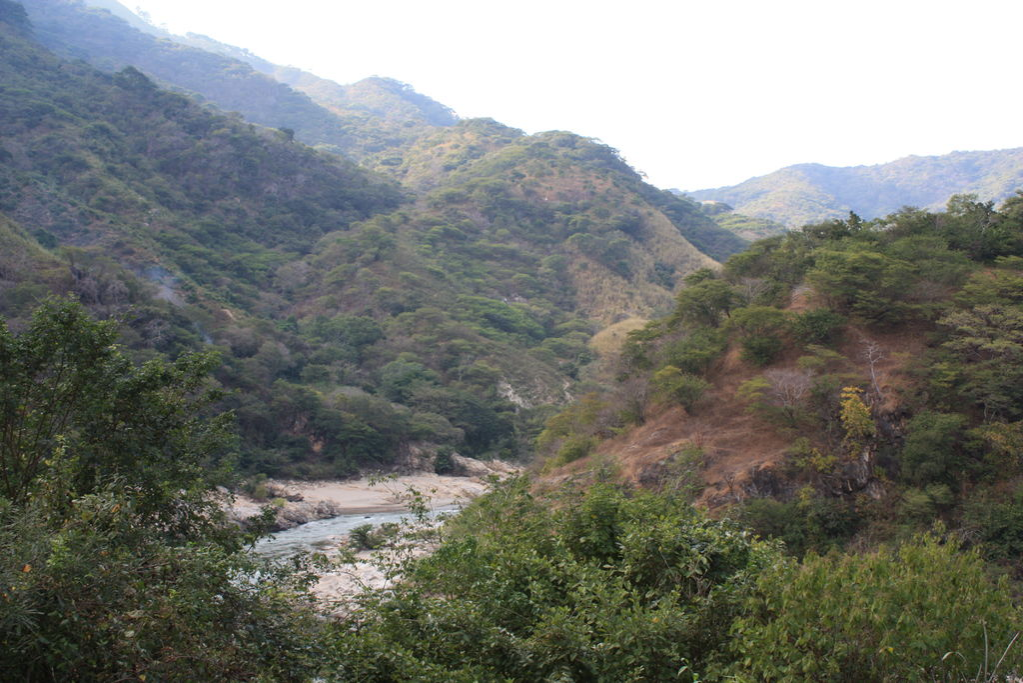
*Brachypelma verdezi* habitat, subtropical dry forest and scrubland, near Acapulco, Guerrero state, Mexico. Photo: Stuart J. Longhorn.

Figure(s) or Photo(s): Fig. 63

##### Habitat

Habitat importance: Major Importance

Habitats: 1.5. Forest - Subtropical/Tropical Dry

##### Habitat

Habitat importance: Marginal

Habitats: 14.2. Artificial/Terrestrial - Pastureland14.6. Artificial/Terrestrial - Subtropical/Tropical Heavily Degraded Former Forest

#### Habitat

Habitat importance: Major Importance

Habitats: 1.5. Forest - Subtropical/Tropical Dry

#### Habitat

Habitat importance: Marginal

Habitats: 14.2. Artificial/Terrestrial - Pastureland14.6. Artificial/Terrestrial - Subtropical/Tropical Heavily Degraded Former Forest

#### Ecology

Size: 65 mm (female); 55 mm (male).

Generation length (yr): 7

Dependency of single sp?: No

Ecology and traits (narrative): *Brachypelma verdezi* is a fossorial species that constructs or modifies burrows, often clearly defined obligate burrow-like retreats under debris, such as large rocks and tree roots in subtropical dry forest. The burrows slope steeply for several centimetres, ending in a chamber. Burrows will typically have a layer of silk around the entrance to transmit the vibrations of prey movement and can be sealed with a further thin layer of silk across the diameter during daylight that may deter predators (e.g. ants, wasps etc.) and/or help maintain humidity inside the retreat. These spiders are nocturnal predators that wait near the entrance of their refuge from dusk and into the night to feed primarily on ground-dwelling arthropods (insects, other arachnids and some myriapods) or even small vertebrates. The mating season occurs during the last part of the rainy and first part of dry seasons (August to January) when mature males wander in the open to search for females. The males are likely most active at night, cooler daylight hours and throughout overcast days. Adult females typically moult once per year, just prior to the onset of the annual male emergence. Females will produce cocoons (large silken egg sacs) during the drier winter months with young emerging about two months later, with most young dispersing in the late spring or early summer, just before the onset of the early summer rains.

#### Threats

Justification for threats: Habitat loss due to deforestation (Global Forest Watch 2019) is considered as an important threat. Urbanisation and related transport infrastructure are rapidly expanding in the southern coastal region of its range and either threaten or have destroyed several subpopulations. In particular, the port city of Acapulco is continuing to expand, developing new urban areas in the foothills, as well as expansion of several linked coastal towns often dependent on tourism. Much of the development is linked to increases in small-scale agriculture (e.g. corn and beans), further destroying available habitat. There is legal trade of captive-bred live specimens, but also an unknown amount of illegal pet trade of live animals. Roads facilitate the access of smugglers to wild populations and are also a source *per se* of habitat degradation.

##### Threats

Threat type: Ongoing

Threats: 1.1. Residential & commercial development - Housing & urban areas2.1.2. Agriculture & aquaculture - Annual & perennial non-timber crops - Small-holder farming2.1.3. Agriculture & aquaculture - Annual & perennial non-timber crops - Agro-industry farming4.1. Transportation & service corridors - Roads & railroads5.1.1. Biological resource use - Hunting & trapping terrestrial animals - Intentional use (species is the target)

#### Threats

Threat type: Ongoing

Threats: 1.1. Residential & commercial development - Housing & urban areas2.1.2. Agriculture & aquaculture - Annual & perennial non-timber crops - Small-holder farming2.1.3. Agriculture & aquaculture - Annual & perennial non-timber crops - Agro-industry farming4.1. Transportation & service corridors - Roads & railroads5.1.1. Biological resource use - Hunting & trapping terrestrial animals - Intentional use (species is the target)

#### Conservation

Justification for conservation actions: Important conservation actions include protecting the natural habitat of *Brachypelma verdezi*, creating management plans and conducting systematic monitoring to provide information about the recovery of subpopulations. The re-introduction of the species in some areas is also relevant for conservation and is being planned by a Mexican Unit for Management (UMA). As there is no official protected area in its range of occurrence, it is highly recommended to establish one in central Guerrero State in order to help in conservation, recovery and protection, not only of *B. verdezi*, but of *B. smithi* as well, since they are sympatric in that area. Adding to this, in order to avoid international trade incompatible with its survival, this species is currently listed on CITES Appendix II, along with all other species of the genus *Brachypelma* (CITES 2019). In Mexico, *Brachypelma verdezi* is being reared in captivity in a single UMA and sold legally. However, there is still an unknown amount of illegal pet trade of this species. Thus, the government of Mexico should collaborate with Mexican tarantula breeders to develop a system for certifying the origin of specimens used in the breeding programmes of the UMAs and developing better enforcement actions to curb the illegal pet trade by increasing surveillance and control of specimens collected and traded. Tax advantages for legal dealers would make their prices more competitive with the ones in the black market. Regarding public awareness, the single UMA that rears this species in captivity promotes educational activities regarding tarantula spiders aimed at students and the general public.

##### Conservation actions

Conservation action type: In Place

Conservation actions: 3.3.1. Species management - Species re-introduction - Reintroduction3.4.1. Species management - Ex-situ conservation - Captive breeding/artificial propagation5.1.1. Law & policy - Legislation - International level4.3. Education & awareness - Awareness & communications

##### Conservation actions

Conservation action type: Needed

Conservation actions: 1.1. Land/water protection - Site/area protection1.2. Land/water protection - Resource & habitat protection3.2. Species management - Species recovery5.4.1. Law & policy - Compliance and enforcement - International level5.4.2. Law & policy - Compliance and enforcement - National level5.4.3. Law & policy - Compliance and enforcement - Sub-national level6.4. Livelihood, economic & other incentives - Conservation payments

#### Conservation actions

Conservation action type: In Place

Conservation actions: 3.3.1. Species management - Species re-introduction - Reintroduction3.4.1. Species management - Ex-situ conservation - Captive breeding/artificial propagation5.1.1. Law & policy - Legislation - International level4.3. Education & awareness - Awareness & communications

#### Conservation actions

Conservation action type: Needed

Conservation actions: 1.1. Land/water protection - Site/area protection1.2. Land/water protection - Resource & habitat protection3.2. Species management - Species recovery5.4.1. Law & policy - Compliance and enforcement - International level5.4.2. Law & policy - Compliance and enforcement - National level5.4.3. Law & policy - Compliance and enforcement - Sub-national level6.4. Livelihood, economic & other incentives - Conservation payments

#### Other

Justification for use and trade: In Mexico, *Brachypelma verdezi* is being reared in captivity in a single UMA and sold legally. There is legal trade of captive-bred live specimens, but an unquantified amount of wild caught animals illegally traded is also known. The species might become more popular when it is easier to identify it. Captive-bred juveniles are sold for approximately US$15–$30 in Canada and the United States, for US$7 in Mexico and for US$22 in the EU (CEC 2017). Adult females are sold for approximately US$80 in the EU. During 2006-2016, between 58–64 live specimens of *B. verdezi* were traded internationally, none of these specimens was wild-caught and all were traded for commercial purposes (Cooper et al. 2019).

##### Use and trade

Use type: International

Use and trade: 13. Pets/display animals, horticulture16. Establishing ex-situ production *

##### Ecosystem services

Ecosystem service type: Less important

##### Research needed

Research needed: 2.3. Conservation Planning - Harvest & Trade Management Plan3.1. Monitoring - Population trends3.2. Monitoring - Harvest level trends3.3. Monitoring - Trade trends

Justification for research needed: This species is currently listed on CITES Appendix II and thus its international trade is regulated by an international agreement (CITES 2019). Thus, the government of Mexico should collaborate with Mexican tarantula breeders to develop a system for certifying the origin of specimens used in the breeding programmes of the Units for Management (UMAs). Additionally, systematic monitoring and protection could be undertaken in known subpopulations. Further studies are needed to confirm population, harvest and trade trends and how the latter affect harvest levels.

#### Use and trade

Use type: International

Use and trade: 13. Pets/display animals, horticulture16. Establishing ex-situ production *

#### Ecosystem services

Ecosystem service type: Less important

#### Research needed

Research needed: 2.3. Conservation Planning - Harvest & Trade Management Plan3.1. Monitoring - Population trends3.2. Monitoring - Harvest level trends3.3. Monitoring - Trade trends

Justification for research needed: This species is currently listed on CITES Appendix II and thus its international trade is regulated by an international agreement (CITES 2019). Thus, the government of Mexico should collaborate with Mexican tarantula breeders to develop a system for certifying the origin of specimens used in the breeding programmes of the Units for Management (UMAs). Additionally, systematic monitoring and protection could be undertaken in known subpopulations. Further studies are needed to confirm population, harvest and trade trends and how the latter affect harvest levels.

#### Viability analysis

### Sandinista lanceolatum

#### Species information

Scientific name: Sandinista lanceolatum

Species authority: (Simon, 1891)

Synonyms: *Aphonopelma lanceolatum* (Simon, 1891); *Brachypelma fossoria* Valerio, 1980

Common names: Costa Rican rustbrown.

Kingdom: Animalia

Phylum: Arthropoda

Class: Arachnida

Order: Araneae

Family: Theraphosidae

Taxonomic notes: Recently, Longhorn and Gabriel (2019) considered *Brachypelma fossorium* Valerio, 1980 a junior synomym of *Aphonopelma lanceolatum* (Simon, 1891). Adding to this, they conclude the species does not belong to *Aphonopelma* Pocock, 1901 and erected the monotypic genus *Sandinista* Longhorn & Gabriel, 2019 to accommodate it. As this species is currently listed on CITES under the genus *Brachypelma*, it was included in this work.

**Figure 64.**
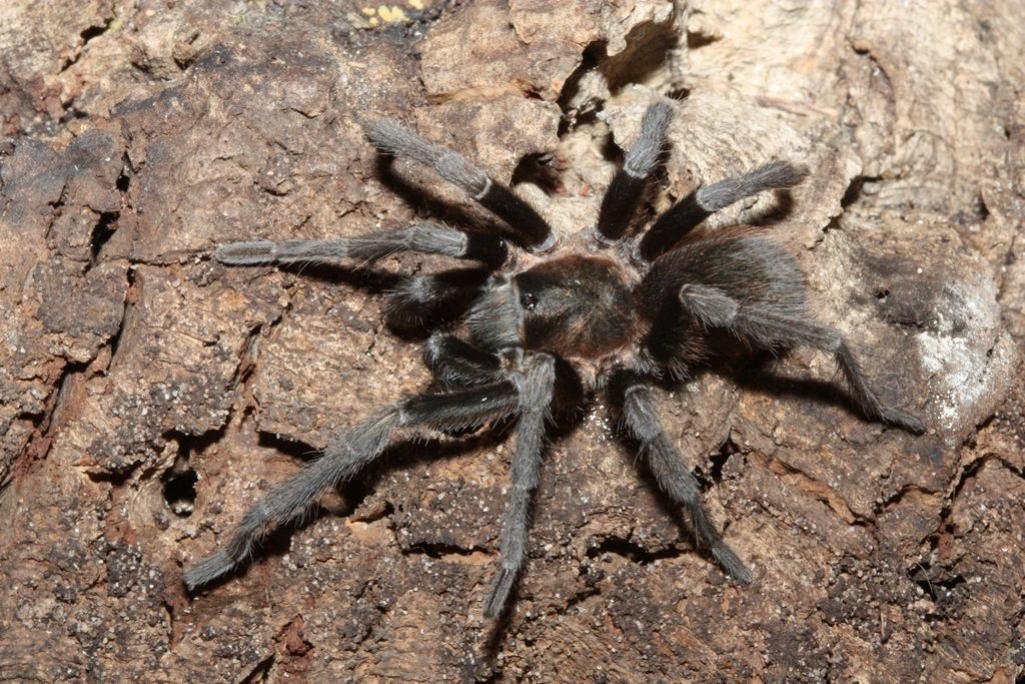
*Sandinista lanceolatum* (Simon, 1891) male from Jinotepe, Carazo department, Nicaragua. Photo: Stuart J. Longhorn.

**Figure 65.**
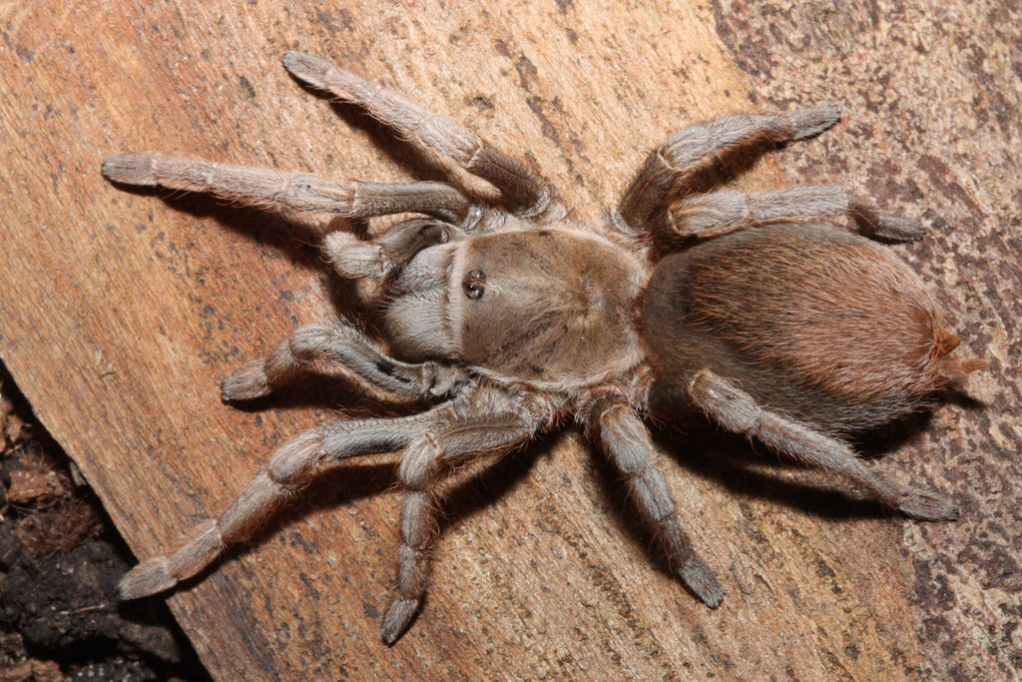
*Sandinista lanceolatum* (Simon, 1891) female from Granada, Granada department, Nicaragua. Photo: Stuart J. Longhorn.

Figure(s) or Photo(s): Figs 64, 65

Region for assessment: Global

#### Geographic range

Biogeographic realm: Neotropical

Countries: Costa RicaNicaragua

Map of records (Google Earth): Suppl. material 19

Basis of EOO and AOO: Species Distribution Model

Basis (narrative): A species distribution modelling has been performed to predict its potential range. See methods for details.

Min Elevation/Depth (m): 0

Max Elevation/Depth (m): 1040

Range description: *Sandinista lanceolatum* is restricted to the Pacific lowlands, recorded from north-western Guanacaste Province in Costa Rica (Valerio 1980, Gabriel and Longhorn 2015, [Bibr B4347904][Bibr B5309324]) to western Nicaragua, in the Departments of Granada, Managua and Carazo (Longhorn and Gabriel 2019). It has also been observed *in situ* in the southern Rivas department, eastern Boaco and Chinandega and northern department of León (Longhorn and Gabriel 2019). Two other Nicaraguan regions, where it has not yet been recorded (but expected), are the departments of Chontales and Mayasa, both of which are either near or on the Pacific lowland western coast (Longhorn and Gabriel 2019). Adding to this, the species distribution modelling predicts the occurrence of the species also in the Pacific lowlands of Honduras and El Salvador.

#### New occurrences

#### Extent of occurrence

EOO (km2): 81310

Trend: Decline (inferred)

Justification for trend: A decline in AOO is inferred from habitat loss due to deforestation, urbanisation and agricultural activities within its range.

Causes ceased?: No

Causes understood?: Yes

Causes reversible?: No

Extreme fluctuations?: No

#### Area of occupancy

Trend: Decline (inferred)

Justification for trend: A decline in AOO is inferred from habitat loss due to deforestation, urbanisation and agricultural activities within its range.

Causes ceased?: No

Causes understood?: Yes

Causes reversible?: No

Extreme fluctuations?: No

AOO (km2): 30300

#### Locations

Number of locations: Unknown

Justification for number of locations: Given the large range, the number of locations is much above that of any category thresholds.

Trend: Unknown

Extreme fluctuations?: No

#### Population

Number of individuals: Unknown

Trend: Decline (inferred)

Justification for trend: A decline in population size is inferred from habitat loss due to deforestation, urbanisation and agricultural activities within its range. Additionally, the population is harvested for the pet trade in Nicaragua.

Basis for decline: (c) a decline in area of occupancy, extent of occurrence and/or quality of habitat(d) actual or potential levels of exploitation

Causes ceased?: No

Causes understood?: Yes

Causes reversible?: No

Extreme fluctuations?: Unknown

Population Information (Narrative): A decline in population size is inferred from habitat loss due to deforestation, urbanisation and agricultural activities within its range. Additionally, the population is harvested for the pet trade in Nicaragua.

#### Subpopulations

Number of subpopulations: Unknown

Trend: Decline (inferred)

Justification for trend: A decline in subpopulation numbers is inferred from habitat loss due to deforestation, urbanisation and agricultural activities within its range.

Extreme fluctuations?: No

Severe fragmentation?: No

Justification for fragmentation: To our knowledge, the species is not subject to severe fragmentation.

#### Habitat

System: Terrestrial

Habitat specialist: No

Habitat (narrative): *Sandinista lanceolatum* inhabits the tropical dry forest of Central America, which consists of lower thorn brush vegetation with an upper canopy of mostly deciduous trees. The species can also adapt to some human-disturbed habitats such as scrub, farmland and edges of other human development areas in this zone with similar distinct wet and dry seasons (Longhorn and Gabriel 2019).

Trend in extent, area or quality?: Decline (inferred)

Justification for trend: The habitat of this species is inferred to be declining in area, extent and quality due to human activity, especially due to deforestation, in particular for agricultural activities.

**Figure 66.**
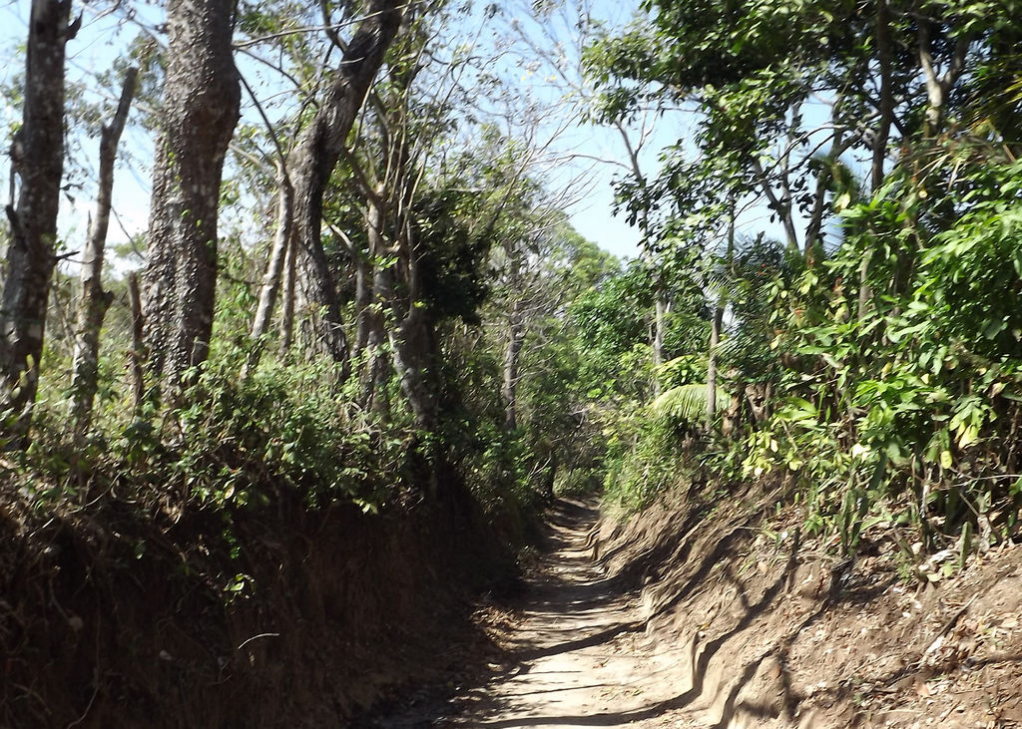
*Sandinista lanceolatum* habitat, tropical dry forest and thorn, near Granada, Granada department, Nicaragua. Photo: Ray Gabriel.

Figure(s) or Photo(s): Fig. 66

##### Habitat

Habitat importance: Major Importance

Habitats: 1.5. Forest - Subtropical/Tropical Dry

##### Habitat

Habitat importance: Marginal

Habitats: 14.2. Artificial/Terrestrial - Pastureland14.6. Artificial/Terrestrial - Subtropical/Tropical Heavily Degraded Former Forest

#### Habitat

Habitat importance: Major Importance

Habitats: 1.5. Forest - Subtropical/Tropical Dry

#### Habitat

Habitat importance: Marginal

Habitats: 14.2. Artificial/Terrestrial - Pastureland14.6. Artificial/Terrestrial - Subtropical/Tropical Heavily Degraded Former Forest

#### Ecology

Size: 39 mm (female); 23-33 mm (male).

Generation length (yr): 7

Dependency of single sp?: No

Ecology and traits (narrative): *Sandinista lanceolatum* is a species that modifies previously excavated burrows or can excavate their own unaided, often clearly defined obligate burrow-like retreats about 20 centimetres deep, sometimes under debris, such as large rocks and tree roots in subtropical dry forest. The burrows slope steeply for several centimetres, ending in a chamber. Burrows will typically have a layer of silk around the entrance to transmit the vibrations of prey movement and can be sealed with a further thin layer of silk across the diameter during daylight that may deter predators (e.g. ants, wasps etc.) and/or help maintain humidity inside the retreat. They appear to be tolerant of human-disturbance and burrows also can be sometimes found in scrub, farmland and edges of other non-intensive human development areas. Some small colonies were identified, containing several closely-spaced burrows, each containing a large individual, possibly mature females, as several other juveniles in burrows were observed mostly around bases of trees, either close to human habitation, in farmland or natural scrub, (Longhorn and Gabriel 2019). These spiders are nocturnal predators that wait near the entrance of their refuge from dusk and into the night to feed primarily on ground-dwelling arthropods (insects, other arachnids and some myriapods) or even small vertebrates. The mating season appears to be primarily in the dry months (October to December) when mature males wander in the open to search for females, although notably the male, described as *B. fossorium*, was collected in July which can suggest either a disjunct breeding season further south or just longer persistence of wandering males. The males are likely most active at night, cooler daylight hours and throughout overcast days. Adult females typically moult once per year, just prior to the onset of the annual male emergence. Females will produce cocoons (large silken egg sacs) in the dry season (December to April), with young emerging about two months later, with most young dispersing around the beginning of the rainy season (April/May) (Longhorn and Gabriel 2019). Valerio (1980) collected a female specimen along with several juveniles from the same burrow found in a cattle field, indicating that young may be remarkably tolerant of each other prior to dispersal.

#### Threats

Justification for threats: The ongoing major threat to this species is habitat destruction, especially regarding the subpopulations of *S. lanceolatum* around the Nicaraguan cities of León, Managua, Rivas and the Costa Rican city of Filadelfia de Guanacaste, that are expected to be declining due to the expansion of intense agriculture and urbanisation. Vehicular traffic on roads, as the main Panamerican highway goes straight through nearly the whole of its range along that coast, can also pose a threat since males are frequently found run over on them during the mating season. Adding to this, the construction of roads causes degradation of the natural habitat and also facilitates the access of smugglers to wild populations. Despite an official large-scale trade not being recorded for *S. lanceolatum*, it is illegally traded as *A. seemani* and/or *B. albopilosum* (Sherwood et al. 2016) due to its general similarity and co-occurrence with these species (Longhorn and Gabriel 2019) or as *Aphonopelma paloma* Prentice, 1993. Thus illegal commerce can also be considered a threat, although a very small one.

##### Threats

Threat type: Ongoing

Threats: 1.1. Residential & commercial development - Housing & urban areas2.1.2. Agriculture & aquaculture - Annual & perennial non-timber crops - Small-holder farming2.1.3. Agriculture & aquaculture - Annual & perennial non-timber crops - Agro-industry farming4.1. Transportation & service corridors - Roads & railroads5.1.1. Biological resource use - Hunting & trapping terrestrial animals - Intentional use (species is the target)

#### Threats

Threat type: Ongoing

Threats: 1.1. Residential & commercial development - Housing & urban areas2.1.2. Agriculture & aquaculture - Annual & perennial non-timber crops - Small-holder farming2.1.3. Agriculture & aquaculture - Annual & perennial non-timber crops - Agro-industry farming4.1. Transportation & service corridors - Roads & railroads5.1.1. Biological resource use - Hunting & trapping terrestrial animals - Intentional use (species is the target)

#### Conservation

Justification for conservation actions: Despite some protected areas being found in the range of *S. lanceolatum* (such as the National Parks Palo Verde and Santa Rosa in Guanacaste Province, Costa Rica, as well as Laguna de Apoyo Reserve and possibly lower parts of Volcán Masaya National Park in Nicaragua), the ongoing major threat to this species is habitat loss. Thus, it is imperative to adopt measures to protect and recover the natural habitat of the species, for example around Filadelfia de Guanacaste, Costa Rica, where agriculture is very intense. The creation of management plans and systematic monitoring would help to provide information about the recovery of subpopulations. Regarding international trade, this species is currently listed on CITES Appendix II, under the name *Brachypelma fossorium* (CITES 2019). However, Longhorn and Gabriel (2019) suggest the removal of the species from the CITES list, because its small scale commercial trade can be considered sustainable.

##### Conservation actions

Conservation action type: In Place

Conservation actions: 1.1. Land/water protection - Site/area protection5.1.1. Law & policy - Legislation - International level

##### Conservation actions

Conservation action type: Needed

Conservation actions: 1.1. Land/water protection - Site/area protection1.2. Land/water protection - Resource & habitat protection2.1. Land/water management - Site/area management2.3. Land/water management - Habitat & natural process restoration3.2. Species management - Species recovery

#### Conservation actions

Conservation action type: In Place

Conservation actions: 1.1. Land/water protection - Site/area protection5.1.1. Law & policy - Legislation - International level

#### Conservation actions

Conservation action type: Needed

Conservation actions: 1.1. Land/water protection - Site/area protection1.2. Land/water protection - Resource & habitat protection2.1. Land/water management - Site/area management2.3. Land/water management - Habitat & natural process restoration3.2. Species management - Species recovery

#### Other

Justification for use and trade: This species is currently listed on CITES Appendix II under the genus *Brachypelma* and thus its international trade is regulated by an international agreement (CITES 2019). Officially, only a single wild-caught, live specimen, traded for personal purposes, was reportedly imported by the USA from Mexico in 2006 and thus it is considered rare in international trade (Cooper et al. 2019). In accordance with Longhorn and Gabriel (2019), the commercial trade in this species (under any of its associated taxonomic names), is limited, neither in large-scale nor persistent. As of early 2018, Longhorn and Gabriel (2019) recognise that less than 500 individuals entered the pet hobby market and consider this sustainable. Some misidentified individuals of *S. lanceolatum* were exported in the few years prior to 2016 from Nicaragua for live trade into both North America and Europe, together with exports of *A. seemanni* and/or *B. albopilosum* (Sherwood et al. 2016, Longhorn and Gabriel 2019).

##### Use and trade

Use type: International

Use and trade: 13. Pets/display animals, horticulture

##### Ecosystem services

Ecosystem service type: Less important

##### Research needed

Research needed: 1.1. Research - Taxonomy1.2. Research - Population size, distribution & trends1.4. Research - Harvest, use & livelihoods3.2. Monitoring - Harvest level trends3.3. Monitoring - Trade trends

Justification for research needed: As also pointed by Longhorn and Gabriel (2019), the habitat degradation is the most relevant threat to the species. Thus, it would be important to research the impact of land use and habitat degradation in the distribution and viability of *S. lanceolatum.* Harvesting and trade tendencies should also be studied to better provide information about conservation decisions.

#### Use and trade

Use type: International

Use and trade: 13. Pets/display animals, horticulture

#### Ecosystem services

Ecosystem service type: Less important

#### Research needed

Research needed: 1.1. Research - Taxonomy1.2. Research - Population size, distribution & trends1.4. Research - Harvest, use & livelihoods3.2. Monitoring - Harvest level trends3.3. Monitoring - Trade trends

Justification for research needed: As also pointed by Longhorn and Gabriel (2019), the habitat degradation is the most relevant threat to the species. Thus, it would be important to research the impact of land use and habitat degradation in the distribution and viability of *S. lanceolatum.* Harvesting and trade tendencies should also be studied to better provide information about conservation decisions.

#### Viability analysis

### Sericopelma angustum

#### Species information

Scientific name: Sericopelma angustum

Species authority: (Valerio, 1980)

Synonyms: *Brachypelma augusta* Valerio, 1980; *Brachypelma angustum* Valerio, 1980; *Euathlus angustus* (Valerio, 1980).

Common names: Costa Rican redrump

Kingdom: Animalia

Phylum: Arthropoda

Class: Arachnida

Order: Araneae

Family: Theraphosidae

Taxonomic notes: CITES Appendix II has not been amended to reflect the taxonomic revision. Thus, this species is in CITES as *Brachypelma angustum* Valerio, 1980. The male is unknown and undescribed.

Region for assessment: Global

#### Geographic range

Biogeographic realm: Neotropical

Countries: Costa Rica

Map of records (Google Earth): Suppl. material 20

Basis of EOO and AOO: Unknown

Basis (narrative): This species EOO and AOO are unknown.

Range description: *Sericopelma angustum* is endemic to Costa Rica and only known from the type locality - San Pedro de Arenal, Alajuela province (Valerio 1980). It probably inhabits northern Costa Rica (Gabriel and Longhorn 2015), in the plains of San Carlos cantón (Valerio 1980), although this can only be speculated.

#### New occurrences

#### Extent of occurrence

EOO (km2): Unknown

Trend: Unknown

Causes ceased?: Unknown

Causes understood?: Unknown

Causes reversible?: Unknown

Extreme fluctuations?: Unknown

#### Area of occupancy

Trend: Unknown

Causes ceased?: Unknown

Causes understood?: Unknown

Causes reversible?: Unknown

Extreme fluctuations?: Unknown

AOO (km2): Unknown

#### Locations

Number of locations: Unknown

Trend: Unknown

Extreme fluctuations?: Unknown

#### Population

Number of individuals: Unknown

Trend: Unknown

Causes ceased?: Unknown

Causes understood?: Unknown

Causes reversible?: Unknown

Extreme fluctuations?: Unknown

Population Information (Narrative): No population information is available.

#### Subpopulations

Number of subpopulations: Unknown

Trend: Unknown

Extreme fluctuations?: Unknown

Severe fragmentation?: Unknown

#### Habitat

System: Terrestrial

Habitat specialist: Unknown

Habitat (narrative): *Sericopelma angustum* likely inhabits moist tropical broadleaf forests of Central America, yet it is only known from its type locality (San Pedro de Arenal, Alajuela province, Costa Rica), hence its potential habitat is unclear (after Gabriel and Longhorn 2015).

Trend in extent, area or quality?: Unknown

##### Habitat

Habitat importance: Major Importance

Habitats: 1.6. Forest - Subtropical/Tropical Moist Lowland

#### Habitat

Habitat importance: Major Importance

Habitats: 1.6. Forest - Subtropical/Tropical Moist Lowland

#### Ecology

Size: 58.9 mm (holotype female), unknown male.

Generation length (yr): 7

Dependency of single sp?: No

Ecology and traits (narrative): Since the single specimen known is the female holotype, there is virtually no data regarding ecological aspects of this species. It probably presents the same ecological traits as other members of the genus *Sericopelma* i.e. fossorial species that modifies previously excavated burrows or self-excavates their own, often only making minor alterations to natural small cavities under debris, such as tree roots and/or those made and abandoned by rodents in shaded forest. The burrows are expected to have no traces of silk around the entrance, giving no clear indication that there is a spider inside. These spiders are expected to be nocturnal predators that wait near the entrance of their refuge from dusk and into the night to feed primarily on ground-dwelling arthropods (insects, other arachnids and some myriapods) or even small vertebrates. The male of this species in undescribed and nothing is known on its reproductive lifestyle.

#### Threats

Justification for threats: Much of its probable habitat has already been disrupted by human activity, such as sugar cane plantations, cattle pasture (Gabriel and Longhorn 2015) and urbanisation. Although small, harvesting to meet trafficking demand may also be of concern.

##### Threats

Threat type: Ongoing

Threats: 1.1. Residential & commercial development - Housing & urban areas2.1.2. Agriculture & aquaculture - Annual & perennial non-timber crops - Small-holder farming2.3.2. Agriculture & aquaculture - Livestock farming & ranching - Small-holder grazing, ranching or farming5.1.1. Biological resource use - Hunting & trapping terrestrial animals - Intentional use (species is the target)

#### Threats

Threat type: Ongoing

Threats: 1.1. Residential & commercial development - Housing & urban areas2.1.2. Agriculture & aquaculture - Annual & perennial non-timber crops - Small-holder farming2.3.2. Agriculture & aquaculture - Livestock farming & ranching - Small-holder grazing, ranching or farming5.1.1. Biological resource use - Hunting & trapping terrestrial animals - Intentional use (species is the target)

#### Conservation

Justification for conservation actions: The major threat to the species seems to be habitat destruction, as it is for most theraphosid spiders (Gabriel and Longhorn 2015). As human-modification of land through agriculture (particularly sugar-cane plantations and cattle pasture) are degrading the possible natural habitat of S. augustum, important conservation actions include the protection of its natural habitat through the creation of protected areas. This species was previously included in Brachypelma and was listed on CITES Appendix II, along with all other members of the genus. As of 2018, Appendix II still lists the species as B. angustum (CITES 2019).

##### Conservation actions

Conservation action type: In Place

Conservation actions: 5.1.1. Law & policy - Legislation - International level

##### Conservation actions

Conservation action type: Needed

Conservation actions: 1.1. Land/water protection - Site/area protection5.1.2. Law & policy - Legislation - National level

#### Conservation actions

Conservation action type: In Place

Conservation actions: 5.1.1. Law & policy - Legislation - International level

#### Conservation actions

Conservation action type: Needed

Conservation actions: 1.1. Land/water protection - Site/area protection5.1.2. Law & policy - Legislation - National level

#### Other

Justification for use and trade: This species is currently listed on CITES Appendix II and thus its international trade is regulated by an international agreement (CITES 2019). Officially, just a small trade of *S. angustum* is recorded Twenty three live specimens were traded internationally under the synonymous name of '*Brachypelma angustum*' during 2006–2016, all for commercial purposes and none was declared as wild-caught (Cooper et al. 2019). However, it is probable that none of those specimens actually corresponded to *S. angustum*, instead being, in fact, hobby-bred hybrids of different species. Regardless, international trade in the species is still regulated by CITES, even after being transferred from the genus *Brachypelma* to *Sericopelma*. No specimens were traded internationally after this taxonomic transfer (Cooper et al. 2019).

##### Use and trade

Use type: International

Use and trade: 13. Pets/display animals, horticulture

##### Ecosystem services

Ecosystem service type: Less important

##### Research needed

Research needed: 1.1. Research - Taxonomy1.2. Research - Population size, distribution & trends1.3. Research - Life history & ecology1.5. Research - Threats

Justification for research needed: Since the only specimen known is the holotype, after taxonomic clarification, the first step is to obtain basic information on distribution, population and ecology of *S. angustum*, along with possible threats within its range.

#### Use and trade

Use type: International

Use and trade: 13. Pets/display animals, horticulture

#### Ecosystem services

Ecosystem service type: Less important

#### Research needed

Research needed: 1.1. Research - Taxonomy1.2. Research - Population size, distribution & trends1.3. Research - Life history & ecology1.5. Research - Threats

Justification for research needed: Since the only specimen known is the holotype, after taxonomic clarification, the first step is to obtain basic information on distribution, population and ecology of *S. angustum*, along with possible threats within its range.

#### Viability analysis

### Sericopelma embrithes

#### Species information

Scientific name: Sericopelma embrithes

Species authority: (Chamberlin & Ivie, 1936)

Synonyms: *Eurypelma embrithes* Chamberlin & Ivie, 1936; *Brachypelma embrithes* (Chamberlin & Ivie, 1936); *Avicularia embrithes* (Chamberlin & Ivie, 1936); *Aphonopelma embrithes* (Chamberlin & Ivie, 1936).

Common names: Barro Colorado Island brown

Kingdom: Animalia

Phylum: Arthropoda

Class: Arachnida

Order: Araneae

Family: Theraphosidae

Taxonomic notes: CITES Appendix II has not been amended to reflect the taxonomic revision. Thus, this species is listed in CITES as *Brachypelma embrithes* (Chamberlin & Ivie, 1936). Despite being known and currently present in several zoological collections, the male of this species remains undescribed.

**Figure 67.**
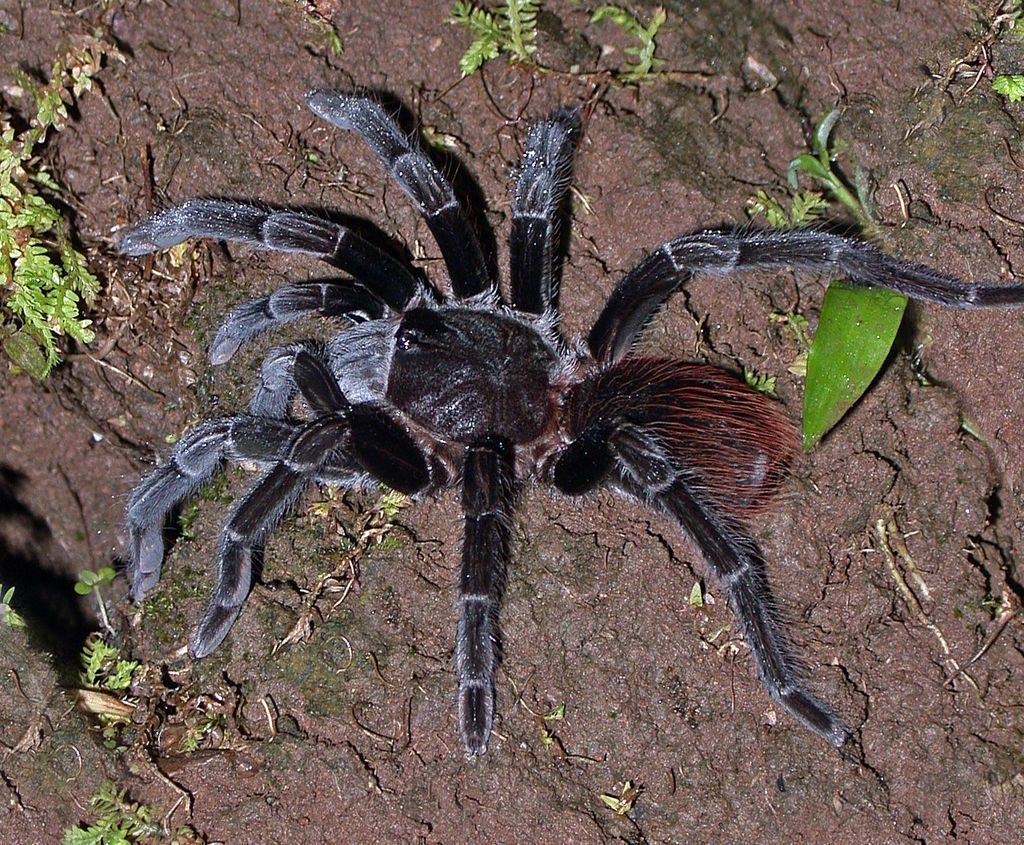
*Sericopelma embrithes* (Chamberlin & Ivie, 1936) female from Barro Colorado Island, Panama province, Panama. Photo: Neville Winchester.

**Figure 68.**
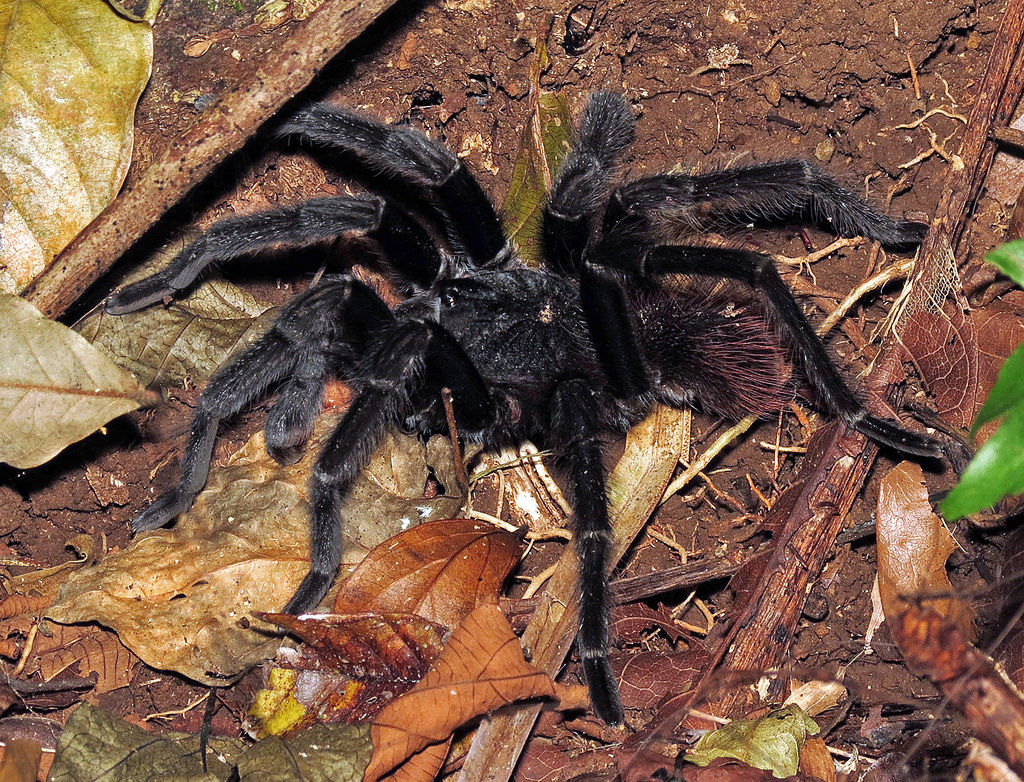
*Sericopelma embrithes* (Chamberlin & Ivie, 1936) male from Barro Colorado Island, Panama. Photo: Katja Schultz.

Figure(s) or Photo(s): Figs 67, 68

Region for assessment: Global

#### Geographic range

Biogeographic realm: Neotropical

Countries: Panama

Map of records (Google Earth): Suppl. material 21

Basis of EOO and AOO: Unknown

Basis (narrative): This species EOO and AOO are unknown.

Range description: *Sericopelma embrithes* is only known from the type locality, Barro Colorado Island, in Panama (Chamberlin and Ivie 1936, Gabriel and Longhorn 2015). However, it is likely that its original range included areas destroyed during damming of the Chagres River for the construction of the Panama canal (unpublished data).

#### New occurrences

#### Extent of occurrence

EOO (km2): Unknown

Trend: Unknown

Causes ceased?: Unknown

Causes understood?: Unknown

Causes reversible?: Unknown

Extreme fluctuations?: Unknown

#### Area of occupancy

Trend: Unknown

Causes ceased?: Unknown

Causes understood?: Unknown

Causes reversible?: Unknown

Extreme fluctuations?: Unknown

AOO (km2): Unknown

#### Locations

Number of locations: Unknown

Trend: Unknown

Extreme fluctuations?: No

#### Population

Number of individuals: Unknown

Trend: Unknown

Causes ceased?: Unknown

Causes understood?: Unknown

Causes reversible?: Unknown

Extreme fluctuations?: Unknown

Population Information (Narrative): No population information is available.

#### Subpopulations

Number of subpopulations: Unknown

Trend: Unknown

Extreme fluctuations?: Unknown

Severe fragmentation?: Unknown

#### Habitat

System: Terrestrial

Habitat specialist: Unknown

Habitat (narrative): *Sericopelma embrithes* is only known from its type locality, Barro Colorado Island, which is covered by a semi-evergreen moist tropical forest (Knight 1975). Barro Colorado is a biological reserve that receives an annual average rainfall of 2,631 mm, has an annual average daily maximum air temperature of 28.5°C and currently is 100% forested, with a few man-made clearings (Basset et al. 2015).

Trend in extent, area or quality?: Unknown

Justification for trend: The habitat of this species is of unknown trend as there has been no research on this for *S. embrithes.* It remains to be studied if the species range extends throughout adjacent regions and, if so, the impact of urbanisation and other human modifications on the habitat around the Panama canal zone urgently need to be evaluated.

##### Habitat

Habitat importance: Major Importance

Habitats: 1.6. Forest - Subtropical/Tropical Moist Lowland

#### Habitat

Habitat importance: Major Importance

Habitats: 1.6. Forest - Subtropical/Tropical Moist Lowland

#### Ecology

Size: 58.6 mm (female holotype); unknown male.

Generation length (yr): 7

Dependency of single sp?: No

Ecology and traits (narrative): Since the single specimen described is the female holotype, there is virtually no data regarding ecological aspects of this species. It probably presents the same ecological traits as other members of the genus *Sericopelma* i.e. fossorial species that modifies previously excavated burrows or self-excavates their own, often only minor alterations to natural small cavities being made under debris such as tree roots and/or those made and abandoned by rodents in shaded forest. The burrows are expected to have no traces of silk around the entrance, giving no clear indication that there is a spider inside. These spiders are expected to be nocturnal predators that wait near the entrance of their refuge from dusk and into the night to feed primarily on ground-dwelling arthropods (insects, other arachnids and some myriapods) or even small vertebrates. The male of this species is undescribed and nothing is known on its reproductive lifestyle.

#### Threats

Justification for threats: It is likely that the original range of *S. embrithes* included areas destroyed by the damming of the Chagres River for the construction of the Panama Canal. Although no major disturbance of the island's vegetation has taken place after Barro Colorado was set aside as a reserve in 1923 (Croat 1978), a major portion of the island lay deforested until 1905 (Knight 1975), which could have affected the existing subpopulation of *S. embrithes*. There is no record of this species in trade between 2006-2016 (Cooper et al. 2019).

##### Threats

Threat type: Past

Threats: 7.2.10. Natural system modifications - Dams & water management/use - Large dams

#### Threats

Threat type: Past

Threats: 7.2.10. Natural system modifications - Dams & water management/use - Large dams

#### Conservation

Justification for conservation actions: The type locality of Barro Colorado Island is a protected national biological reserve since 1923. Thus, the population of *S. embrithes* on the island is not threatened. However, it is necessary to protect other areas with similar habitat where this species may live, in order to guarantee the necessary genetic diversity. Adding to this, *S. embrithes* is currently listed on CITES Appendix II and thus its international trade is regulated by an international agreement (CITES 2019). This species belonged to *Brachypelma* when the genus was included in the CITES Appendix II and remained listed on it, despite being transferred to another genus (CITES 2019).

##### Conservation actions

Conservation action type: In Place

Conservation actions: 1.1. Land/water protection - Site/area protection5.1.1. Law & policy - Legislation - International level

##### Conservation actions

Conservation action type: Needed

Conservation actions: 1.2. Land/water protection - Resource & habitat protection

#### Conservation actions

Conservation action type: In Place

Conservation actions: 1.1. Land/water protection - Site/area protection5.1.1. Law & policy - Legislation - International level

#### Conservation actions

Conservation action type: Needed

Conservation actions: 1.2. Land/water protection - Resource & habitat protection

#### Other

Justification for use and trade: This species is currently listed on CITES Appendix II and thus is protected by an international agreement (CITES 2019). No trade in specimens of *S. embrithes* (or *B. embrithes*) was recorded in the UNEP-WCMC CITES Trade Database during 2006–2016 (Cooper et al. 2019). As in *S. angustum*, an important point to be highlighted is that *S. embrithes* is still protected by international commercial trade regulation (CITES), even after being transferred from the genus *Brachypelma* to *Sericopelma*.

##### Use and trade

Use type: International

##### Ecosystem services

Ecosystem service type: Less important

##### Research needed

Research needed: 1.1. Research - Taxonomy1.2. Research - Population size, distribution & trends1.3. Research - Life history & ecology1.5. Research - Threats

Justification for research needed: The identity of this species is poorly known. Taxonomic research is still needed to solve this problem, as well as to generate geographic considerations that could be vital to make confident decisions about both generic and species identities, since many tarantula species have narrow distributions (Gabriel and Longhorn 2015). After taxonomic clarification, the first step is to obtain basic information on distribution, population and ecology of *S. embrithes*, along with possible threats within its range.

#### Use and trade

Use type: International

#### Ecosystem services

Ecosystem service type: Less important

#### Research needed

Research needed: 1.1. Research - Taxonomy1.2. Research - Population size, distribution & trends1.3. Research - Life history & ecology1.5. Research - Threats

Justification for research needed: The identity of this species is poorly known. Taxonomic research is still needed to solve this problem, as well as to generate geographic considerations that could be vital to make confident decisions about both generic and species identities, since many tarantula species have narrow distributions (Gabriel and Longhorn 2015). After taxonomic clarification, the first step is to obtain basic information on distribution, population and ecology of *S. embrithes*, along with possible threats within its range.

#### Viability analysis

## Discussion

Turner et al. (2018) have discussed the implications of taxonomic uncertainty for the effectiveness of conservation strategies for vulnerable lineages. As pointed out before, a taxonomic review of the genus *Brachypelma* is being published soon and some taxonomic changes are to occur, including a split in the genus. However, we need to stress that this revision will not invalidate the discussion done here because, although some names will change, the species boundaries will remain the same. Besides, our focus in this paper is more the CITES-listed species than the *Brachypelma* genus *per se*.

Discussion

### Ecology

Ecological data about most tarantula species are scarce and *Brachypelma*, *Aphonopelma*, *Sandinista* and *Sericopelma* are no exception to the rule. To date, amongst the species listed by CITES, the ones with the most information available regarding ecological traits are *B. klaasi* (Yáñez 1998, Yáñez and Locht 1998, Yáñez et al. 1999, Locht et al. 1999, Yáñez and Floater 2000) and *B. vagans* (M'rabet et al. 2005, Machkour‐M'Rabet et al. 2009, Machkour-M’Rabet et al. 2011, Machkour-M’Rabet et al. 2012, Hénaut et al. 2015), which have been the subject of continued research for several years. In general, *Brachypelma* spp., *S. lanceolatum* and *A. pallidum* are fossorial animals which either modify previously excavated burrows or can excavate their own, altering natural small cavities or building burrow-like retreats under debris (such as rocks) or amongst larger tree roots in dense thickets of subtropical dry forest, pine-oak forest or, exceptionally, around edges of cultivated fields. Regarding *Sericopelma* spp., there is virtually no data on the species listed by CITES, since only few specimens are known for *S. angustum* and *S. embrithes*. They probably present the same ecological traits as other members of the genus *Sericopelma*, i.e. being fossorial species that modify previously excavated burrows or can excavate their own, often with only minor alterations to natural small cavities under debris, such as tree roots and/or those made and abandoned by rodents in shaded forest.

*Brachypelma vagans* is a unique case amongst all species assessed concerning two aspects of its biology. It is the only species that was ever reported to be invasive outside its range of origin and it is the only with relevance for biological control. Subpopulations of *B. vagans* were first reported by Machkour-M’Rabet et al. (2012) in Cozumel, an island in the Mexican Caribbean. Some individuals were released in the island in 1971 and now populations of *B. vagans* are very well established, being present in numerous villages on the island (Machkour-M’Rabet et al. 2012). Presumably specimens from the pet trade have also colonised central Florida, an area with soil type, vegetation type and climate very similar to its natural habitat on the Yucatán Peninsula in Mexico, indicating the high adaptability of this species (Edwards and Hibbard 1999). Thus, the monitoring of the population trends in both areas is very important to prevent a loss of biodiversity or changes to the structure and composition of native communities.

*Brachypelma vagans* is considered a potential predator of *Centruroides* Marx, 1890, scorpions that live in peri-domestic areas in Yucatán, where they represent a real human health problem (Dor et al. 2011). The presence of tarantulas in backyards might actually prove to be a good way to prevent scorpions from entering houses and be used as an argument for protecting these spiders (Dor et al. 2011). No information is known about other types of ecological services for the other species assessed, but it is likely that other species of *Brachypelma* can act as biological control agents of other arthropods as other tarantula species probably do (Pérez‐Miles et al. 2005).

### Threat assessments

Amongst all 21 species assessed, only 16 had sufficient data on their distribution, ecology and threats to properly understand their current status and suggest possible conservation measures. Species presenting EOO and AOO smaller than 20,000 km^2^ and 2,000 km^2^, respectively, are considered with restricted distributions and can be included in threatened categories according to IUCN (2019), if they meet other requirements. The species with the more restricted estimated range are *B. boehmei* (EOO and AOO < 500 km^2^), *B. baumgarteni* (EOO and AOO < 3,000 km^2^), *B. klaasi* (EOO < 7,000 km^2^ and AOO < 3,000 km^2^) and *B. schroederi* (EOO < 4,000 km^2^ and AOO < 3,000 km^2^), all endemic to Mexico. The most widespread species have both EOO and AOO above 50,000 km^2^, such as *B. vagans* (EOO > 121,000 km^2^ and AOO > 73,000 km^2^) and *B. kahlenbergi* (EOO > 89,000 km^2^ and AOO > 51,000 km^2^).

A decline in AOO and EOO was inferred to all species for which these values are estimated, except *A. pallidum* whose EOO is considered to be stable. The main reason for the declining AOO and EOO for the majority of species is deforestation caused by human activities, which often lead to the complete loss of subpopulations across their range. Urbanisation also threatens all the species assessed here except *B. auratum*, *B. sabulosum*, *S. embrithes* (which is found only in Barro Colorado island, a conservation unit), *B. andrewi* and *B. aureoceps* (both species without reliable locality information). The other most common anthropogenic factor that threatens the CITES-listed spiders is agricultural activity, mainly poorly managed non-industrial agriculture of small-holders, but also large-scale agricultural production of key crops. Adding to this, there is substantial livestock farming affecting some species, partly through extensive ranching, but also several areas with more intensive and destructive practices. Six of the species do not appear to tolerate habitats with some degree of human disturbance, namely anthropogenic alterations: *B. hamorii*, *B. auratum*, *B. baumgarteni*, *B. boehmei*, *B. klaasi* and *B. smithi*.

Although affecting in a less serious way the EOO and AOO of species, the main cause for loss of individuals in most species is overharvesting, particularly for adults that reach much higher prices in the market with consequent higher demand and that are the only ones contributing to the effective population size, according to the IUCN criteria. Harvesting of wild populations to meet the illegal trade is causing the decline in populations of at least 14 species of *Brachypelma*. Most of the illegal trade information comes from grey literature and anecdotal reports, since there is a lack of consistent data about tarantula trafficking and the few seizures do not provide a good estimation of the real volume of smuggled spiders. Although there is barely any baseline data for *Brachypelma* populations prior to illegal collection and the rates of illegal collection are unknown, researchers working for many years with wild populations have noticed a decline in those populations associated with a rise in the pet trade demand. The inclusion of the genus on CITES Appendix II may have reduced the exploitation of wild populations for meeting the international trade, but not the now growing Mexican pet trade (Turner et al. 2018).

The high demand by the illegal pet market for some species such as *B. auratum*, *B. baumgarteni* and *B. smithi* (also reported by Arroyo-Quiroz and Wyatt 2019) can be considered as a critical threat since the legally regulated captivity production is comparably low. Traders often collect the specimens by themselves, but they can also reward local people for doing this. This last situation occurs very often with *B. auratum*, the most illegally traded species currently in Mexico, which is caught in the wild by locals and then is regularly sold in Mexico City markets. *Brachypelma auratum*, along with *B. emilia*, is replacing *B. smithi* in the black market within Mexico. *Brachypelma smithi* is still very common in the legal pet trade, both within Mexico and worldwide and, even in recent years, many specimens have again been taken from the wild due to international demand for what collectors call ʺpure blood linesʺ. Consequently, some wild populations of this species had severe reductions. *Brachypelma schroederi* is considered rare in trade but has a high demand and *B. klaasi* seems to be rare in trade market, but adults appear at times from unknown sources, increasing demand when available.

Overharvesting to meet the demands of the illegal pet trade is common for many *Brachypelma* species, but some additional collection for use in traditional medicine also occurs. For instance, there is a cultural association with the Ch’ol, a Mayan ethnic group from Chiapas and Campeche States and *B. vagans* since they use it in their traditional medicine (Machkour-M’Rabet et al. 2011). A similar situation occurs with *B. smithi*, since it is also used in traditional medicine, especially in the Pacific coastal area of Guerrero State (George Odell and Alejandro Alagon, pers. comm.). Yet, the collection for medicinal purposes should be insignificant compared with the pressure exerted by the pet trade business.

Apart from ecological, economic or marketing reasons, both legal and illegal pet trade are intrinsically related to taxonomic aspects and are changed and regulated by them. Specimens of *S. lanceolatum*, previously known as *B. fossorium*, from Nicaragua are mistakenly traded as *Aphonopelma seemanni* (F. O. Pickard-Cambridge, 1897) or as *Aphonopelma paloma* Prentice, 1993. *Brachypelma hamorii* is often misidentified and traded as *B. smithi* and *B. kahlenbergi* has often been misidentified in the pet trade with other species of *Brachypelma* with red abdomen, such as *B. vagans* and *B. sabulosum*. The difficulty in identifying several such species can also affect their amount of trade. This may be the case for *B. verdezi and B. epicureanum*, that might become more popular when it is easier to identify them. Polymorphisms in some species, such as *B. vagans* and *B. smithi*, can also cause misidentifications. Many specimens of *B. smithi* were sold under the junior synonym *B. annitha* (Mendoza and Francke 2017) and so the numbers of specimens traded by both names should be considered to see the complete scenario of the trade of *B. smithi*. Thus, taxonomic issues can affect the pet trade numbers and trends. Changes in the status of a name, i.e. synonymies and transfers to another genus, should also be considered when analysing market trends. On the other hand, collection intensity of some areas can change in response to the demands of the market. Thus, it is essential to understand and to track the fluctuations in the legal and illegal trade, being able to understand all these taxonomic issues and take them into consideration when planning conservation actions and proposing measures to curb trafficking.

For some species, such as *B. epicureanum*, *B. smithi* and *B. verdezi*, touristic activity is another human factor very relevant to the decline in EOO and AOO. The latter two are found in the coastal area of Guerrero State in Mexico, where many small towns are rapidly developing into major tourist centres. Equally, *B. epicureanum* is found in Yucatán Peninsula, a place with an intense tourist activity due to the Mayan archaeological sites in the area. Tourist activities negatively affect the species habitat since they imply growth of urbanised areas, road construction and other anthropogenic alterations.

Natural phenomena can also affect the occurrence of species in some areas. Hurricanes and frequent rising water, which are increasing in frequency due to climate change, can cause decline in habitat quality and consequent EOO and AOO of species such as *B. kahlenbergi* and *B. epicureanum*. The area where these species occur, respectively, the coastal area of Veracruz and Quintana Roo States in Mexico, are constantly affected by hurricanes and tropical depressions or storms (SEMARNAT 2008). A direct effect of natural disasters in *Brachypelma* is known for *B. smithi*, since two subpopulations were almost decimated by the flooding of Papagayo River in Guerrero Mexico during the hurricane season of 2012-2013 (Mendoza and Francke 2017). Local sources indicate that several subpopulations of *B. boehmei* and *B. baumgarteni* in coastal areas of Guerrero and Michoacán States of Mexico, respectively, are also affected by hurricanes. Thus, natural phenomena should also be considered when planning conservation actions for these species.

Climate change is therefore also a factor to be considered. Projections for Mexican fauna under liberal and conservative global climate change scenarios show that, despite extinctions and drastic range reductions being predictable to few taxa, the species turnover in some communities is predicted to be high, resulting in severe ecological disturbances with unpredictable consequences over the biotic network (Peterson et al. 2002).

Habitat fragmentation is a well-known source of negative impacts on many different taxonomic groups, including invertebrates (Didham et al. 1996). Agriculture and urbanisation has caused severe alterations on landscape structure throughout the world and developing tropical areas such as Mexico are no different (Machkour-M’Rabet et al. 2012). Anthropogenic disturbances lead to habitat fragmentation and consequently to barriers that decrease the connectivity amongst populations (Coulon et al. 2004). The lack of connectivity amongst small subpopulations increases the extinction risk to the taxon and, in this situation, the term severely fragmented can be applied to a species (IUCN 2019).

Severe fragmentation was detected in 13 species, namely *B. albiceps*, *B. auratum*, *B. baumgarteni*, *B. boehmei*, *B. emilia*, *B. epicureanum*, *B. hamorii*, *B. klaasi*, *B. kahlenbergi*, *B. schroederi*, *B. smithi*, *B. vagans* and *B. verdezi*. Most of the range of these species is in habitat patches that are separated from other patches by a large distance when considering the relatively poor tarantula dispersal abilities, often lower than other arthropod taxa (Janowski-Bell and Horner 1999). Thus, considering IUCN definitions (IUCN 2019), as the available data indicate that at least half of the individuals of the 13 aforementioned tarantula species are not sustainable and rescue effects are not possible given the fragmentation of the species habitat, they were considered severely fragmented. Severe fragmentation is therefore one of the most relevant threats to the most endangered *Brachypelma* species and should be made a priority aspect to deal with when proposing conservation actions for the group.

Roads are per se a source of habitat degradation, as they cause habitat loss and fragmentation which, in turn, lead to a decline in some *Brachypelma* subpopulations. However, the influence of road construction can go further: besides allowing the fast growth of the urban areas, many *Brachypelma* males are run over while crossing roads during the mating season. Furthermore, roads facilitate the access to wild subpopulations of tarantulas by poachers, who can collect hundreds of specimens in the same area, severely depleting the subpopulations to meet the illegal pet trade market. This is the case of subpopulations of *B. albiceps* from the Mexican state of Morelos, *B. albopilosum* and *S. lanceolatum* from Nicaragua, *B. auratum* in many Mexican areas and of the subpopulation of *B. hamorii* from the state of Colima in Mexico, from where a disproportionate amount of trafficked specimens of these tarantulas seem to originate.

In general, *Brachypelma* species are prone to local extinction owing to their life history traits such as slow growth to sexual maturity, natural high juvenile mortality rate (Locht et al. 1999, Hénaut et al. 2015) and vulnerability to habitat loss (Turner et al. 2018). The Red Leg lineage encompasses the species with higher conservation concerns according to the IUCN criteria, but the Red Rump group also presents species in threatened categories, such as *B. schroederi*. However, as pointed by Turner et al. (2018), the Red Rump group is a distinct evolutionary lineage and it is going to be accommodated in a new genus (Mendonza and Francke in press). Thus, due to their different evolutionary history, taxonomic uniqueness and threatened status, both groups could be considered as having high priority in conservation, different from the proposal of downgrading the conservational status the Red Rump group made by Turner et al. (2018).

### Conservation actions

The most important conservation actions identified across species include preserving their natural habitat through protected areas, establishing management plans for both the species and their habitats and undertaking systematic monitoring to provide information about population recovery and species re-introduction programmes. Areas covered by tropical dry forest can be considered priority, given that these are where more than half of the assessed species are found, including the seven most threatened species.

Special attention regarding conservation actions and research plans has to be given to the central Pacific coastal area of Mexico, particularly around Guerrero State where five species of *Brachypelma* occur (*B. smithi*, *B. verdezi*, *B. baumgarteni*, *B. boehmei* and *B. hamorii*). Urbanisation and related transport infrastructure are rapidly expanding and either threaten or have already depleted several subpopulations. In particular, the port city of Acapulco is continuing to expand into new urban areas in the foothills, as well as expansion of several other neighbouring coastal towns often depending on tourism. Finally, there are few officially protected natural areas in this part of the country. Therefore, it is a priority geographic area regarding conservation planning and actions for the genus.

Critically, for some of the most endangered species, such as *B. baumgarteni* and *B. hamorii*, there is no officially protected area in their range of occurrence. It would therefore be highly recommended to establish at least one conservation unit which focuses on protecting each of these species *in situ.* For other species, where some subpopulations can already be found in protected areas, such as *B. vagans* and *B. klaasi*, these areas can be suitable for developing conservation initiatives, including the confirmation of population, harvest and their trade trends and how the latter affect the population sustainability.

Besides the central Pacific coastal area of Mexico, some other areas can be considered relevant to *Brachypelma* conservation. The states of Morelos and Colima are important regions for establishing conservation actions to *B. albiceps* and *B. hamorii*, respectively. On the other hand, the areas around Chihuahua City in Mexico and the cities of Managua in Nicaragua and Filadelfia de Guanacaste in Costa Rica are relevant to *A. pallidum* and *S. lanceolatum*, respectively and need to be protected from the fast expansion of urbanisation and agriculture.

Currently, some 13 species of *Brachypelma* are being reared in captivity in at least one Mexican Unit for Management to meet the legal trade. Many species have also been bred internationally in captivity for several years. Successful breeding and marketing of captive-produced *B. smithi* and *B. auratum*, in particular, became more notably successful and popular within the International 'arachnoculture' back in the early-80s. However, there is also an illegal trade of many species, especially to the USA and Europe and demand for more exports from countries of origin still exists. Thus, we propose that the government of Mexico should collaborate with Mexican tarantula breeders to develop a system for certifying the origin of specimens used in UMA breeding programmes and to increase surveillance and control of specimens collected from the wild as breeding stock and those traded. Tax advantages for legal dealers would make their prices more competitive with others in the black market and was the subject of a request made by the Mexican breeders in a Tarantula Trinational Trade and Enforcement Workshop held in the beginning of 2018 in Guadalajara City, Mexico. Captive breeding seems to be a good method to reduce the overharvesting of wild populations. *Brachypelma vagans* is a good example. Its trade seems to be of limited extent and stable for now due, in part, to the relative ease to breed this species in captivity by hobbyists. Therefore, for species with suitable reproductive characteristics, this activity has to be stimulated and supported by the appropriate government agencies in the different countries where the tarantulas occur. In the same way, specimen re-introductions into the wild, such as those being planned by Mexican UMAs for *B. klaasi*, *B. smithi*, *B. verdezi* and *B. boehmei* should also be encouraged by authorities, prioritising, for future re-introduction programmes, species whose subpopulations are known to be severely depleted or fragmented.

The Mexican Management Units (UMAs) can also play an important role in conservation besides supplying the market with captive-bred animals and potentially playing vital roles in re-introduction programmes. They should promote educational and awareness activities about tarantula spiders aimed at both students and the general public. In order to motivate public involvement in tarantula conservation efforts, one or more species of *Brachypelma* could be used as flagship species. *Brachypelma albiceps* seems to be a good candidate for central Mexico since it meets many criteria of ideal flagship species: it is endemic to the region, presents economic importance to the area and its population is in decline (Smith and Sutton 2008). The same could be done with *B. epicureanum* in the Yucatán peninsula and with *B. boehmei* in the coastal Pacific area of Mexico. Another possibility would be to flag a single species, such as the widely recognisable *B. smithi* to be used as a flagship species for the whole country.

### Research needed

Different research priorities are suggested across species. In general, we propose to prioritise and support research on the population trends and distribution, as well as on the impact of land use and habitat degradation on those. As mentioned previously, the central Pacific coast is one of the most human-disturbed areas of Mexico and encompasses several different species of *Brachypelma*. Therefore, it is a priority area regarding conservation planning and actions for the genus.

In the case of *B. vagans*, where the ecological features are one of the best known amongst tarantulas at a handful of well-studied sites, it is necessary to re-evaluate the ecological and genetic situation over its whole geographic range, in order to access the real conservation status of the species (Machkour-M’Rabet et al. 2012). In some cases, such as *B. andrewi*, *B. aureoceps* and *B. sabulosum*, as well as *Sericopelma* spp., basic taxonomic research is needed before development of any appropriate conservation action can be proposed.

Another point to have in consideration is the lack of information about the occurrence, status, trends and management of populations of CITES-listed tarantulas and their legal and illegal trade in other countries, apart from Mexico where those species occur. The knowledge of this basic information is vital to establish suitable conservation actions that could include a collaborative network encompassing all the countries from Mexico to Panama in order to protect strategic natural areas and to curb more efficiently the traffick across borders.

Despite *Brachypelma* being one of the most traded tarantula genera in the world and with *B. smithi* being so well known by the general public, there are relatively few detailed scientific studies on any of the species of this genus. The current situation is even worse for the less popular genera such as *Sericopelma*. This scenario is changing, at least for *Brachypelma*, since a taxonomic review is being carried out, with many new field records added to the few previous ones, shedding some light on these high-profile tarantulas both in Mexico and Central America. However, basic knowledge is still missing for many tarantula species throughout the world and financial support for initiatives that aim to solve taxonomic, ecological and conservation challenges are vital to effectively preserve the group for the foreseeable future (Laverde-R and Cadena 2014, Costello et al. 2015, Dubois 2003).

## Supplementary Material

B9A9C5B6-CA5D-5702-9B82-8DEEB3D6F4DB 10.3897/BDJ.7.e39342.suppl1 Supplementary material 1 Distribution of *Aphonopelma pallidum* (F. O. Pickard-Cambridge, 1897) Data type: Distribution
Brief description: Species Distribution of *Aphonopelma pallidum* (F. O. Pickard-Cambridge, 1897)
File: oo_321379.kml https://binary.pensoft.net/file/321379 Cardoso, P.

07A115C8-CC9E-512E-99FE-43477EA265D7 10.3897/BDJ.7.e39342.suppl2 Supplementary material 2 Distribution of *Brachypelma albiceps* (Pocock, 1903) Data type: Distribution
Brief description: Species distribution of *Brachypelma albiceps* (Pocock, 1903).
File: oo_321380.kml https://binary.pensoft.net/file/321380 Cardoso, P.

3BE0FB8C-FB45-5799-BD67-F1FD43754BBE 10.3897/BDJ.7.e39342.suppl3 Supplementary material 3 Distribution of *Brachypelma albopilosum* Valerio, 1980 Data type: Distribution
Brief description: Species distribution of *Brachypelma albopilosum* Valerio, 1980.
File: oo_321381.kml https://binary.pensoft.net/file/321381 Cardoso, P.

0F5140AC-89D7-5A64-95CE-54D76AB6C913 10.3897/BDJ.7.e39342.suppl4 Supplementary material 4 Distribution of *Brachypelma andrewi* Schmidt, 1992 Data type: Distribution
Brief description: Species distribution of *Brachypelma andrewi* Schmidt, 1992.
File: oo_321382.kml https://binary.pensoft.net/file/321382 Cardoso, P.

EA130F3C-88CD-5764-BD41-3650F514FAFF 10.3897/BDJ.7.e39342.suppl5 Supplementary material 5 Distribution of *Brachypelma auratum* Schmidt, 1992 Data type: Distribution
Brief description: Species distribution of *Brachypelma auratum* Schmidt, 1992.
File: oo_321383.kml https://binary.pensoft.net/file/321383 Cardoso, P.

7811645C-C562-5007-86FC-1667173B2B2B 10.3897/BDJ.7.e39342.suppl6 Supplementary material 6 Distribution of *Brachypelma aureoceps* (Chamberlin, 1917) Data type: Distribution
Brief description: Species distribution of *Brachypelma aureoceps* (Chamberlin, 1917)
File: oo_321384.kml https://binary.pensoft.net/file/321384 Cardoso, P.

9488D6A4-3288-5C56-AA88-3C56CCA0089F 10.3897/BDJ.7.e39342.suppl7 Supplementary material 7 Distribution of *Brachypelma baumgarteni* Smith, 1993 Data type: Distribution
Brief description: Species distribution of *Brachypelma baumgarteni* Smith, 1993.
File: oo_321385.kml https://binary.pensoft.net/file/321385 Cardoso, P.

8C214712-B716-5743-AFC6-183EB51A922F 10.3897/BDJ.7.e39342.suppl8 Supplementary material 8 Distribution of *Brachypelma boehmei* Schmidt & Klaas, 1993 Data type: Distribution
Brief description: Species distribution of *Brachypelma boehmei* Schmidt & Klaas, 1993.
File: oo_321386.kml https://binary.pensoft.net/file/321386 Cardoso, P.

6B67DEE2-94B7-584A-A4BE-DAC440606D48 10.3897/BDJ.7.e39342.suppl9 Supplementary material 9 Distribution of *Brachypelma emilia* (White, 1856) Data type: Distribution
Brief description: Species distribution of *Brachypelma emilia* (White, 1856)
File: oo_321387.kml https://binary.pensoft.net/file/321387 Cardoso, P.

50FCC037-03F4-58AE-A2FC-3B19FF783936 10.3897/BDJ.7.e39342.suppl10 Supplementary material 10 Distribution of *Brachypelma epicureanum* (Chamberlin, 1925) Data type: Distribution
Brief description: Species distribution of *Brachypelma epicureanum* (Chamberlin, 1925).
File: oo_321388.kml https://binary.pensoft.net/file/321388 Cardoso, P.

B9CEA293-97CD-520A-A0AC-68DB80D02F26 10.3897/BDJ.7.e39342.suppl11 Supplementary material 11 Distribution of *Brachypelma hamorii* Tesmoingst, Cleton & Verdez, 1997 Data type: Distribution
Brief description: Species distribution of *Brachypelma hamorii* Tesmoingst, Cleton & Verdez, 1997.
File: oo_321390.kml https://binary.pensoft.net/file/321390 Cardoso, P.

2F094FB5-C90F-59E2-A1E8-683DEF80287B 10.3897/BDJ.7.e39342.suppl12 Supplementary material 12 Distribution of *Brachypelma kahlenbergi* Rudloff, 2008 Data type: Distribution
Brief description: Species distribution of *Brachypelma kahlenbergi* Rudloff, 2008.
File: oo_321391.kml https://binary.pensoft.net/file/321391 Cardoso, P.

7DF38B48-B393-589C-B56C-85BA8BAC3955 10.3897/BDJ.7.e39342.suppl13 Supplementary material 13 Distribution of *Brachypelma klaasi* (Schmidt & Krause, 1994) Data type: Distribution
Brief description: Species distribution of *Brachypelma klaasi* (Schmidt & Krause, 1994).
File: oo_321392.kml https://binary.pensoft.net/file/321392 Cardoso, P.

C2F2D264-49DE-581E-A6E1-4B20F7684D70 10.3897/BDJ.7.e39342.suppl14 Supplementary material 14 Distribution of *Brachypelma sabulosum* (F. O. Pickard-Cambridge, 1897) Data type: Distribution
Brief description: Species distribution of *Brachypelma sabulosum* (F. O. Pickard-Cambridge, 1897).
File: oo_321393.kml https://binary.pensoft.net/file/321393 Cardoso, P.

FD38401C-66EB-5335-AB4C-9BA160A285EC 10.3897/BDJ.7.e39342.suppl15 Supplementary material 15 Distribution of *Brachypelma schroederi* Rudloff, 2003 Data type: Distribution
Brief description: Species distribution of *Brachypelma schroederi* Rudloff, 2003
File: oo_321395.kml https://binary.pensoft.net/file/321395 Cardoso, P.

66D7C2FE-05E0-5A4B-A308-6ADA74285399 10.3897/BDJ.7.e39342.suppl16 Supplementary material 16 Distribution of *Brachypelma smithi* (F. O. Pickard-Cambridge, 1897) Data type: Distribution
Brief description: Species distribution of *Brachypelma smithi* (F. O. Pickard-Cambridge, 1897).
File: oo_321396.kml https://binary.pensoft.net/file/321396 Cardoso, P.

F88134E9-DB7B-537A-BABD-0C94DA69A515 10.3897/BDJ.7.e39342.suppl17 Supplementary material 17 Distribution of *Brachypelma vagans* (Ausserer, 1875) Data type: Distribution
Brief description: Species distribution of *Brachypelma vagans* (Ausserer, 1875).
File: oo_321397.kml https://binary.pensoft.net/file/321397 Cardoso, P.

7216B418-00C6-5BC0-B3EF-1C3D21701EA9 10.3897/BDJ.7.e39342.suppl18 Supplementary material 18 Distribution of *Brachypelma verdezi* Schmidt, 2003 Data type: Distribution
Brief description: Species distribution of *Brachypelma verdezi* Schmidt, 2003.
File: oo_321398.kml https://binary.pensoft.net/file/321398 Cardoso, P.

392D5B25-316A-580F-8380-7F38280C5AB8 10.3897/BDJ.7.e39342.suppl19 Supplementary material 19 Distribution of *Sandinista lanceolatum* (Simon, 1891) Data type: Distribution
Brief description: Species distribution of *Sandinista lanceolatum* (Simon, 1891).
File: oo_327101.kml https://binary.pensoft.net/file/327101 Cardoso, P.

A924336F-7A20-56AB-8C20-14671C4CF44E 10.3897/BDJ.7.e39342.suppl20 Supplementary material 20 Distribution of *Sericopelma angustum* (Valerio, 1980) Data type: Distribution
Brief description: Species distribution of *Sericopelma angustum* (Valerio, 1980).
File: oo_321399.kml https://binary.pensoft.net/file/321399 Cardoso, P.

A416A99E-5E17-53A7-841B-0832594E8839 10.3897/BDJ.7.e39342.suppl21 Supplementary material 21 Distribution of *Sericopelma embrithes* (Chamberlin & Ivie, 1936) Data type: Distribution
Brief description: Species distribution of *Sericopelma embrithes* (Chamberlin & Ivie, 1936)
File: oo_321400.kml https://binary.pensoft.net/file/321400 Cardoso, P.
